# Automated Parallel Synthesis Accelerates Virtual Screening Hit Discovery

**DOI:** 10.1021/jacs.6c05055

**Published:** 2026-07-10

**Authors:** Sean M. McKenna, Martin Šícho, Cas van der Horst, Jesse Maasland, Edith van der Nol, Gianluca Turco, Andrius Bernatavicius, Gerard J.P. van Westen, Laura H. Heitman, Sebastian J. Pomplun

**Affiliations:** † Leiden Academic Centre for Drug Research, 4496 Leiden University, 2333 CC Leiden, The Netherlands; ‡ Oncode Institute, 3521 AL Utrecht, The Netherlands; § CZ-OPENSCREEN, National Infrastructure for Chemical Biology, Department of Informatics and Chemistry, Faculty of Chemical Technology, University of Chemistry and Technology Prague, Technická 5, 166 28 Prague, Czech Republic

## Abstract

Virtual screening (VS) is a powerful approach to exploring a vast chemical space, encompassing libraries of millions to billions of compounds. However, the low hit rates of VS require testing numerous candidates to validate true binders, followed by iterative optimization cycles, which makes experimental validation costly and time-consuming. Here, we report COMBINAUT, an automated parallel synthesis platform that generates diverse chemical scaffolds to accelerate hit validation and refinement. Using a faculty-wide collection of in-house building blocks, the system enables enumeration of over 22.9 million compounds, each designed for parallelized synthesis within 32 h using repurposed solid-phase peptide synthesis equipment. Using this platform, we performed large-scale VS targeting the allosteric pocket of the immuno-oncology target, C–C chemokine receptor 2 (CCR2). Our approach facilitated the rapid synthesis and testing of 100 VS hits spanning diverse molecular architectures. In radioligand binding assays, we successfully validated nine hits with distinct scaffolds, including completely novel CCR2 ligand chemotypes. Iterative hit-to-lead optimization using the automated workflow produced cell-active CCR2 antagonists. This work demonstrates the synergy of automated synthesis and VS, enabling the efficient exploration of chemical space and the rapid discovery of novel ligands.

## Graphical abstract

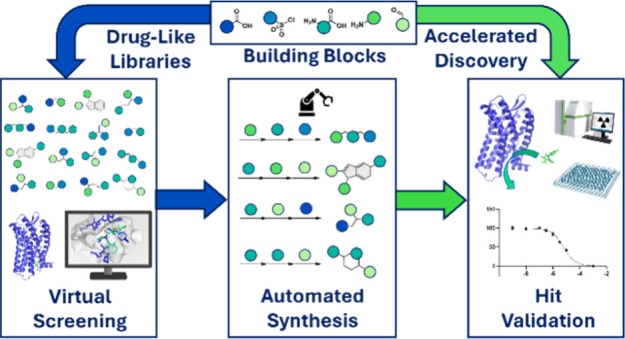

## Introduction

Virtual screening (VS) has become a powerful approach for exploring vast regions of chemical space, enabling the enumeration and prioritization of millions to billions of candidate molecules without the need to physically generate large compound libraries.^1–8^ Advancements in docking,^9–11^ scoring,^12,13^ and machine learning methods,^14–19^ have further expanded the accessibility and scope of VS across a wide range of targets. However, despite these computational capabilities, experimental validation remains a fundamental bottleneck. VS campaigns typically require the synthesis and testing of large numbers of predicted hits to reliably identify true binders, followed by multiple rounds of iterative optimization.^20–23^ The limited throughput and speed of chemical synthesis, therefore, substantially constrain the effectiveness of VS-driven discovery and iterative hit refinement.

Hit validation studies commonly report fewer than 20%, and in many cases below 5%, of VS-predicted candidates correspond to true binders.^24–28^ In two recent crowdsourced benchmarking studies of VS methods, hit-finding performance was evaluated for the Parkinson’s disease target LRRK2 and SARS-CoV-2 helicase Nsp13. While VS informed the testing of nearly 2000 compounds, just 73 hits (3.7%) and 46 hits (2.3%) could be validated against their respective targets.^29,30^ The role of VS in hit discovery is therefore critically dependent on rapid, scalable access to synthesizable compounds.^3,27^

The availability of databases such as ZINC, REAL (Enamine), and Galaxi (WuXi), linked with commercial “make-on-demand” services, has substantially supported developments and applications of VS.^20–22,31,32^ However, the lead time for synthesis and shipping is commonly 4–6 weeks, representing a substantial bottleneck in the design-make-test-analyze (DMTA) cycle, especially when multiple iterations are required for discovery and optimization of validated hits. The cost of acquiring compounds for preliminary studies can also be a significant hurdle, with make-on-demand services costing in excess of €150/mg/compound, bringing the cost of the initial validation of a set of 100 VS hits in excess of €15,000.

Automation offers a powerful strategy to accelerate chemical synthesis and streamline discovery workflows.^33–37^ Automated synthesis of peptide libraries is an expanding field of hit discovery, though transformations are commonly limited to amide coupling chemistries.^38–40^ A wider suite of bond-forming reactions has also been translated to automation, but these methods are dependent upon proprietary or acutely bespoke equipment and are not always compatible with parallelized synthesis.^33–35,37^ While automated synthesis has previously been proposed as a platform to empower VS,^33^ work to date has largely been limited to exploring local chemical space around a known hit.^36,37^ Solid-phase synthesis (SPS) has historically been used as a vehicle to quickly access many compounds via parallel library generation;^34,37,39^ however, to our knowledge, there are no reports systematically applying solid-phase small molecule synthesis for the rapid preparation and validation of diverse VS hits. We therefore proposed the development of a holistic workflow integrating VS with automated SPS to enable discovery of novel hits.

Here, we introduce COMBINAUT, a generalizable platform for multistep parallelized combinatorial automated synthesis to explore drug-like chemical space. We established protocols for a diverse set of automatable transformations that, when combined with a faculty-wide collection of 715 building blocks, enabled the enumeration of a 22.9 million-member virtual library, wherein compounds could be prepared in parallel within 32 h using a repurposed peptide synthesizer. In this study, we prioritized medicinal chemistry “workhorse” transformations (e.g., sulfonylations, reductive aminations, amidations, nucleophilic substitutions, reductions, and heterocyclizations), but in principle, the same approach could be readily extended to additional transformations, substantially expanding rapidly accessible chemical space. Using this platform, we applied docking-based VS against an allosteric pocket of the C–C chemokine receptor 2 (CCR2), performed the automated parallel synthesis of 100 VS hits, and experimentally validated multiple hits spanning diverse chemical scaffolds (Figure 1). Our automated workflow also supported rapid iterative hit optimization, ultimately yielding novel cell-active allosteric CCR2 antagonists with low micromolar to nanomolar activity.

**Figure 1:**
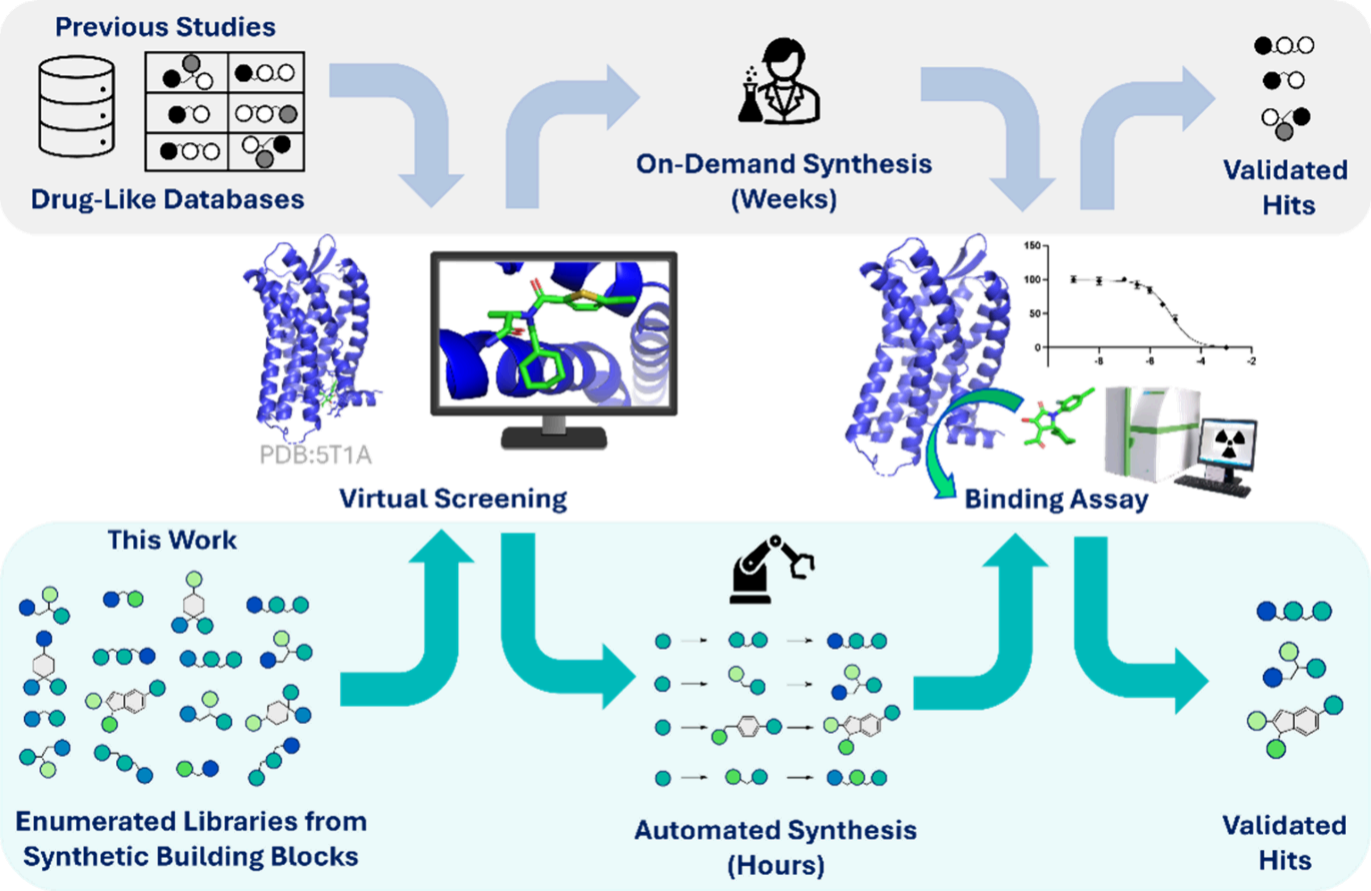
Direct coupling of virtual screening to automated synthesis eliminates reliance on commercial make-on-demand libraries for hit validation. Unlike previous studies leveraging databases of drug-like compounds and requiring commercial make-on-demand services for hit validation, this work evaluated rapidly accessible compound libraries from in-house building blocks enabled by automated synthesis.

## Results and Discussion

### Curation of Building Block Sets

In order to access diverse drug-like chemical space, a set of carboxylic acids, sulfonyl chlorides, Fmoc-protected amino acids, primary amines, and aldehyde building blocks was compiled from several synthetic chemistry laboratories within our institution. Building blocks were curated to exclude duplicate entries, incompatible protecting group and functional group combinations, racemates, and highly bespoke scaffolds. Analysis of these building blocks was performed to evaluate their suitability for the construction of drug-like small molecule libraries (Figure S1). Across building block classes, the majority of entries exhibited desirable “rule of two” properties (H-bond donors ≤2, H-bond acceptors ≤4, *M*_W_ ≤ 200, log *P* ≤ 2), with 85–97% of entries committing one or fewer violations of the rules set out by Goldberg et al.^41^ As per the original study, these criteria were applied predominantly as a guideline for building block curation and were therefore only used to exclude building blocks that were in violation of all rules. This exercise yielded a set of 715 distinct building blocks (Figure 2a).

**Figure 2:**
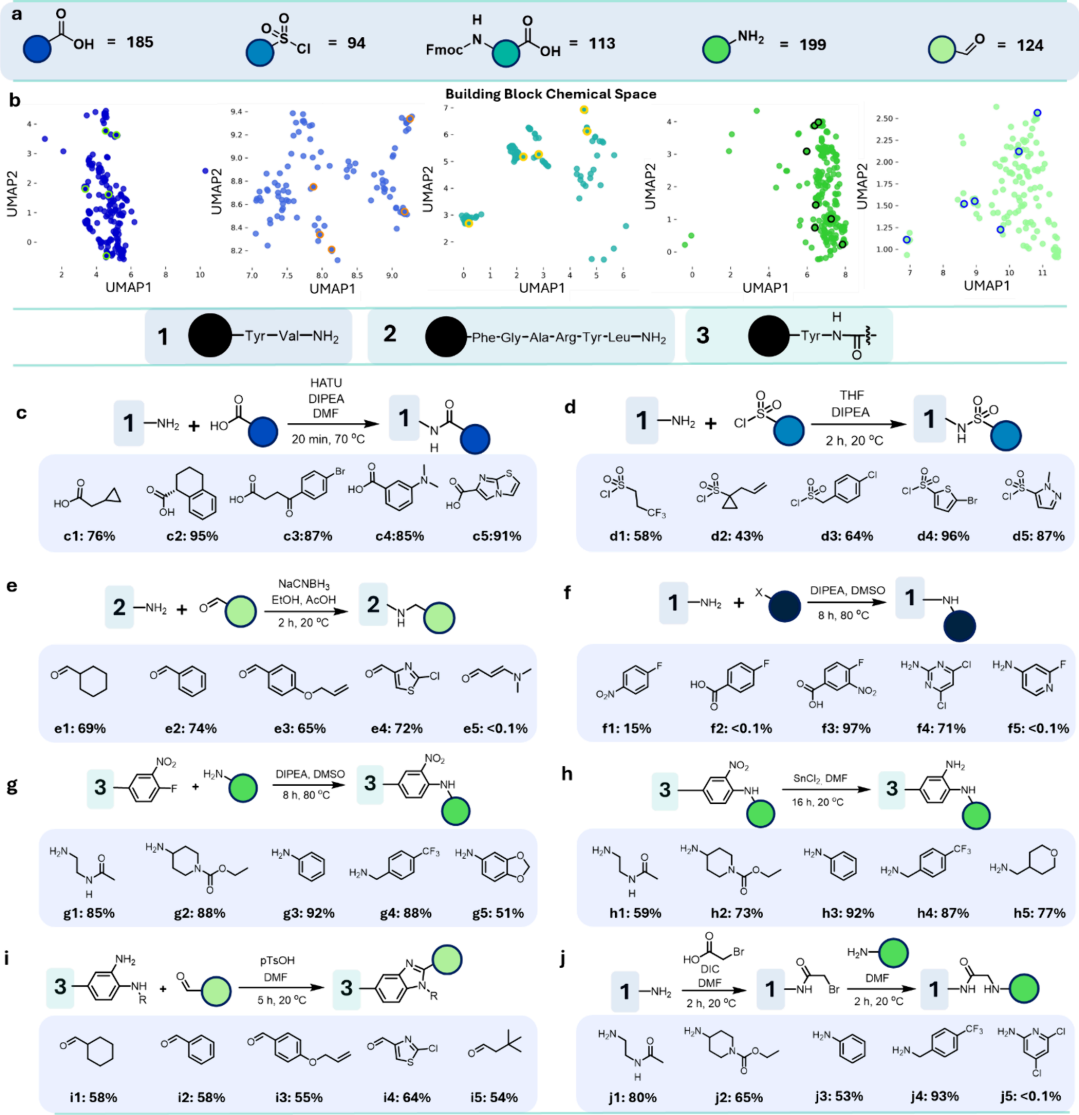
Curated and chemically diverse set of building blocks enables broad reaction coverage for automated synthesis of drug-like libraries. (a) Curated set of 715 in-house building blocks was arranged by functional group, (b) UMAP plots were generated to visualize building block chemical space. A set of representative building blocks (circled) were chosen from each class and applied as part of a focused reactivity scope study on model substrates **1**–**3** to establish conditions for automated synthesis of drug-like libraries; (c) amide coupling, (d) sulfonamide coupling, (e) reductive amination, (f) nucleophilic aromatic substitution (S_N_Ar) with variable electrophiles, (g) nucleophilic aromatic substitution (S_N_Ar) with variable nucleophiles, (h) nitro reduction, (i) heterocyclization, and (j) nucleophilic substitution (S_N_2).

### Development of SPS Methodology

Next, a range of common bond-forming reactions were evaluated for their suitability in an automated parallel synthesis workflow. Reaction methodology development was initially performed using manual SPS as a precursor to automation. Crucially, SPS could enable the preparation of diverse, feature-rich scaffolds through multiple sequential bond-forming reactions, extending beyond the solvent and reagent limitations inherent in direct-to-biology libraries.^42,43^ Among the reactions commonly leveraged in medicinal chemistry, amide coupling, sulfonamide coupling, reductive amination, and nucleophilic substitution were identified for their utility in preparing drug-like scaffolds and their relative tolerance to air and moisture.^44–47^ Uniform manifold approximation and projection (UMAP) was used to visualize the chemical space of each building block class based on high-dimensional descriptors. These plots were used for the selection of representative members for evaluating reaction conditions and substrate scope (Figure 2b).

Given the extensive methodology available for amide coupling reactions in SPS for the preparation of peptides, initial work was performed to evaluate amide coupling chemistry.^48^ HATU was applied as a coupling reagent for the preparation of dipeptide substrate **1**, to which a representative set of carboxylic acids was coupled using HATU. Following cleavage from resin and HPLC-MS analysis, carboxamide products **c1**–**c5** were detected with 76–95% conversion (Figure 2c).

For the preparation of sulfonamides from sulfonyl chlorides with primary or secondary amines, a small reaction screen was first performed to test a range of solvents and bases. Excellent conversion was observed for reactions performed in THF with DIPEA at ambient temperature (Figure S2). Using these conditions, we coupled a representative set of sulfonyl chlorides to dipeptide **1** to give the corresponding sulfonamides **d1–d5** with conversions of 43–96% (Figure 2d).

Next, reductive amination was investigated as a method for obtaining compact trifunctionalized scaffolds around a central nitrogen atom. To streamline the initial condition screening, we performed the reaction on the peptide linker **2**. This strategy enabled precipitation of the reaction products upon cleavage, separating them from the other components of the cleavage eluent. Following a conditions screen, excellent conversion was seen when reactions were performed in ethanol with 1% acetic acid using sodium cyanoborohydride as the reducing agent (Figure S3). In a substrate scope experiment, the majority of aldehydes gave the desired secondary amines in good to excellent conversions of 65–74% for **e1–e4** (Figure 2e). However, no product was observed in the case of **e5**, highlighting the occurrence of incompatibility in some building-block combinations.

Nucleophilic aromatic substitution (S_N_Ar) was next tested as a means for accessing scaffolds around a polyfunctionalized arene core. Dipeptide **1** was tested with a range of electron-poor haloarenes toward the preparation of *N*-substituted anilines (Figure 2f). While good to excellent conversion was seen for **f3** and **f4**, the majority of electron-poor arenes gave little or no conversion. Due to the narrow scope of this reaction, attention was shifted to the application of S_N_Ar with variable amine nucleophiles to access 1,2,5-trisubstituted benzimidazoles.^49^ A range of primary amine-containing building blocks was tested with fluoroarene-bearing model substrate **3**, where modest to excellent conversions of 51–92% were observed for **g1**–**g5** (Figure 2g). Nitro reduction was also found to be broadly tolerant of a range of *N*-substituted anilines **h1**–**h5** with 59–92% conversion (Figure 2h), while condensation with a range of aldehydes produced benzimidazoles **i1**–**i5** with moderate conversions of 54–64% (Figure 2i).

Expanding accessible scaffolds through nucleophilic substitution chemistry, bromoacetic acid was coupled to **1** using DIC, providing an electrophilic handle for reaction with primary amine building blocks.^50^ Secondary amines **j1–j4** were observed to form with 53–93% conversion, though **j5** was not present in detectable quantities, reflective of the low nucleophilicity of some primary amines within the building block set (Figure 2j).

### Application of Automated SPS to Prepare Drug-like Compounds

Automation enables parallelized synthetic throughput and around-the-clock productivity. To evaluate the translatability of established manual SPS methodologies, a selection of synthetically accessible compounds was chosen for preparation by automated SPS (Figure 3). Reaction methodologies were readily adapted for execution on a Biotage Syro I parallel peptide synthesizer; however, we anticipate that instruments with similar liquid handling, mixing, and heating functionality could also be adapted to carry out these reactions, thereby lowering the barrier for adoption of automated synthesis as a tool for other research laboratories.

**Figure 3:**
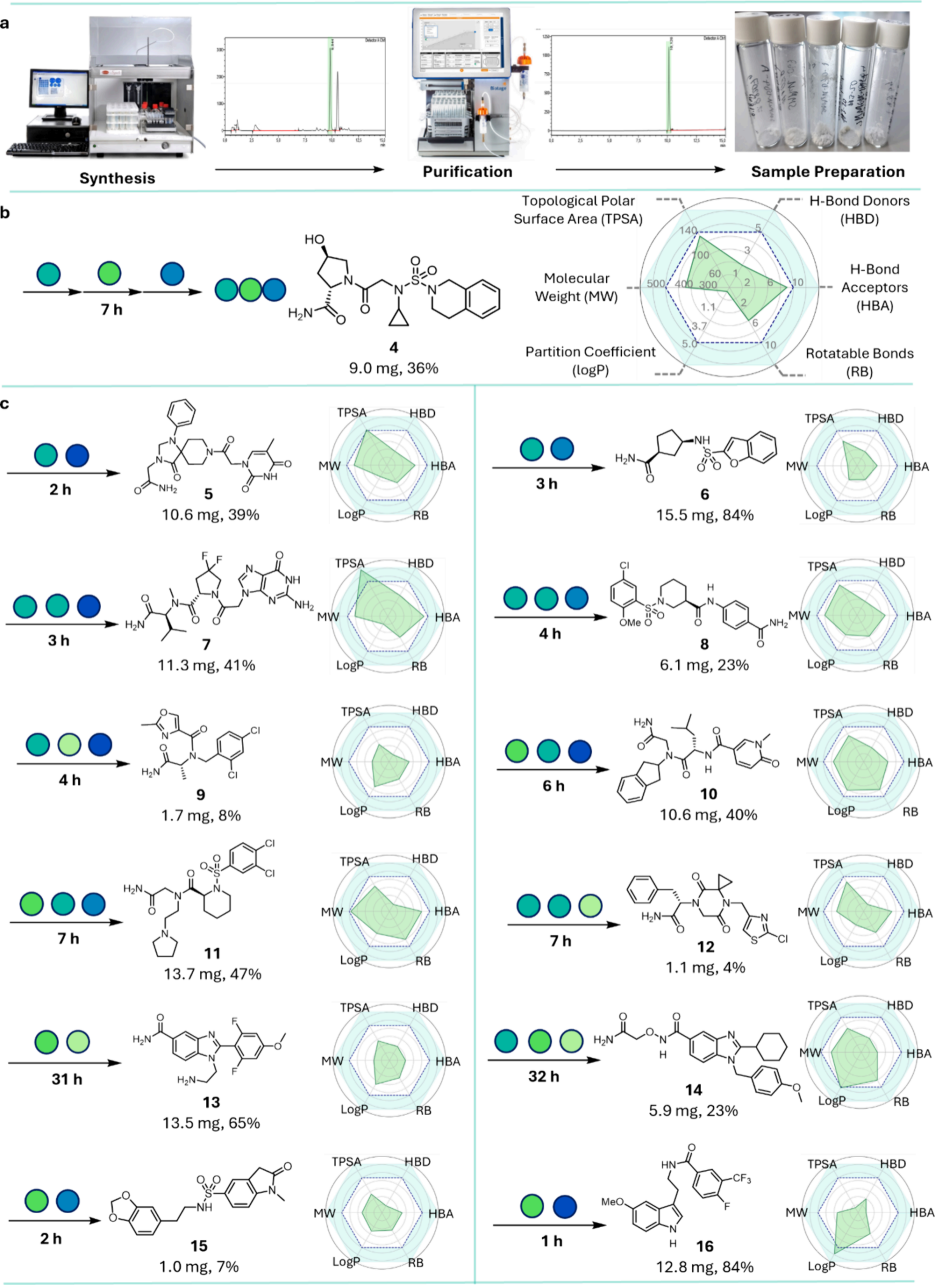
Translation of established SPS methodologies enables a representative set of diverse drug-like molecules to be prepared by automated synthesis. (a) Workflow for automated synthesis, purification, and preparation of compounds for screening and (b) automated synthesis of test compound **4** through sequential amide coupling, nucleophilic substitution, and sulfonamide coupling. Classes of composite building blocks, total reaction time, and mass of isolated product are given. A radar plot depicts calculated compound properties, where the location of vertices indicates compliance (white area) or violation (blue area) with Lipinski and Veber rules for orally bioavailable drugs; (c) automated synthesis of 12 additional test compounds **5**–**16**.

Briefly, stock solutions of building blocks and reagents were prepared in the chosen reaction solvent, while fritted syringes were charged with 60 μmoles of Rink-functionalized ProTide resin. Resin swelling, building block conjugation, resin washing, orthogonal deprotection, and solving switches were all written as custom methods within the existing Syro XP software, such that manual intervention would not be necessary once the synthesis was initiated. Upon completion, synthesized compounds were cleaved from resin under acidic conditions and subjected to a standardized 18 min automated reverse-phase chromatography purification step (Figure 3a). Purification is a common bottleneck in synthetic throughput; however, this was included in the workflow to account for discrepancies in product yield and purity. However, it may be conceivable to perform crude screening for some high percentage conversion libraries. Product fractions were lyophilized, from which samples could be prepared for biological screening. Success criteria for target compound fulfillment was established, where the isolated mass for the product must be ≥ 0.5 mg, while compound purity must be ≥80% based on UV absorption at 254 nm, and a matching mass ion must be found by HPLC-MS analysis.

Pleasingly, the established SPS methodologies translated effectively to automation. Preparation of example compound **4** was achieved in just 7 h by sequential immobilization of (2*S*,4*R*)-4-hydroxypyrrolidine-2-carboxylic acid, coupling of bromoacetic acid, nucleophilic substitution with cyclopropanamine, and sulfonamide coupling with 3,4-dihydroisoquinoline-2­(1*H*)-sulfonyl chloride. After cleavage, 9.0 mg (21 μmoles, 36% yield) of **4** was isolated (Figure 3b). Calculation of compound properties supports that COMBINAUT enables the rapid preparation of a lead-like compound in compliance with Lipinski and Veber rules for orally bioavailable drugs.

To explore the synthetic capabilities of COMBINAUT further, an additional 12 compounds composed of two and three-building-block combinations were prepared as representatives of accessible scaffolds (Figure 3c). Conjugation of amino acids with carboxylic acids or sulfonyl chlorides enabled compounds **5**–**8** to be expediently synthesized in only 2–4 h. Reductive amination chemistry was effectively automated in the preparation of compound **9**, while coupling conditions were adapted to use bromoacetic acid as a bridging motif to the Rink linker, enabling primary amines to be introduced as the first variable building block in the preparation of compounds **10** and **11** in just 6–7 h. Recognizing that bromoacetic acid could also be a valuable electrophilic motif for intramolecular reactions, a small screen was performed to test bases for 1,6 cyclization (Figure S4). NaH in THF was found to be effective for preparing a modest quantity of 2,5-diketopiperazine **12**. Due to the elongated reaction times required for S_N_Ar and nitro reduction, benzimidazole targets **13** and **14** required the longest times for synthesis, with 31 and 32 h, respectively, though this still represented a substantial time saving relative to manual synthesis protocols.

Finally, automating one-step solution-phase amide coupling and sulfonamide coupling reactions was explored. Fritted syringes were replaced with uncapped 2 mL Eppendorf tubes, and the reaction scale was decreased to account for the reduced vessel volume. Test compounds **15** and **16** were prepared in 1–2 h. While these represent trivial transformations for either manual or automated synthesis, validating one-step solution-phase synthesis unlocked additional chemical space for our set of building blocks while also enabling solution-phase and solid-phase reactions to be performed in parallel.

To interrogate the structure of isolated reaction products, ^1^H NMR data were obtained for compounds **4**–**16**. Peaks were analyzed based on chemical shift, splitting pattern, and coupling constants and were found to be in good agreement with the structures of the expected products. Taking ^1^H NMR and HPLC-MS data together, it appeared that our automated platform could effectively apply established transformations to generate a variety of target molecules with good structural fidelity.

With automated SPS demonstrated for the preparation of several representative drug-like compounds, accessible scaffolds were proposed through combinations of established methods (Figure S5), and libraries were enumerated based on available building blocks (Figure 4). This exercise produced a fully enumerated virtual library of 22.9 million compounds, of which 17.3 million (75.5%) adhere to Lipinski rules for drug-like small molecules (Figure S6). Compounds are derived from scaffolds exhibiting Lipinski compliance rates ranging from 60.7 to 99.9% (Figure S7).

**Figure 4:**
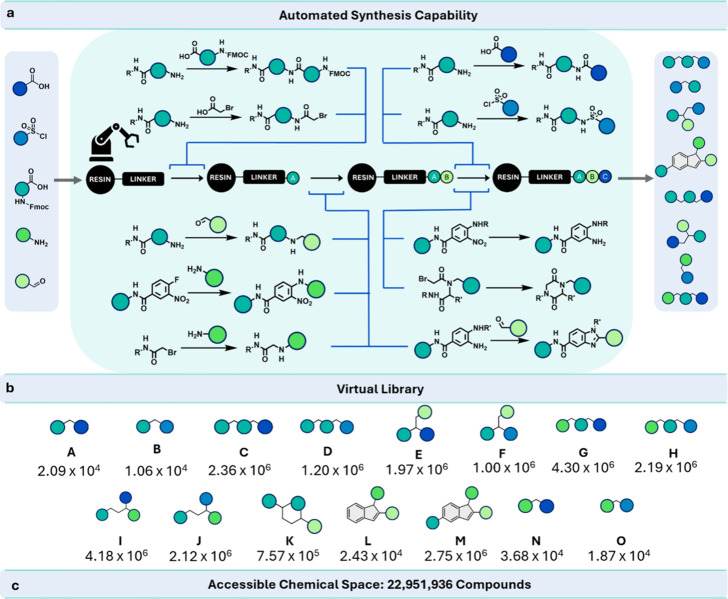
Automated synthesis makes multimillion member chemical space rapidly accessible. (a) Overview of automated synthesis reaction capabilities toward the creation of diverse scaffolds and (b) generic structures of 15 accessible scaffold motifs. Individual scaffold library size (10^4^–10^6^); (c) total accessible chemical space.

### VS Workflow Enables Hit Identification for CCR2 Allosteric Pocket

To evaluate COMBINAUT for the discovery of novel binders, we initiated a VS campaign on the immuno-oncology therapeutic target CCR2. CCR2 is expressed on natural killer cells, dendritic cells, and macrophages, guiding the migration of immune cells to sites of inflammation.^51–53^ Membrane-bound GPCRs such as CCR2 can be poorly amenable to high-throughput screening, while also representing challenging targets for screening using encoded libraries.^49,54^ Therefore, VS offers a vital platform for the discovery of novel ligands for membrane-bound receptor targets.^55–58^

A docking model for the intracellular allosteric pocket of CCR2 was first prepared from a ligand-bound structure reported by Zheng et al. (PDB accession code 5T1A).^59^ A selection of allosteric ligands for CCR2 was extracted from the ChEMBL database and docked using AutoDock VINA. The resulting poses were subsequently analyzed using Protein–Ligand Interaction Profiler (PLIP),^60,61^ which evaluates protein–ligand interactions based on the spatial and angular geometry of the ligand relative to nearby residues. Recurrent interactions observed across multiple ligand series were identified as key binding features and were incorporated into a weighted scoring function. Thus, in combination, both docking score and PLIP fingerprint scores could be used to rank VS hits.

While exhaustive docking analysis of >10^8^ member libraries has been reported, the computational resources required for such a screen render this approach broadly impractical.^3,21,62^ Instead, efficient sampling methods, such as fragment-based VS, synthon-based VS, and machine learning have been applied to screen chemical space representative of >10^10^ drug-like molecules.^6,14,62,63^ Our synthetically accessible 22.9 million member virtual library was sampled using the synthon-based V-SYNTHES method described by Sadybekov et al.^62^ Briefly, representative synthon libraries were prepared by modular combinations of two building blocks, while the third building block was replaced with a placeholder group, e.g., CH_3_ and Gly. All possible building block synthon pairs were enumerated for each accessible scaffold (Figure S9)**.** Two building block scaffolds (**A**, **B**, **L**, **N**, and **O**) were retained intact due to their smaller library sizes. Together, this produced a representative set of 505,832 V-SYNTHES compounds for initial docking using AutoDock VINA and interaction fingerprint scoring using PLIP (Figure 5a). For V-SYNTHES hits containing a placeholder group, compounds were enumerated with a third building block and redocked and scored. Overall, this approach represented a 30-fold improvement in computational throughput relative to brute-force docking. The resulting hits were clustered based on structural similarity, and ∼2000 VS hits were subjected to visual inspection of docked poses. Manual selection was necessary at this stage, as docking and PLIP scoring did not fully exclude poor-quality poses or adequately penalize polar insertions into apolar regions. This intervention is consistent with previous reports, which highlight the importance of visual inspection for quality control in VS workflows.^64^ 371 VS hits were shortlisted following this process, from which 100 representative members were selected for automated synthesis using COMBINAUT.

**Figure 5:**
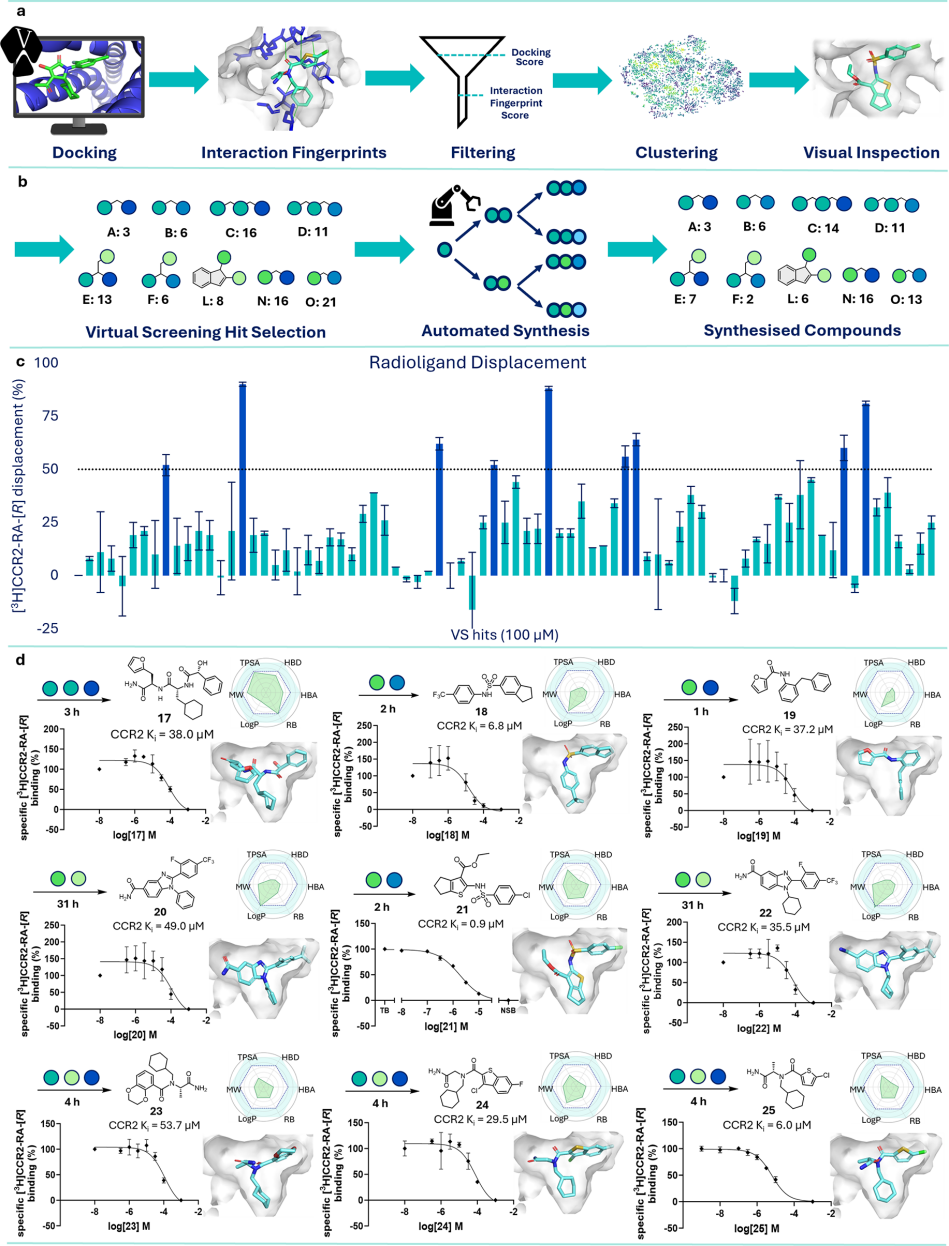
Automated synthesis enables rapid validation of diverse VS hits. (a) Docking-based VS workflow for identification of novel ligands for CCR2. V-SYNTHES libraries were filtered based on docking score, PLIP score, and novelty. Hits were clustered and docked poses were visually inspected, (b) compounds selected for synthesis and fulfillment of purified compounds following automated parallel synthesis, as grouped by number of scaffold representatives, (c) radioligand displacement assay with VS hit compounds tested at 100 μM vs radioligand [^3^H]­CCR2-RA-[*R*] in the presence of CCR2-overexpressing U2OS cell membranes, (d) structure of nine validated VS hits, with composite building blocks, reaction time, drug-likeness radar plots, and docked pose. For characterization of *K*_i_ values, [^3^H]­CCR2-RA-[*R*] displacement was measured with increasing concentrations of compounds **17**–**25** in U2OS-CCR2 at 25 °C. Data are normalized to specific binding in the absence of compound (set as 100%), and presence of excess competing reference ligand (set at 0%). Curves were obtained from nonlinear regression fitting.

The 100 VS hits were composed of members of 9 of the 15 screened scaffolds (Figure 5b). Automated parallel synthesis was initiated, and semiautomated purification of crude reaction mixtures resulted in 78 compounds 78% fulfilling the success criteria (Figure S10). To accelerate the make-test phase, compounds were progressed based on matching HPLC–MS data alone, with detailed characterization of the most promising binders deferred to a later stage of hit discovery.

While the majority of VS hits were prepared successfully, fulfillment within some scaffolds was less consistently achieved. The source of this low conversion appears to be the result of some incompatible aldehyde and amino acid building block combinations for reductive aminations (scaffolds **E** and **F**), and the application of poorly nucleophilic primary amines to participate in S_N_Ar (scaffold **L**) and sulfonamide coupling (scaffold **O**) reactions. Overall, the success rate of the resynthesis compares well to the rates of on-demand synthesis of catalogue compounds from commercial databases.^31,32^

### COMBINAUT Platform Leads to Nine Experimentally Validated VS Hits Spanning Diverse Molecular Scaffolds

With 78 compounds prepared from VS hits, a competition assay with a radioligand was performed to validate binding to the allosteric pocket of CCR2.^65^ Compounds were screened at 100 μM in a radioligand displacement assay with [^3^H]­CCR2-RA-[*R*], and those exhibiting ≥50% displacement of the radioligand were classified as validated hits (Figure 6b). Nine compounds matched these criteria, corresponding to a 12% hit rate. Validated hits consisted of representatives of five distinct scaffolds (**C**, **E**, **L**, **N**, and **O**), with a slight enrichment of two building block combinations (56%) over three building block combinations (44%), reflecting the size constraints of the allosteric pocket of CCR2. Hits were obtained directly from both automated SPS (**17**, **20**, and **22**–**25**) and automated solution-phase synthesis (**18**, **19**, and **21**), highlighting the complementary value of these approaches in accessing chemical space. The presence of such diverse chemotypes within the validated set points to the potential for identifying novel binding interactions, which could be leveraged in future studies on C–C chemokine receptor homologue subtype selectivity.

**Figure 6:**
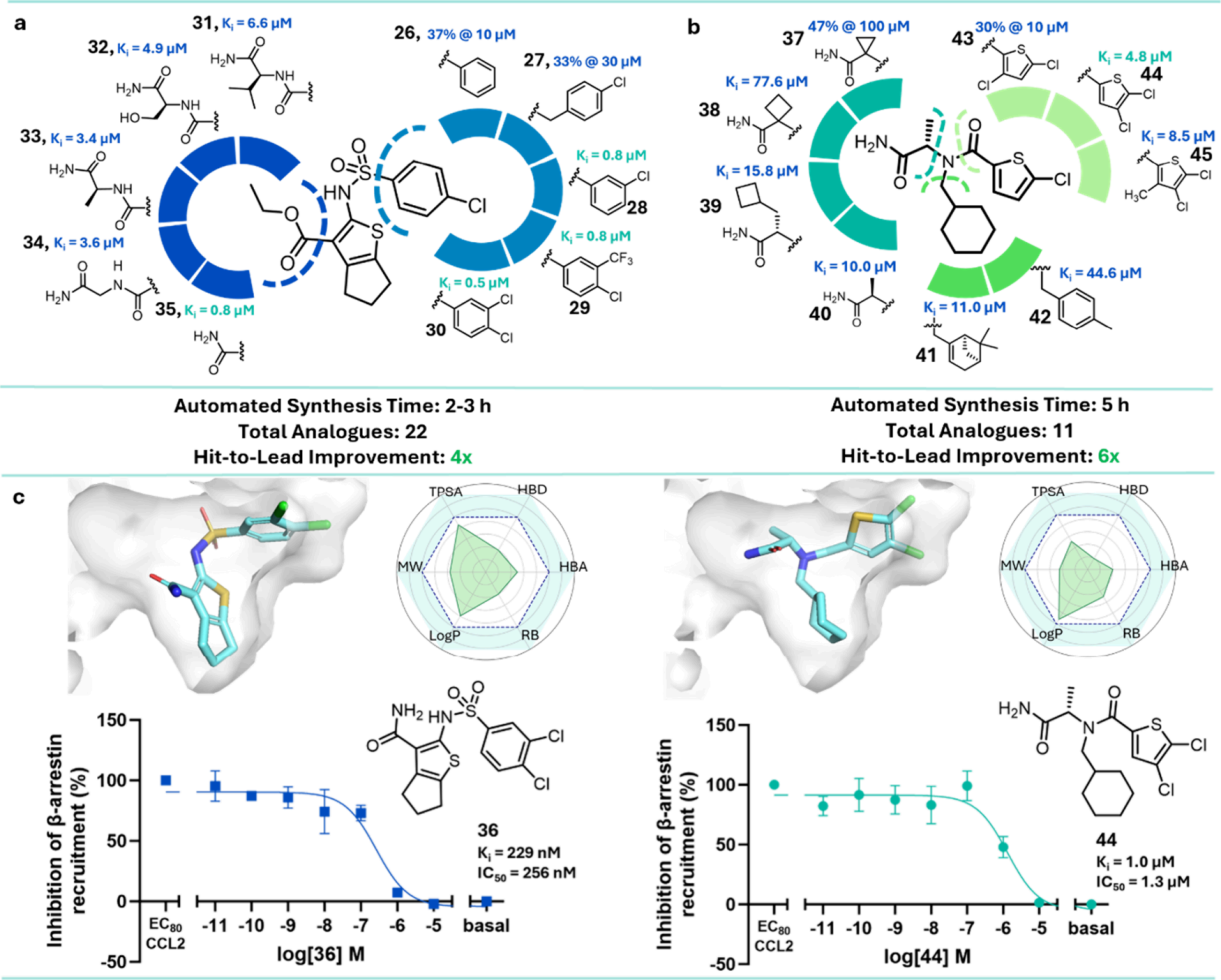
Optimization of VS hits into cell-active CCR2 antagonists is accelerated by automated synthesis. (a) [^3^H]­CCR2-RA-[*R*] displacement by increasing concentrations of **26**–**35** in U2OS-CCR2 at 25 °C, leading to identification of optimized hit **36**, (b) [^3^H]­CCR2-RA-[*R*] displacement by increasing concentrations of **37**–**45** in U2OS-CCR2 at 25 °C, leading to identification of optimized hit **44**, and (c) inhibition of CCL2 stimulated β-arrestin recruitment in U2OS-CCR2 cells by increasing concentrations of compounds **36** and **44**, after stimulation with an EC_80_ concentration of CCL2 (set as 100%).

To explore validated VS hits further, *K*_i_ values were determined for each compound (Figure 5). Six of the nine hits (67%) exhibited double-digit micromolar binding affinities for the allosteric pocket of CCR2 (*K*_i_ = 29.5–53.7 μM), while **18** and **25** displayed single-digit micromolar binding affinities (*K*_i_ = 6.8 μM and *K*_i_ = 6.0 μM, respectively), and **21** exhibited submicromolar binding (*K*_i_ = 0.9 μM). Notably, **21** contains a chlorophenyl sulfonamide group, previously found as a motif in allosteric binders of multiple C–C chemokine receptors; however, its overall structure remains distinct from known ligands (Tanimoto score based on Morgan fingerprints, radius 2 = 0.30). This suggests that our screening approach is capable of identifying compounds that retain key binding features while accessing novel chemical space. The small number of validated, high potency hits is partially attributable to the size of the virtual library, as larger compound databases have been reported to deliver a greater proportion of high-quality hits.^1^ This outcome must also be considered in the context of the intrinsic challenges associated with targeting a small and conformationally restricted allosteric pocket. Nonetheless, constraining an initial VS round to a library of rapidly synthesizable compounds represents a reasonable trade-off, enabling faster interrogation of target engagement and clearer focus for subsequent VS campaigns.

In addition to the notable diversity within the hit set, the novelty of the binders from previously reported CCR2 allosteric antagonists was significant, with Tanimoto similarity scores ranging from 0.15 to 0.30 (Figure S11). Evaluation of compound properties also highlights trends within the hit set, including that the majority of validated VS hits are low molecular weight (median *M*_W_ = 382 Da), corresponding with few rotatable bonds (median RBC = 5) and low topological polar surface area (median TPSA = 82 Å). Hits contain relatively few H-bond donors (median HBD = 1) and acceptors (median HBA = 4); thus, compounds tend toward higher lipophilicity (median XlogP = 3.4). These patterns mimic the properties of reported allosteric CCR2 antagonists (Figure S12).

### COMBINAUT Enables Rapid Optimization of VS Hits into Cell-Active CCR2 Antagonists

Validated hits identified in a first round of a VS campaign are valuable tools to inform additional docking and iterative optimization efforts toward the identification of improved ligands.^29,30,55^ COMBINAUT appeared to be a highly practical tool to support the rapid optimization of VS hits **21** and **25**; thus, a focused hit optimization campaign was initiated.

Based on docked poses, the sulfonamide of compound **21** fulfilled H-bond acceptor interactions with the backbones of Lys311 and Phe312, orienting the phenyl group to participate in a network of π-interactions with Tyr305, Phe312, and Tyr315 (Figure S13a). The 4-chloro substituent was understood to make a putative halogen bonding interaction with the carbonyl backbone of Val63. Automated synthesis was initiated to investigate the replacement of the 4-chlorophenyl ring via coupling of the corresponding sulfonyl chloride. Changes to ring composition (**46,** Figure S15) and substitution (**47–50,** Figure S15) were moderately tolerated; however, the introduction of bulkier substituents (**51**, Figure S15), bridging groups (**27**), or the absence of a substituent (**26**) was poorly tolerated. Improvements to binding affinity were observed for meta-substituted compound **28** and disubstituted compounds **29** and **30** (Figure 6a) as a result of increased hydrophobic interactions with Leu67.

The cyclopentathiophene ring of **21** was observed to make hydrophobic contacts with Leu81, Leu134, and Ile245. Notably, this modality appeared to more effectively exploit the lipophilic pocket of CCR2 than the benchmark ligand CCR-RA-[*R*] (Figure S13b). Meanwhile, the ester carbonyl of **21** was oriented toward the solvent-exposed Arg138, while the ethyl ester tail offered potential interactions with Val244, Ala241, and Glu310. To exploit additional interactions in this region, an Fmoc-protected cyclopentathiophene amino acid building block was prepared to create a second point of diversity. Several analogues **31**–**34** and **52–56** (Figure S15) containing an additional amino acid building block were prepared, but these modifications were found to have a detrimental effect on binding in all instances. However, the truncated analogue **35** gave a minor boost in potency over the original hit, indicating that the ethyl group of **21** did not make a significant contribution to the binding affinity. Interestingly, docked poses for this series position the carbonyl motif roughly equivalent to the acetyl group of CCR2-RA-[*R*] in the ligand-bound crystal structure of CCR2 (PDB: 5T1A). This supports the hypothesis that a carbonyl group in this region can serve as a H-bond acceptor to Arg138 (Figure S14).^66^ Following automated synthesis of a small set of matched pair analogues, optimized hit **36** was identified, resulting from the automated synthesis and screening of 22 analogues (Figure S15), requiring only 2–3 h per round of parallel synthesis.

The docked pose of **25** revealed that for this validated hit, the tertiary amide carbonyl served as the H-bond acceptor to the backbone of Lys311 and Phe312, while the 2-chlorothiophene ring participates in π-bonding and halogen bonding interactions analogous to compound **21** (Figure S16a). The methylcyclohexane group is orientated into the lipophilic pocket, achieving similar contacts to the equivalent group in CCR2-RA-[*R*] (Figure S16b). Meanwhile, the alanine residue makes a hydrophobic interaction with Val244, while the terminal amide was orientated toward solvent, enabling possible water-mediated H-interactions with Lys311 and Arg138.

Automated synthesis was first focused on viable replacements for l-alanine; however, the inclusion of larger substituents, or those presenting additional H-bonding groups to nearby residues, was found to be detrimental (**37**–**40 and** **57–58**). A small number of methylcyclohexane replacements were also prepared (**41**–**42** and **59**), but these modifications were detrimental to target binding (Figure 6b). The observed binding affinity improvement previously observed for disubstituted rings **29** and **30** motivated us to enrich our suite of carboxylic acid building blocks with a small set of commercially available dichlorothiophenes. Analogues **43**–**45** were prepared by automated synthesis, and **44** gave an improved binding affinity, attributed to improved lipophilic binding with Leu67. Our automated synthesis-enabled study resulted in the preparation of 11 new analogues of **25** (Figure S15), requiring just 5 h of parallelized synthesis time.

Due to our focus on the application of COMBINAUT to accelerate hit validation and optimization, we had maintained our success criteria for all screening compounds (≥80% purity at 254 nm by HPLC-MS) to this stage. In order to verify the structure of our optimized binders, we prepared new, high-purity (≥98.0%) batches of compounds **36** and **44** and characterized the products using 1D and 2D NMR. We also screened our high-purity compounds by radioligand displacement assay, revealing good agreement between the calculated binding affinity of the moderate and high-purity samples. **36** showed excellent correlation between the two batches (≥80%, *K*_i_ = 204 nM and ≥98.0%, *K*_i_ = 229 nM), while a stronger binding affinity was measured for **44** (>98.0%, *K*_i_ = 1.0 μM) relative to the lower purity sample (≥80%, *K*_i_ = 4.8 μM). These results support that striking a balance in favor of throughput over purity empowers rapid hit validation and SAR interrogation. Our streamlined workflow resulted in 4- and 6-fold improvements in binding affinity, going from VS hits **21** and **25** to our optimized compounds **36** and **44**, respectively.

With optimized hits **36** and **44**, we moved on to evaluate whether our novel binders could antagonize CCL2-CCR2 signaling in the cellular environment. For this, we performed a Tango β-arrestin recruitment assay in U2OS cells overexpressing CCR2.^67^ Cells were incubated with the putative antagonists prior to stimulation with CCL2. β-arrestin recruitment was then assessed by quantifying fluorescence from a transcriptional reporter gene. Pleasingly, both optimized hits exhibited functional inhibition of β-arrestin recruitment (Figure 6c). **36** gave an IC_50_ = 256 nM, closely aligning with the binding affinity data measured in radioligand displacement. **44** inhibited β-arrestin recruitment with an IC_50_ = 1.3 μM, again, closely correlating with our binding data. Taken together, these results demonstrate that automated synthesis can be an effective tool not just to empower VS but also to accelerate hit optimization for the preparation of cell-active therapeutic modulators.

## Conclusions

Here, we have demonstrated the power of automated synthesis to empower VS-based hit discovery and optimization, substantially accelerating the DMTA cycle in early drug discovery. In establishing protocols for automated SPS of several commonplace medicinal chemistry transformations without the need for highly bespoke or proprietary equipment, we propose supporting the democratization of automated synthesis as an asset in early drug discovery.

We describe the application of eight transformations to the preparation of 15 readily accessible drug-like scaffolds, from which over 22.9 million compounds can be prepared in a maximum of 32 h using 715 building blocks, all available in-house. Our rapidly accessible compound library was applied in a docking-based VS campaign against the immuno-oncology target CCR2, from which 100 VS hits were selected for automated synthesis. Nine of 78 successfully prepared compounds were validated by radioligand displacement assay, spanning double-digit micromolar to submicromolar binders. Automation was then applied to the optimization of two validated hits, resulting in higher affinity binders that exhibited functional antagonism of CCL2-CCR2 signaling in a β-arrestin recruitment assay.

Although the transformations employed in this study are relatively straightforward, they already provide access to multiple diverse scaffolds and functionalities commonly found in approved drugs or compounds under development. We further envision that a broad range of additional chemical transformations could be incorporated into our automated platform without major technical hurdles. Among the most accessible options, several cross-coupling reactions have been established for solid-supported chemistry and could substantially expand the accessible drug-like chemical space. Likewise, carbonate and carbamate formations, as well as copper-catalyzed click reactions, would be directly applicable and enable more diverse connectivity between building blocks. Additional SPS-compatible heterocyclization reactions could include, among others, thiazole and oxadiazole formations,^68^ as well as Pictet–Spengler reactions and their variants.^69^

The chemical space generated using our fully in-house building block collection and a selected set of transformations comprises 22.9 million enumerated compounds. While this library size enabled a successful VS campaign and the discovery of several diverse chemotypes targeting the allosteric site of CCR2, we acknowledge that access to a less-restricted chemical space could yield even more powerful outcomes. Indeed, exploration of billion-membered libraries has been shown to increase the probability of identifying highly potent hits.^1^ Nevertheless, we believe that the diversity afforded by our approach is well-suited for early stage investigations and represents an ideal first-pass VS strategy. As demonstrated here, the platform also supports accelerated hit-to-lead development, which could be combined with iterative docking cycles. Moreover, positive and negative data generated from COMBINAUT validation assays could be used to inform subsequent VS campaigns on even larger libraries, potentially improving hit rates and making the costs and timelines of commercial on-demand libraries more justifiable.

In addition to VS, COMBINAUT could, in principle, also be applied to hit exploration from other combinatorial discovery platforms such as DNA-encoded libraries (DELs). DEL selection workflows typically yield multiple hits but also suffer from substantial false positive rates, making hit validation a major bottleneck. As such, libraries are generated under permissive and combinatorial conditions; many of the underlying transformations could likely be adapted to the COMBINAUT workflow if not already covered by the conditions presented here.

Beyond the discovery of several new classes of allosteric ligands for CCR2, shown in this study, our synergized VS-automated synthesis platform has the potential to be translated to the discovery of novel ligands for almost any druggable protein. The likelihood of identifying validated binders is greatest when high-quality structural information is available, particularly from ligand-bound X-ray crystal or cryo-EM structures. However, advances in protein structure prediction tools, such as AlphaFold, together with a range of pocket prediction methods, suggest that our platform could also be applied to identify binders for a diversity of proteins lacking experimentally determined structures.

Expanding our set of building blocks and adapting the platform setup to control additional reaction parameters will enrich our range of available transformations, thereby exponentially increasing the chemical space that can be accessed through automation. Further advancements could include the development of a closed-loop discovery platform that automates both synthesis and screening, realizing the vision of a self-driving medicinal chemistry laboratory.

## Supplementary Material



## Data Availability

The virtual screening workflow for this study is available through GitHub (https://github.com/CDDLeiden/combinaut/).

## Notes

Supplementary figures; abbreviations; experimental section; synthetic procedures and compound characterization; computational methods; biological methods; building block libraries; analytical data (HPLC–MS and NMR); and biological activity data (PDF)
