# Supplementary material for: Automated Parallel Synthesis Accelerates Virtual Screening Hit Discovery
Source: J Am Chem Soc. 2026 Jul 10;148(28):29908–20. doi: 10.1021/jacs.6c05055 (PMC13397544; doi:10.1021/jacs.6c05055)
Supplement: Supplementary file 1 [file ja6c05055_si_001.pdf]

Supporting Information

Automated Parallel Synthesis Accelerates Virtual Screening Hit Discovery

Sean M. McKenna[1,2], Martin Šícho[1,3], Cas van der Horst[1,2], Jesse Maasland[1], Edith van der Nol[1,2], Gianluca Turco[1,2], Andrius Bernatavicius[1], Gerard J.P. van Westen[1]*, Laura H. Heitman[1,2]*, Sebastian J. Pomplun[1,2]*

[1] Leiden Academic Centre for Drug Research, Leiden University, Leiden, 2333 CC, The Netherlands

[2] Oncode Institute; Utrecht, 3521 AL, The Netherlands

[3] CZ-OPENSCREEN: National Infrastructure for Chemical Biology, Department of Informatics and Chemistry, Faculty of Chemical Technology, University of Chemistry and Technology Prague, Technická 5, 166 28, Prague, Czech Republic

*Corresponding author; Email: Gerard J.P. van Westen: gerard@lacdr.leidenuniv.nl, Laura H. Heitman: l.h.heitman@lacdr.leidenuniv.nl, Sebastian J. Pomplun: s.j.pomplun@lacdr.leidenuniv.nl

Table of Contents

1. Supplementary figures 4
2. Abbreviations 19
3. Experimental 20
4. Chemistry Materials & Methods 20
    a. Materials 20
    b. Stock solutions 20
    c. General Synthesis Procedures (Manual/Automated) 20
    d. Semi-automated purification 23
    e. Characterisation 23
    f. Synthesis of Building Block **132** 24
    g. Resynthesis of >98.0% purity compounds **36** and **44** 26
5. Computational Methods 28
    a. Building block selection 28
    b. Library enumeration 28
    c. Molecular docking 29
    d. Interaction fingerprint analysis and scoring 30
    e. Tanimoto similarity scoring 30
6. Biological Materials & Methods 31
    a. Materials 31
    b. Cell culture 31
    c. Membrane preparation 31
    d. [^{3}H]CCR2-RA-[*R*] binding assays 31
    e. Tango β-Arrestin Recruitment Assay 32
    f. Data analysis 32
7. Building Block Libraries 33
    a. Amino acids 33
    b. Aldehydes 34
    c. Carboxylic acids 35
    d. Primary amines 37
    e. Sulfonyl chlorides 39
8. Analytical Data 40
    a. Substrate scope screen by HPLC-MS 40
    b. HPLC-MS Data for compounds **1-132** 48
    c. NMR Data for compound **4-16**, **36**, **44** and **130-132** 93
9. Biological Data 105
    a. Binding Affinity Determination: Compounds **17-58** 105

b. Ki Determination: High-purity Compounds **36** & **44** 110
c. β-Arrestin Recruitment IC_{50} Determination for **36** and **44** 111
10. References 112

Figure S1

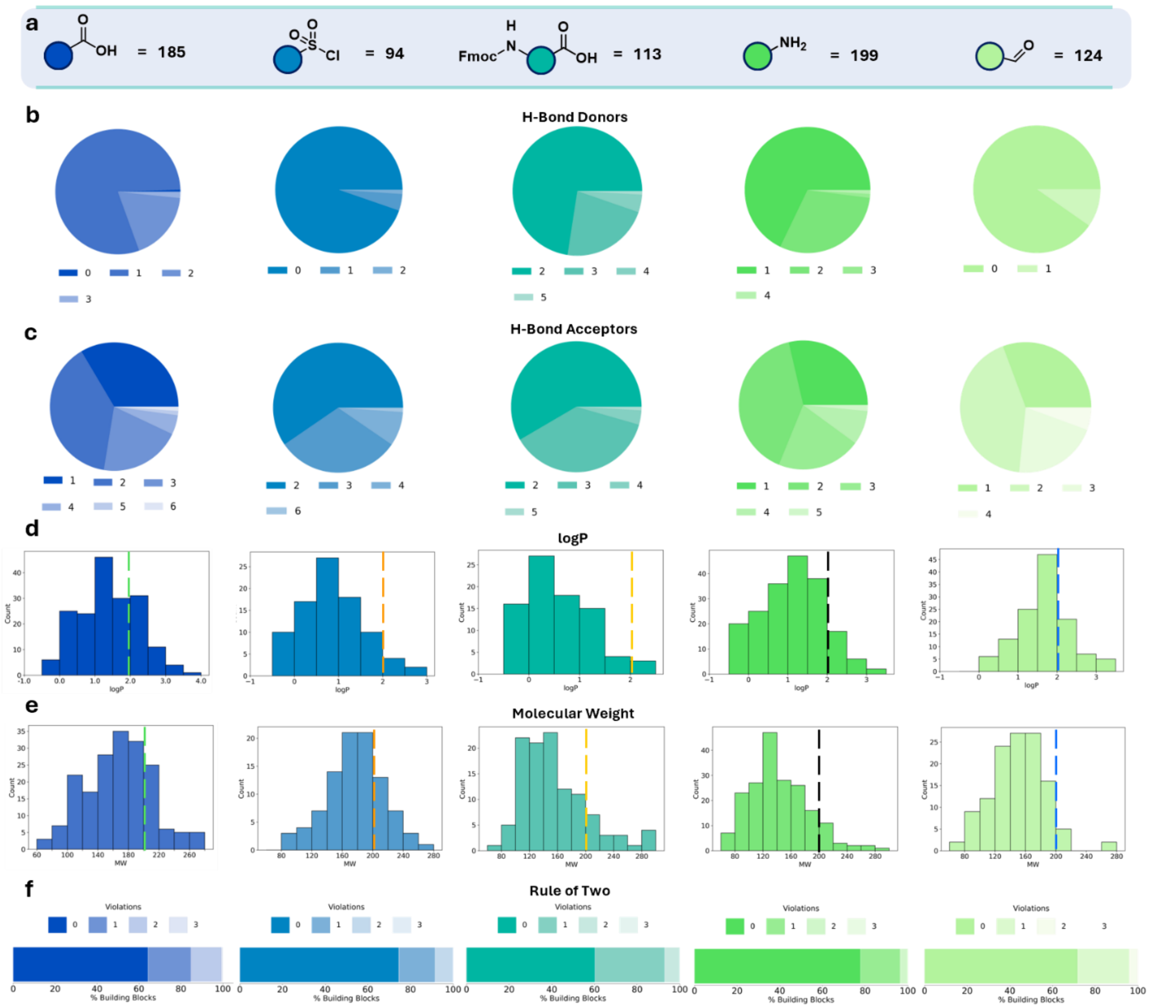

Figure S1. Building block curation and analysis for the construction of drug-like libraries. a) Building block classes and respective frequency within the curated building block set, b) H-bond donors, c) H-bond acceptors, d) logP, e) Molecular weight were calculated for all building blocks and plotted. Vertical dashed lines represent 'rule of two' boundaries. f) Cumulative 'rule of two' violations for building blocks are plotted below.

Figure S2

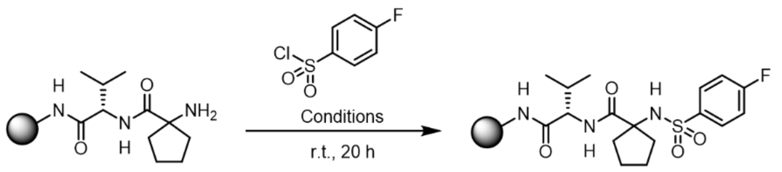

128

Conditions	Solvent	Base	Conversion (%)
A	$Et_2O + H_2O$	NaOH	86
B	Toluene + H_2O	Na_2CO_3	99
C	DCM	Et_3N	79
D	DCM	Pyridine	4
E	THF	DIPEA	89
F	DMF	Pyridine	32

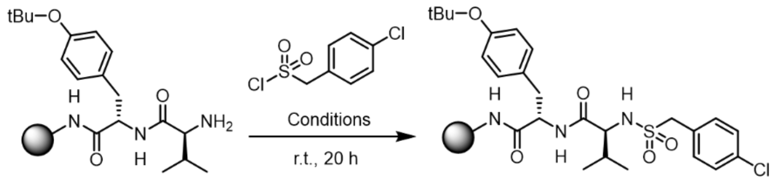

1

Conditions	Solvent	Base	Conversion (%)
A	Toluene + H_2O	Na_2CO_3	37
B	THF	DIPEA	64
C	DCM	Et_3N	61

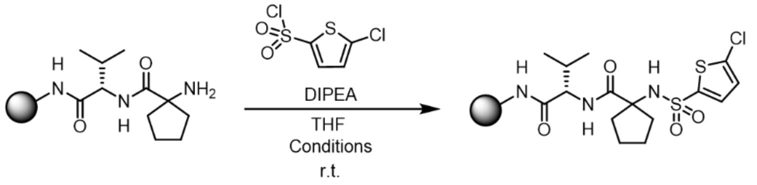

128

Conditions	time (h)	Conversion (%)
A	1	97
B	2	99
C	4	98
D	8	>99
E	24	>99

*Figure S2. Conditions screen for coupling of an amine with a sulfonyl chloride. Resin-bound dipeptides **128** and **1** (30 µmoles) were treated with sulfonyl chloride (3 equiv.) and base (5 equiv.) and agitated at ambient temperature. % conversion was determined by HPLC-MS analysis of crude reaction mixture following cleavage.*

Figure S3

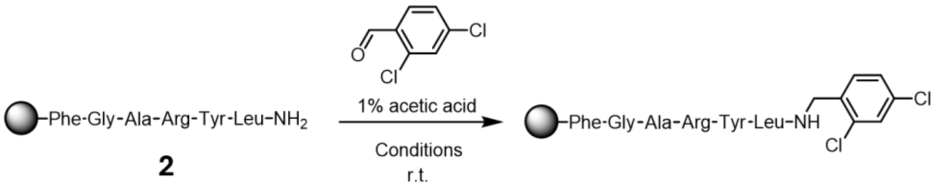

2

Conditions	Solvent	Reducing Agent	Conversion (%)
A	MeOH	$NaBH_3CN$	95
B	EtOH	$NaBH_3CN$	95
C	THF	$NaBH_3CN$	94
D	Toluene	$NaBH_3CN$	94
E	IPA	$NaBH_3CN$	40
F	MeCN	$NaBH_3CN$	92
G	MeOH	$NaBH(OAc)_3$	12
H	EtOH	$NaBH(OAc)_3$	17
I	THF	$NaBH(OAc)_3$	18
J	Toluene	$NaBH(OAc)_3$	14
K	IPA	$NaBH(OAc)_3$	14
L	MeCN	$NaBH(OAc)_3$	26

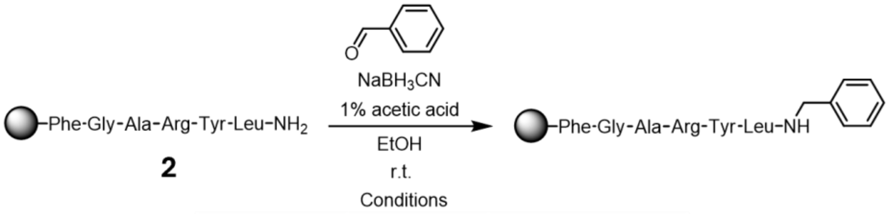

2

Conditions	time (h)	Conversion (%)
A	1	86
B	2	96
C	4	96
D	6	93

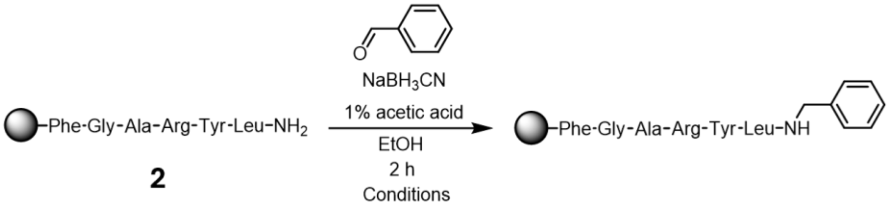

2

Conditions	Temperature (°C)	Conversion (%)
A	20	86
B	40	86

*Figure S3. Conditions screen for combining primary amine and aldehyde building blocks. Resin-bound peptide **2** (30 µmoles) was agitated with aldehyde (3 equiv.) and reducing agent (3 equiv.). % conversion was determined by HPLC-MS analysis of pelleted precipitate following cleavage, treatment with cold ether and centrifugation.*

Figure S4

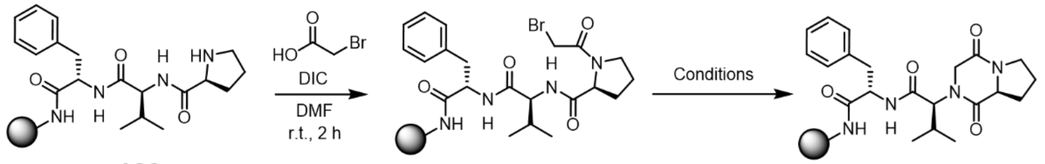

129

Conditions	Solvent	Base	Temperature (°C)	Conversion (%)
A	DMF	K_2CO_3	80	3
B	Toluene	Na_2CO_3	80	<0.1
C	THF	NaH	20	12
D	DCM	Pyridine	20	<0.1
E	DMF	K_2CO_3	80	4

*Figure S4. Conditions screen for 1,6-cyclisation. Resin-bound trimer peptide **129** (30 µmoles) was coupled to bromoacetic acid, then treated with base and agitated for 1 h. % conversion was determined by HPLC-MS analysis of crude reaction mixture following cleavage.*

Figure S5

Scaffold A:

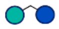

1) Resin swelling
2) Fmoc-deprotection
3) Amide coupling [A]
4) Fmoc-deprotection
5) Amide coupling [B]

Scaffold B:

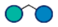

1) Resin swelling
2) Fmoc-deprotection
3) Amide coupling [A]
4) Fmoc-deprotection
5) Solvent Exchange (THF)
6) Sulfonylation [A]

Scaffold C:

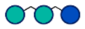

1) Resin swelling
2) Fmoc-deprotection
3) Amide coupling [A]
4) Fmoc-deprotection
5) Amide coupling [A]
6) Fmoc-deprotection
7) Amide coupling [B]

Scaffold D:

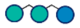

1) Resin swelling
2) Fmoc-deprotection
3) Amide coupling [A]
4) Fmoc-deprotection
5) Amide coupling [A]
6) Fmoc-deprotection
7) Solvent Exchange (THF)
8) Sulfonylation [A]

Scaffold E:

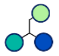

1) Resin swelling
2) Fmoc-deprotection
3) Amide coupling [A]
4) Fmoc-deprotection
5) Solvent Exchange (EtOH)
6) Reductive Amination
7) Amide coupling [B]

Scaffold F:

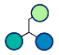

1) Resin swelling
2) Fmoc-deprotection
3) Amide coupling [A]
4) Fmoc-deprotection
5) Solvent Exchange (EtOH)
6) Reductive Amination
7) Sulfonylation [A]

Scaffold G:

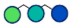

1) Resin swelling
2) Fmoc-deprotection
3) Amide coupling [C]
4) Nucleophilic substitution
5) Amide coupling [A]
6) Fmoc-deprotection
7) Amide coupling [B]

Scaffold H:

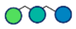

1) Resin swelling
2) Fmoc-deprotection
3) Amide coupling [C]
4) Nucleophilic substitution
5) Amide coupling [A]
6) Fmoc-deprotection
7) Solvent Exchange (THF)
8) Sulfonylation [A]

Scaffold I:

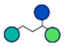

1) Resin swelling
2) Fmoc-deprotection
3) Amide coupling [A]
4) Fmoc-deprotection
5) Amide coupling [C]
6) Nucleophilic substitution
7) Amide coupling [B]

Scaffold J:

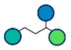

1) Resin swelling
2) Fmoc-deprotection
3) Amide coupling [A]
4) Fmoc-deprotection
5) Amide coupling [C]
6) Nucleophilic substitution
7) Solvent Exchange (THF)
8) Sulfonylation [A]

Scaffold K:

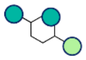

1) Resin swelling
2) Fmoc-deprotection
3) Amide coupling [A]
4) Fmoc-deprotection
5) Amide coupling [A]
6) Fmoc-deprotection
7) Solvent Exchange (EtOH)
8) Reductive amination
9) Amide coupling [C]
10) Nucleophilic substitution
11) Solvent Exchange (THF)
12) Base cyclisation

Scaffold L:

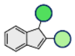

1) Resin swelling
2) Fmoc-deprotection
3) Amide coupling [B]
4) Solvent Exchange (DMSO)
5) S_NAr [A]
6) Nitro Reduction
7) Acid catalysed hetereocyclisation

Scaffold M:

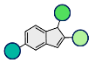

1) Resin swelling
2) Fmoc-deprotection
3) Amide coupling [A]
4) Fmoc-deprotection
5) Amide coupling [B]
6) Solvent Exchange (DMSO)
7) S_NAr [A]
8) Nitro Reduction
9) Acid catalysed hetereocyclisation

Scaffold N:

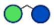

1) Amide coupling [D]

Scaffold O:

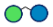

1) Sulfonylation [B]

*Figure S5. Sequence of automated transformations, deprotections and solvent exchanges established for accessing scaffolds **A**-**M** using automated solid-phase synthesis, and scaffolds **N**-**O** using automated solution-phase synthesis. See methods section for respective conditions.*

Figure S6

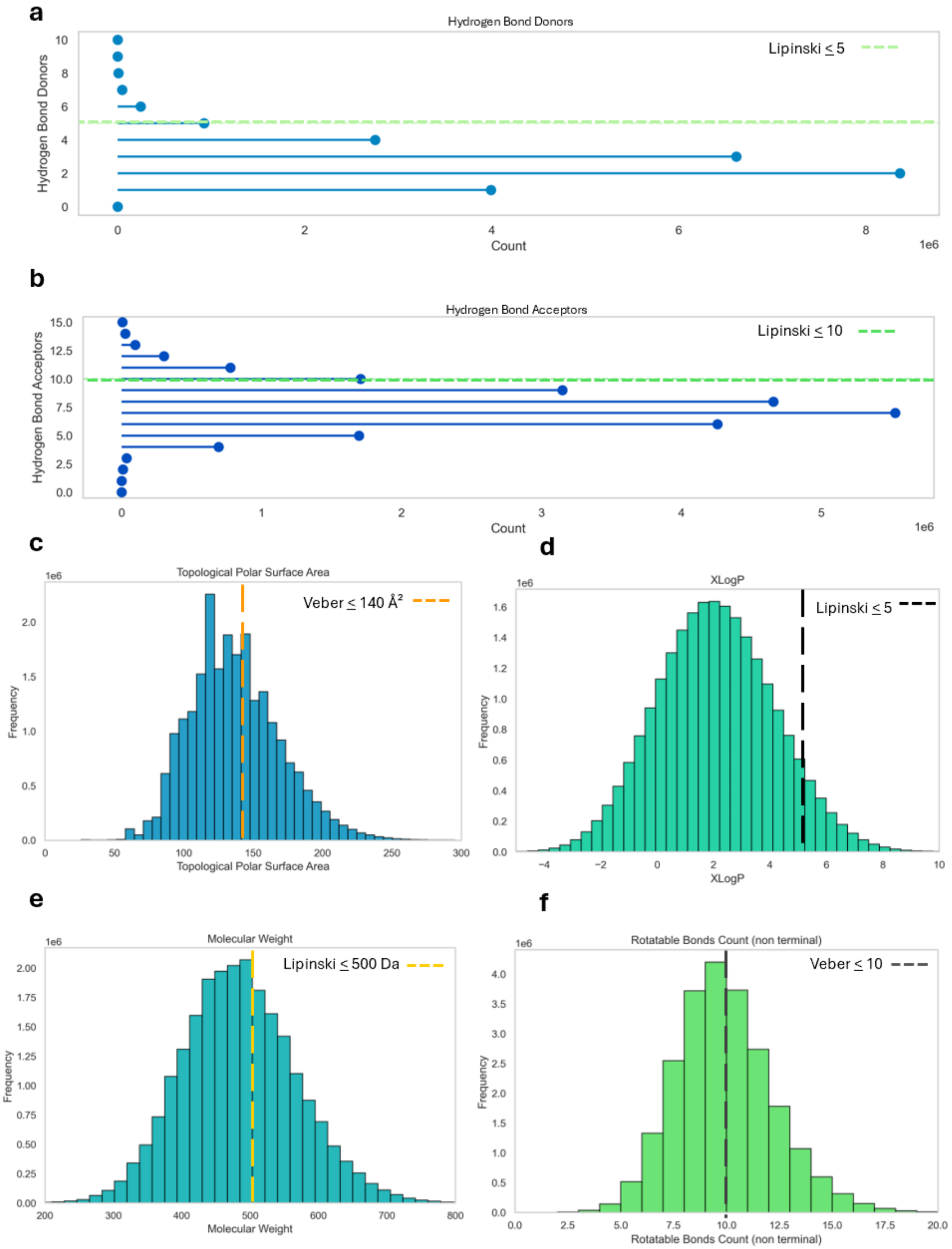

Figure S6. Analysis of fully enumerated 22.9 million member library for drug-like properties. a) H-bond donors, b) H-bond acceptors, c) topological polar surface area, d) logP, e) molecular weight, f) number of non-terminal rotatable bonds were calculated for all library members. Dashed lines on plotted lollipop and histogram plots represent upper limits as rules for orally bioavailable drugs as defined by Lipinski and Veber.

Figure S7:

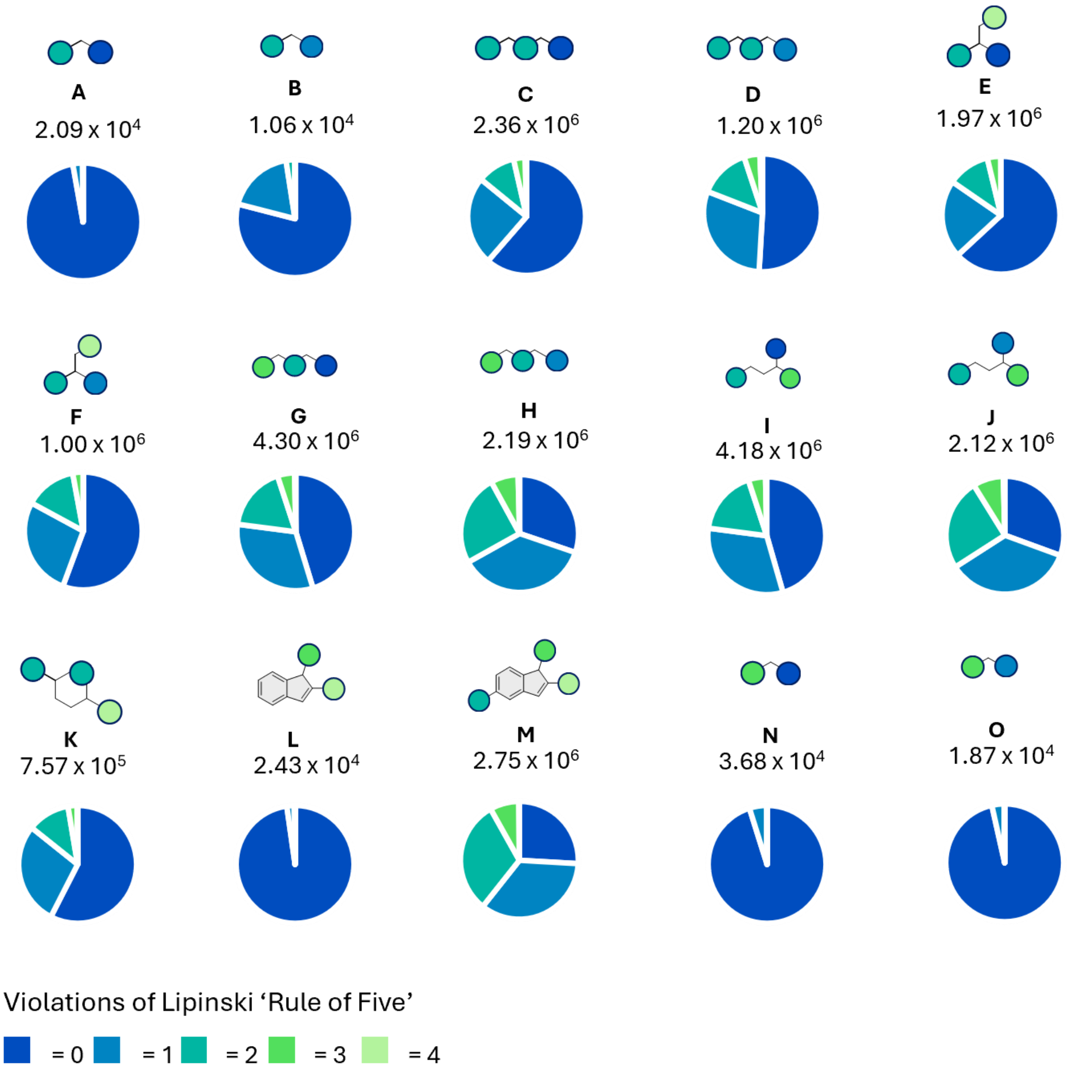

Figure S7. Analysis of fully enumerated library scaffolds for drug-like properties. H-bond donors, H-bond acceptors, logP, molecular weight are calculated, and cumulative Lipinski 'rule of five' violations per library member are plotted as pie charts.

Figure S8:

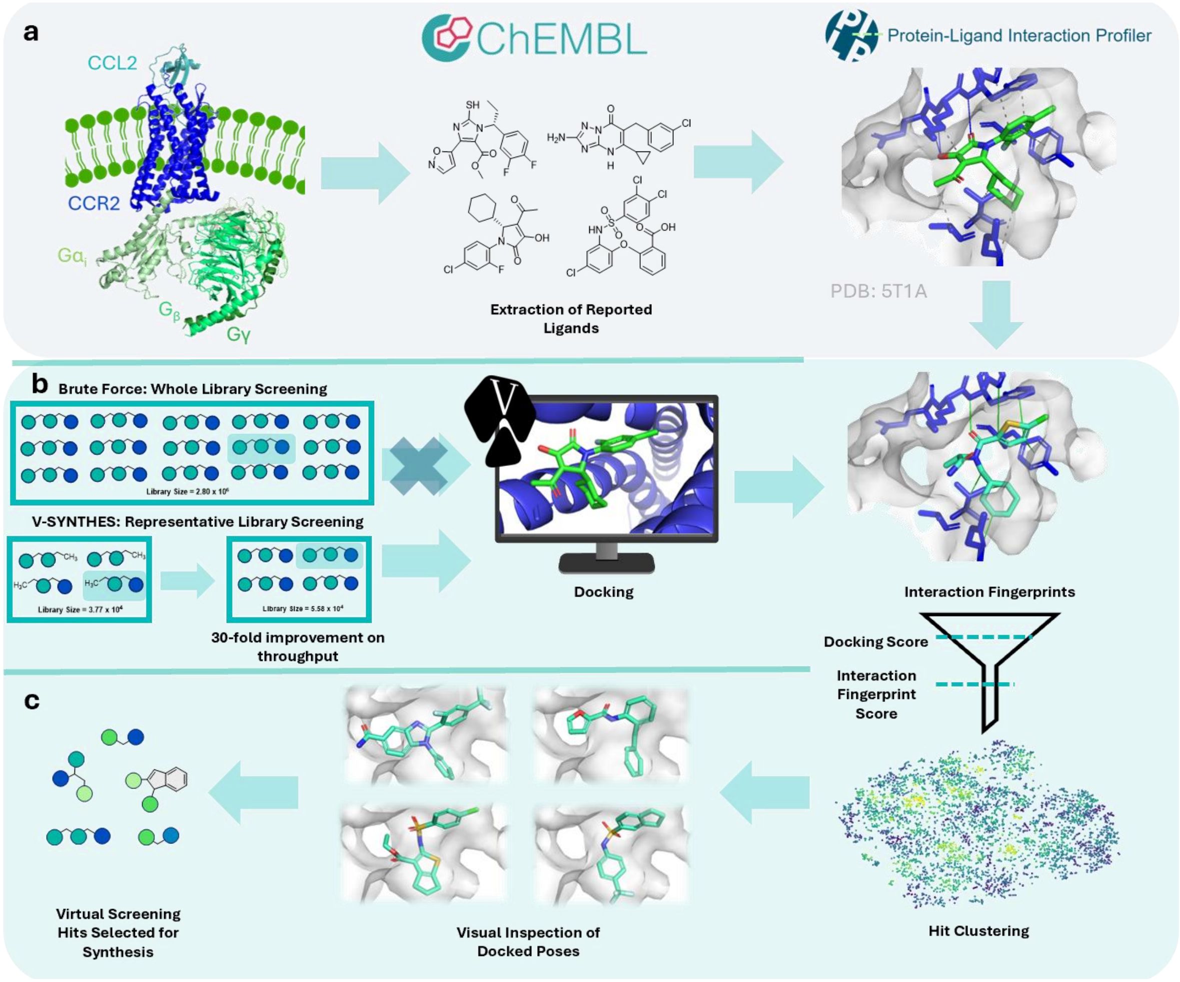

Figure S8. Summary of virtual screening workflow. a) To identify ligands for CCR2, a docking model was prepared from a ligand bound structure of the target protein (PDB:5T1A). Reported allosteric CCR2 ligands were extracted from ChEMBL and docked using AutoDock VINA. Thereafter, bound poses were analysed using PLIP and used to establish a consensus pharmacophore, from which a scoring function was generated to evaluate binding poses for virtual library members. b) Representative V-SYNTHES libraries were docked using AutoDock VINA and interaction fingerprints (IFP) were determined using PLIP. Library members were filtered based on minimum affinity score (≤-8.0 kcal/mol) and ranked by IFP score. c) Hits were clustered in TSNE plots, from which high-scoring members were evaluated by visual inspection. Compounds with rational docked poses were selected for preparation by automated synthesis.

Figure S9:

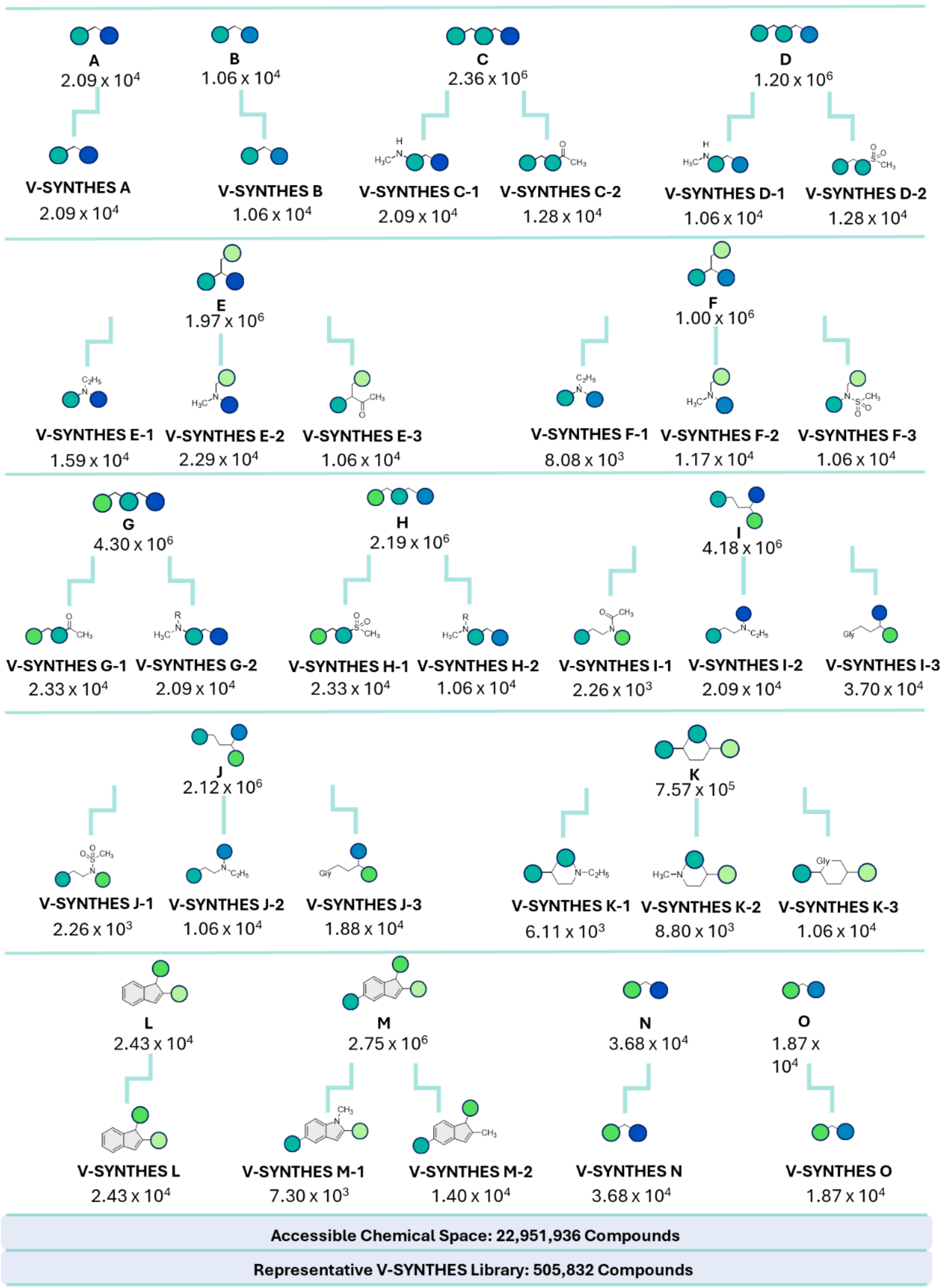

Figure S9. Summary of V-SYNTHES approach to sample multi-million member chemical space through generation of representative synthon libraries. In three building block scaffolds, a single building block is replaced by a placeholder group.

Figure S10:

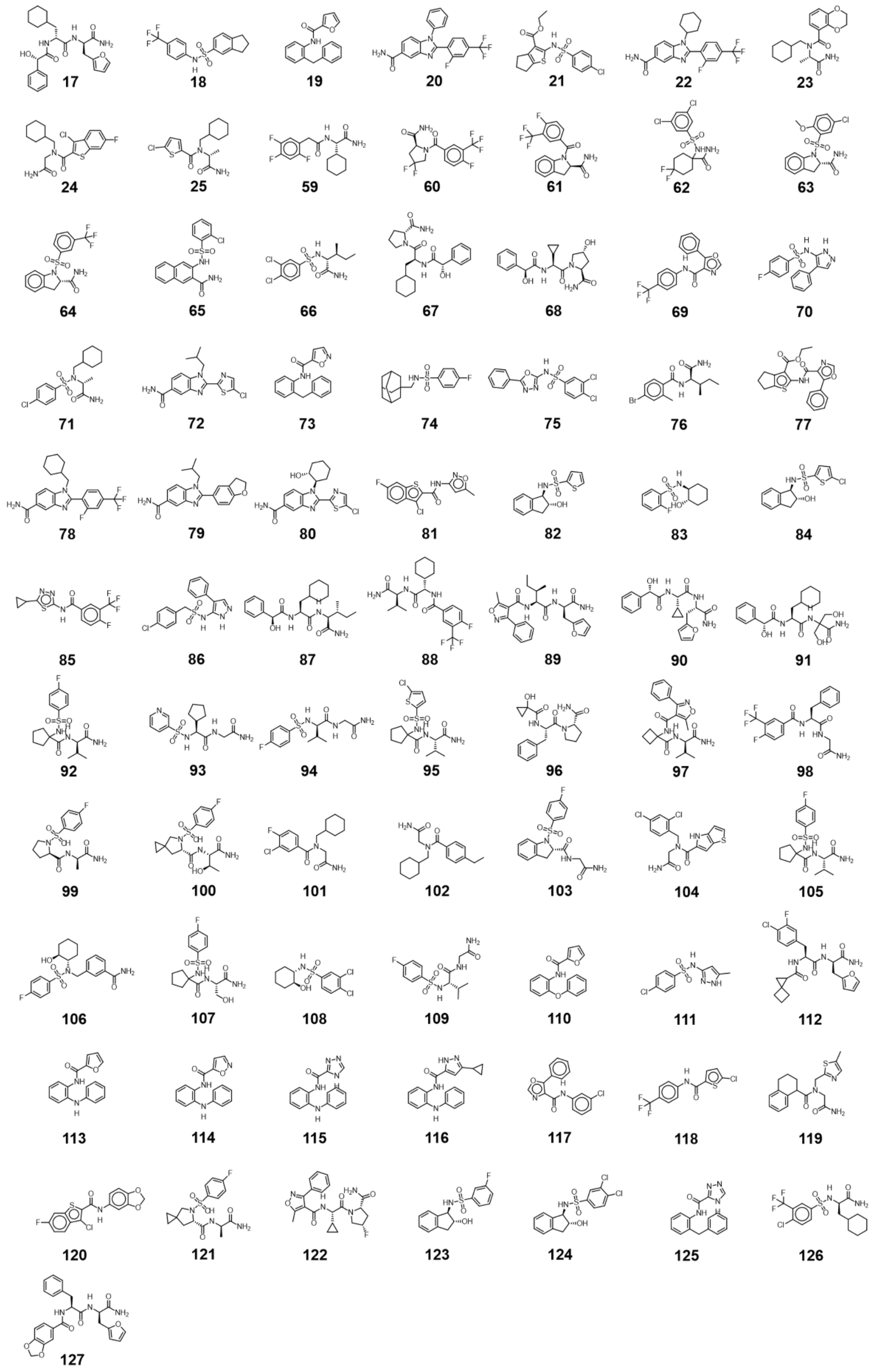

*Figure S10. Virtual screening hits **17**-**25** and **59**-**127** prepared using COMBINAUT.*

Figure S11:

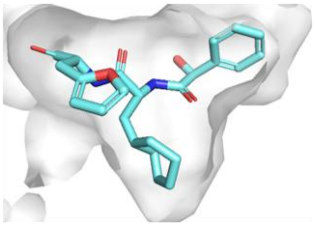

17
CCR2 K_i = 38.0 μM
Similarity Score: 0.15

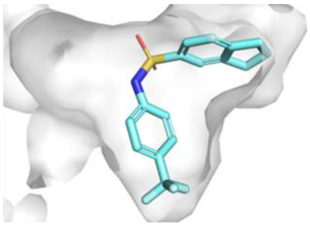

18
CCR2 K_i = 6.8 μM
Similarity Score: 0.30

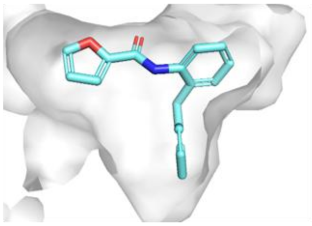

19
CCR2 K_i = 37.2 μM
Similarity Score: 0.20

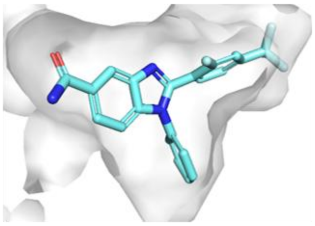

20
CCR2 K_i = 49.0 μM
Similarity Score: 0.23

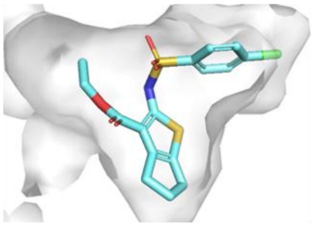

21
CCR2 K_i = 0.9 μM
Similarity Score: 0.30

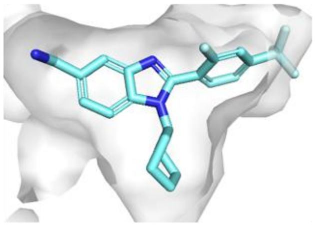

22
CCR2 K_i = 35.5 μM
Similarity Score: 0.23

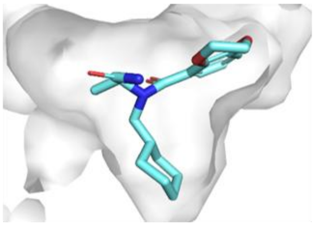

23
CCR2 K_i = 53.7 μM
Similarity Score: 0.18

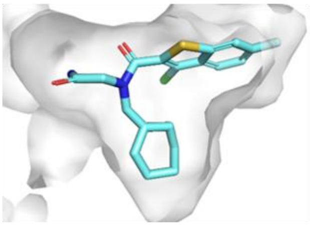

24
CCR2 K_i = 29.5 μM
Similarity Score: 0.22

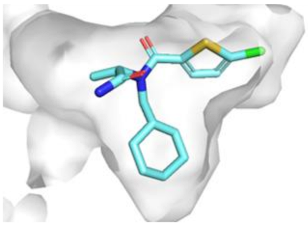

25
CCR2 K_i = 6.0 μM
Similarity Score: 0.22

*Figure S11. Tanimoto similarity scores based on Morgan fingerprints calculated for virtual screening hits **17**-**25** against previously reported CCR2 allosteric ligands. Reported here is the maximum Tanimoto similarity score each validated hit was assigned from the range of previously reported CCR2 allosteric ligands sampled.*

Figure S12:

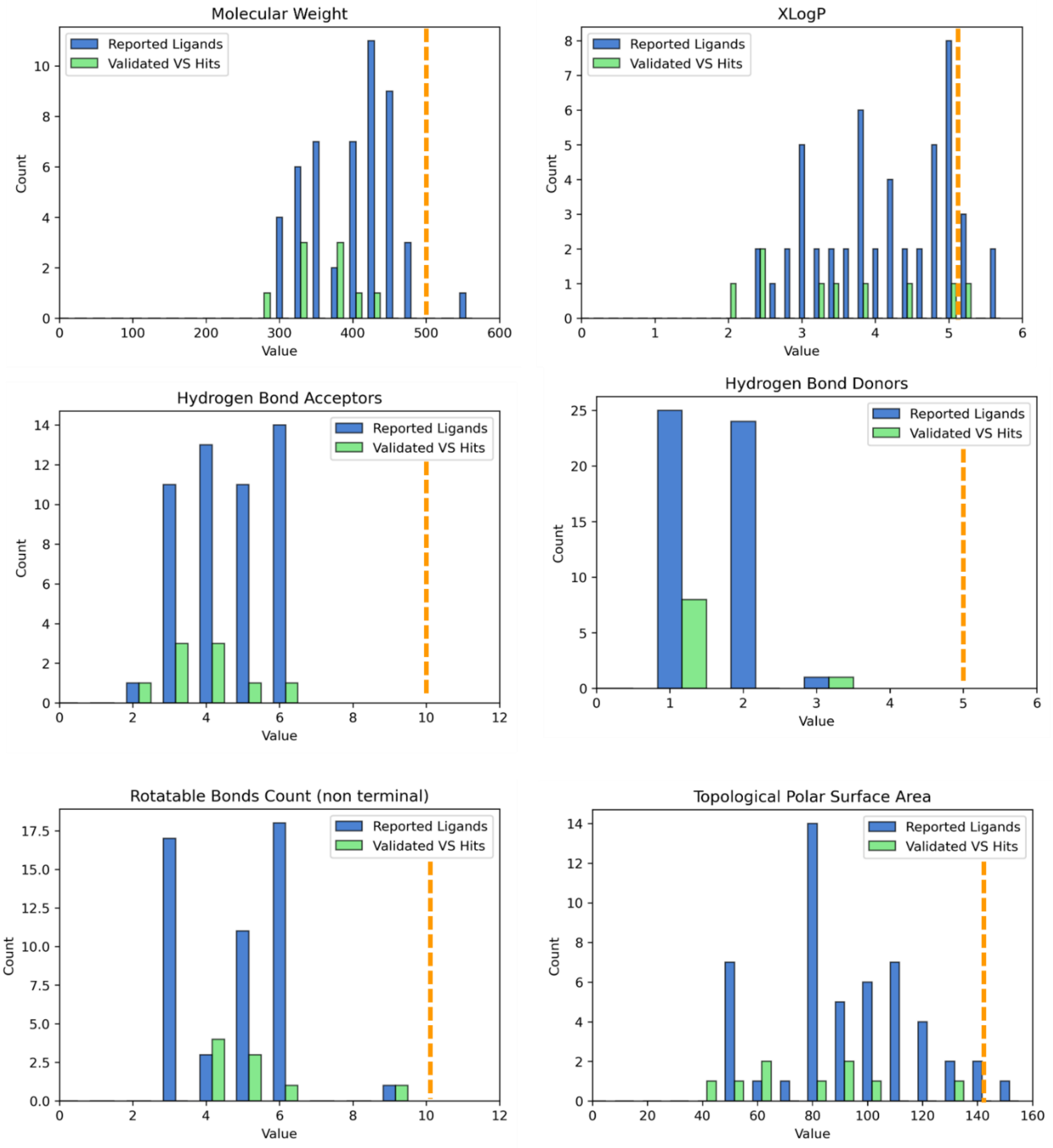

*Figure S12: Compound property profile of virtual screening hits **17**-**25** against previously reported CCR2 allosteric ligands. Lipinski and Veber rules for drug-like chemical space are indicated as upper limits with a dashed orange line.*

Figure S13

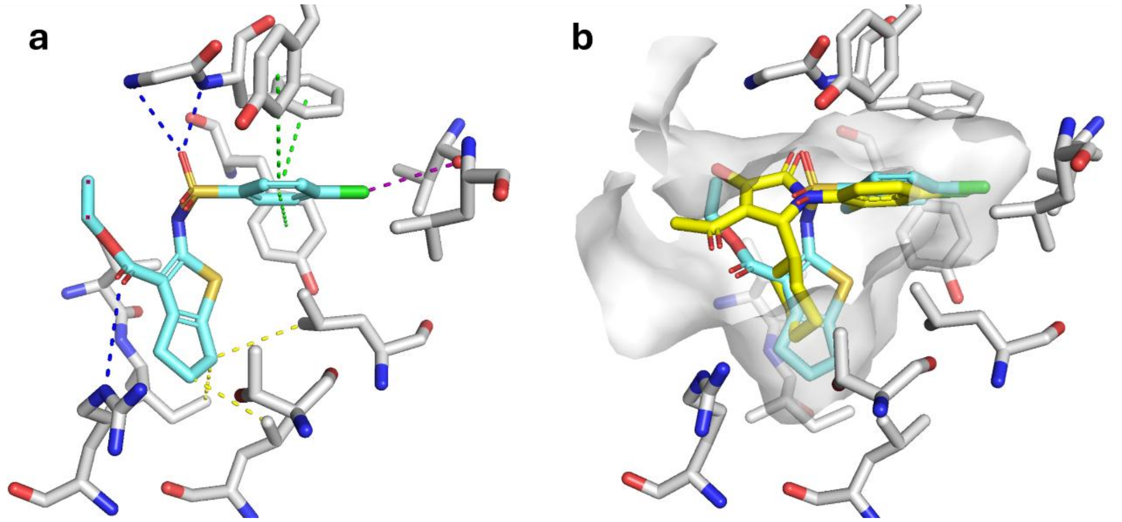

*Figure S13: a) Docked pose of validated VS hit **21** with π-bonding interactions (green), halogen bonding (purple), H-bonding (blue) and hydrophobic interactions (yellow) indicated. b) Overlay of **21** (turquoise) and CCR2-RA-[R] (yellow) highlights common key interactions, and underscores how the cyclopenta(b)thiophene ring system effectively exploits the lipophilic pocket formed by Leu81, Leu134, and Ile245.*

Figure S14

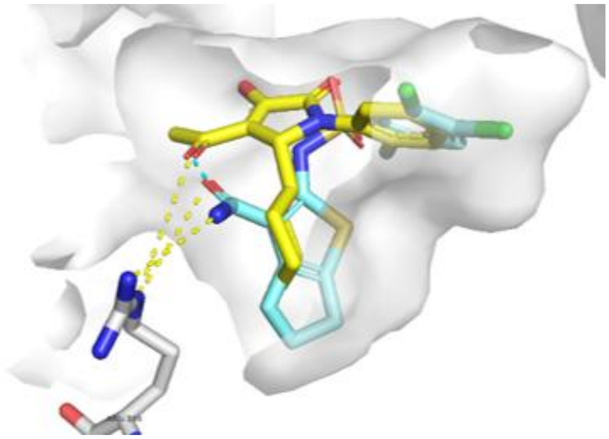

*Figure S14: Overlay of docked pose of optimized virtual screening hit **36** (turquoise) with ligand bound crystal structure of CCR2-RA-[R] (yellow) (PDB: 5T1A). The carbonyl of the terminal amide and acetyl groups are located 1.1Å apart, highlighting the possibility that **36** may participate in a similar H-bonding interaction with proximal residue Arg138.*

Figure S15

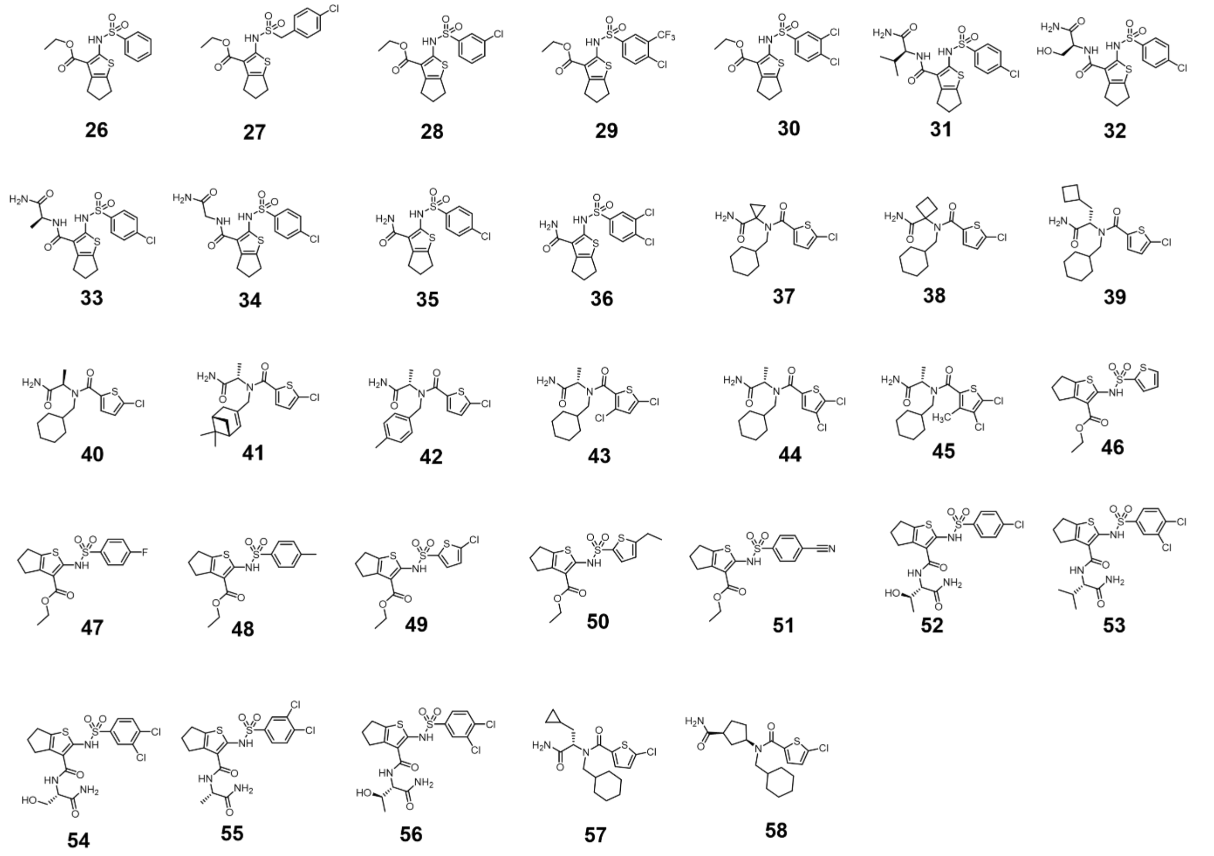

*Figure S15. Compounds **26**-**58** were prepared using COMBINAUT towards the optimization of validated virtual screening hits **21** and **25**,*

Figure S16

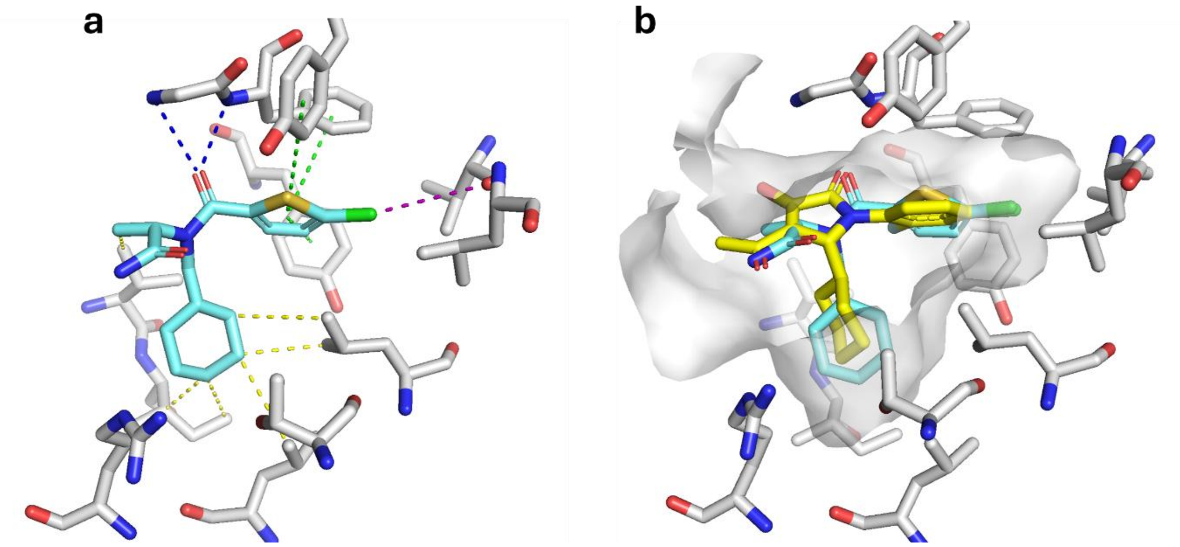

*Figure S16: a) Docked pose of validated VS hit **25** with π-bonding interactions (green), halogen bonding (purple), H-bonding (blue) and hydrophobic interactions (yellow) indicated. b) Overlay of **25** (turquoise) and CCR2-RA-[R] (yellow) highlights common key interactions and shape similarity.*

Abbreviations:

BB	Building block
CCL2	C-C motif chemokine ligand 2
CCR2	C-C chemokine receptor type 2
COMBINAUT	Combinatorial automated synthesis
DCM	Dichloromethane
DIC	N,N'-Diisopropylcarbodiimide
DIPEA	N, N-Diisopropylethylamine
DMTA	Design-make-test-analyse
GPCR	G-protein coupled receptor
HATU	Hexafluorophosphate azabenzotriazole tetramethyl uronium
HBA	Hydrogen bond acceptor
HBD	Hydrogen bond donor
HPLC-MS	High-performance liquid chromatography- mass spectrometry
IFP	Interaction fingerprint
LRRK2	Leucine-rich repeat kinase 2
MeCN	Acetonitrile
MW	Molecular weight
NaH	Sodium hydride
NMR	Nuclear magenetic resonance spectroscopy
PLIP	Protein-ligand interaction profiler
r.t.	Room temperature
RBC	(Non-terminal) rotatable bond count
S_N2	Substitution nucleophilic bimolecular
S_NAr	Nucleophilic aromatic substitution
SPS	Solid-phase synthesis
TFA	Trifluoroacetic acid
THF	Tetrahydrofuran
TPSA	Topological polar surface area
U2OS	Uppsala 2 osteosarcoma cell line
UMAP	Uniform manifold approximation and projection
VS	Virtual screening

Experimental

Chemistry Methods:

Materials:

Reagents and solvents were purchased from Merck, Fisher Scientific, Fluorochem or VWR and were used without further purification. Building blocks grouped as Fmoc-protected amino acids, carboxylic acids, aldehydes, primary amines and sulfonyl chlorides were collated from synthetic chemistry labs within our institution, and were originally purchased from BLDpharm, Tokyo Chemical Industry (TCI), ABCR, and Chemspace. Rink Amide ProTide™ Resin (CEMR002-C) was purchased from CEM.

Stock Solutions:

Piperidine solution: 20% piperidine, 2% formic acid, 78% DMF

Acetic acid solution: 10% acetic acid in EtOH

Cleavage cocktail: 49% TFA, 49% DCM, 2% triisopropylsilane

General Synthesis Procedures

Solid-phase synthesis protocols were developed manually before being translated to automation using a Biotage Syro I parallel peptide synthesizer. Reaction screening and substrate scope experiments were performed using 30 µmoles Rink amide ProTide™ Resin. Synthesis of target products was performed using 60 µmoles Rink amide ProTide™ Resin.

Resin Swelling:

In a fritted syringe, ProTide™ resin (60 µmoles) was treated with DMF (2 mL) and incubated at ambient temperature for 5 min. Syringe was thereafter drained under reduced pressure.

Solvent Exchange (THF, EtOH, DMSO).

The resin was washed with the new reaction solvent (2 mL), agitated for 1 min and drained (3 x cycles).

FMOC-deprotection:

To resin was added piperidine solution (1.5 mL) and agitated at 70 °C for 4 mins. The resin was drained and thereafter subject to washes with DMF (3 x 1.2 mL).

Amide coupling [A]:

To resin was added a 0.4M solution of Fmoc-protected amino acid in DMF (0.75 mL, 0.3 mmoles, 5.0 equiv.), followed by treatment with 0.36M HATU in DMF (0.75 mL, 0.27 mmol,

4.5 equiv.) and DIPEA (85 µL, 0.48 mmol, 8.0 equiv.). The reaction was agitated at 70 °C for 40 mins, and thereafter resin was subject to washes with DMF (3 x 1.2 mL).

Amide coupling [B]:

To resin was added a 0.4M solution of carboxylic acid in DMF (0.45 mL, 0.18 mmoles, 3.0 equiv.), followed by treatment with 0.36M HATU in DMF (0.45 mL, 0.16 mmol, 2.7 equiv.) and DIPEA (85 µL, 0.48 mmol, 8.0 equiv.). The reaction was agitated at 70 °C for 40 mins, and thereafter resin was subject to washes with DMF (3 x 1.2 mL).

Amide coupling [C]:

To resin was added a 1M solution of bromoacetic acid in DMF (0.6 mL, 0.6 mmoles, 10.0 equiv.) and 1M DIC in DMF (0.6 mL, 0.6 mmoles, 10.0 equiv.). The reaction was agitated at ambient temperature for 2 h, and thereafter resin was subject to washes with DMF (3 x 1.2 mL).

Amide coupling [D]:

Solution phase amide coupling was performed in a 2mL eppendorf tube. A 0.4M solution of carboxylic acid in DMF (0.15 mL, 60 µmoles, 1.5 equiv.) was combined with 0.36M HATU in DMF (0.15 mL, 54 µmoles 1.35 equiv.) and treated with DIPEA (21 µL, 0.12 mmoles, 3 equiv.). To this mixture was added a 0.4 M solution of primary amine in DMF (0.1 mL, 40 µmoles, 1.0 equiv.). The reaction was agitated at 70 °C for 40 mins. Crude reaction mixture was transferred to semi-automated purification without additional manipulation.

Sulfonylation [A]:

To resin was added a 0.2M solution of sulfonyl chloride in THF (0.9 mL, 0.18 mmoles, 3.0 equiv.), followed by treatment with DIPEA (85 µL, 0.48 mmol, 8.0 equiv.). The reaction was agitated at ambient temperature for 2 h and the syringe was thereafter drained under reduced pressure. Resin was washed with DMF (3 x 1.2 mL).

Sulfonylation [B]:

Solution phase sulfonylation was performed in a 2mL eppendorf tube. A 0.2M solution of sulfonyl chloride in THF (0.3 mL, 60 µmoles, 1.5 equiv.) was combined with a 0.4M solution of primary amine in THF (0.1 mL, 40 µmoles, 1.0 equiv.). The mixture was treated with DIPEA (21 µL, 0.12 mmoles, 3 equiv.) and agitated at ambient temperature for 2 h. Crude reaction mixture was evaporated to dryness under a flow of compressed air.

Reductive amination:

To resin was added a 0.2M solution of aldehyde in EtOH (0.9 mL, 0.6 mmoles, 3.0 equiv.), followed by acetic acid solution (0.2 mL) and 0.2M $NaBH_3CN$ in EtOH (0.9 mL, 0.18 mmol, 3.0 equiv.). The reaction was agitated at ambient temperature for 2 h and the syringe was thereafter drained under reduced pressure. Resin was washed with EtOH (3 x 2 mL) and subsequently with DMF (3 x 1.2 mL).

S_NAr [A] (primary amine):

To resin was added a 0.4M solution of primary amine in DMSO (1.5 mL, 0.6 mmoles, 10.0 equiv.) and DIPEA (105 µL, 0.6 mmol, 10.0 equiv.). The reaction was agitated at 80 °C for 8 h, and thereafter resin was subject to washes with DMF (3 x 1.2 mL).

S_NAr [B] (aryl halide):

To resin was added a 0.4M solution of aryl halide in DMSO (1.5 mL, 0.6 mmoles, 10.0 equiv.) and DIPEA (105 µL, 0.6 mmol, 10.0 equiv.). The reaction was agitated at 80 °C for 8 h, and thereafter resin was subject to washes with DMF (3 x 1.2 mL).

Nitro reduction:

To resin was added a 1M solution of tin (II) chloride dihydrate in DMF (2.4 mL, 2.4 mmol, 40 equiv.). The reaction was agitated at ambient temperature for 16 h, and thereafter resin was subjected to washes with 50% MilliQ H_2O in DMF (5 x 1.5 mL) and with DMF (5 x 1.2 mL).

Acid-catalysed hetereocyclisation:

To resin was added a 0.4M solution of aldehyde in DMF (0.75 mL, 0.3 mmoles, 5.0 equiv.) and 0.4 M solution of p-toluene sulfonic acid in DMF (0.75 mL, 0.3 mmoles, 5.0 equiv.). The reaction was agitated at ambient temperature for 5 h and the syringe was thereafter drained under reduced pressure. Resin was washed with DMF (3 x 1.2 mL).

Nucleophilic substitution:

To resin was added a 0.4M solution of primary amine in DMF (1.5 mL, 0.6 mmoles, 10.0 equiv.). The reaction was agitated at ambient temperature for 2 h, and thereafter resin was subject to washes with DMF (3 x 1.2 mL).

Base cyclisation:

To resin was added a 0.2M solution of NaH in THF (2.4 mL, 0.48 mmoles, 8.0 equiv.). The reaction was agitated at ambient temperature for 1 h, and thereafter resin was subject to washes with THF (3 x 2 mL) and DMF (3 x 1.2 mL).

Linker Cleavage:

Resin was washed with DCM (5 x 2 mL), then capped and treated with cleavage cocktail (1 mL). The reaction was agitated for 1 h at ambient temperature. Eluent was collected from syringe, washed with DCM (1 mL) and evaporated to dryness under a flow of compressed air. Crude residue was dissolved in DMF (0.2 mL) prior to purification.

Semi-automated purification:

C-18 Column chromatography

Crude products were purified by automated reverse phase chromatography using a Biotage Selekt System with a pre-packed column (Biotage® Sfär Bio 10 g, C18 - Duo 300 Å 20 µm). A standardized 5–100% MeCN/MilliQ H_2O (0.1% TFA), 18 min gradient was applied and UV absorbance was measured at 254 nm.

Sample Preparation:

Column fractions containing a UV-active product at 254 nm and a matching mass ion for the target product were concentrated *in vacuo* and lyophilized to dryness. DMSO stock solutions were prepared and stored at -20 °C.

Characterization:

HPLC-MS

Mass spectra were measured using a Shimadzu Prominence LC-MS-2020 system and a Gemini C18 Phenomenex column (50 × 3 mm, 3 µm) flow rate = 1.3 mL/min, using a gradient of 10–90% MeCN/H_2O (0.1% FA) and measuring UV absorbance at 254 nm

NMR:

^{1}H spectra were recorded on a Bruker AV-400 (400 MHz) spectrometer. ^{13}C NMR were recorded on a Bruker AV-400 (400 MHz) spectrometer or Bruker AV-500 (500 MHz) spectrometer. Chemical shift values are reported in parts per million (ppm) and designated by δ. Tetramethylsilane or solvent resonance was used as internal standard. Coupling constants (J) are reported in Hertz (Hz) and multiplicities are indicated by s (singlet), br s (broad singlet), d (doublet), t (triplet), td (triplet of doublets), or m (multiplet).

Synthesis of Building Block 132:

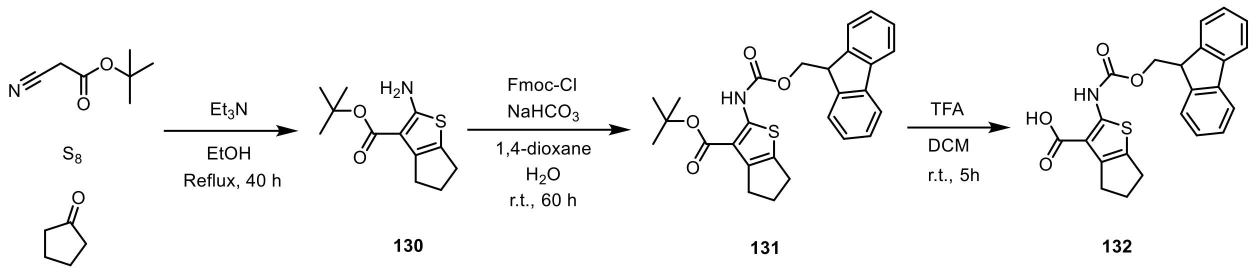

130 **131** **132**

Tert-butyl 2-amino-5,6-dihydro-4H-cyclopenta[b]thiophene-3-carboxylate (130)

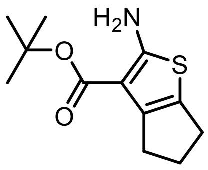

Sulfur (0.71 g, 22 mmoles, 1.1 equiv.) was suspended in EtOH (40 mL), to which was added cyclopentanone (1.77 mL, 20 mmoles, 1.0 equiv.) and tert-butyl 2-cyanoacetate (2.85 mL, 20 mmoles, 1.0 equiv.). Triethylamine (5.58 mL, 40 mmoles, 2.0 equiv.) was added and the reaction was heated to reflux for 40 h. The reaction was cooled to ambient and concentrated to an oil *in vacuo*. The crude was separated between ethyl acetate (150 mL) and water (100 mL). Organics were washed with brine (100 ml) and dried over $MgSO_4$. Organic extract was purified by normal phase chromatography (0-5% ethyl acetate in petroleum ether). Product fractions were evaporated to dryness to give tert-butyl 2-amino-5,6-dihydro-4H-cyclopenta[b]thiophene-3-carboxylate as a yellow oil (1.99 g, 8.31 mmol, 42%).

^{1}H NMR: (400 MHz, $CDCl_3$) δ 2.80 (m, 2H), 2.70 (m, 2H), 2.29 (m, 2H), 1.53 (s, 9H). ^{13}C NMR (101 MHz, $CDCl_3$) δ 165.33, 164.99, 142.47, 120.63, 103.98, 79.42, 30.60, 28.50, 28.15, 26.80. MS (ESI): m/z calculated for $C_{12}H_{17}NO_2S$ [M+H]= 239.038, found = 239,950

Tert-butyl 2-((((9H-fluoren-9-yl)methoxy)carbonyl)amino)-5,6-dihydro-4H-cyclopenta[b]thiophene-3-carboxylate (131)

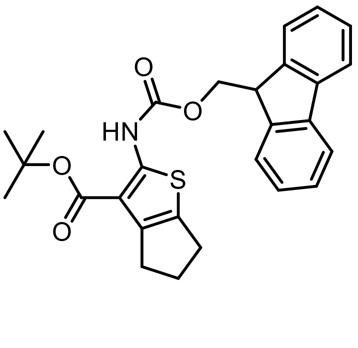

Intermediate 130 (1.55 g, 6.48 mmol, 1.0 equiv.) was dissolved up in 1,4-dioxane (20 mL), to which was added Fmoc-Cl (1.84 g, 7.12 mmol, 1.1 equiv.), sodium hydrogen carbonate (2.72 g, 32.4 mmol, 5.0 equiv.) and water (10 mL). The mixture was stirred at ambient temperature for 60 h. HPLC-MS indicated majority formation of target product after this time. The reaction was concentrated *in vacuo* and the crude residue was redissolved in ethyl acetate (80 mL) and water (80 mL). Organics were washed with brine (50 mL) and dried over anhydrous $MgSO_4$. The organic extract was partially concentrated in vacuo, resulting in the formation of a white precipitate. Solids were collected by sintered funnel filtration and dried *in vacuo*. Tert-butyl 2-((((9H-fluoren-9-yl)methoxy)carbonyl)amino)-5,6-dihydro-4H-cyclopenta[b]thiophene-3-carboxylate was collected as a white solid (1.87 g, 4.05 mmol, 63%).

^{1}H NMR: (400 MHz, $CDCl_3$) δ 10.41 (s, 1H), 7.78 (d, J = 7.4 Hz, 2H), 7.65 (d, J = 7.6 Hz, 2H), 7.42 (t, J = 7.6 Hz, 2H), 7.34 (td, J = 7.1, 1.3 Hz, 2H), 4.51 (d, J = 7.2 Hz, 2H), 4.31 (t, J = 7.2 Hz, 1H), 2.86 (m, 4H), 2.37 (m, 2H), 1.59 (s, 9H). ^{13}C NMR (101 MHz, $CDCl_3$) δ 165.14, 152.81, 152.23, 143.53, 141.93, 141.26, 130.42, 127.81, 127.16, 125.13, 120.02, 109.15, 81.27, 68.11, 46.93, 30.59, 28.86, 28.36, 27.68.MS (ESI): m/z calculated for $C_{27}H_{27}NO_4S$ [M+H]= 461.166, found = 462,050.

2-((((9H-fluoren-9-yl)methoxy)carbonyl)amino)-5,6-dihydro-4H-cyclopenta[b] thiophene-3-carboxylic acid (132)

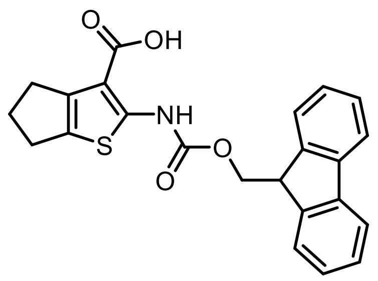

Intermediate 131 (1.75 g, 3.79 mmol, 1.0 equiv.) was dissolved in DCM (38 mL) and treated with TFA (1.45 mL, 18.96 mmol, 5.0 equiv.). The resultant mixture was stirred at ambient temperature for 16 h. Volatiles were removed *in vacuo* and the crude was purified by reverse phase column chromatography (5-100% MeCN in dH_2O). The product was lyophilized to dryness, giving 2-((((9H-fluoren-9-yl)methoxy)carbonyl)amino)-5,6-dihydro-4H-cyclopenta[b] thiophene-3-carboxylic acid as an off-white solid (174 mg, 0.43 mmol, 11%).

^{1}H NMR: (400 MHz, $CDCl_3$) δ 7.71 (d, J = 7.0 Hz, 2H), 7.56 (d, J = 7.4 Hz, 2H), 7.35 (t, J = 7.3 Hz, 2H), 7.26 (td, J = 7.1, 1.3 Hz, 2H), 4.44 (d, J = 7.4 Hz, 2H), 4.24 (t, J = 7.4 Hz, 1H), 2.85 (t, J = 7.1 Hz 2H), 2.77 (t, J = 7.6 Hz 2H), 2.31 (m, 2H). ^{13}C NMR (101 MHz, $CDCl_3$) δ 174.01,

152.95, 151.94, 143.31, 142.25, 141.15, 130.48, 127.73, 127.04, 124.92, 119.92, 107.79, 67.95, 46.76, 30.10, 28.72, 27.65. MS (ESI): m/z calculated for $C_{23}H_{19}NO_4S$ [M+H]= 405.104, found = 406,050.

Resynthesis of >98.0% purity compounds 36 and 44

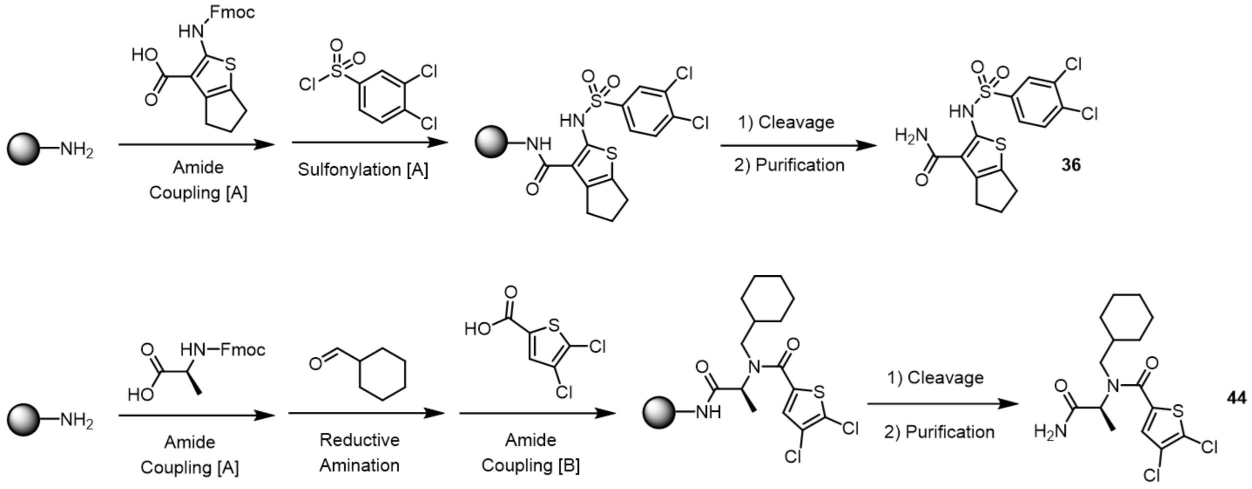

2-((3,4-dichlorophenyl)sulfonamido)-5,6-dihydro-4H-cyclopenta[b]thiophene-3-carboxamide (36)

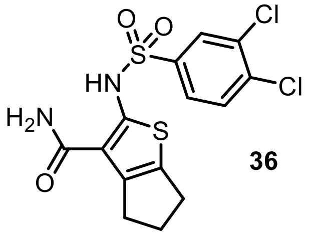

36

Resynthesis of **36** was performed at 3-fold scale (180 µmoles) according to the protocol for scaffold B compounds. Following linker cleavage, the crude extract was subject to normal phase column chromatography (0-5% MeOH in DCM), followed by reverse phase column chromatography (5-100% MeCN in MilliQ H_2O, 0.1% TFA). Product fractions were lyophilized to dryness to give 2-((3,4-dichlorophenyl)sulfonamido)-5,6-dihydro-4H-cyclopenta[b]thiophene-3-carboxamide as a white solid (7.5 mg, 20.7 µmoles, 12%).

^{1}H NMR (400 MHz, d_6-DMSO) δ 7.96 (d, J = 2.2 Hz, 1H), 7.87 (d, J = 8.5 Hz, 1H), 7.71 (d, J = 8.5 Hz, 2.2 Hz, 1H), 2.78 – 2.81 (m, 4H), 2.27 – 2.20 (m, 2H) ppm. ^{13}C NMR (125 MHz, d_6-DMSO) δ 167.86, 165.13, 141.99, 134.04, 131.72, 128.60, 127.06, 126.70, 126.21, 120.79, 111.21, 29.29, 28.77, 26.96 ppm. MS (ESI): m/z calculated for $C_{14}H_{12}Cl_2N_2O_3S_2$ [M+H]= 389.97, found = 390.80

(S)-N-(1-amino-1-oxopropan-2-yl)-4,5-dichloro-N-(cyclohexylmethyl) thiophene-2-carboxamide (44)

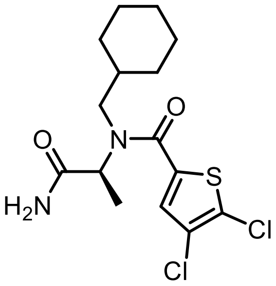

44

Resynthesis of **44** was performed at 3-fold scale (180 µmoles) according to the protocol for scaffold E compounds. Following linker cleavage, the crude extract was subject to normal phase column chromatography (0-5% MeOH in DCM), followed by reverse phase column chromatography (5-100% MeCN in MilliQ H_2O, 0.1% TFA). Product fractions were lyophilized to dryness to give (S)-N-(1-amino-1-oxopropan-2-yl)-4,5-dichloro-N-(cyclohexylmethyl) thiophene-2-carboxamide as a white solid (6.6 mg, 16.9 µmoles, 9%).

^{1}H NMR (400 MHz, d_6-DMSO) δ 7.50 (s, 1H), 4.08 (app s, 1H), 3.42 (app s, 2H), 1.71 – 1.61 (m, 6H), 1.39 (d, J = 6.9 Hz, 3H), 1.17 – 1.10 (m, 3H), 0.90 – 0.76 (m, 2H) ppm. ^{13}C NMR (125 MHz, d_6-DMSO) δ 163.42, 162.65, 136.40, 128.37, 127.26, 123.09, 57.53, 36.86, 36.13, 31.11, 30.44, 26.25, 25.83 ppm. MS (ESI): m/z calculated for $C_{15}H_{20}Cl_2N_2O_2S$ [M+H]= 362.06, found = 363.90

Computational Methods:

Building block selection:

The reagent databases of several synthetic chemistry labs in our institution were collated, from which sets of Fmoc-protected amino acids, aldehydes, primary amines, sulfonyl chlorides and carboxylic acid building blocks were extracted using a KNIME workflow. Duplicate entries, incompatible functional group or protecting group combinations, and compounds with unassigned stereochemistry were removed. Physicochemical properties were calculated for all building blocks using CDK toolkit within the molecular properties node of KNIME, and properties were scored against Goldberg rule of 2 parameters (MW ≥ 200 Da, HBD ≥ 2, HBA ≥ 4, logP ≥ 2).[1] Building blocks violating ≤ 3 rules were retained. A curated set of 715 building blocks was finalized, composed of 113 amino acids, 124 aldehydes, 199 primary amines, 94 sulfonyl chlorides and 185 carboxylic acids.

Library Enumeration:

Libraries were enumerated from curated building blocks in a KNIME workflow using reaction SMARTS in RDKit. Physicochemical properties were calculated for all building blocks using CDK toolkit within the molecular properties KNIME node.

	Reaction SMARTS
C-terminus amidation	[#8;A;X1H0-,X2H1:4][#6;A;X3:2]=[O:1]>>[#7;A;X3:3][#6;X3:2]=[O;X1:1]
Amide Coupling	[#7;A;X3;H2,H1;!$(NC=O);!$(NC=CC=O);!$(NC=S);!$(NC=N)!$(N-S):3].[#8;A;X1H0-,X2H1][#6;A;X3:2]=[O:1]>>[#7;A;X3:3][#6;X3:2]=[O;X1:1]
Sulfonamide Coupling	[#7;A;X3;H2,H1;!$(NC=O);!$(NC=CC=O);!$(NC=S);!$(NC=N)!$(N-S):6].[Cl:4][S:1](=[O:3])=[O:2]>>[#7:6][S:1](=[O:2])=[O:3]
Reductive Amination	[#7;A;X3;H2,H1;!$(NC=O);!$(NC=CC=O);!$(NC=S);!$(NC=N)!$(N-S):3].[#6;A;H1X3:2]=[O:1]>>[#6:2]-[#7:3]
S_NAr	[#7:1]-[#6:2](=[O:3])-[c:4]1[c:5][c:6][c:7](F)[c:8]([c:10]1)-[#7+](-[#8-])=O.[#7;A;H2X3;!$(NC=[!#6]);!$(N-S):15]>>[#7:1]-[#6:2](=[O:3])-[c:4]1[c:5][c:6][c:7](-[#7:15])[c:8]([c:10]1)-[#7+](-[#8-])=O
Nitro Reduction	[#7:9]-[#6:7](=[O:10])-[c:5]1[c:4][c:3][c:2][c:1]([c:6]1)-[#7+](-[#8-])=O>>[#7:9]-[#6:7](=[O:10])-[c:5]1[c:4][c:3][c:2][c:1](-[#7:8])[c:6]1
S_N2 (Primary amine + acetyl bromide)	[#7;A;X3;H2:1].[#6;A;X4;H3,H2:2][Br:3]>>[#7;A;X3;H1:1][#6;A;X4:2]
Heterocyclisation	[#7:11]-[#6:9](=[O:10])-[c:6]1[c:5][c:4][c:2](-[#7:18])[c:3](-[#7:8])[c:7]1.[#6:13][#6;A;H1X3:12]=[O:15]>>[#6:13]-

	[c:12]1[n:18][c:2]2[c:4][c:5][c:6]([c:7][c:3]2[n:8]1)-[#6:9](-[#7:11])=[O:10]
1,6-cyclisation	[Br:1][#6;H2,H3:2][#6:3](=[#8])[#7:4][#6:5][#6:6](=[#8])[#7;H1:7]>>[#6:2]1[#6:3](=[#8])[#7:4][#6:5][#6:6](=[#8])[#7:7]1
	Deprotection SMARTS
Fmoc	[#7:17]-[#6:16](=[O:18])-[#8:15]-[#6:14]-[#6:1]-1-[c:5]2[c:9][c:8][c:7][c:6][c:4]2-[c:3]2[c:10][c:11][c:12][c:13][c:2]-12>>[#7:17]
Trityl (amine)	[N:13][C:14]([c:12]1[c:1][c:2][c:3][c:4][c:5]1)([c:11]1[c:6][c:7][c:8][c:9][c:10]1)[c:15]1[c:16][c:17][c:18][c:19][c:20]1>>[N;A;X3,H2:13]
Trityl (aromatic amine)	[n:13][C:14]([c:12]1[c:1][c:2][c:3][c:4][c:5]1)([c:11]1[c:6][c:7][c:8][c:9][c:10]1)[c:15]1[c:16][c:17][c:18][c:19][c:20]1>>[n;A;X3,H1:13]
Boc	[N:3]-[#6:4](=[O:10])-[#8:5][C:6]([#6:7])([#6:8])[#6:9]>>[N;A;X3;H2:3]
Boc (aromatic)	[n:3]-[#6:4](=[O:10])-[#8:5][C:6]([#6:7])([#6:8])[#6:9]>>[n;A;X3;H1:3]
tBu	[#6;H3:5][C:2]([#6;H3:4])([#6;H3:3])[#8:1]>>[#8:1]
Alloc amine	[#7:7]-[#6:1](=[O:2])-[#8:3]-[#6:4]-[#6:5]=[#6:6]>>[#7:7]
Alloc carboxylic acid	[#6:7]-[#6:1](=[O:2])-[#8:3]-[#6:4]-[#6:5]=[#6:6]>>[#6:7]-[#6:1](-[#8:3])=[O:2]
Trityl (N,O,S)	[#7,#8,#16;A:13][C:14]([c:12]1[c:1][c:2][c:3][c:4][c:5]1)([c:11]1[c:6][c:7][c:8][c:9][c:10]1)[c:15]1[c:16][c:17][c:18][c:19][c:20]1>>[#7,#8,#16;A:13]
Pbf	[#6:11]-[#8:10]-[c:9]1[c:8][c:6](-[#6:7])[c:5]([c:14](-[#6:15])[c:12]1-[#6:13])[S:2]([#7:1])(=[O:3])=[O:4]>>[#7:1]

Molecular docking:

A docking model for CCR2 was prepared from a ligand-bound structure of CCR2 (PDB accession code 5T1A). Examination of the targeted VT5 binding pocket indicated that protein–ligand interactions occurred exclusively through direct contacts with the amino acid residues and backbone of CCR2. Therefore, lysozyme, ions, and water molecules were removed from the structure. A review of mutations introduced to facilitate crystallization identified several substitutions in the 5T1A structure that deviated from the canonical sequence of CCR2 isoform B. Reverse mutations Glu238Arg, Arg237Lys, and Lys240Arg were performed to better reflect the composition of the VT5 pocket in the canonical isoform.[2] Rotamers for the mutated residues were generated using the mutagenesis tool implemented in PyMOL, and the resulting CCR2 model was processed using the ADFR software suite to generate an AutoDock VINA input file with hydrogen atoms added.

Members of V-SYNTHES representative libraries were docked into a grid at 16.2 x 17.8 x 17.4 Å^3 with an exhaustiveness value of 8. Predicted binding affinity of ≤-8.0 kcal/mol was applied as a post-docking filter for selection of virtual screening hits.

Interaction Fingerprint Analysis and Scoring

The scoring function was generated in the form of a weighted average:

$$IFPscore_i = \frac{w_{required}p_{required} + w_{essential}p_{essential} + w_{interesting}p_{interesting}}{w_{required} + w_{essential} + w_{interesting}}$$

where i denotes a single docked pose and w and p are the weight and interaction percentage, respectively. The weights are determined based on observing the fractions of PLIP-calculated molecular interactions for the top poses of the top 5 most active compounds recorded in literature for each identified chemical series. The interactions were divided into three groups: (1) required (2) essential and (3) interesting. The first group is comprised of interactions common for nearly all of the identified poses, the second of interactions that we regard as essential for high activity and include interactions with moieties commonly identified in literature as increasing binding to the intracellular side of CCR2 and the third of interactions that are less common in active compounds, but we postulate that they could further enhance CCR2 binding. The weights assigned to these three terms were 1.5, 1.0 and 0.5, respectively. This was done empirically based on the correlation of this score with experimental data. The percentages are determined for each evaluated top pose by calculating how many interactions of the given type are found by PLIP. Allocated interaction fingerprint score was used to prioritize virtual screening hits as cluster representatives for visual inspection.

The complete code implementing the elucidation of the workflow is available in the associated data package and GitHub.[3]

Tanimoto Similarity Scoring

To score validated virtual screening hits on similarity, 100 allosteric ligands with reported nanomolar binding affinity for CCR2 were pulled as reference compounds from BindingDB. Tanimoto similarity scores were assigned based on Morgan fingerprints. A table of reference compounds is included as appendix table S1.

Biological Materials & Methods:

Materials

Human recombinant chemokine CCL2 was purchased from PeproTech (Rocky Hill, NJ). All screening compounds were synthesized in-house. [^{3}H]-CCR2-RA-[*R*] (specific activity 59.6 Ci mmol^{-1}) was custom-labeled by Vitrax (Placentia, CA). Bovine serum albumin was purchased from SigmaAldrich (St. Louis, MO). Bicinchoninic acid (BCA) and Pierce BCA protein assay kit were purchased from Pierce Biotechnology (Thermo Scientific, Rockford, IL). Tango U2OS cells stably expressing human CCR2b (U2OS-CCR2) were purchased from Invitrogen (Carlsbad, CA). All other chemicals were obtained from standard commercial sources.

Cell Culture

U2OS-CCR2b cells were cultured in McCoy's 5A medium supplemented with 10% (v/v) fetal calf serum, 2 mM glutamine, 0.1 mM nonessential amino acids, 25 mM HEPES, 1 mM sodium pyruvate, 200 IU/mL penicillin, 200 µg/ mL streptomycin, 100 µg/mL G418, 40 µg/mL hygromycin B gold, and 125 µg/mL zeocin. Cells were grown until 80% confluence and cultured twice-weekly on 10 or 15 cm ⌀ plates by trypsinization. Dialyzed fetal calf serum was used when culturing cells for functional assays or as a last step before membrane preparation.

Membrane Preparation

Membranes from U2OS-CCR2 cells were prepared as previously described for CCR2.[4] Briefly, U2OS-CCR2 cells were scraped from confluent 15 cm ⌀ plates using phosphate buffered saline (PBS) and subsequently centrifuged at 1000 × g for 5 min. Pellets were then resuspended in ice-cold Tris buffer (50 mM Tris-HCl, 5 mM MgCl2, pH 7.4) before homogenization with an Ultra Turrax homogenizer (IKA-Werke GmbH & Co. KG, Staufen, Germany). Membranes and cytosolic contents were separated using an Optima LE-80 K ultracentrifuge (Beckman Coulter, Inc., Fullerton, CA) at 100 000 × g for 20 min at 4 °C. After a second cycle of homogenization and centrifugation, the final pellet was resuspended and homogenized in ice-cold Tris buffer, aliquoted, and stored at −70 °C. Finally, membrane protein concentrations were determined using a BCA protein determination assay, as described by the manufacturer (Pierce BCA protein assay kit).

[^{3}H]CCR2-RA-[*R*] Binding Assays

For [^{3}H]CCR2-RA-[*R*] displacement assays, U2OS-CCR2b membrane homogenates (10–20 µg total protein) were incubated with approximately 6 nM [^{3}H]CCR2-RA-[*R*] in the presence of competing ligands in a final volume of 100 µL of assay buffer (50 mM Tris-HCl, 5 mM

$MgCl_2$, 0.1% CHAPS, pH 7.4). For single-point screening experiments, compounds were tested at a concentration of 100 μM, and specific binding was expressed as percent displacement relative to total and nonspecific binding controls. For affinity determination, at least six increasing concentrations of competing ligand were tested to generate full curves. Ligands were diluted to the desired concentrations using an HP D300 digital dispenser (Tecan, Giessen, The Netherlands). Total radioligand binding did not exceed 10% of the amount added to prevent ligand depletion, and nonspecific binding was determined in the presence of 10 μM JNJ-2714191. The final DMSO concentration was ≤1%. After incubation for 2 h at 25 °C, binding was terminated by rapid filtration through a 96-well GF/C filter plate using a PerkinElmer FilterMate harvester and ice-cold wash buffer (50 mM Tris-HCl, 5 mM $MgCl_2$, 0.05% CHAPS, pH 7.4). Filters were washed ten times with ice-cold wash buffer and dried at 55 °C for 30 min. Following addition of 25 μL Microscint scintillation cocktail (PerkinElmer) and 3 h incubation, filter-bound radioactivity was quantified by scintillation counting using a P-E 2450 $MicroBeta^2$ counter (PerkinElmer).

Tango β-Arrestin Recruitment Assay

β-Arrestin recruitment was measured using the Tango CCR2-bla cell based assay (Invitrogen) according to the manufacturer's protocol. Briefly, U2OS-CCR2b cells were grown until approximately 80% confluence and detached by trypsinization. Cells were recovered by centrifugation at 200 × g for 5 min, resuspended in assay medium (FreeStyle Expression Medium, Invitrogen) to a density of 40 000 cells per well and seeded into black-wall, clearbottom, 96-well assay plates (Corning).. Cells were preincubated with increasing concentrations of antagonist diluted in assay medium for 30 min at room temperature, before a 16 hour co-incubation with an EC_{80} concentration of CCL2 (0.4 nM) at 37 °C and 5% CO_2. The final DMSO concentration in the assay was 0.25%. After 16 h cells were loaded in the dark with 32 μL of LiveBLAzer-FRET B/G substrate (Invitrogen) and incubated for 2 h at room temperature. Finally, fluorescence emission at 460 and 535 nm was measured in an EnVision multilabel plate reader (PerkinElmer) after excitation at 400 nm. The ratio of emission at 460 and 535 nm was calculated for each well.

Data Analysis

All experiments were analyzed using nonlinear regression curve fitting program Graphpad Prism 10 (Dotmatics, Boston, MA). K_i, EC_{50}, EC_{80}, E_{max}, and IC_{50} values from binding and functional assays were obtained by nonlinear regression analysis. All values obtained are the mean ± SEM of at least three separate experiments performed in duplicate, unless stated otherwise. For radioligand binding assays, K_i values were determined using the Cheng–Prussoff equation using a K_D of 6.3 nM for the radioligand.[5]

Building Blocks

Amino Acids

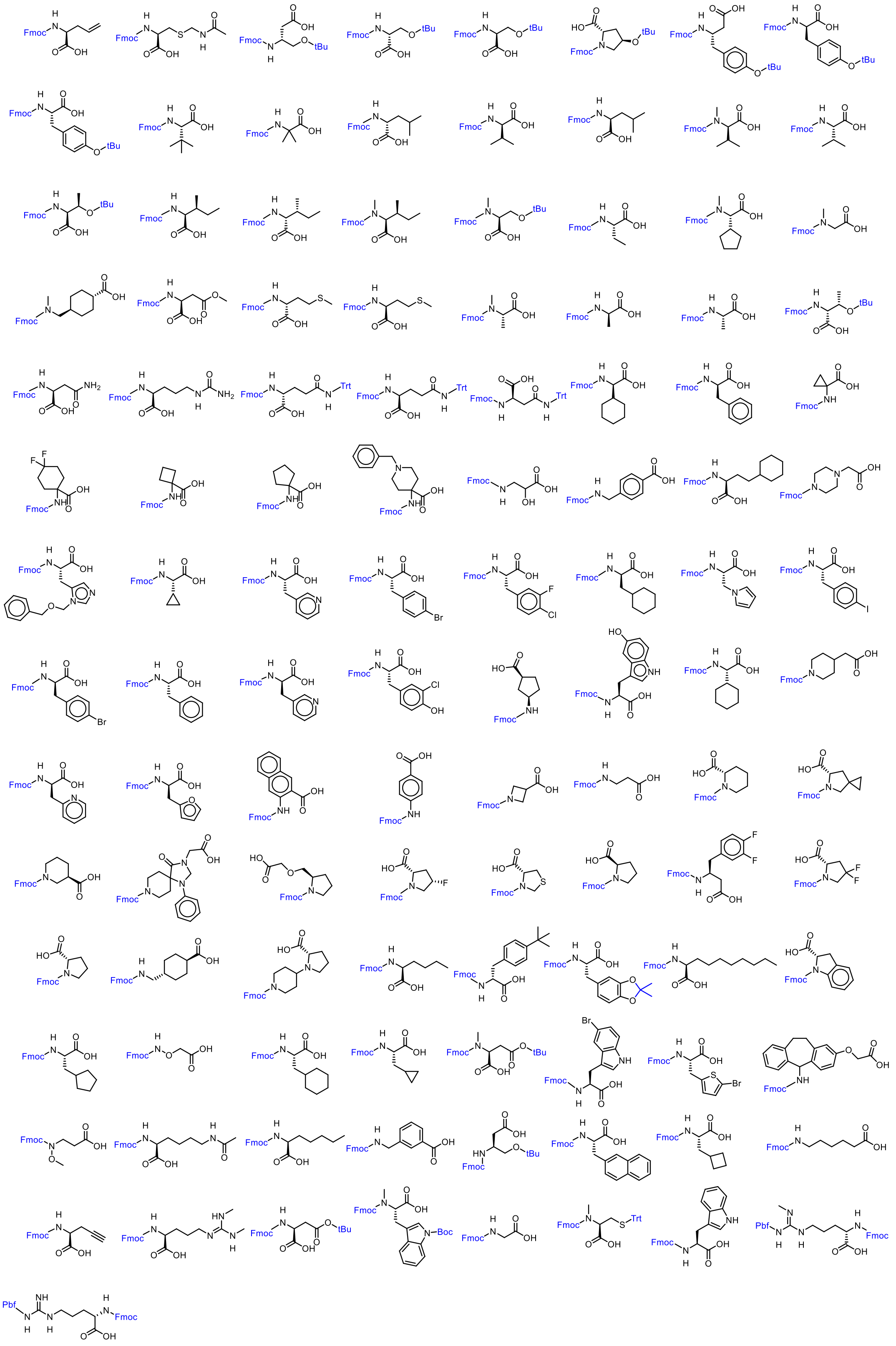

Aldehydes

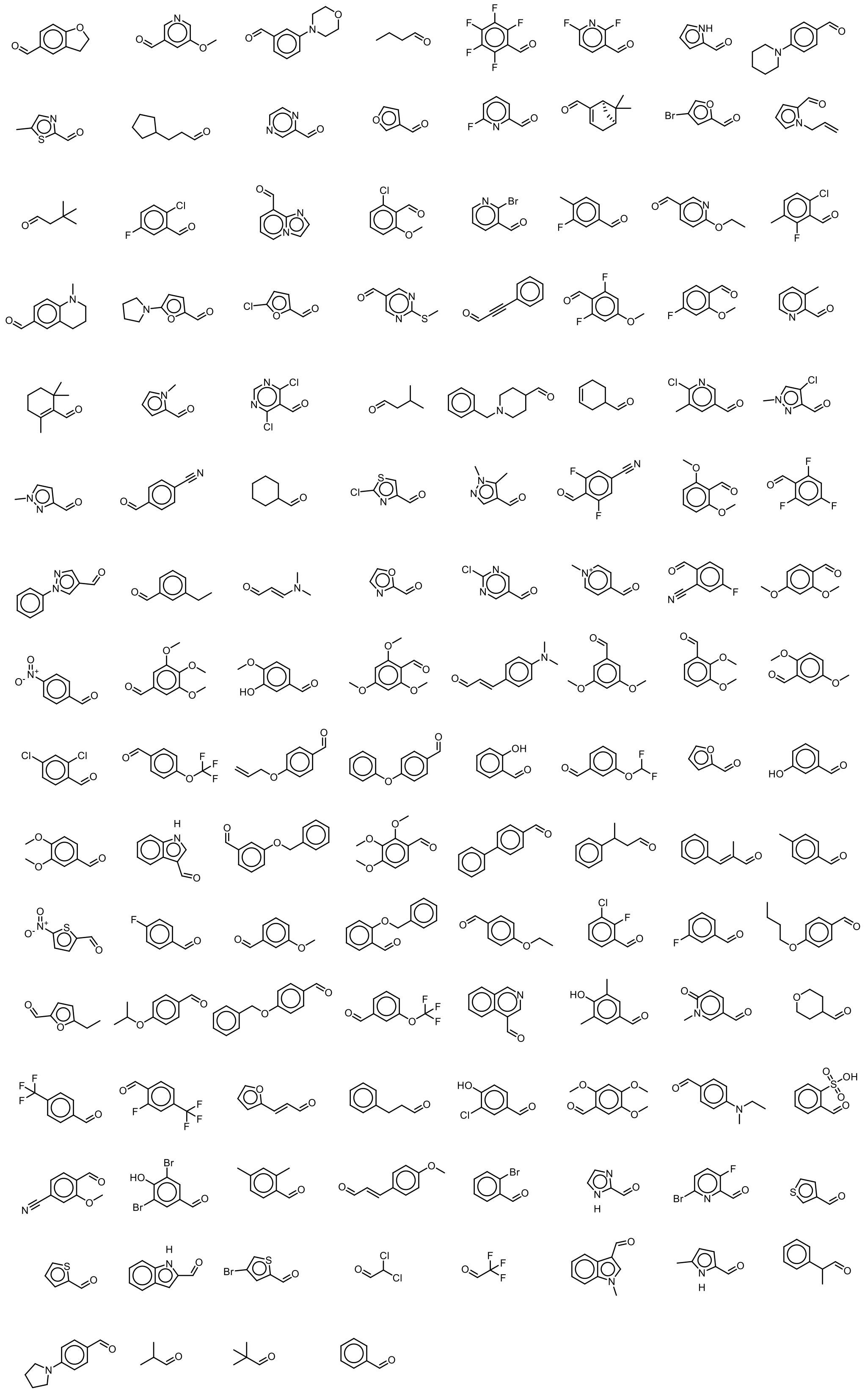

Carboxylic acids

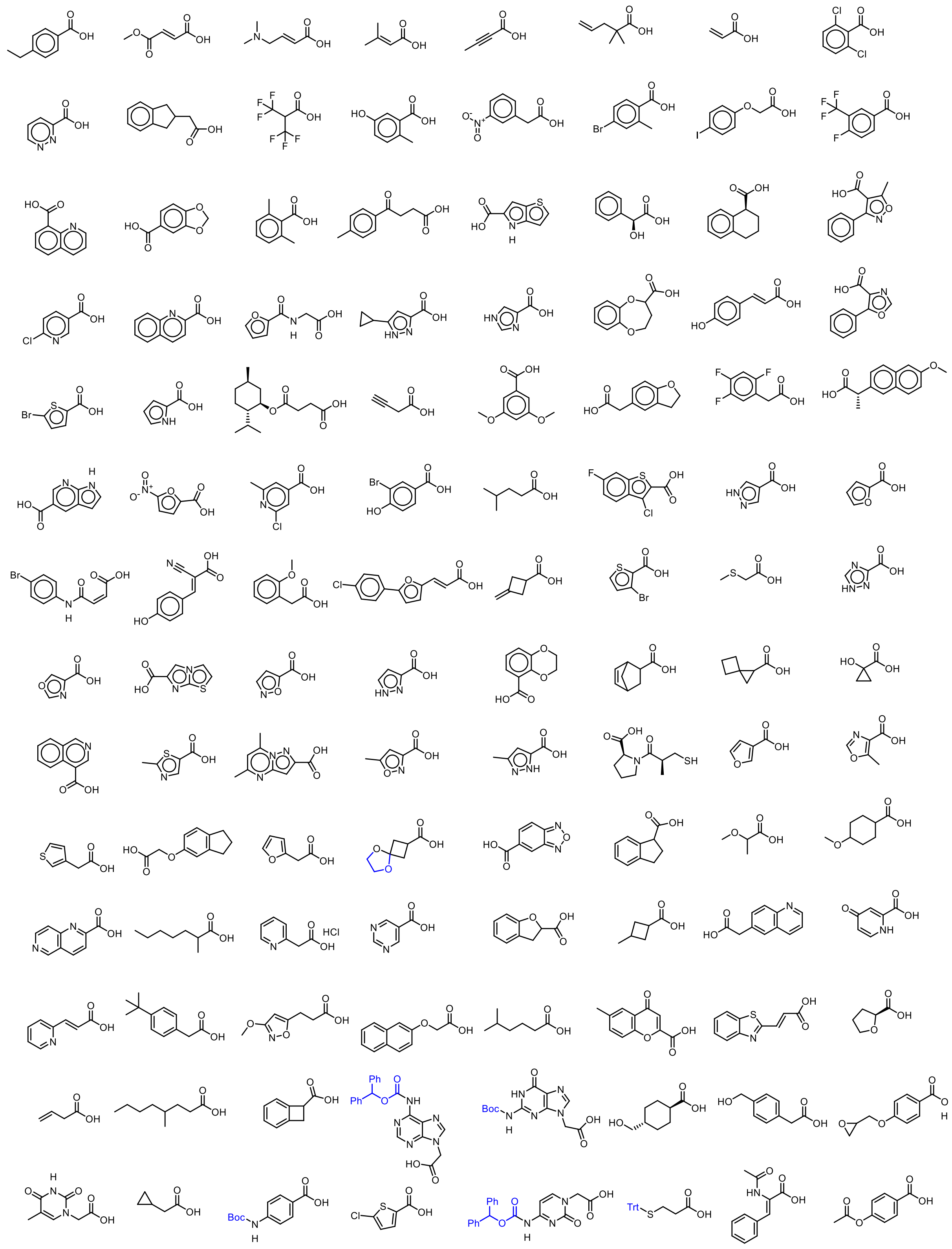

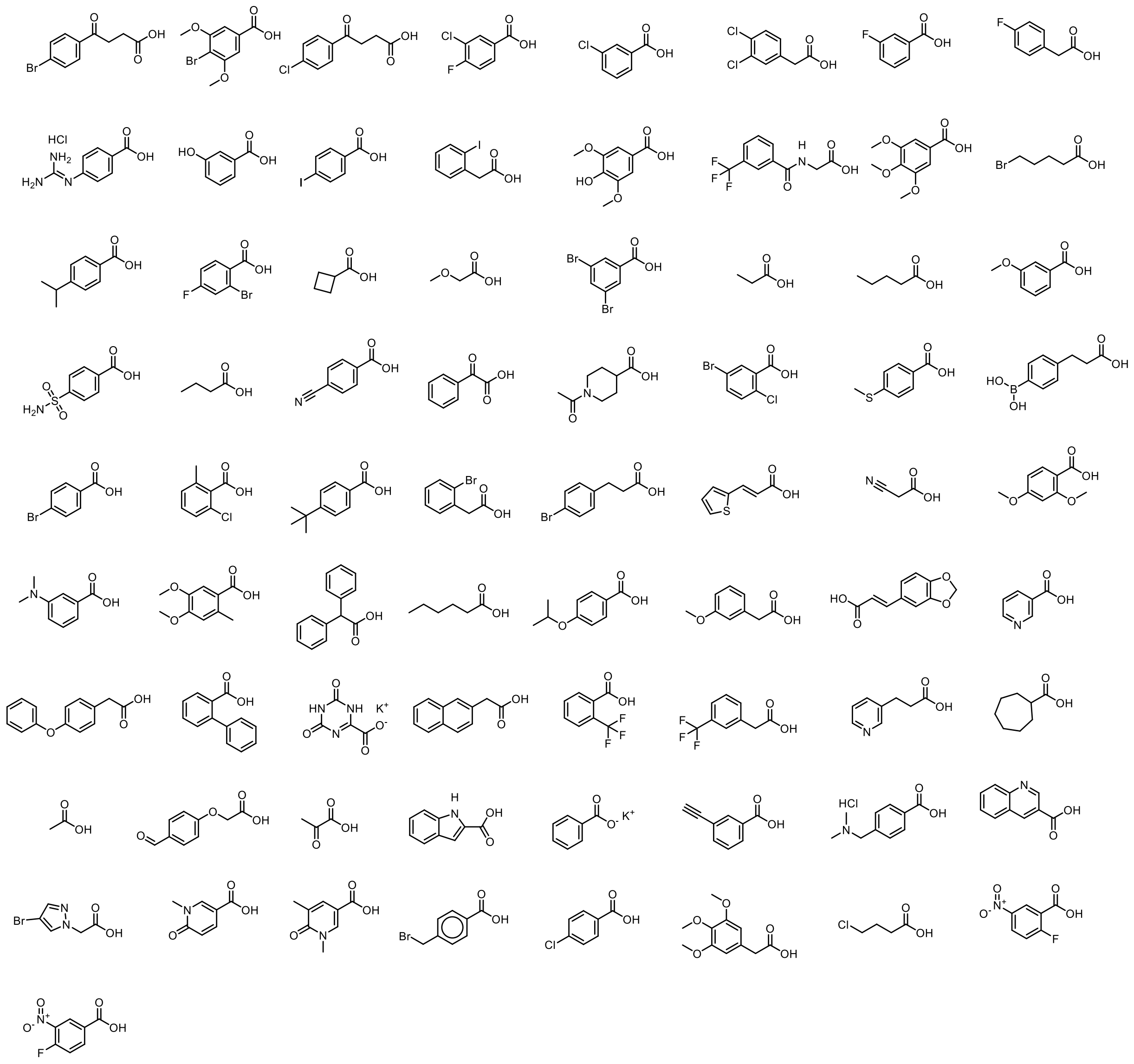

Primary Amines

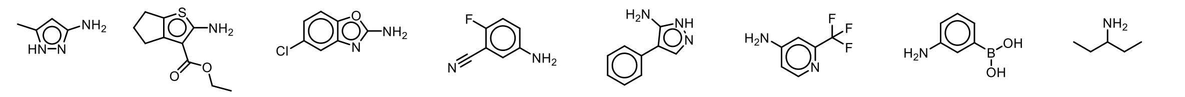

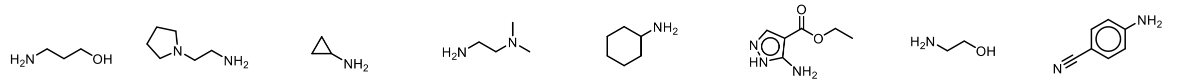

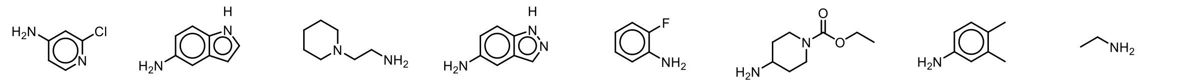

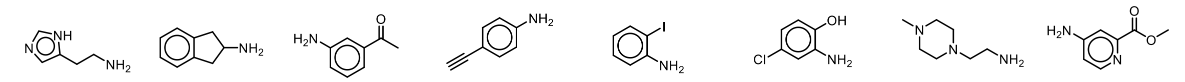

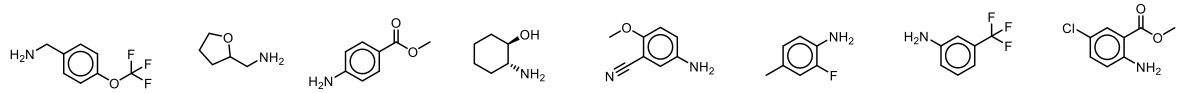

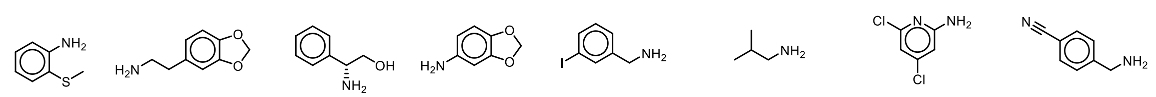

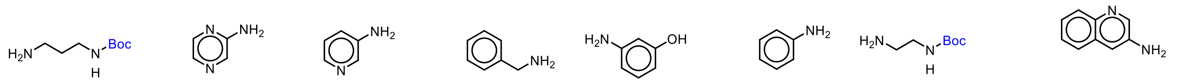

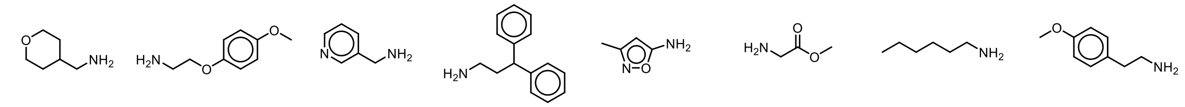

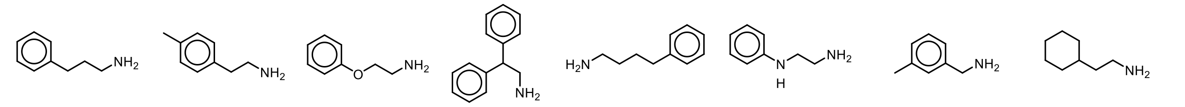

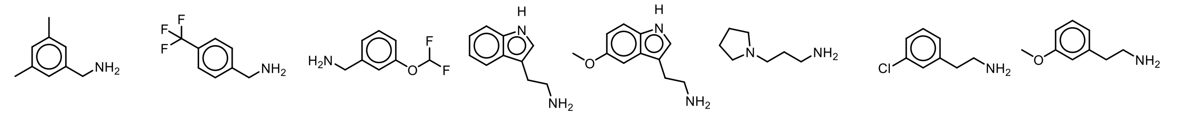

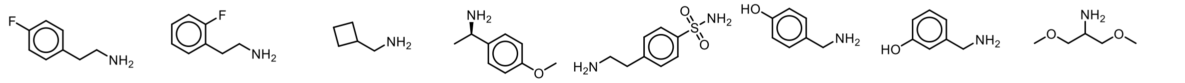

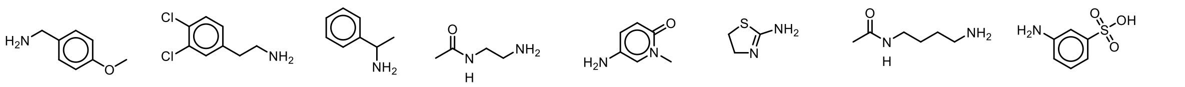

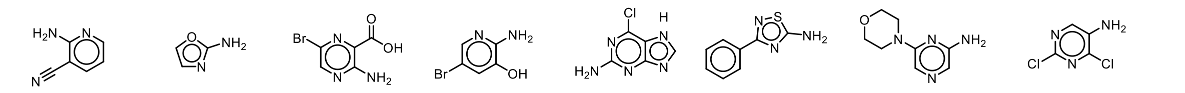

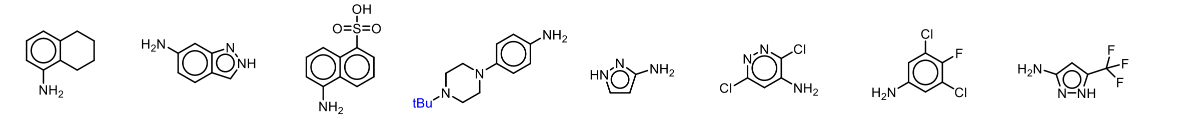

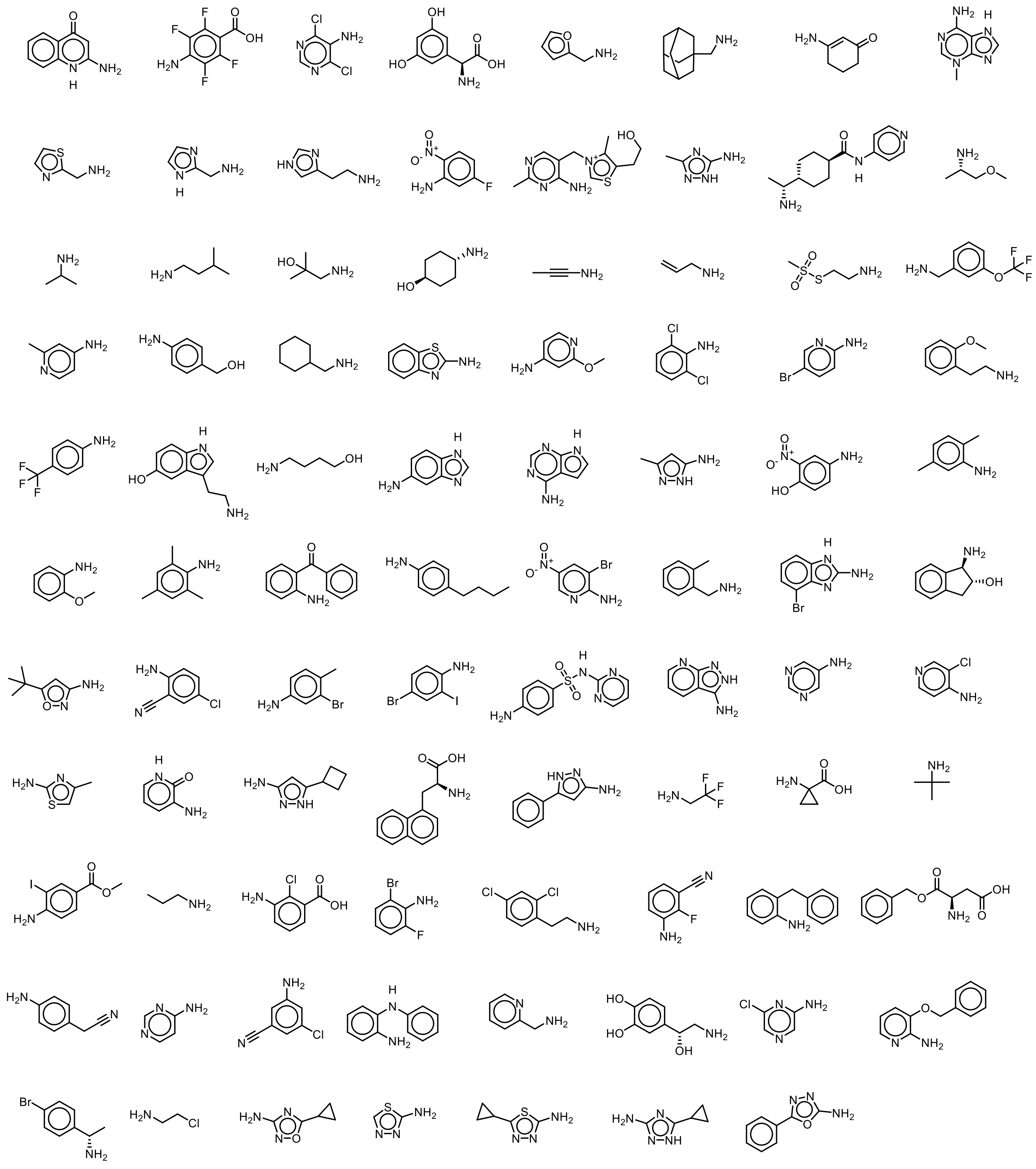

Sulfonyl chlorides

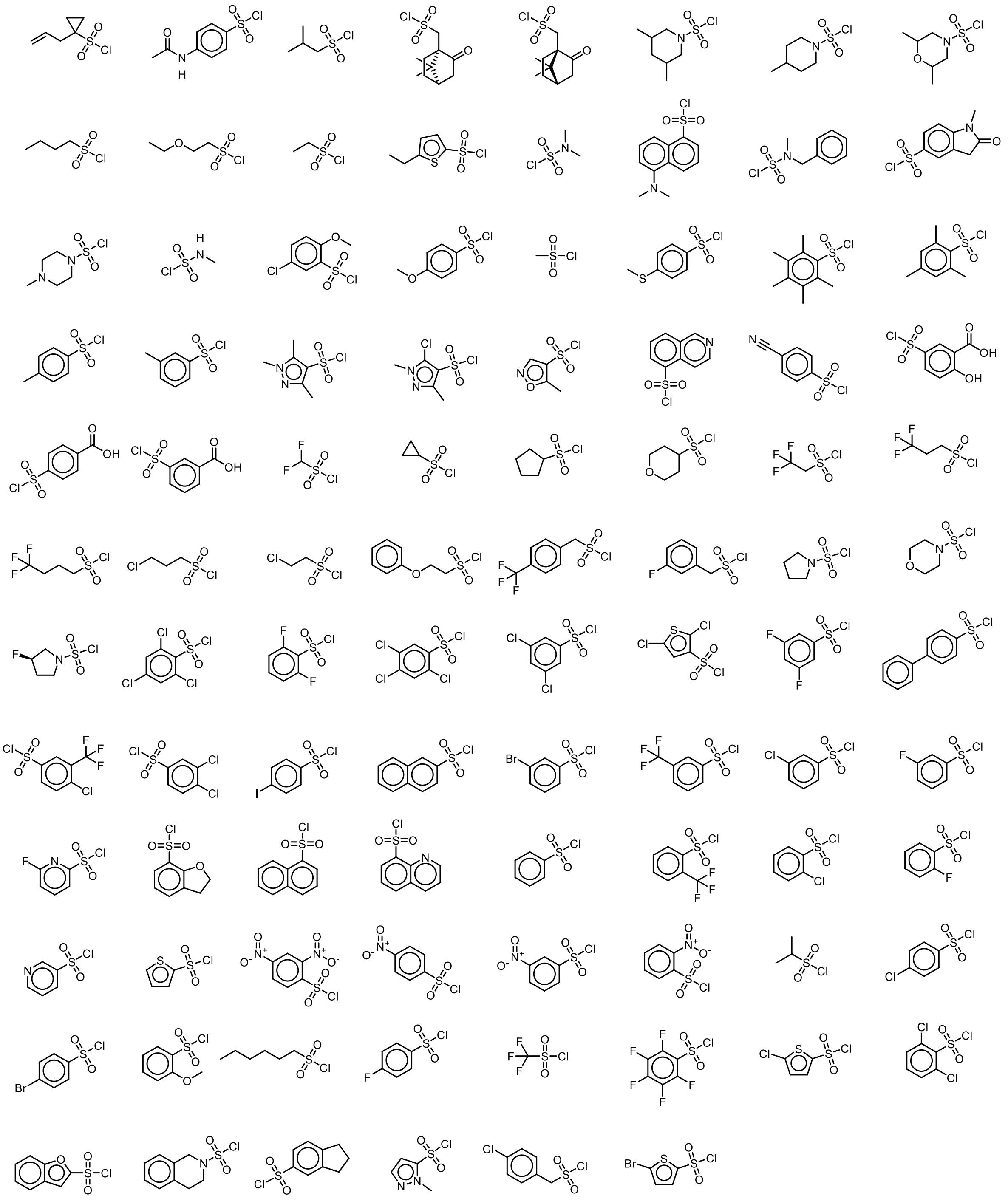

Analytical Data

Substrate Scope Screen by HPLC-MS

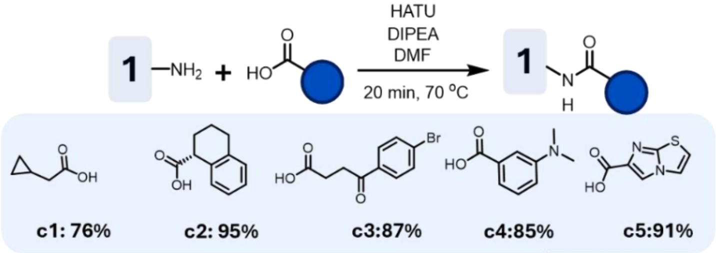

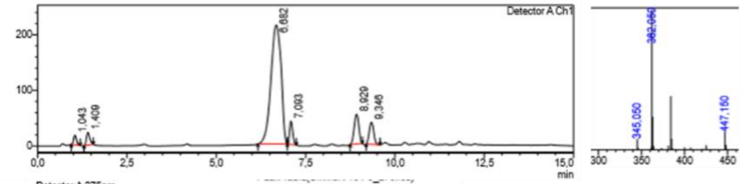

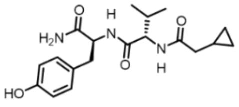

Exact Mass: 361.2002

Observed $[M+H]^+$=362,050

Detector A 275nm

Peak#	Ret. Time	Area	Height	Mark	Conc.	Area%
1	1,043	96176	17372	M	1,741	1,741
2	1,409	149124	22702	M	2,699	2,699
3	6,682	4211760	213355	M	76,238	76,238
4	7,093	250678	41391	M	4,538	4,538
5	8,929	484800	53338	M	8,776	8,776
6	9,346	331916	39439	M	6,008	6,008
Total		5524454	387598		100,000	100,000

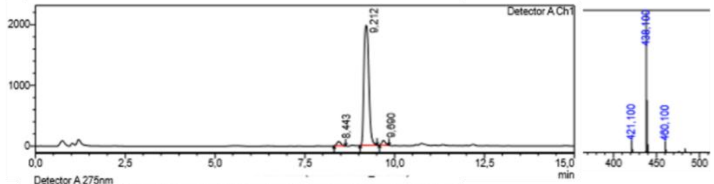

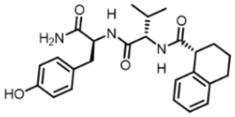

Exact Mass: 437.2315

Observed $[M+H]^+$=438,100

Detector A 275nm

Peak#	Ret. Time	Area	Height	Mark	Conc.	Area%
1	8,443	551693	71645	M	2,771	2,771
2	9,212	18815632	1973928	M	94,500	94,500
3	9,690	543487	75905	M	2,730	2,730
Total		19910813	2121480		100,000	100,000

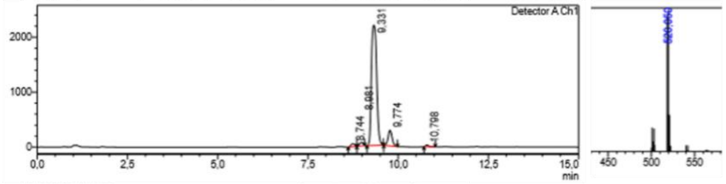

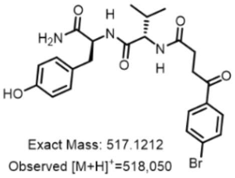

Exact Mass: 517.1212

Observed $[M+H]^+$=518,050

Detector A 275nm

Peak#	Ret. Time	Area	Height	Mark	Conc.	Area%
1	8,744	393086	55445	M	1,577	1,577
2	8,981	434569	65548	M	1,743	1,743
3	9,331	21718267	2186136	M	87,111	87,111
4	9,774	2189105	284558	M	8,780	8,780
5	10,798	196686	29837	M	0,789	0,789
Total		24931713	2621524		100,000	100,000

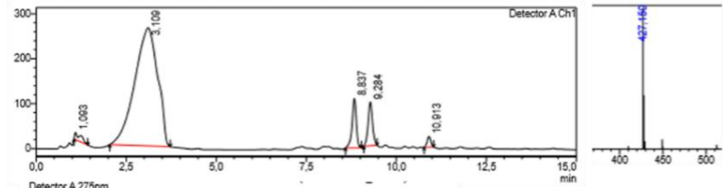

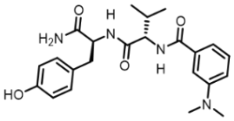

Exact Mass: 426.2267

Observed $[M+H]^+$=427,150

Detector A 275nm

Peak#	Ret. Time	Area	Height	Mark	Conc.	Area%
1	1,093	194693	15889	M	1,417	1,417
2	3,109	11659627	262700	M	84,844	84,844
3	8,837	895397	109651	M	6,516	6,516
4	9,284	824156	96974	M	5,997	5,997
5	10,913	168629	23372	M	1,227	1,227
Total		13742501	508586		100,000	100,000

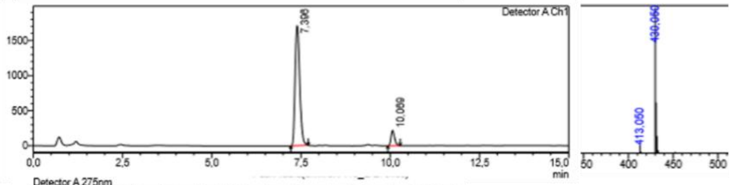

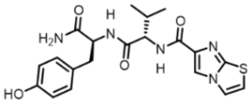

Exact Mass: 429.1471

Observed $[M+H]^+$=430,050

Detector A 275nm

Peak#	Ret. Time	Area	Height	Mark	Conc.	Area%
1	7,396	15406276	1707513	M	91,145	91,145
2	10,069	1496753	215929	M	8,855	8,855
Total		16903029	1923442		100,000	100,000

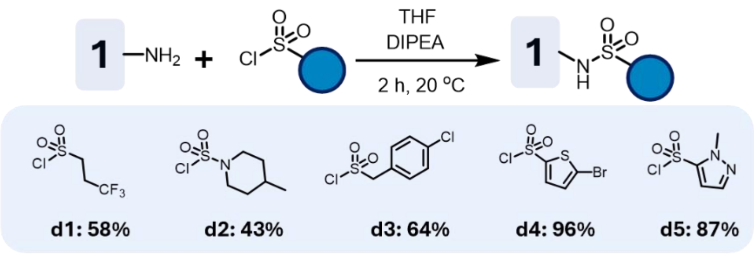

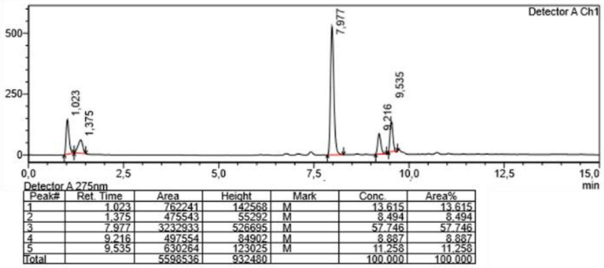

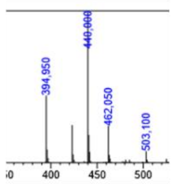

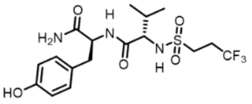

Exact Mass: 439.1389
Observed [M+H]$^+$=440,000

Detector A 275nm

Peak#	Ret. Time	Area	Height	Mark	Conc.	Area%
1	1.023	762241	142568	M	13.615	13.615
2	1.375	475543	55292	M	8.494	8.494
3	7.977	3232933	526695	M	57.746	57.746
4	9.216	497554	84902	M	8.887	8.887
5	9.535	630264	123025	M	11.258	11.258
Total		5598536	932480		100.000	100.000

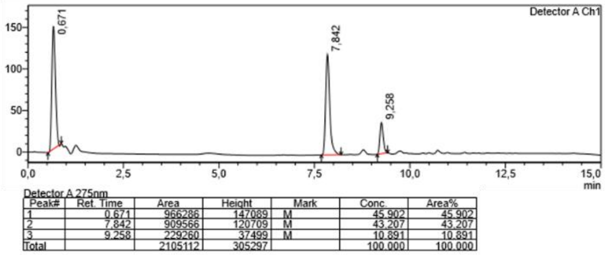

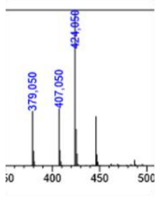

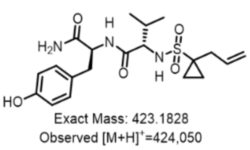

Exact Mass: 423.1828
Observed [M+H]$^+$=424,050

Detector A 275nm

Peak#	Ret. Time	Area	Height	Mark	Conc.	Area%
1	0.671	966286	147089	M	45.902	45.902
2	7.842	909566	120709	M	43.207	43.207
3	9.258	229260	37499	M	10.891	10.891
Total		2105112	305297		100.000	100.000

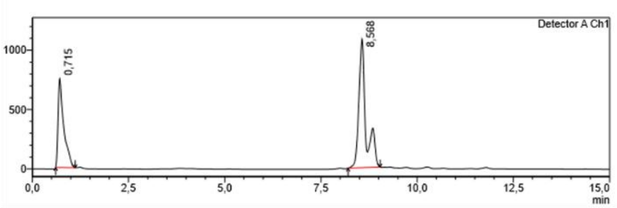

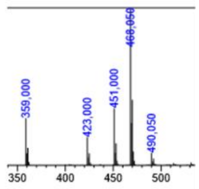

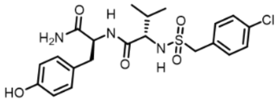

Exact Mass: 467.1282
Observed [M+H]$^+$=468,050

Detector A 275nm

Peak#	Ret. Time	Area	Height	Mark	Conc.	Area%
1	0.715	7729035	750494	M	35.884	35.884
2	8.568	13809835	1083406	M	64.116	64.116
Total		21538870	1833900		100.000	100.000

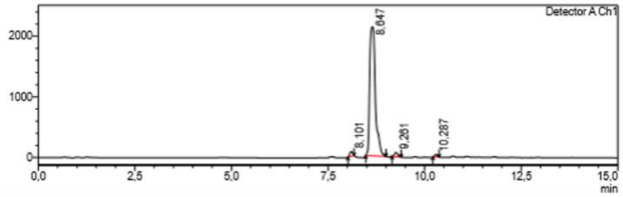

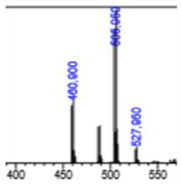

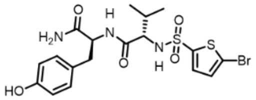

Exact Mass: 503.0184
Observed [M+H]$^+$=503,950

Detector A 275nm

Peak#	Ret. Time	Area	Height	Mark	Conc.	Area%
1	8.101	423400	79879	M	1.858	1.858
2	8.647	21763185	2124388	M	95.521	95.521
3	9.261	395281	69858	M	1.735	1.735
4	10.287	201708	40734	M	0.885	0.885
Total		22783573	2314859		100.000	100.000

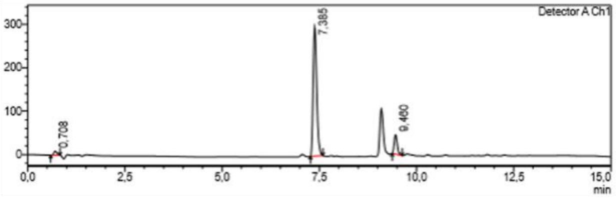

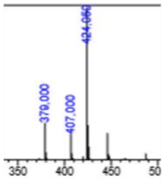

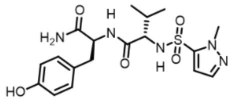

Exact Mass: 423.1576
Observed [M+H]$^+$=424,050

Detector A 275nm

Peak#	Ret. Time	Area	Height	Mark	Conc.	Area%
1	0.708	52235	9214	M	2.385	2.385
2	7.385	1899326	297358	M	86.713	86.713
3	9.460	238790	44428	M	10.902	10.902
Total		2190350	351001		100.000	100.000

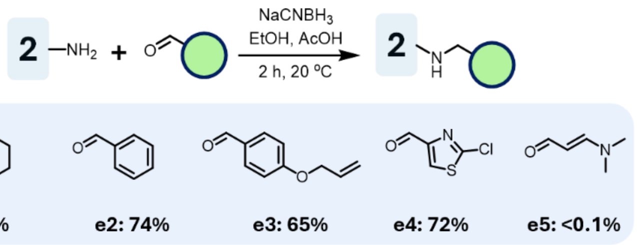

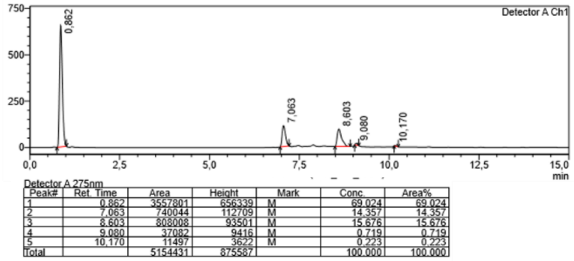

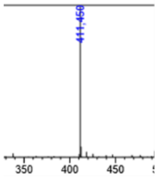

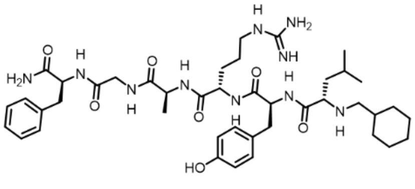

Exact Mass: 820.4959

Observed [M+2H]$^{2+}$= 411,450

Detector A 275nm

Peak#	Ret. Time	Area	Height	Mark	Conc.	Area%
1	0,862	3557801	656339	M	69,024	69,024
2	7,063	740044	112709	M	14,357	14,357
3	8,603	808008	93501	M	15,676	15,676
4	9,080	37082	9416	M	0,719	0,719
5	10,170	11497	3622	M	0,223	0,223
Total		5154431	875587		100,000	100,000

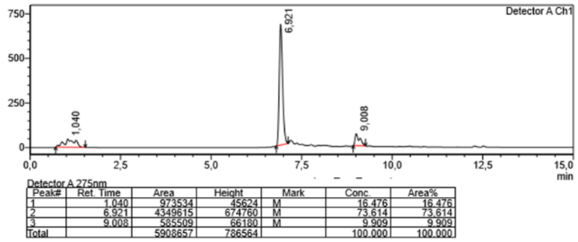

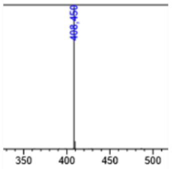

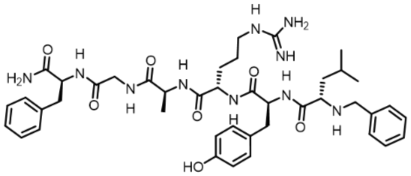

Exact Mass: 814.4490

Observed [M+2H]$^{2+}$= 408,450

Detector A 275nm

Peak#	Ret. Time	Area	Height	Mark	Conc.	Area%
1	1,040	973534	45624	M	16,476	16,476
2	6,921	4349615	674760	M	73,614	73,614
3	9,008	585509	66180	M	9,909	9,909
Total		5908657	786564		100,000	100,000

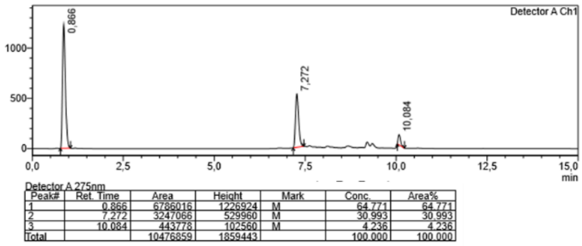

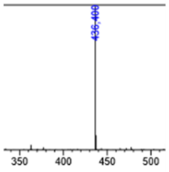

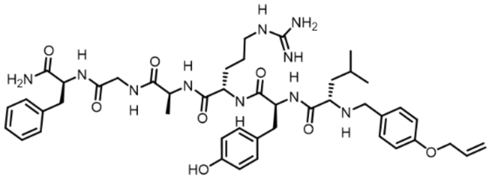

Exact Mass: 870.4752

Observed [M+2H]$^{2+}$= 436,450

Detector A 275nm

Peak#	Ret. Time	Area	Height	Mark	Conc.	Area%
1	0,866	6786016	1226924	M	64,771	64,771
2	7,272	3247066	529960	M	30,993	30,993
3	10,084	443778	102560	M	4,236	4,236
Total		10476859	1859443		100,000	100,000

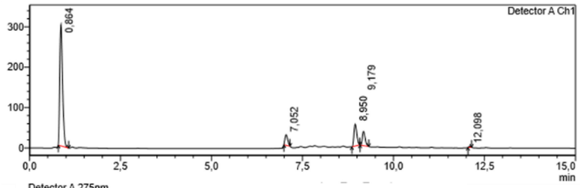

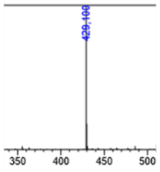

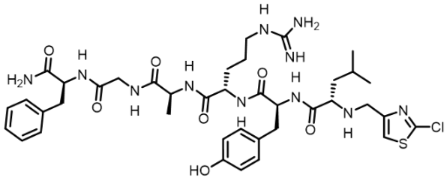

Exact Mass: 855.3617

Observed [M+2H]$^{2+}$= 429,100

Detector A 275nm

Peak#	Ret. Time	Area	Height	Mark	Conc.	Area%
1	0,864	1610725	301047	M	71,841	71,841
2	7,052	139223	25957	M	6,210	6,210
3	8,950	295043	54262	M	13,159	13,159
4	9,179	186613	35049	M	8,323	8,323
5	12,098	10478	2886	M	0,467	0,467
Total		2242082	419200		100,000	100,000

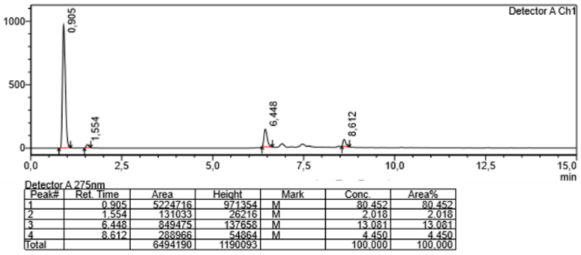

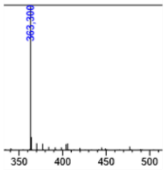

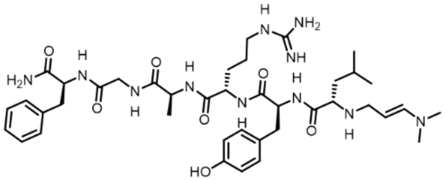

Exact Mass: 807.4755

Not Observed

Detector A 275nm

Peak#	Ret. Time	Area	Height	Mark	Conc.	Area%
1	0,905	5224716	971354	M	80,452	80,452
2	1,554	131033	26216	M	2,018	2,018
3	6,448	849475	137658	M	13,081	13,081
4	8,612	288966	54864	M	4,450	4,450
Total		6494190	1190093		100,000	100,000

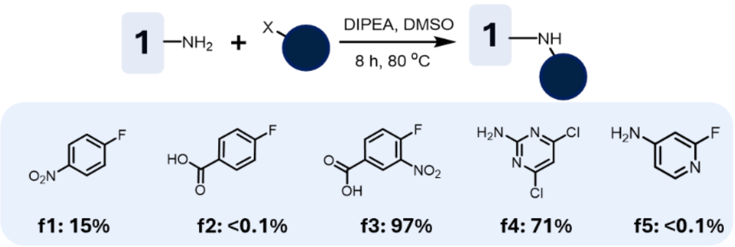

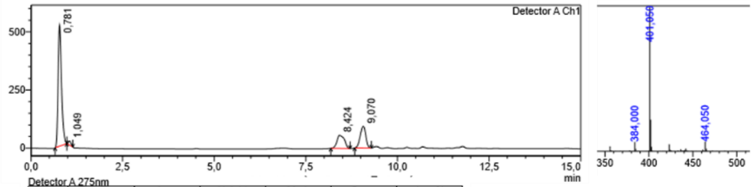

Detector A 275nm

Peak#	Ret. Time	Area	Height	Mark	Conc.	Area%
1	0.781	3372222	518613	M	64.509	64.509
2	1.049	82837	17533	M	1.585	1.585
3	8.424	788532	57035	M	15.084	15.084
4	9.070	983936	93009	M	18.822	18.822
Total		5227527	686190		100.000	100.000

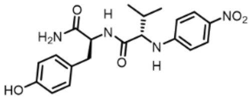

Exact Mass: 400.1747

Observed $[M+H]^+$=401,050

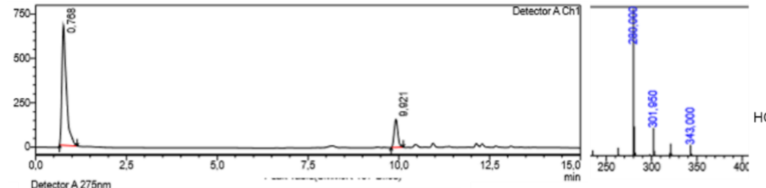

Detector A 275nm

Peak#	Ret. Time	Area	Height	Mark	Conc.	Area%
1	0.768	5739808	674873	M	83.711	83.711
2	9.921	1116920	158367	M	16.289	16.289
Total		6856728	833240		100.000	100.000

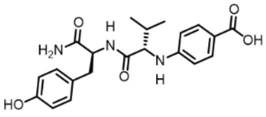

Exact Mass: 399.1794

Not Observed

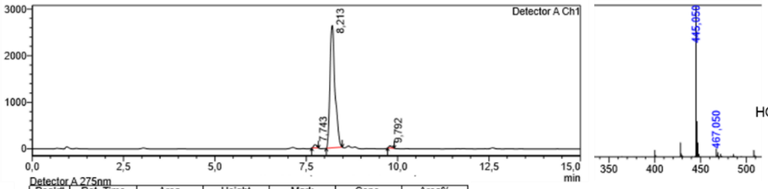

Detector A 275nm

Peak#	Ret. Time	Area	Height	Mark	Conc.	Area%
1	7.743	414104	71211	M	1.584	1.584
2	8.213	25460338	2616719	M	97.402	97.402
3	9.792	265129	43733	M	1.014	1.014
Total		26139571	2731664		100.000	100.000

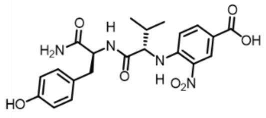

Exact Mass: 444.1645

Observed $[M+H]^+$=445,050

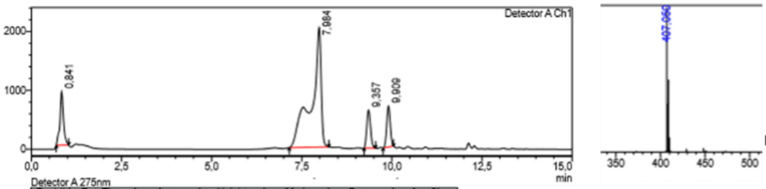

Detector A 275nm

Peak#	Ret. Time	Area	Height	Mark	Conc.	Area%
1	0.841	6292010	909097	M	11.646	11.646
2	7.984	38494056	2028533	M	71.247	71.247
3	9.357	4484080	643357	M	8.299	8.299
4	9.909	4759082	696755	M	8.808	8.808
Total		54029228	4277741		100.000	100.000

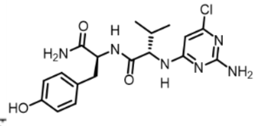

Exact Mass: 406.1520

Observed $[M+H]^+$=440,000

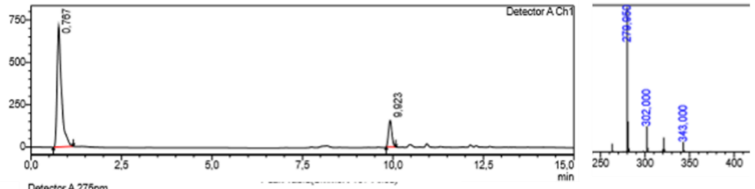

Detector A 275nm

Peak#	Ret. Time	Area	Height	Mark	Conc.	Area%
1	0.767	6046725	715130	M	85.828	85.828
2	9.923	998446	155208	M	14.172	14.172
Total		7045171	870338		100.000	100.000

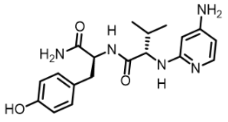

Exact Mass: 371.1957

Not Observed

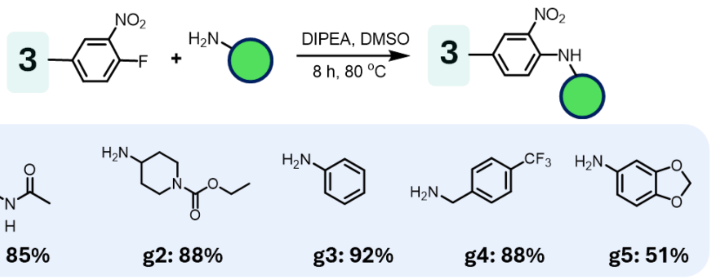

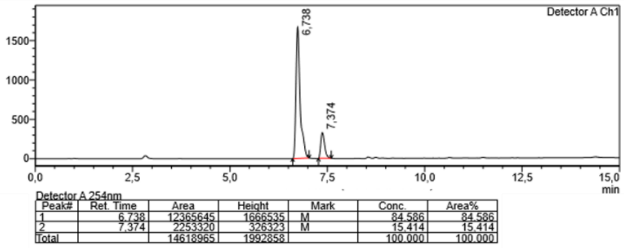

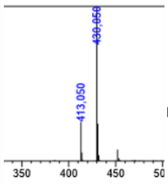

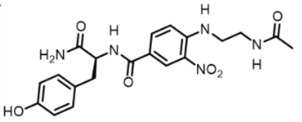

Exact Mass: 429.1648
Observed [M+H]+=430,050

Detector A 254nm

Peak#	Ret. Time	Area	Height	Mark	Conc.	Area%
1	6,738	12365645	1666535	M	84,586	84,586
2	7,374	2253320	326323	M	15,414	15,414
Total		14618965	1992858		100,000	100,000

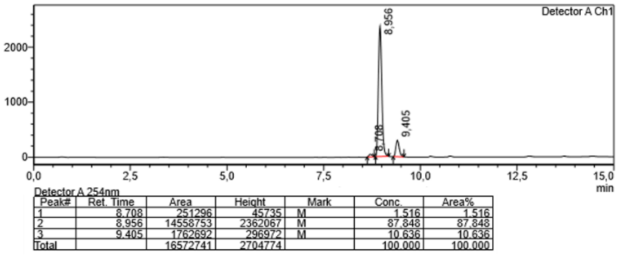

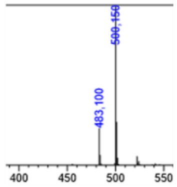

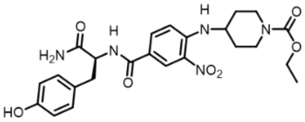

Exact Mass: 499.2067
Observed [M+H]+=500,150

Detector A 254nm

Peak#	Ret. Time	Area	Height	Mark	Conc.	Area%
1	8,708	251296	45735	M	1,516	1,516
2	8,956	14558753	2362067	M	87,848	87,848
3	9,405	1762692	296972	M	10,636	10,636
Total		16572741	2704774		100,000	100,000

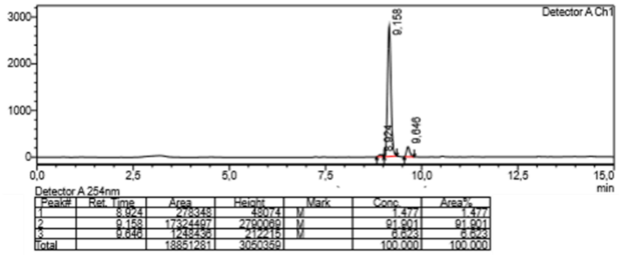

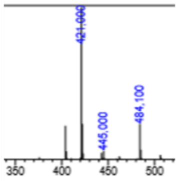

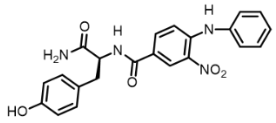

Exact Mass: 420.1434
Observed [M+H]+=421,000

Detector A 254nm

Peak#	Ret. Time	Area	Height	Mark	Conc.	Area%
1	8,924	278348	48074	M	1,477	1,477
2	9,158	17324497	2790069	M	91,901	91,901
3	9,646	1248436	212215	M	6,623	6,623
Total		18851281	3050359		100,000	100,000

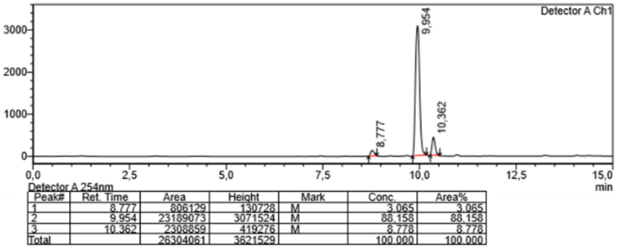

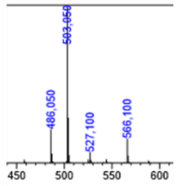

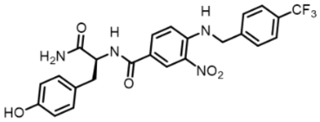

Exact Mass: 502.1464
Observed [M+H]+=503,050

Detector A 254nm

Peak#	Ret. Time	Area	Height	Mark	Conc.	Area%
1	8,777	806129	130728	M	3,065	3,065
2	9,954	23189073	3071524	M	88,158	88,158
3	10,362	2308859	419276	M	8,778	8,778
Total		26304061	3621529		100,000	100,000

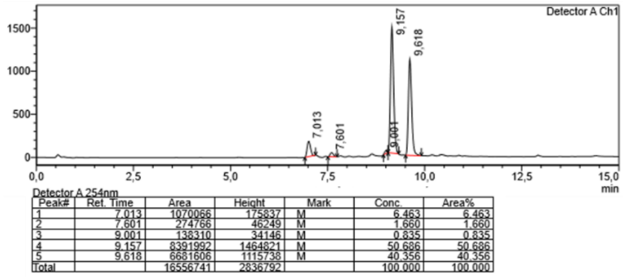

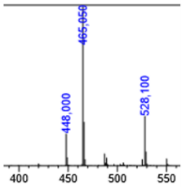

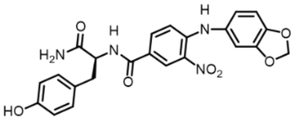

Exact Mass: 464.1332
Observed [M+H]+=465,050

Detector A 254nm

Peak#	Ret. Time	Area	Height	Mark	Conc.	Area%
1	7,013	1070066	175837	M	6,463	6,463
2	7,601	274766	46249	M	1,660	1,660
3	9,001	138310	34146	M	0,835	0,835
4	9,157	8391992	1464821	M	50,686	50,686
5	9,618	6681606	1115738	M	40,356	40,356
Total		16556741	2836792		100,000	100,000

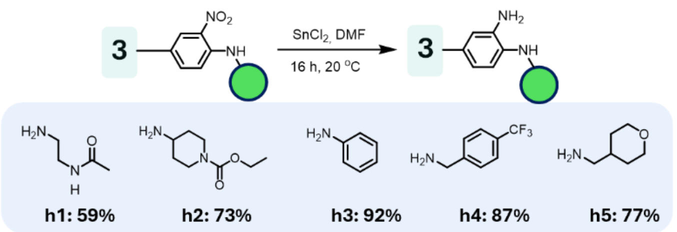

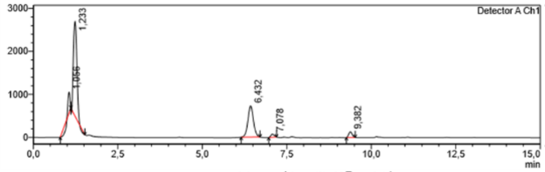

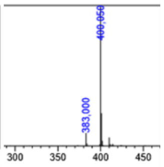

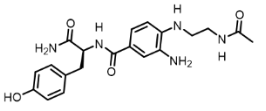

Molecular Weight: 399.4510

Observed [M+H]+=400,050

Detector A 275nm

Peak#	Ret. Time	Area	Height	Mark	Conc.	Area%
1	1,056	955263	483339	M	3,659	3,659
2	1,233	15486284	2196374	M	59,311	59,311
3	6,432	8256651	726834	M	31,622	31,622
4	7,078	500293	69400	M	1,916	1,916
5	9,382	911614	130778	M	3,491	3,491
Total		26110105	3606726		100,000	100,000

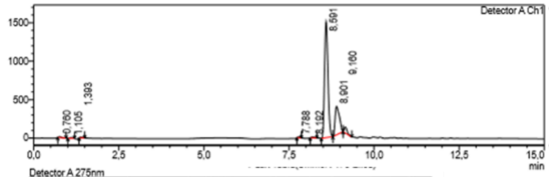

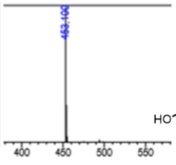

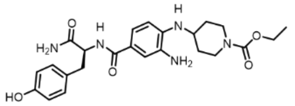

Molecular Weight: 469.5420

Observed [M-NH2]+=453,100

Detector A 275nm

Peak#	Ret. Time	Area	Height	Mark	Conc.	Area%
1	0,760	40511	10531	M	0,278	0,278
2	1,105	67824	10071	M	0,466	0,466
3	1,393	49369	8356	M	0,339	0,339
4	7,788	59573	12525	M	0,409	0,409
5	8,192	73702	11846	M	0,506	0,506
6	8,591	10606875	1506958	M	72,846	72,846
7	8,901	3249210	370167	M	22,315	22,315
8	9,160	413531	77666	M	2,840	2,840
Total		14560596	2008120		100,000	100,000

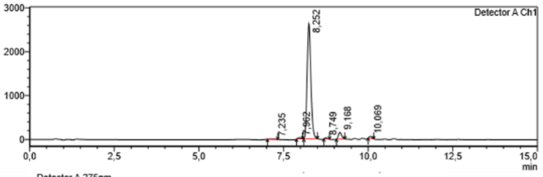

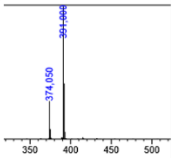

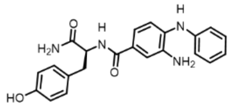

Molecular Weight: 390.4430

Observed [M+H]+=391,000

Detector A 275nm

Peak#	Ret. Time	Area	Height	Mark	Conc.	Area%
1	7,235	76252	7563	M	0,345	0,345
2	7,962	202868	32434	M	0,917	0,917
3	8,252	20410030	2634574	M	92,281	92,281
4	8,749	137003	25154	M	0,619	0,619
5	9,168	1041652	155792	M	4,710	4,710
6	10,069	249374	46156	M	1,128	1,128
Total		22117179	2901674		100,000	100,000

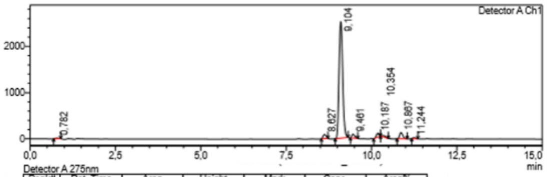

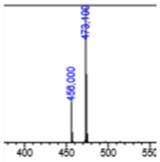

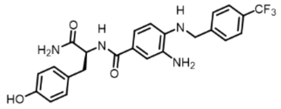

Molecular Weight: 472.4682

Observed [M+H]+=473,100

Detector A 275nm

Peak#	Ret. Time	Area	Height	Mark	Conc.	Area%
1	0,782	98220	12793	M	0,458	0,458
2	8,627	530624	87072	M	2,475	2,475
3	9,104	18715221	2517814	M	87,288	87,288
4	9,461	480323	81164	M	2,240	2,240
5	10,187	449499	82626	M	2,096	2,096
6	10,354	149967	27581	M	0,699	0,699
7	10,867	898356	137184	M	4,190	4,190
8	11,244	118618	21863	M	0,553	0,553
Total		21440829	2968096		100,000	100,000

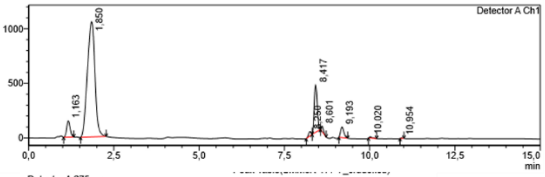

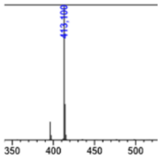

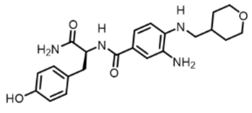

Molecular Weight: 412.4900

Observed [M+H]+= 413,100

Detector A 275nm

Peak#	Ret. Time	Area	Height	Mark	Conc.	Area%
1	1,163	1033844	147215	M	4,947	4,947
2	1,850	16068439	1054871	M	76,890	76,890
3	8,250	202202	39056	M	0,968	0,968
4	8,417	2633178	433474	M	12,600	12,600
5	8,601	216851	49054	M	1,038	1,038
6	9,193	640146	95175	M	3,063	3,063
7	10,020	75447	11727	M	0,361	0,361
8	10,954	27946	6598	M	0,134	0,134
Total		20898053	1837169		100,000	100,000

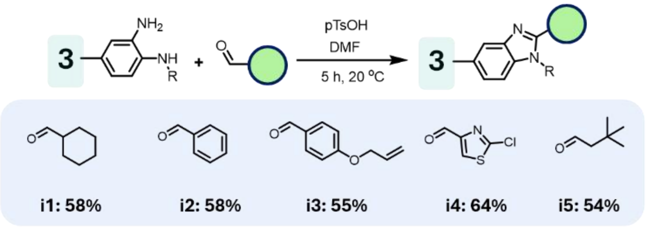

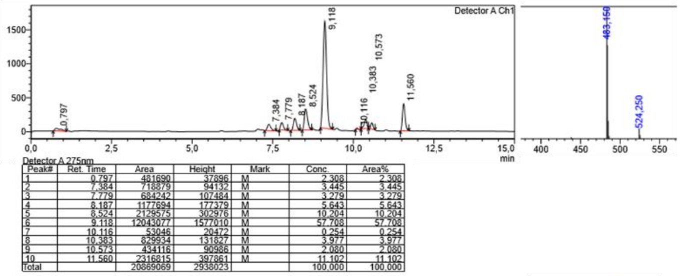

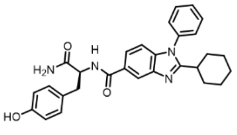

Molecular Weight: 482.5840
Observed [M+H]+=483,150

Detector A 275nm

Peak#	Ret. Time	Area	Height	Mark	Conc.	Area%
1	0.797	481690	37896	M	2.308	2.308
2	7.384	718879	94132	M	3.445	3.445
3	7.779	684242	107484	M	3.279	3.279
4	8.187	1177694	177379	M	5.643	5.643
5	8.524	2129575	302976	M	10.204	10.204
6	9.118	12043077	1577010	M	57.708	57.708
7	10.116	53046	20472	M	0.254	0.254
8	10.383	829934	131827	M	3.977	3.977
9	10.573	434116	90986	M	2.080	2.080
10	11.560	2316815	397861	M	11.102	11.102
Total		20869069	2938023		100.000	100.000

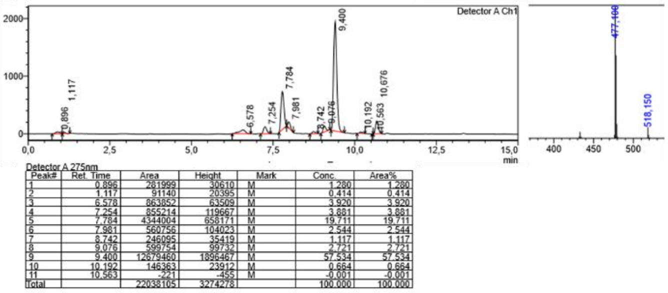

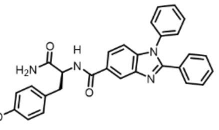

Molecular Weight: 476.5360
Observed [M+H]+=477,100

Detector A 275nm

Peak#	Ret. Time	Area	Height	Mark	Conc.	Area%
1	0.896	281999	30610	M	1.280	1.280
2	1.117	91140	20395	M	0.414	0.414
3	6.578	863852	63509	M	3.920	3.920
4	7.254	855214	119667	M	3.881	3.881
5	7.784	4344004	658171	M	19.711	19.711
6	7.981	560756	104023	M	2.544	2.544
7	8.742	246095	35419	M	1.117	1.117
8	9.076	599754	99732	M	2.721	2.721
9	9.400	12679460	1896467	M	57.534	57.534
10	10.192	146363	23912	M	0.664	0.664
11	10.563	-221	-455	M	-0.001	-0.001
Total		22038105	3274278		100.000	100.000

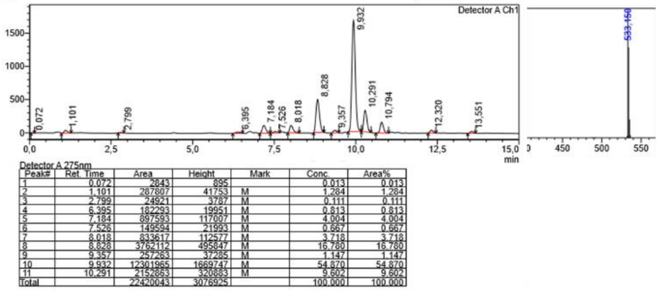

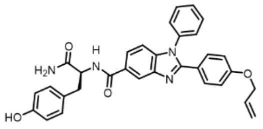

Molecular Weight: 532.6000
Observed [M+H]+=477,100

Detector A 275nm

Peak#	Ret. Time	Area	Height	Mark	Conc.	Area%
1	0.072	2843	895		0.013	0.013
2	1.101	287807	41753	M	1.284	1.284
3	2.799	24921	3787	M	0.111	0.111
4	6.395	182293	19951	M	0.813	0.813
5	7.184	897593	117007	M	4.004	4.004
6	7.526	149594	21993	M	0.667	0.667
7	8.018	833617	112577	M	3.718	3.718
8	8.828	3762112	495847	M	16.780	16.780
9	9.357	257263	37285	M	1.147	1.147
10	9.932	12301965	1669747	M	54.870	54.870
11	10.291	2152863	320883	M	9.602	9.602
Total		22420043	3076925		100.000	100.000

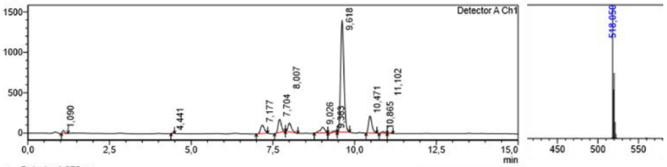

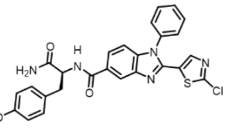

Molecular Weight: 517.9880
Observed [M+H]+=518,050

Detector A 275nm

Peak#	Ret. Time	Area	Height	Mark	Conc.	Area%
1	1.090	149653	34106	M	0.980	0.980
2	4.441	21133	5194	M	0.138	0.138
3	7.177	855191	104379	M	5.600	5.600
4	7.704	1109213	163174	M	7.264	7.264
5	8.007	837149	110196	M	5.482	5.482
6	9.026	698645	71272	M	4.575	4.575
7	9.363	106514	18476	M	0.698	0.698
8	9.618	9741807	1374278	M	63.796	63.796
9	10.471	1571527	214240	M	10.292	10.292
10	10.865	154188	21653	M	1.010	1.010
11	11.102	25106	5232	M	0.164	0.164
Total		15270128	2122199		100.000	100.000

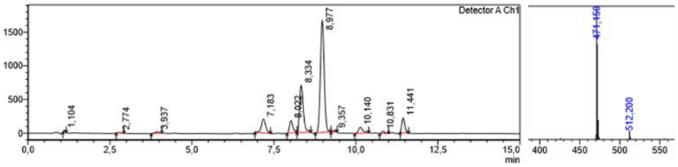

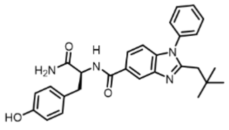

Molecular Weight: 470.5730
Observed [M+H]+= 471,150

Detector A 275nm

Peak#	Ret. Time	Area	Height	Mark	Conc.	Area%
1	1.104	82121	24083	M	0.326	0.326
2	2.774	62448	7873	M	0.248	0.248
3	3.937	186823	17732	M	0.742	0.742
4	7.183	1895568	202944	M	7.527	7.527
5	8.022	1314778	183098	M	5.220	5.220
6	8.334	5606424	698870	M	22.261	22.261
7	8.977	13501224	1658707	M	53.608	53.608
8	9.357	108662	22425	M	0.431	0.431
9	10.140	836500	88242	M	3.321	3.321
10	10.831	203271	30779	M	0.807	0.807
11	11.441	1387303	216954	M	5.508	5.508
Total		25185121	3151706		100.000	100.000

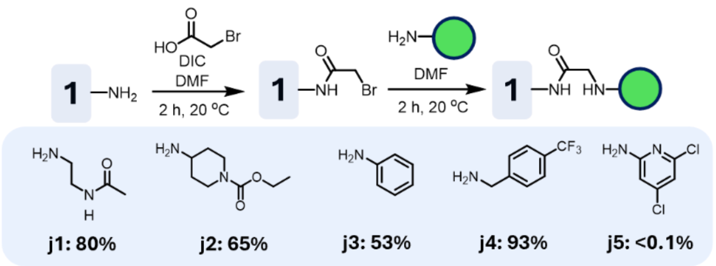

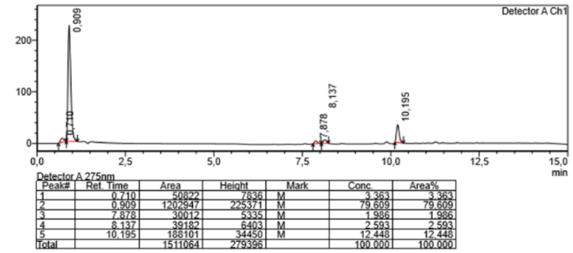

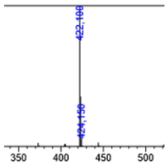

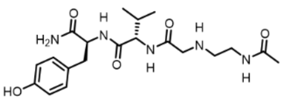

Exact Mass: 421.2325

Observed $[M+H]^+$=422,100

Detector A 275nm

Peak#	Ret. Time	Area	Height	Mark	Conc.	Area%
1	0.710	50822	7836	M	3.363	3.363
2	0.909	1202947	225371	M	79.609	79.609
3	7.878	30012	5335	M	1.986	1.986
4	8.137	39182	6403	M	2.593	2.593
5	10.195	188101	34450	M	12.448	12.448
Total		1511064	279396		100.000	100.000

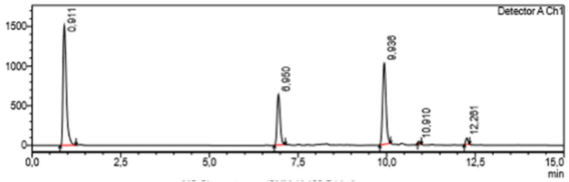

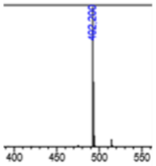

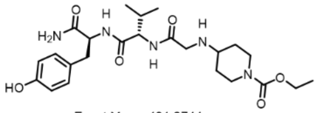

Exact Mass: 491.2744

Observed $[M+H]^+$=492,200

Detector A 275nm

Peak#	Ret. Time	Area	Height	Mark	Conc.	Area%
1	0.911	9504253	1514820	M	46.464	46.464
2	6.950	3824322	640435	M	18.696	18.696
3	9.936	6666462	1024896	M	32.590	32.590
4	10.910	90189	25554	M	0.441	0.441
5	12.261	370083	80745	M	1.809	1.809
Total		20455309	3286450		100.000	100.000

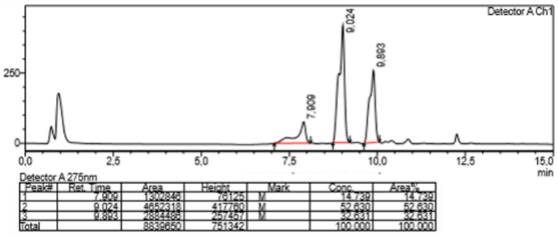

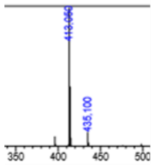

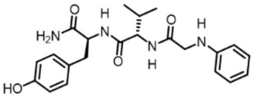

Exact Mass: 412.2111

Observed $[M+H]^+$=413,050

Detector A 275nm

Peak#	Ret. Time	Area	Height	Mark	Conc.	Area%
1	7.909	1302846	76125	M	14.739	14.739
2	9.024	4652318	417760	M	52.630	52.630
3	9.893	2884486	257457	M	32.631	32.631
Total		8839650	751342		100.000	100.000

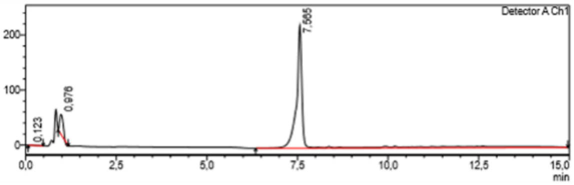

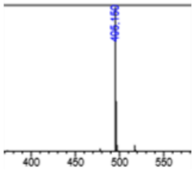

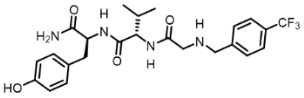

Exact Mass: 494.2141

Observed $[M+H]^+$=495,150

Detector A 275nm

Peak#	Ret. Time	Area	Height	Mark	Conc.	Area%
1	0.123	2729	232		0.087	0.087
2	0.976	218963	35828	M	6.986	6.986
3	7.565	2912479	222809		92.927	92.927
Total		3134170	258869		100.000	100.000

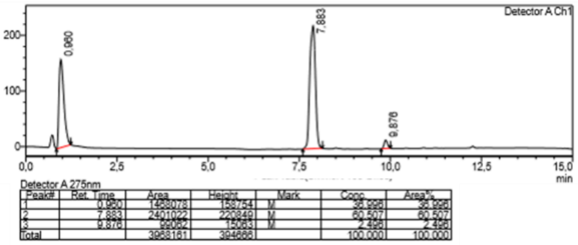

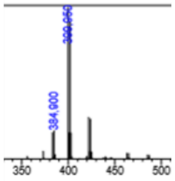

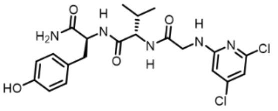

Exact Mass: 481.1284

Not Observed

Detector A 275nm

Peak#	Ret. Time	Area	Height	Mark	Conc.	Area%
1	0.960	1468078	158754	M	36.996	36.996
2	7.883	2401022	220849	M	60.507	60.507
3	9.876	99062	15063	M	2.496	2.496
Total		3968161	394666		100.000	100.000

HPLC-MS Data for compounds 1-132

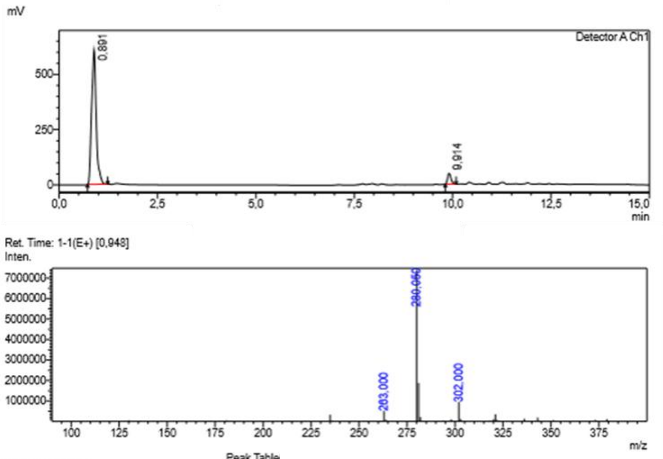

Peak Table

Detector A 275nm

Peak#	Ret. Time	Area	Height	Mark	Conc.	Area%
1	0,891	4897731	609188	M	94,320	94,320
2	9,914	294969	48912	M	5,680	5,680
Total		5192700	658100		100,000	100,000

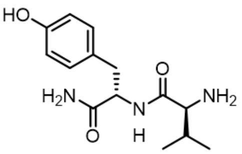

Exact Mass: 279.1583

Observed $[M+H]^{+}$=280,050

1

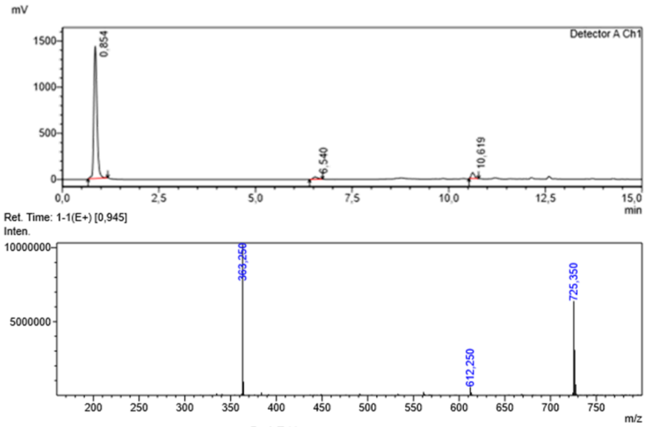

Peak Table

Detector A 275nm

Peak#	Ret. Time	Area	Height	Mark	Conc.	Area%
1	0,854	8067724	1430323	M	93,019	93,019
2	6,540	209902	24931	M	2,420	2,420
3	10,619	395587	64777	M	4,561	4,561
Total		8673212	1520031		100,000	100,000

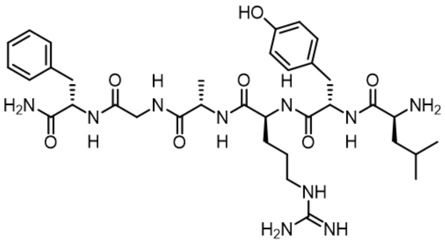

Exact Mass: 724.4020

Observed $[M+H]^{+}$=725,350

2

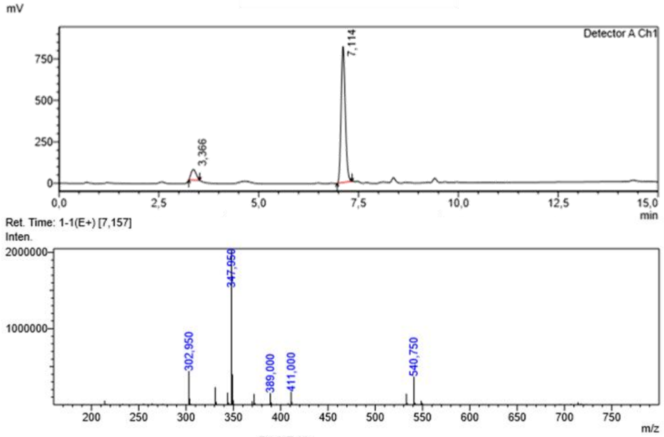

Peak Table

Detector A 254nm

Peak#	Ret. Time	Area	Height	Mark	Conc.	Area%
1	3,366	575838	64188	M	8,719	8,719
2	7,114	6028613	821994	M	91,281	91,281
Total		6604451	886182		100,000	100,000

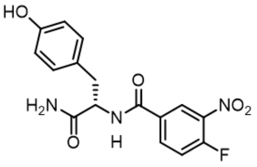

Exact Mass: 347.0917

Observed $[M+H]^{+}$=347,950

3

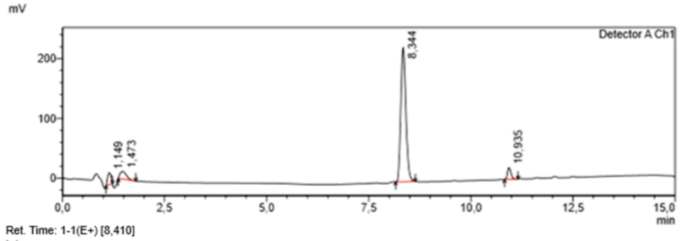

Ret. Time: 1-1(E+) [8,410]
Inten.

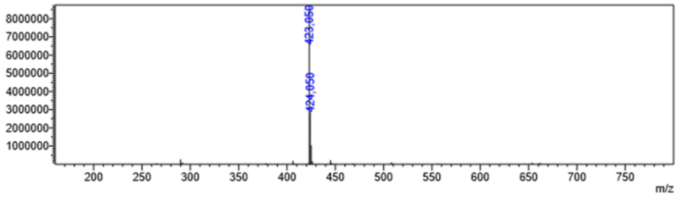

Peak Table

Detector A 254nm

Peak#	Ret. Time	Area	Height	Mark	Conc.	Area%
1	1,149	121290	20004	M	5,243	5,243
2	1,473	139130	12641	M	6,014	6,014
3	8,344	1935137	224921	M	83,643	83,643
4	10,935	118020	19844	M	5,101	5,101
Total		2313578	277410		100,000	100,000

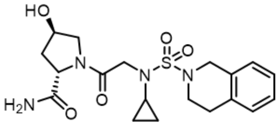

Exact Mass: 422.1624
Observed $[M+H]^+$=423,050
4

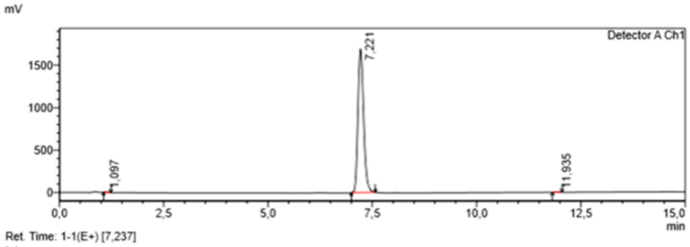

Ret. Time: 1-1(E+) [7,237]
Inten.

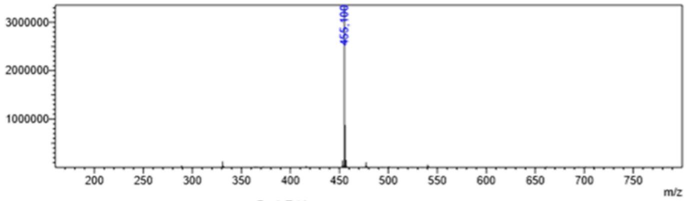

Peak Table

Detector A 254nm

Peak#	Ret. Time	Area	Height	Mark	Conc.	Area%
1	1,097	25106	6092	M	0,156	0,156
2	7,221	16068501	1693637	M	99,706	99,706
3	11,935	22205	3474	M	0,138	0,138
Total		16115812	1703203		100,000	100,000

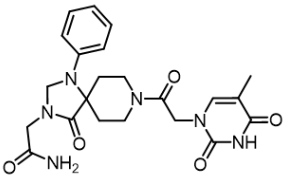

Exact Mass: 454.1965
Observed $[M+H]^+$=455,100

5

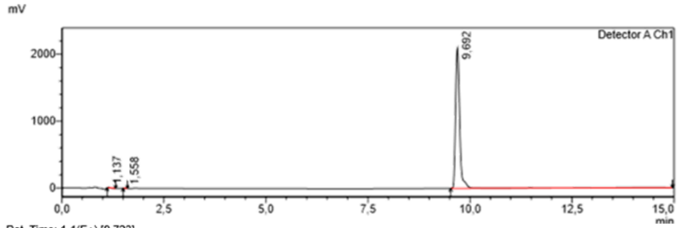

Ret. Time: 1-1(E+) [9,723]
Inten.

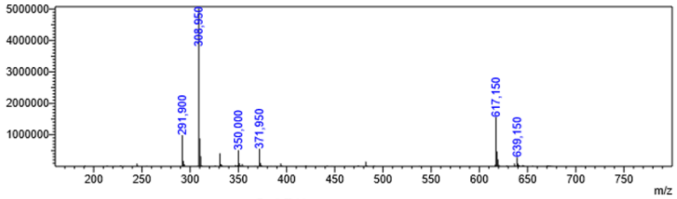

Peak Table

Detector A 254nm

Peak#	Ret. Time	Area	Height	Mark	Conc.	Area%
1	1,137	12836	6768	M	0,081	0,081
2	1,558	6834	1722	M	0,043	0,043
3	9,692	15814304	2099042		99,876	99,876
Total		15833974	2107532		100,000	100,000

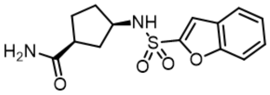

Exact Mass: 308.0831
Observed $[M+H]^+$=308,950
6

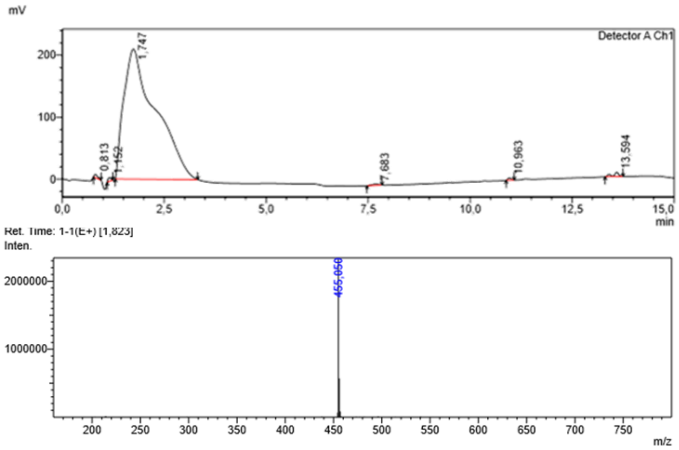

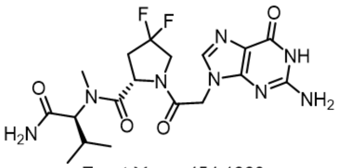

Exact Mass: 454.1889
Observed $[M+H]^+$=455,050

7

Peak Table

Detector A 254nm

Peak#	Ret. Time	Area	Height	Mark	Conc.	Area%
1	0,813	28607	5455	M	0,246	0,246
2	1,152	30894	6133	M	0,266	0,266
3	1,747	11435618	210196	M	98,518	98,518
4	7,683	24120	1828	M	0,208	0,208
5	10,963	21712	3682	M	0,187	0,187
6	13,594	66746	7057	M	0,575	0,575
Total		11607697	234351		100,000	100,000

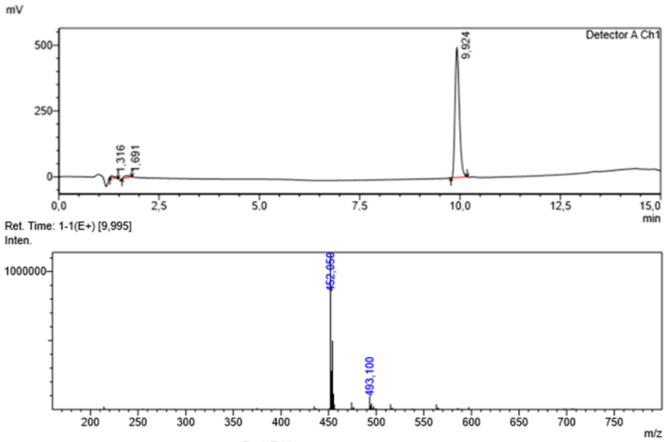

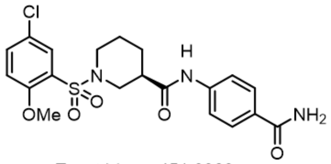

Exact Mass: 451.0969
Observed $[M+H]^+$=452,050

8

Peak Table

Detector A 254nm

Peak#	Ret. Time	Area	Height	Mark	Conc.	Area%
1	1,316	45418	5215	M	1,182	1,182
2	1,691	75283	8123	M	1,960	1,960
3	9,924	3721233	492474	M	96,858	96,858
Total		3841933	505812		100,000	100,000

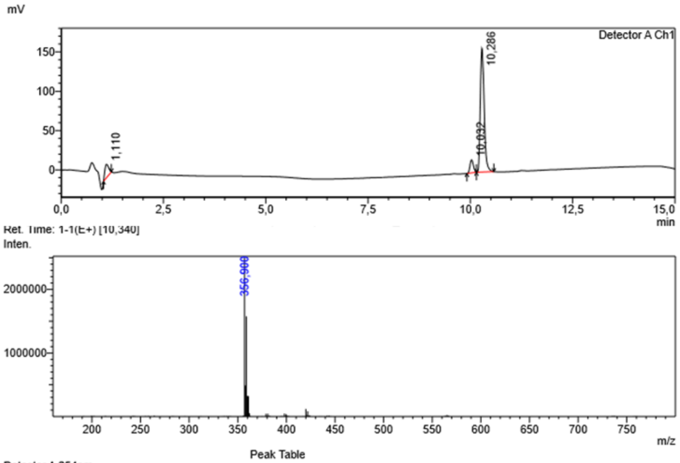

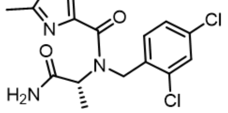

Exact Mass: 355.0490
Observed $[M+H]^+$=356,900

9

Peak Table

Detector A 254nm

Peak#	Ret. Time	Area	Height	Mark	Conc.	Area%
1	1,110	113947	16888	M	9,322	9,322
2	10,032	96305	16537	M	7,879	7,879
3	10,286	1012077	157457	M	82,799	82,799
Total		1222328	190882		100,000	100,000

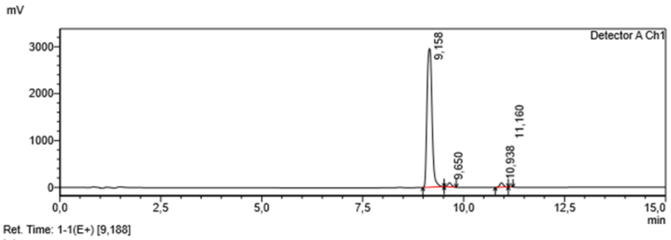

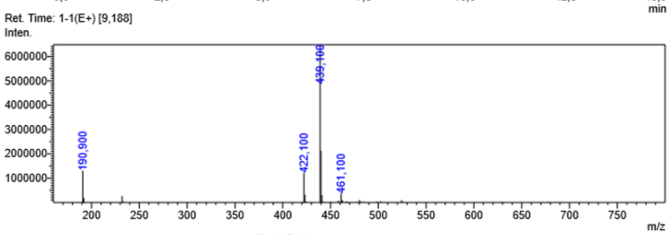

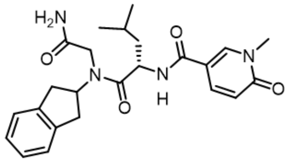

Exact Mass: 438.2267

Observed $[M+H]^{+}$= 439,100

10

Peak Table

Detector A 254nm

Peak#	Ret. Time	Area	Height	Mark	Conc.	Area%
1	9.158	25580461	2958361	M	95.658	95.658
2	9.650	562599	84092	M	2.104	2.104
3	10.938	556224	90988	M	2.080	2.080
4	11.160	42158	10055	M	0.158	0.158
Total		26741442	3143495		100.000	100.000

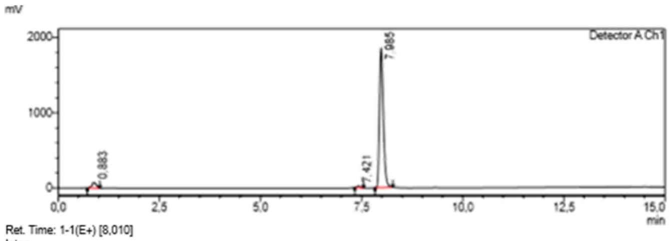

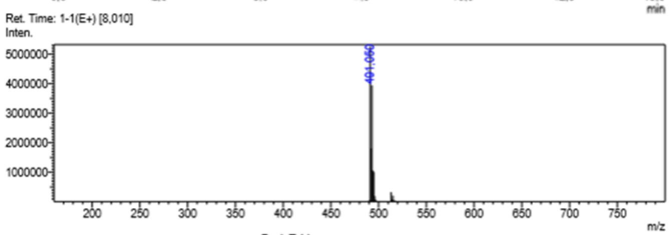

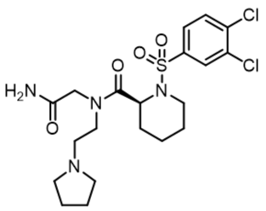

Exact Mass: 490.1208

Observed $[M+H]^{+}$=491,050

11

Peak Table

Detector A 254nm

Peak#	Ret. Time	Area	Height	Mark	Conc.	Area%
1	0.883	548276	76287	M	4.075	4.075
2	7.421	127649	23077	M	0.949	0.949
3	7.985	12780274	1848023	M	94.977	94.977
Total		13456200	1947388		100.000	100.000

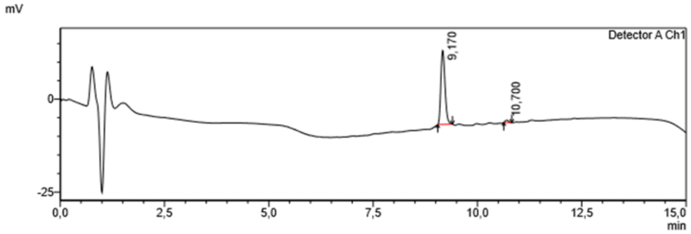

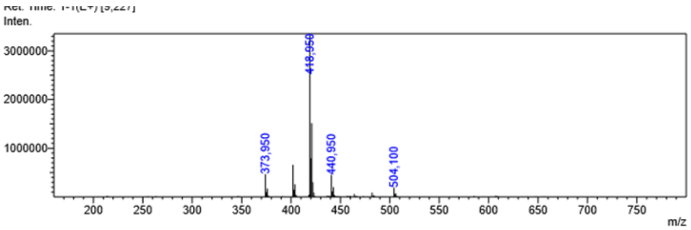

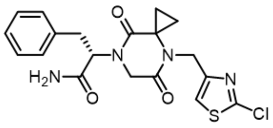

Exact Mass: 418.0866

Observed $[M+H]^{+}$= 418,950

12

Peak Table

Detector A 275nm

Peak#	Ret. Time	Area	Height	Mark	Conc.	Area%
1	9.170	137304	19899	M	97.617	97.617
2	10.700	3351	599	M	2.383	2.383
Total		140655	20498		100.000	100.000

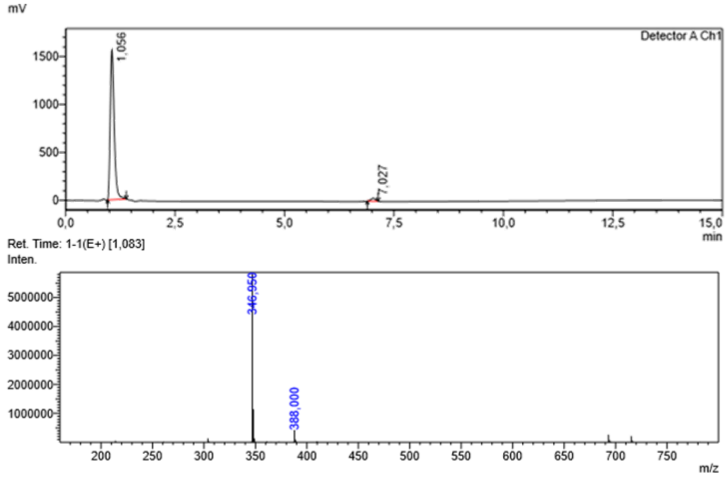

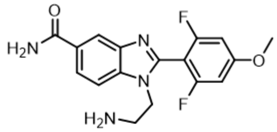

Exact Mass: 346.1241
Observed $[M+H]^+$= 346,950

13

Peak Table

Detector A 254nm

Peak#	Ret. Time	Area	Height	Mark	Conc.	Area%
1	1,056	10017243	1561561	M	97,881	97,881
2	7,027	216891	30016	M	2,119	2,119
Total		10234134	1591577		100,000	100,000

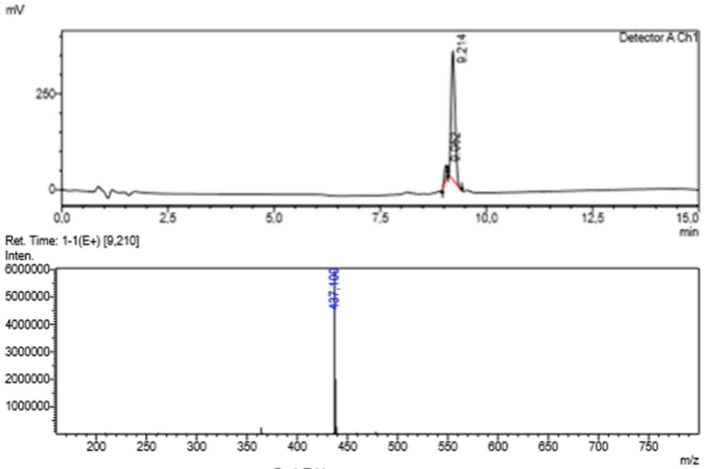

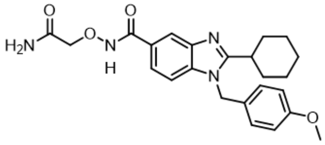

Exact Mass: 436.2111
Observed $[M+H]^+$=437,100

14

Peak Table

Detector A 254nm

Peak#	Ret. Time	Area	Height	Mark	Conc.	Area%
1	9,052	183950	37804	M	7,872	7,872
2	9,214	2152695	335140	M	92,128	92,128
Total		2336645	372944		100,000	100,000

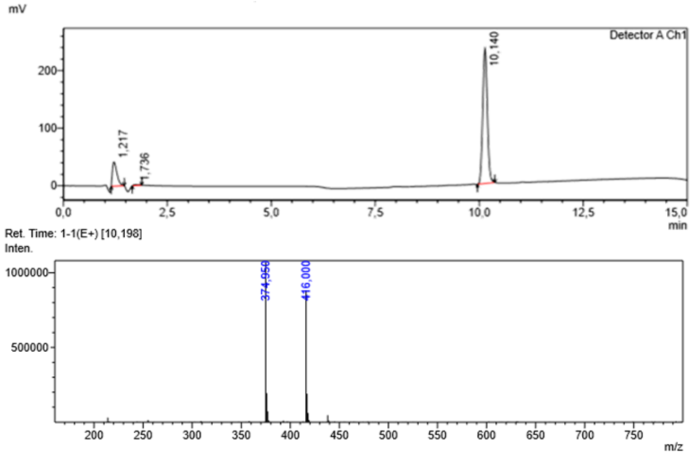

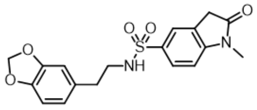

Exact Mass: 374.0936
Observed $[M+H]^+$=375,950

15

Peak Table

Detector A 254nm

Peak#	Ret. Time	Area	Height	Mark	Conc.	Area%
1	1,217	325518	42225	M	15,226	15,226
2	1,736	12635	1487	M	0,591	0,591
3	10,140	1799700	234242	M	84,183	84,183
Total		2137853	277955		100,000	100,000

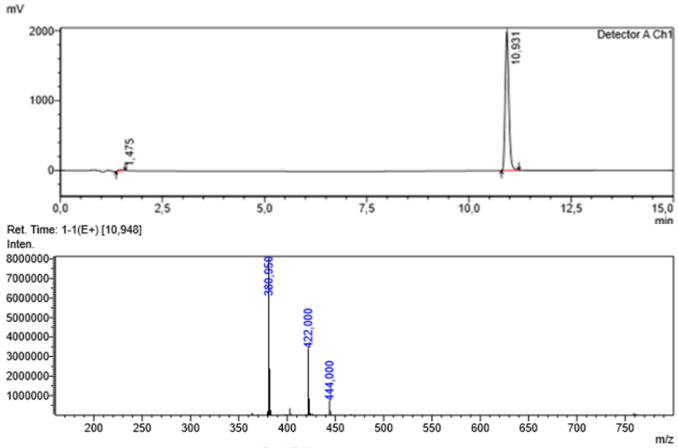

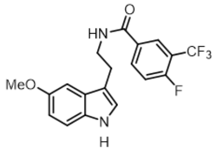

Exact Mass: 380.1148
Observed $[M+H]^+$=380,950

16

Peak Table

Detector A 254nm

Peak#	Ret. Time	Area	Height	Mark	Conc.	Area%
1	1,475	121290	14612	M	0,966	0,966
2	10,931	12430634	1998787	M	99,034	99,034
Total		12551923	2013398		100,000	100,000

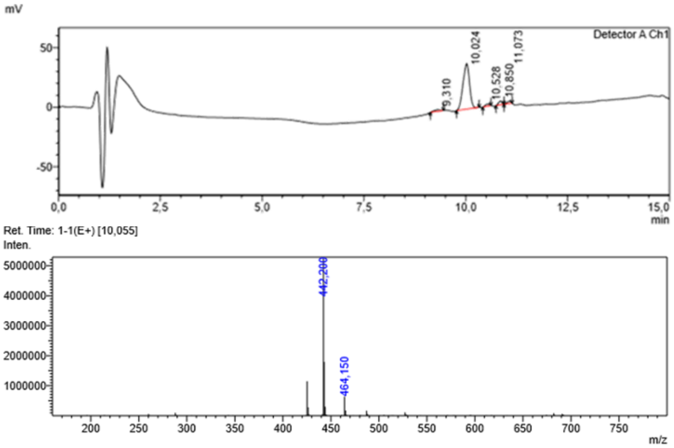

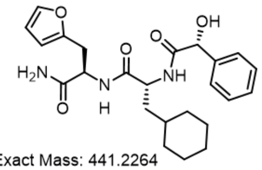

Exact Mass: 441.2264
Observed $[M+H]^+$=442,200

17

Peak Table

Detector A 254nm

Peak#	Ret. Time	Area	Height	Mark	Conc.	Area%
1	9,310	15025	1405	M	3,077	3,077
2	10,024	428183	38346	M	87,688	87,688
3	10,528	12547	1827	M	2,570	2,570
4	10,850	20340	3153	M	4,166	4,166
5	11,073	12205	2051	M	2,500	2,500
Total		488301	46783		100,000	100,000

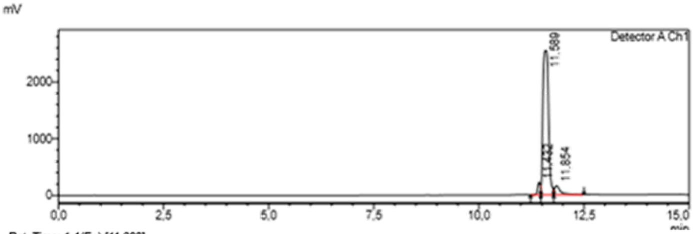

Ret. Time: 1-1(E+) [11,600]
Inten.

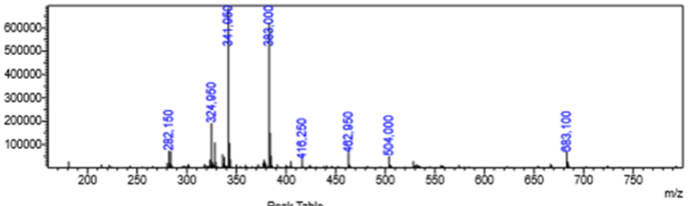

Peak Table

Detector A 254nm

Peak#	Ret. Time	Area	Height	Mark	Conc.	Area%
1	11,432	1081499	223151		4,076	4,076
2	11,589	23628434	2548816	V	89,054	89,054
3	11,854	1822622	165376	V	6,869	6,869
Total		26532554	2937343		100,000	100,000

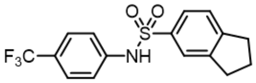

Exact Mass: 341.0697
Observed $[M+H]^+$=341,950

18

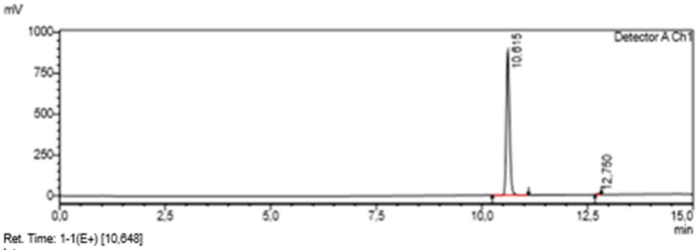

Ret. Time: 1-1(E+) [10,648]
Inten.

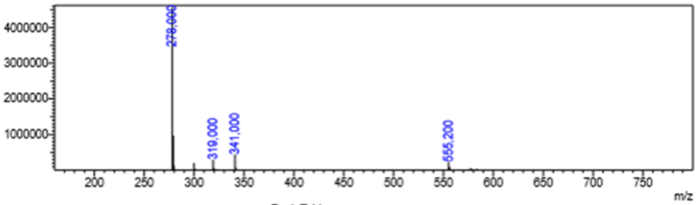

Peak Table

Detector A 254nm

Peak#	Ret. Time	Area	Height	Mark	Conc.	Area%
1	10,615	4796547	884878		99,642	99,642
2	12,750	17217	3640	M	0,358	0,358
Total		4813764	888518		100,000	100,000

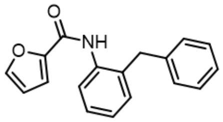

Molecular Weight: 277.3230
Observed $[M+H]^+$=278,000

19

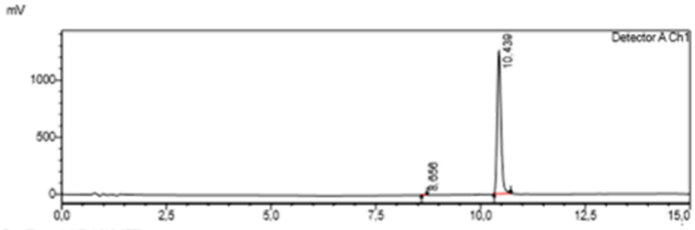

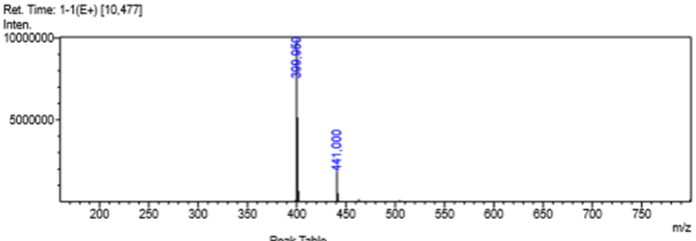

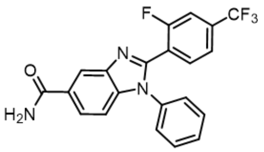

Exact Mass: 399.0995

Observed $[M+H]^+$=399,950

20

Peak Table

Detector A 254nm

Peak#	Ret. Time	Area	Height	Mark	Conc.	Area%
1	8.656	17437	3604	M	0.213	0.213
2	10.439	8159012	1250151	M	99.787	99.787
Total		8176449	1253754		100.000	100.000

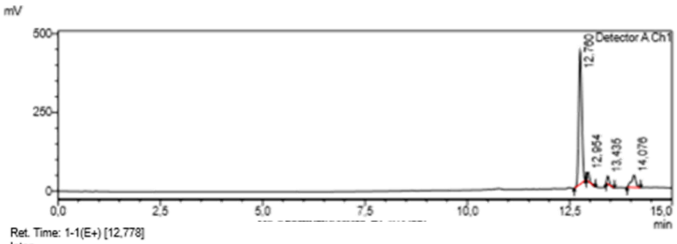

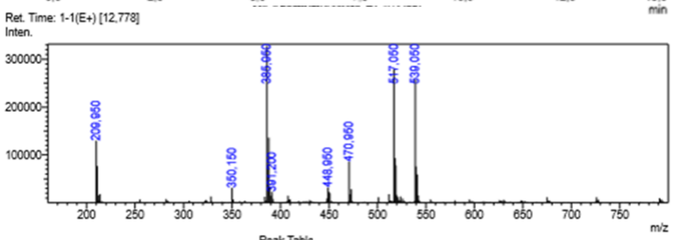

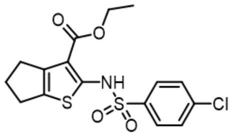

Exact Mass: 385.0209

Observed $[M+H]^+$=385,950

21

Peak Table

Detector A 254nm

Peak#	Ret. Time	Area	Height	Mark	Conc.	Area%
1	12.760	2231746	423179	M	82.496	82.496
2	12.954	107676	29305	M	3.980	3.980
3	13.435	84598	25942	M	3.127	3.127
4	14.076	281269	39190	M	10.397	10.397
Total		2705289	517616		100.000	100.000

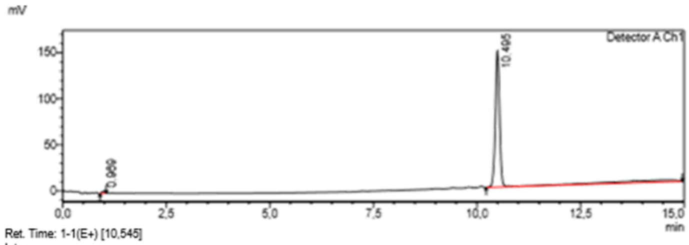

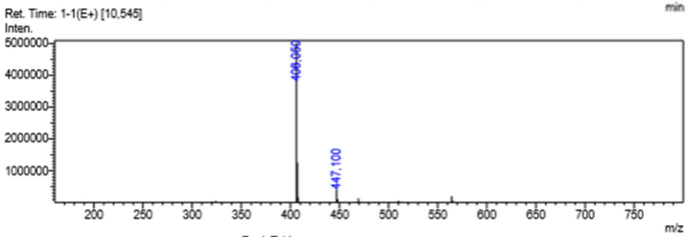

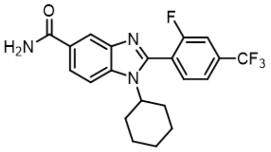

Exact Mass: 405.1464

Observed $[M+H]^+$=406,050

22

Peak Table

Detector A 254nm

Peak#	Ret. Time	Area	Height	Mark	Conc.	Area%
1	0.969	12370	2589	M	0.917	0.917
2	10.495	1337101	148185		99.083	99.083
Total		1349471	150775		100.000	100.000

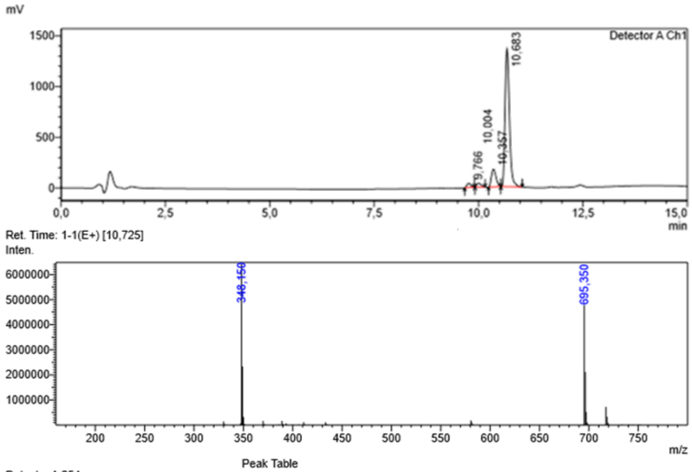

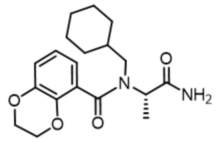

Exact Mass: 346.1893
Obserevd $[M+H]^+$=347,150

23

Peak Table

Detector A 254nm

Peak#	Ret. Time	Area	Height	Mark	Conc.	Area%
1	9,766	283004	42048	M	2,392	2,392
2	10,004	254792	36308	M	2,153	2,153
3	10,357	1321345	176460	M	11,167	11,167
4	10,683	9973645	1357688	M	84,288	84,288
Total		11832786	1612505		100,000	100,000

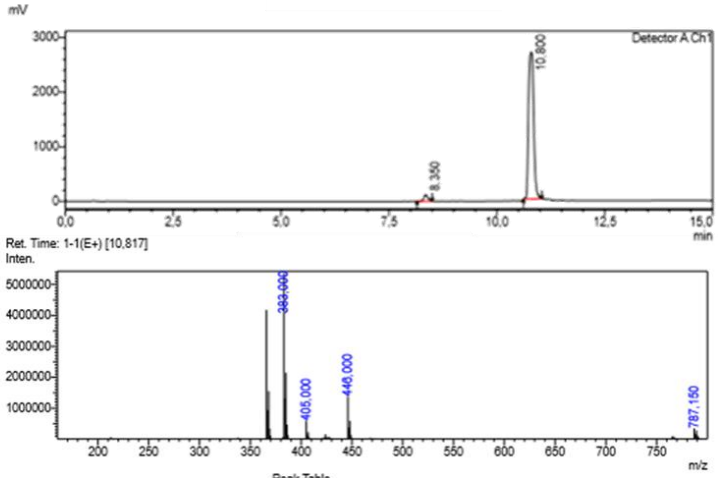

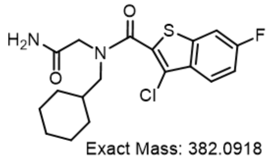

Exact Mass: 382.0918
Observed $[M+H]^+$=383,000

24

Peak Table

Detector A 254nm

Peak#	Ret. Time	Area	Height	Mark	Conc.	Area%
1	8,350	738701	121559	M	3,132	3,132
2	10,800	22848585	2690948	M	96,868	96,868
Total		23587286	2812507		100,000	100,000

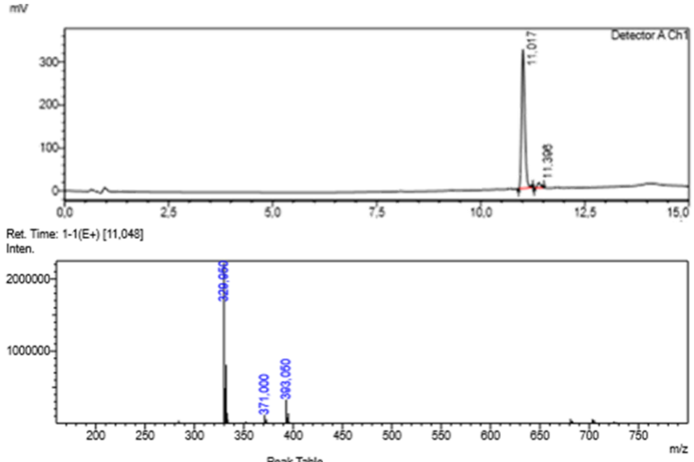

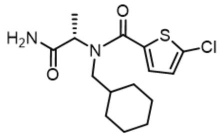

Exact Mass: 328.1012
Observed $[M+H]^+$: 329,950

25

Peak Table

Detector A 254nm

Peak#	Ret. Time	Area	Height	Mark	Conc.	Area%
1	11,017	1791816	324891	M	96,435	96,435
2	11,396	66231	12413	M	3,565	3,565
Total		1858048	337304		100,000	100,000

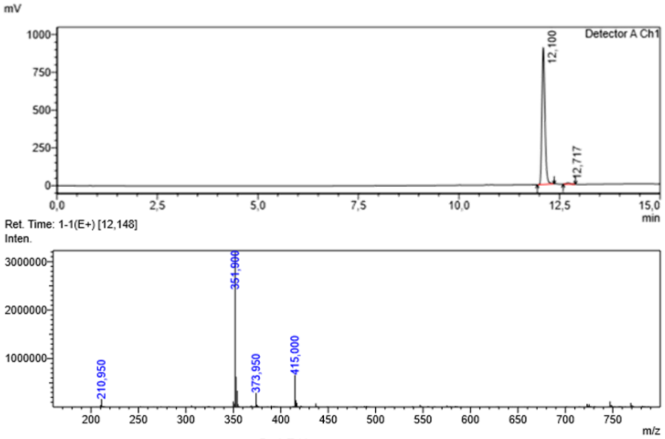

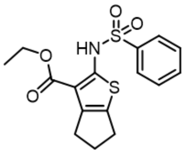

Exact Mass: 351.0599
Observed $[M+H]^+$=351,900

26

Peak Table

Detector A 254nm

Peak#	Ret. Time	Area	Height	Mark	Conc.	Area%
1	12,100	4703647	910393	M	98,639	98,639
2	12,717	64918	8397	M	1,361	1,361
Total		4768564	918791		100,000	100,000

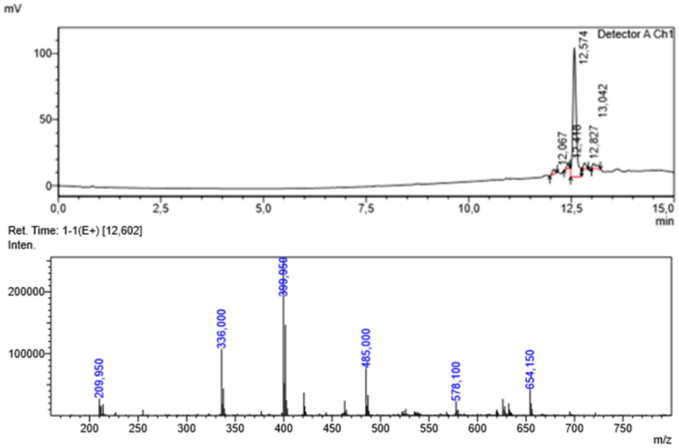

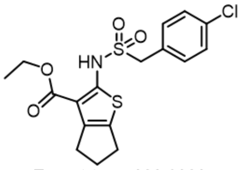

Exact Mass: 399.0366
Observed $[M+H]^+$=399,950

27

Peak Table

Detector A 254nm

Peak#	Ret. Time	Area	Height	Mark	Conc.	Area%
1	12,067	16411	3643	M	2,581	2,581
2	12,418	17244	4586	M	2,712	2,712
3	12,574	550571	97996		86,602	86,602
4	12,827	21604	4397	M	3,398	3,398
5	13,042	29916	3770	M	4,706	4,706
Total		635747	114392		100,000	100,000

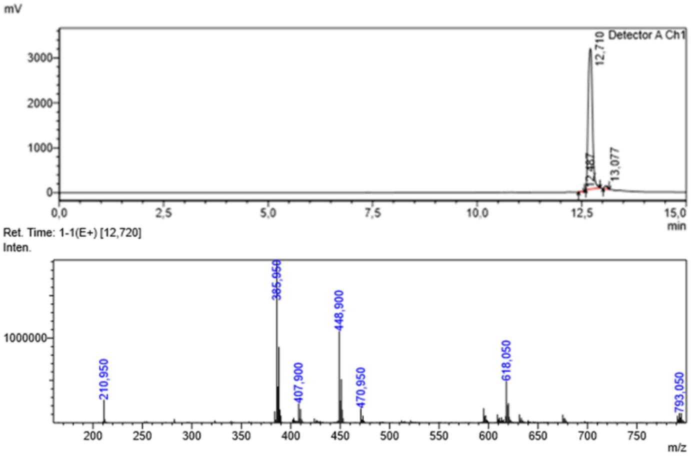

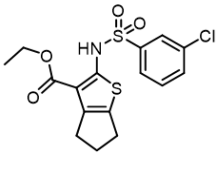

Exact Mass: 385.0209
Observed $[M+H]^+$=385,950

28

Peak Table

Detector A 254nm

Peak#	Ret. Time	Area	Height	Mark	Conc.	Area%
1	12,487	44418	10673	M	0,197	0,197
2	12,710	22224079	3126125	M	98,476	98,476
3	13,077	299521	68470	M	1,327	1,327
Total		22568018	3205268		100,000	100,000

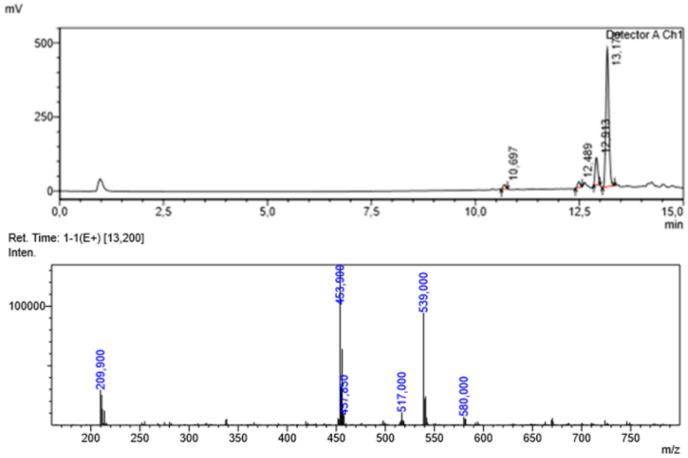

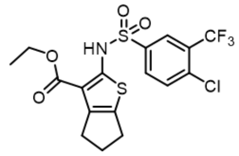

Exact Mass: 453.0083
Observed $[M+H]^+$=453,900

29

Peak Table

Detector A 254nm

Peak#	Ret. Time	Area	Height	Mark	Conc.	Area%
1	10,697	67829	14489	M	2,329	2,329
2	12,489	91198	20002	M	3,131	3,131
3	12,913	422635	92795	M	14,512	14,512
4	13,173	2330627	467821	M	80,027	80,027
Total		2912288	595107		100,000	100,000

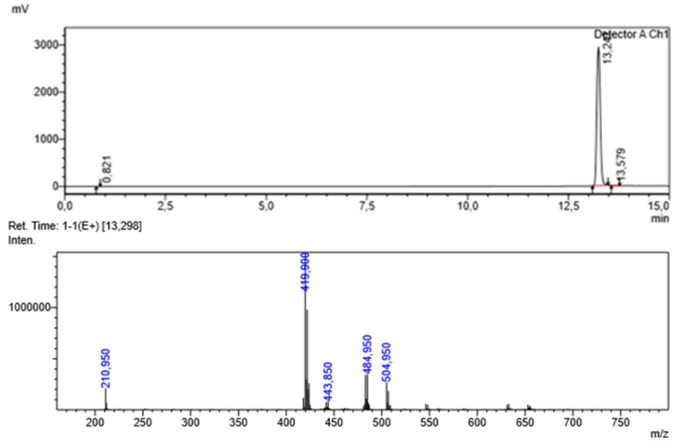

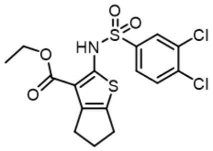

Exact Mass: 418.9820
Observed $[M+H]^+$=419,900

30

Peak Table

Detector A 254nm

Peak#	Ret. Time	Area	Height	Mark	Conc.	Area%
1	0,821	6224	1946	M	0,035	0,035
2	13,247	17790463	2932805	M	99,957	99,957
3	13,579	1487	-145	M	0,008	0,008
Total		17798174	2934606		100,000	100,000

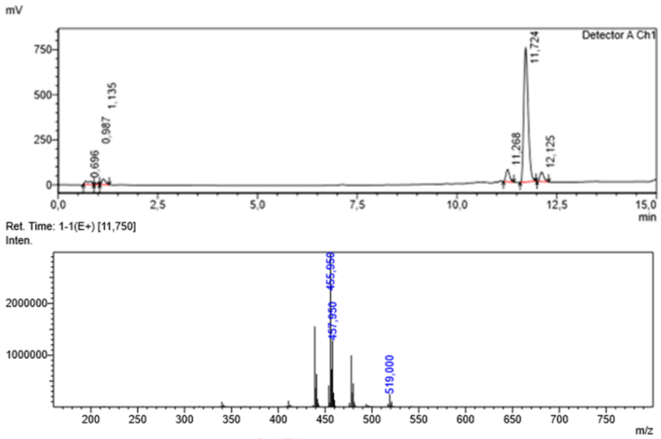

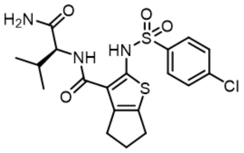

Exact Mass: 455.0740
Observed $[M+H]^+$=455,950

31

Peak Table

Detector A 254nm

Peak#	Ret. Time	Area	Height	Mark	Conc.	Area%
1	0,696	206348	17758	M	2,996	2,996
2	0,987	36219	8401	M	0,526	0,526
3	1,135	193985	31288	M	2,816	2,816
4	11,268	416731	69163	M	6,050	6,050
5	11,724	5681446	743642	M	82,484	82,484
6	12,125	353231	53268	M	5,128	5,128
Total		6887960	923520		100,000	100,000

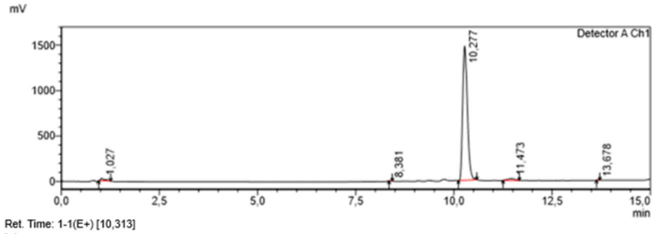

Ret. Time: 1-1(E+) [10,313]

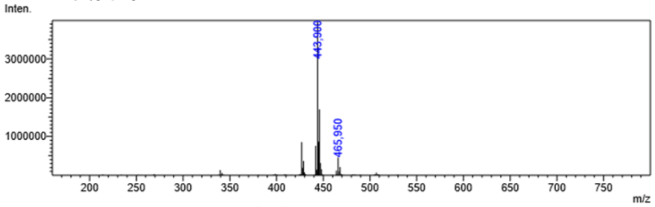

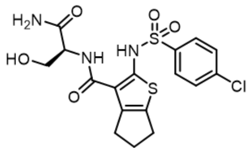

Exact Mass: 443.0376
Observed $[M+H]^+$=443,900

32

Peak Table

Detector A 254nm

Peak#	Ret. Time	Area	Height	Mark	Conc.	Area%
1	1,027	229153	26353	M	1,816	1,816
2	8,381	3849	1235	M	0,030	0,030
3	10,277	12143549	1477973	M	96,230	96,230
4	11,473	239148	18799	M	1,895	1,895
5	13,678	3614	1094	M	0,029	0,029
Total		12619313	1525454		100,000	100,000

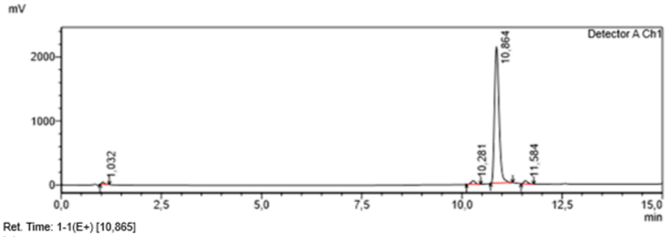

Ret. Time: 1-1(E+) [10,865]

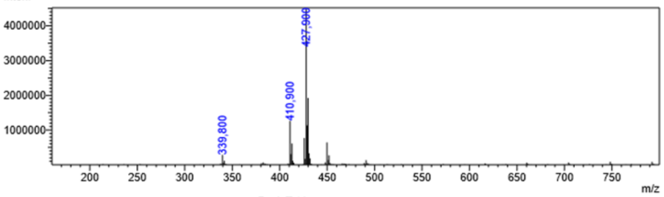

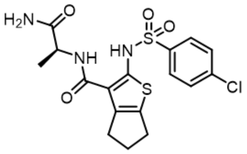

Exact Mass: 427.0427
Observed $[M+H]^+$=427,900

33

Peak Table

Detector A 254nm

Peak#	Ret. Time	Area	Height	Mark	Conc.	Area%
1	1,032	171744	35097	M	0,973	0,973
2	10,281	375862	55456	M	2,129	2,129
3	10,864	16799961	2127686	M	95,143	95,143
4	11,584	310051	47933	M	1,756	1,756
Total		17657617	2266172		100,000	100,000

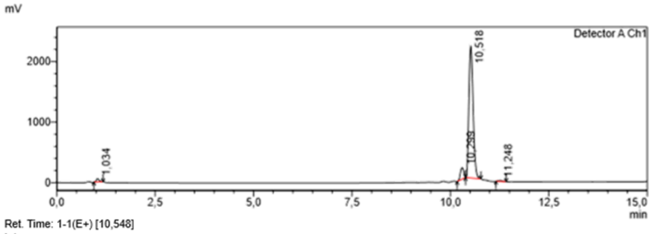

Ret. Time: 1-1(E+) [10,548]

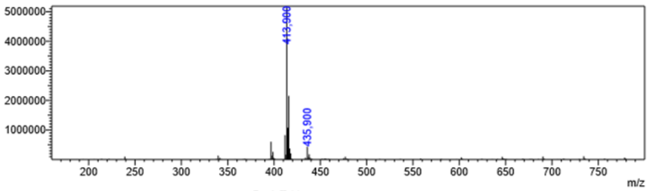

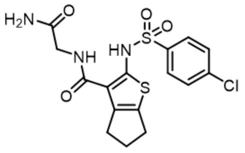

Exact Mass: 413.0271
Observed $[M+H]^+$=413,900

34

Peak Table

Detector A 254nm

Peak#	Ret. Time	Area	Height	Mark	Conc.	Area%
1	1,034	295333	54918	M	1,652	1,652
2	10,299	1098521	190115	M	6,146	6,146
3	10,518	16350179	2175254	M	91,469	91,469
4	11,248	131143	15975	M	0,734	0,734
Total		17875176	2436262		100,000	100,000

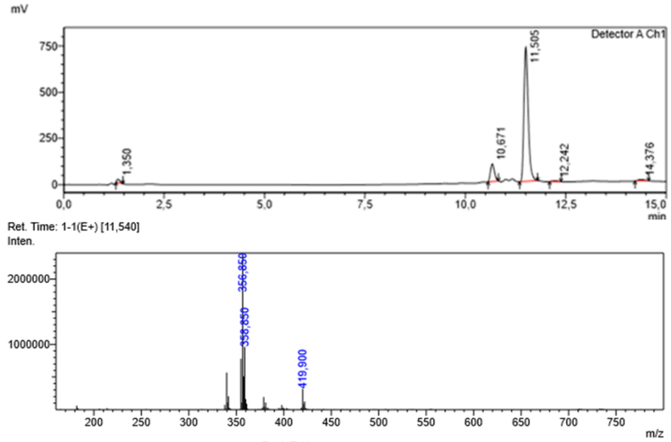

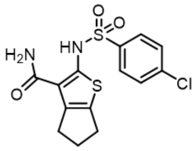

Exact Mass: 356.0056
Observed $[M+H]^+$=356,850

35

Peak Table

Detector A 254nm

Peak#	Ret. Time	Area	Height	Mark	Conc.	Area%
1	1,350	100565	19033	M	1,588	1,588
2	10,671	669109	95918	M	10,565	10,565
3	11,505	5435909	726596	M	85,833	85,833
4	12,242	39659	4231	M	0,626	0,626
5	14,376	87917	7613	M	1,388	1,388
Total		6333159	853390		100,000	100,000

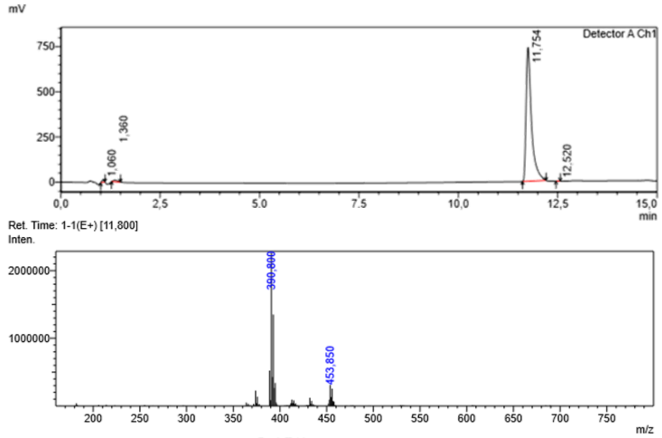

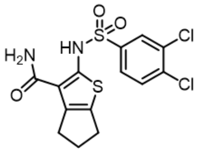

Exact Mass: 389.9666
Observed $[M+H]^+$=390,800

36

Peak Table

Detector A 254nm

Peak#	Ret. Time	Area	Height	Mark	Conc.	Area%
1	1,060	51265	11842	M	0,713	0,713
2	1,360	63768	7466	M	0,887	0,887
3	11,754	7073606	740715	M	98,386	98,386
4	12,520	992	256	M	0,014	0,014
Total		7189630	760278		100,000	100,000

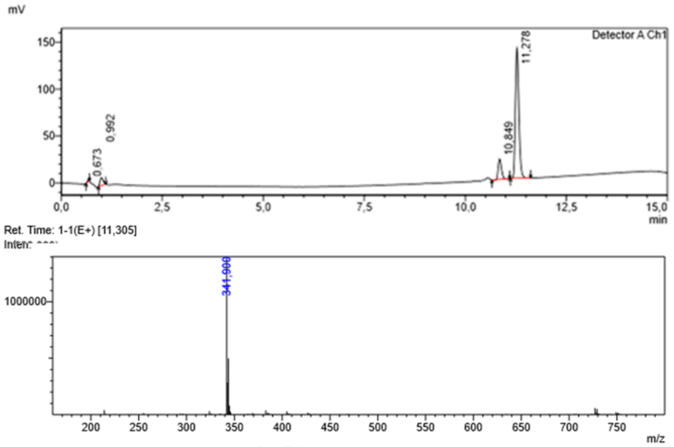

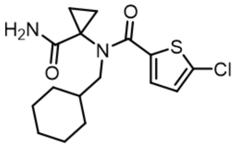

Exact Mass: 340.1012
Observed $[M+H]^+$=341,900

37

Peak Table

Detector A 254nm

Peak#	Ret. Time	Area	Height	Mark	Conc.	Area%
1	0,673	6385	1782	M	0,653	0,653
2	0,992	46351	8597	M	4,738	4,738
3	10,849	116429	21749	M	11,901	11,901
4	11,278	809157	138677	M	82,709	82,709
Total		978321	170804		100,000	100,000

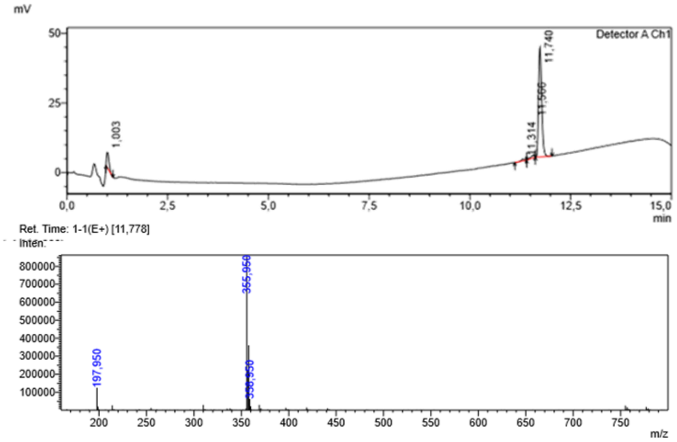

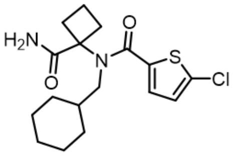

Exact Mass: 354.1169
Observed $[M+H]^+$=355,950

38

Peak Table

Detector A 254nm

Peak#	Ret. Time	Area	Height	Mark	Conc.	Area%
1	1,003	22146	5704	M	8,972	8,972
2	11,314	2865	625	M	1,161	1,161
3	11,566	5924	951	M	2,400	2,400
4	11,740	215904	39326	M	87,467	87,467
Total		246840	46606		100,000	100,000

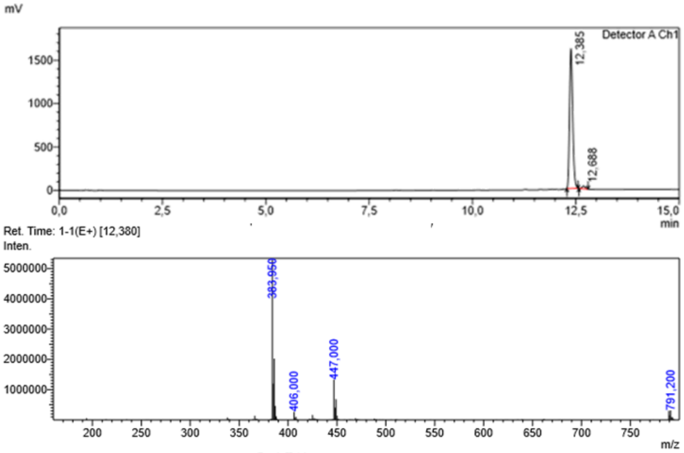

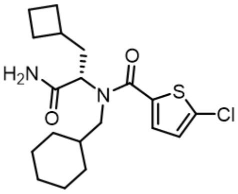

Exact Mass: 382.1482
Observed $[M+H]^+$=383,950

39

Peak Table

Detector A 254nm

Peak#	Ret. Time	Area	Height	Mark	Conc.	Area%
1	12,385	8660659	1618406	M	98,198	98,198
2	12,688	158954	31764	M	1,802	1,802
Total		8819614	1650169		100,000	100,000

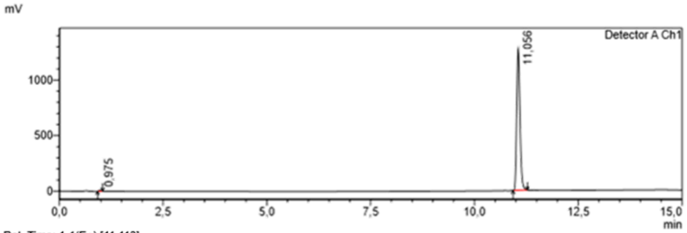

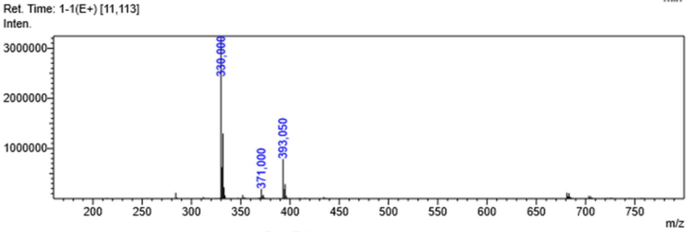

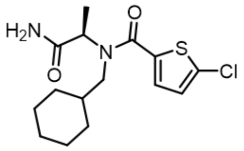

Exact Mass: 328.1012
Observed $[M+H]^+$=330,000

40

Peak Table

Detector A 254nm

Peak#	Ret. Time	Area	Height	Mark	Conc.	Area%
1	0,975	27332	6572	M	0,387	0,387
2	11,056	7042893	1279357	M	99,613	99,613
Total		7070226	1285929		100,000	100,000

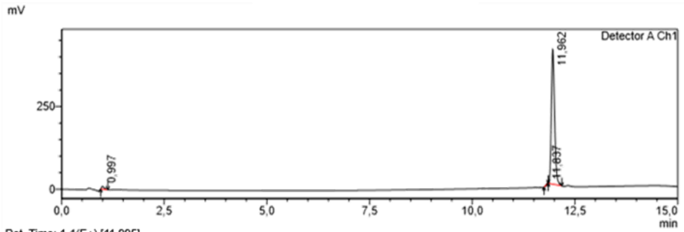

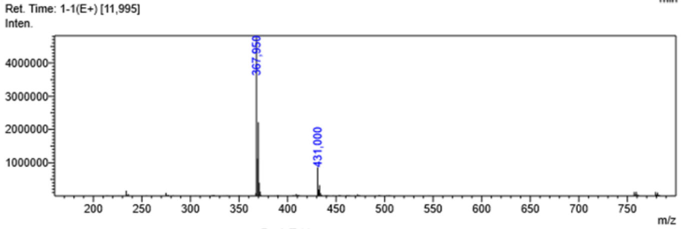

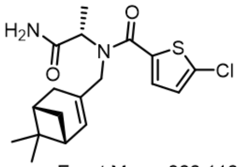

Exact Mass: 366.1169
Observed $[M+H]^+$=367,950

41

Peak Table

Detector A 254nm

Peak#	Ret. Time	Area	Height	Mark	Conc.	Area%
1	0,997	39778	8296	M	1,706	1,706
2	11,837	10158	1828	M	0,436	0,436
3	11,962	2281603	408005	M	97,858	97,858
Total		2331539	418129		100,000	100,000

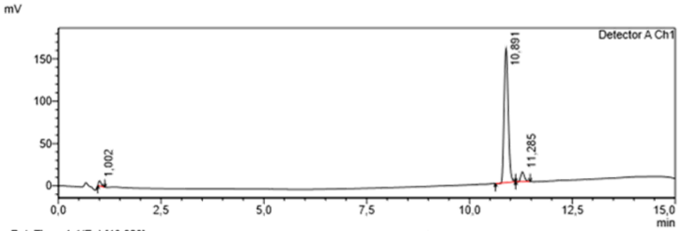

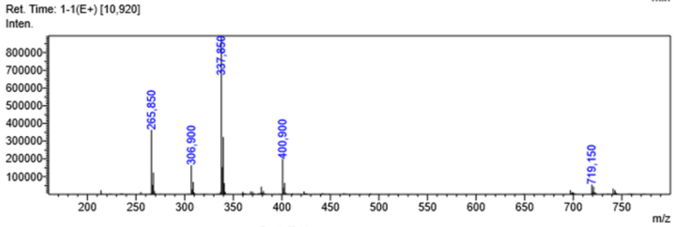

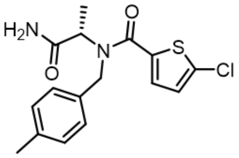

Exact Mass: 336.0699
Observed $[M+H]^+$=337,850

42

Peak Table

Detector A 254nm

Peak#	Ret. Time	Area	Height	Mark	Conc.	Area%
1	1,002	27999	6175	M	2,328	2,328
2	10,891	1104695	159001	M	91,870	91,870
3	11,285	69761	11666	M	5,802	5,802
Total		1202455	176841		100,000	100,000

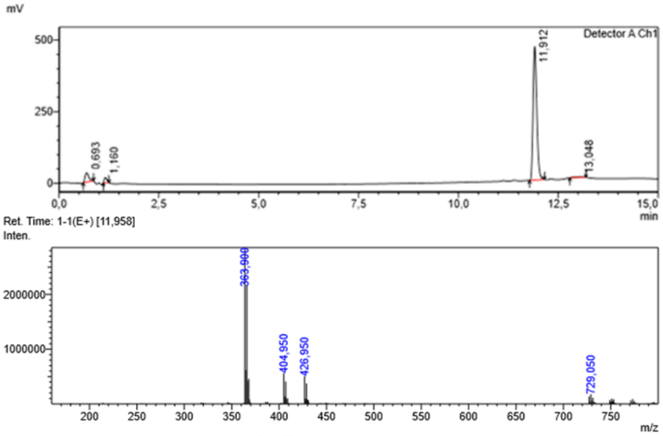

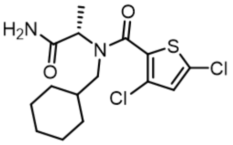

Exact Mass: 362.0623
Observed $[M+H]^+$=363,900
43

Peak Table

Detector A 254nm

Peak#	Ret. Time	Area	Height	Mark	Conc.	Area%
1	0,693	219437	32289	M	6,501	6,501
2	1,160	85505	18426	M	2,533	2,533
3	11,912	3029253	466184	M	89,743	89,743
4	13,048	41297	2527	M	1,223	1,223
Total		3375492	519425		100,000	100,000

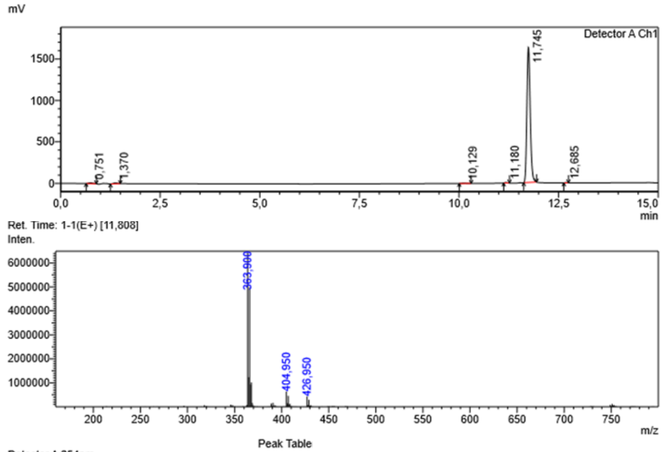

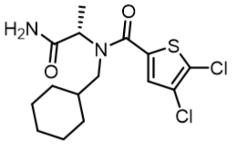

Exact Mass: 362.0623
Observed $[M+H]^+$=363,900

44

Peak Table

Detector A 254nm

Peak#	Ret. Time	Area	Height	Mark	Conc.	Area%
1	0,751	66583	8906	M	0,691	0,691
2	1,370	71819	9216	M	0,745	0,745
3	10,129	25227	3621	M	0,262	0,262
4	11,180	32665	7257	M	0,339	0,339
5	11,745	9434001	1627806	M	97,837	97,837
6	12,685	12245	3059	M	0,127	0,127
Total		9642540	1659866		100,000	100,000

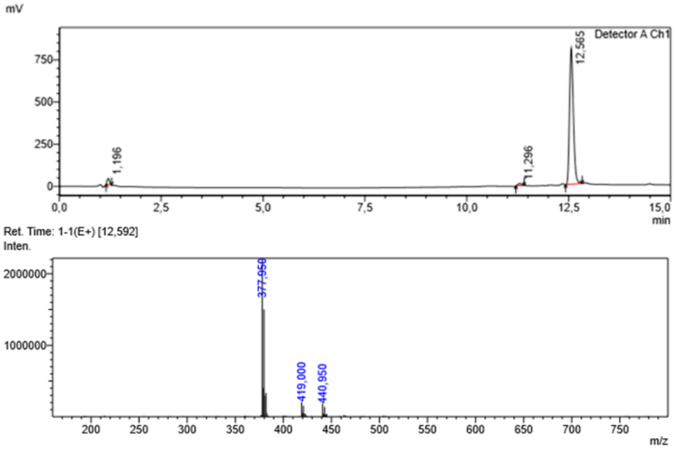

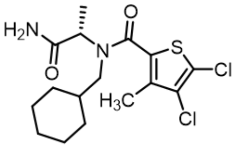

Exact Mass: 376.0779
Observed $[M+H]^+$=377,950
45

Peak Table

Detector A 254nm

Peak#	Ret. Time	Area	Height	Mark	Conc.	Area%
1	1,196	145476	35570	M	2,619	2,619
2	11,296	82932	13216	M	1,493	1,493
3	12,565	5326714	811860	M	95,888	95,888
Total		5555122	860647		100,000	100,000

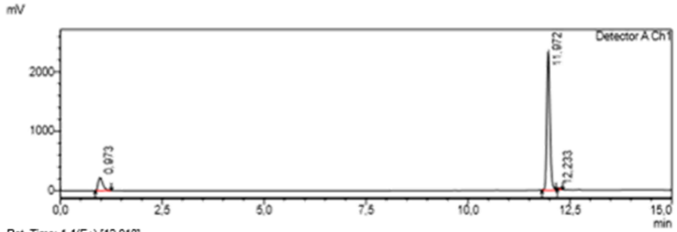

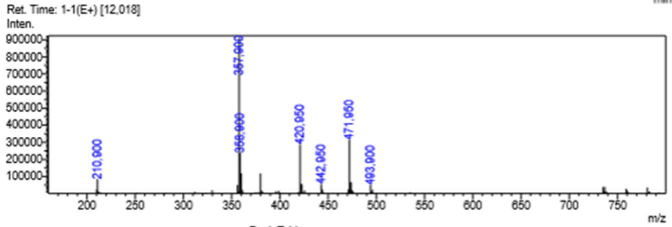

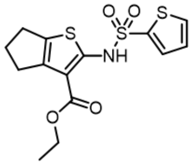

Molecular Weight: 357.4570
Observed $[M+H]^+$=357,900

46

Peak Table

Detector A 254nm

Peak#	Ret. Time	Area	Height	Mark	Conc.	Area%
1	0.973	1754768	220195	M	11.943	11.943
2	11.972	12864659	2342241		87.560	87.560
3	12.233	72893	19010	M	0.496	0.496
Total		14692320	2581445		100.000	100.000

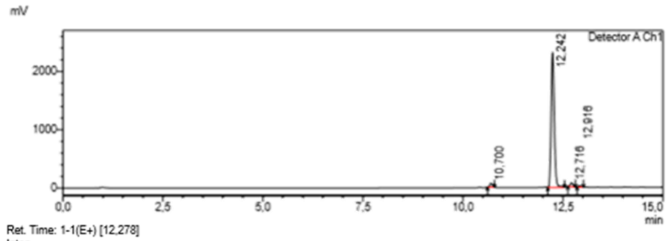

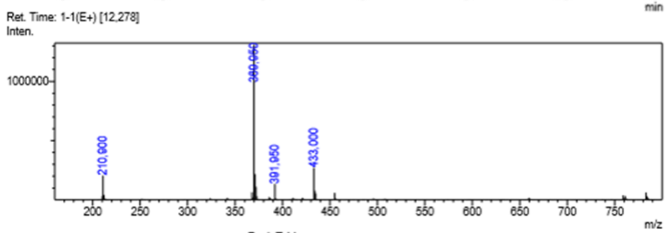

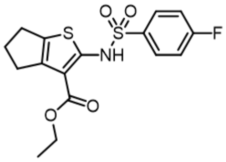

Molecular Weight: 369.4254
Observed $[M+H]^+$=369,950

47

Peak Table

Detector A 254nm

Peak#	Ret. Time	Area	Height	Mark	Conc.	Area%
1	10.700	333367	68387	M	2.489	2.489
2	12.242	12652282	2325465		94.483	94.483
3	12.716	301136	65054	M	2.249	2.249
4	12.916	104340	23670	M	0.779	0.779
Total		13391125	2482577		100.000	100.000

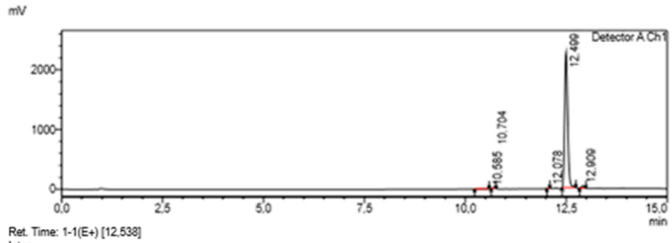

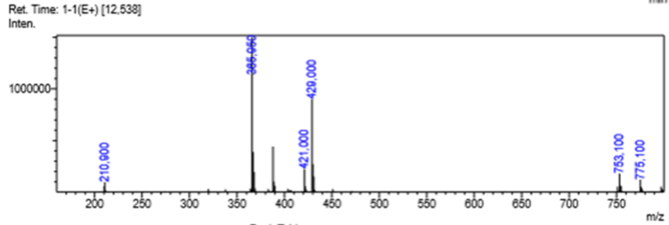

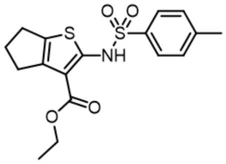

Exact Mass: 365.0756
Observed $[M+H]^+$=365,950

48

Peak Table

Detector A 254nm

Peak#	Ret. Time	Area	Height	Mark	Conc.	Area%
1	10.585	-3712	168	M	-0.030	-0.030
2	10.704	78883	18804	M	0.638	0.638
3	12.078	18116	5465	M	0.147	0.147
4	12.499	12085911	2270872	M	97.786	97.786
5	12.909	180324	37683	M	1.459	1.459
Total		12359523	2332991		100.000	100.000

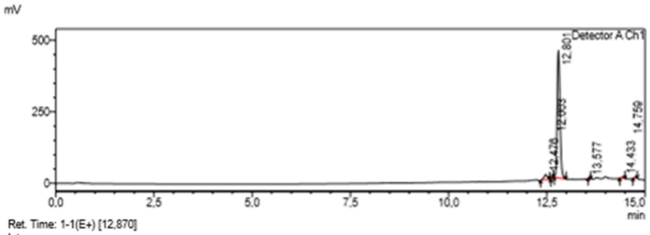

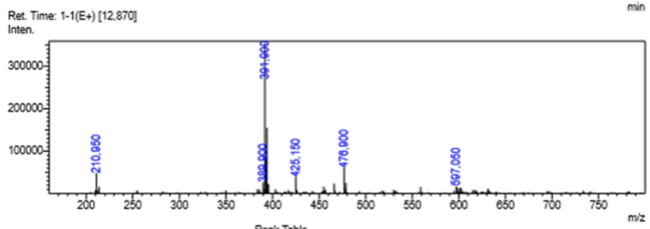

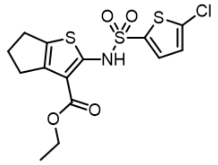

Exact Mass: 390.9773
Observed $[M+H]^+$=391,900

49

Peak Table

Detector A 254nm

Peak#	Ret. Time	Area	Height	Mark	Conc.	Area%
1	12.476	112941	18727	M	4.674	4.674
2	12.663	1353	3	M	0.056	0.056
3	12.801	2264488	447841	M	93.716	93.716
4	13.577	1887	867	M	0.078	0.078
5	14.433	20263	4595	M	0.839	0.839
6	14.759	15408	3958	M	0.638	0.638
Total		2416340	475991		100.000	100.000

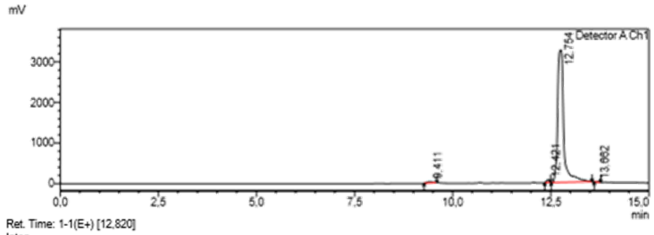

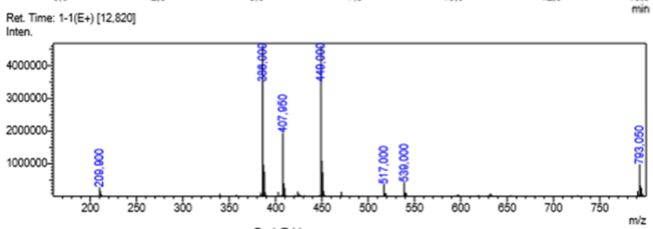

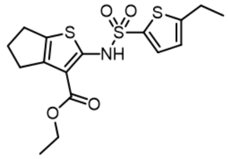

Exact Mass: 385.0476
Observed $[M+H]^+$=386,000

50

Peak Table

Detector A 254nm

Peak#	Ret. Time	Area	Height	Mark	Conc.	Area%
1	9.411	309186	27389	M	0.851	0.851
2	12.421	312762	64816	M	0.861	0.861
3	12.754	35668199	3262874	M	98.202	98.202
4	13.662	31168	5393		0.086	0.086
Total		36321317	3360472		100.000	100.000

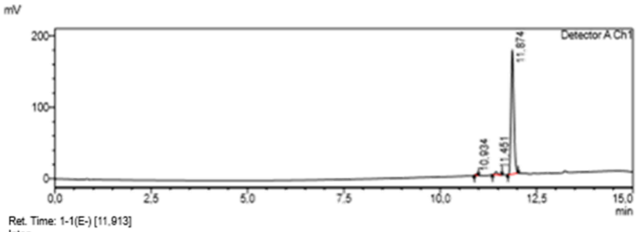

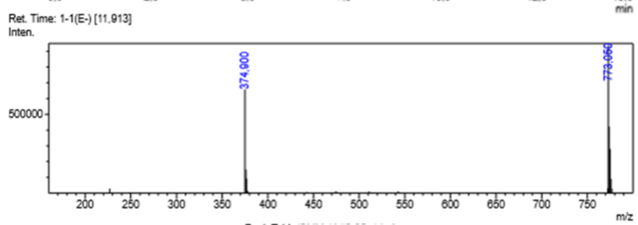

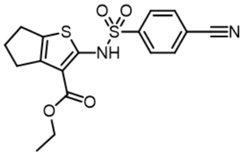

Exact Mass: 376.0551
Observed $[M-H]^-$=374,900

51

Peak Table

Detector A 254nm

Peak#	Ret. Time	Area	Height	Mark	Conc.	Area%
1	10.934	4938	1329	M	0.533	0.533
2	11.451	22135	4283	M	2.389	2.389
3	11.874	899634	174725	M	97.079	97.079
Total		926707	180336		100.000	100.000

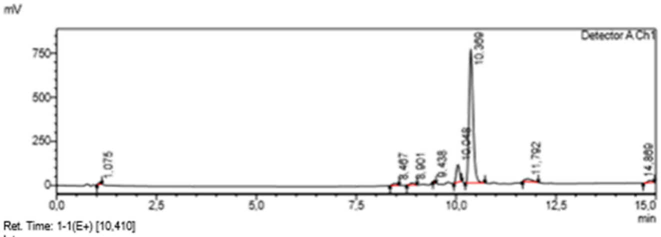

Ret. Time: 1-1(E+) [10,410]

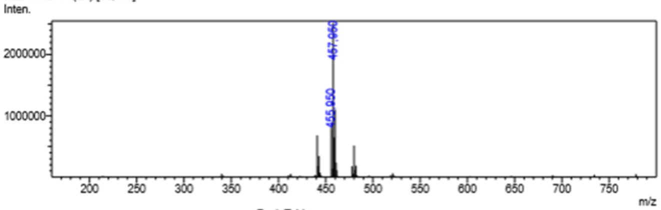

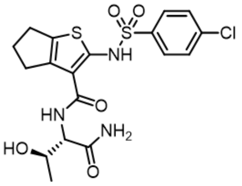

Exact Mass: 457.0533
Observed $[M+H]^+$=457,950

52

Peak Table

Detector A 254nm

Peak#	Ret. Time	Area	Height	Mark	Conc.	Area%
1	1,075	45737	9908	M	0,715	0,715
2	8,467	83518	11733	M	1,306	1,306
3	8,901	88881	12087	M	1,390	1,390
4	9,438	14986	5102	M	0,234	0,234
5	10,048	589352	100576	M	9,215	9,215
6	10,369	5263821	751738	M	82,308	82,308
7	11,792	214170	15931	M	3,349	3,349
8	14,869	94809	11171	M	1,482	1,482
Total		6395275	918247		100,000	100,000

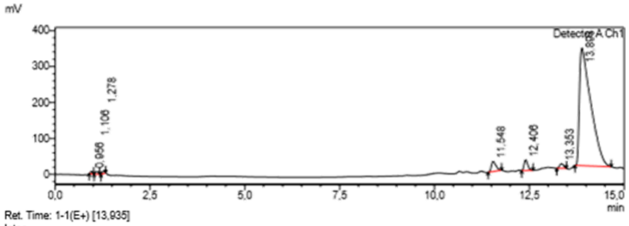

Ret. Time: 1-1(E+) [13,935]

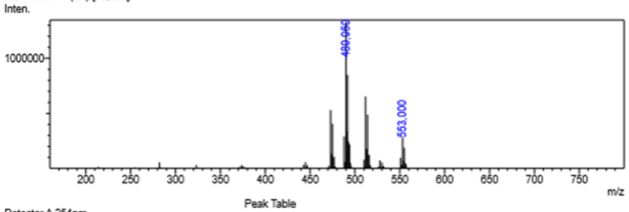

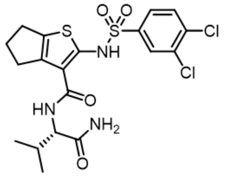

Exact Mass: 489.0351
Observed $[M+H]^+$=490,950

53

Peak Table

Detector A 254nm

Peak#	Ret. Time	Area	Height	Mark	Conc.	Area%
1	0,956	26008	7262	M	0,348	0,348
2	1,106	41020	7599	M	0,549	0,549
3	1,278	33312	7160	M	0,446	0,446
4	11,548	242503	28303	M	3,246	3,246
5	12,406	204705	29845	M	2,740	2,740
6	13,353	98295	12945	M	1,316	1,316
7	13,893	6825846	327617	M	91,356	91,356
Total		7471689	420731		100,000	100,000

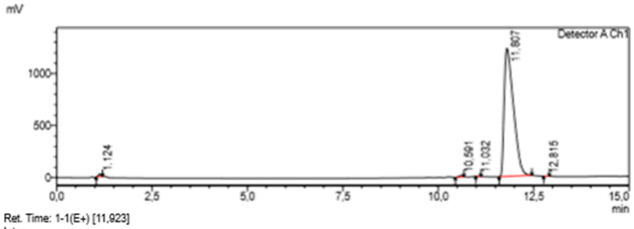

Ret. Time: 1-1(E+) [11,923]

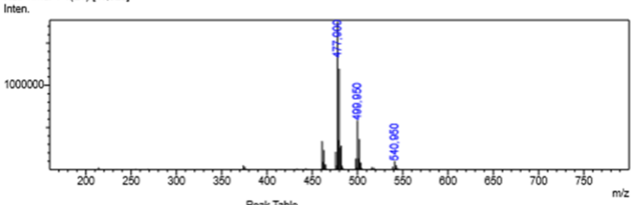

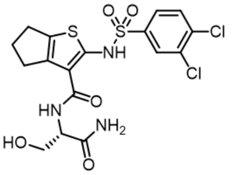

Exact Mass: 476.9987
Observed $[M+H]^+$=477,950
54

Peak Table

Detector A 254nm

Peak#	Ret. Time	Area	Height	Mark	Conc.	Area%
1	1,124	121581	26776	M	0,574	0,574
2	10,591	81518	12905	M	0,385	0,385
3	11,032	12918	2779	M	0,061	0,061
4	11,807	20959132	1232133	M	98,944	98,944
5	12,815	7730	1668	M	0,036	0,036
Total		21182879	1276262		100,000	100,000

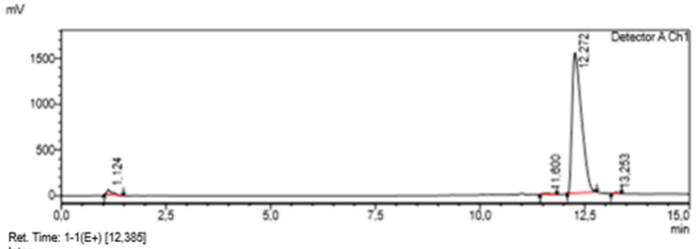

Ret. Time: 1-1(E+) [12,385]
Inten.

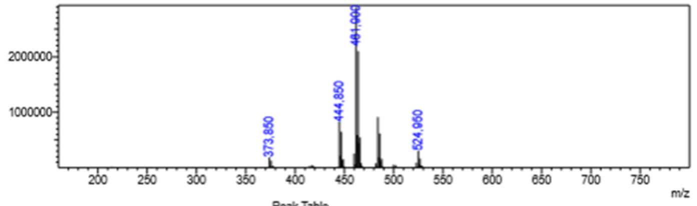

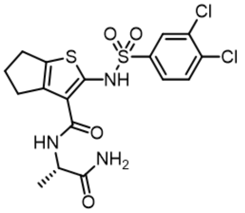

Exact Mass: 461.0038
Observed $[M+H]^+$=461,900

55

Peak Table

Detector A 254nm

Peak#	Ret. Time	Area	Height	Mark	Conc.	Area%
1	1.124	466199	56195	M	1.896	1.896
2	11.600	119135	9959	M	0.485	0.485
3	12.272	23904650	1533357	M	97.235	97.235
4	13.253	94545	13783	M	0.385	0.385
Total		24584528	1613295		100.000	100.000

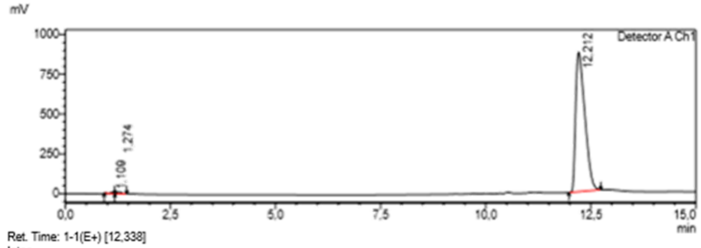

Ret. Time: 1-1(E+) [12,338]
Inten.

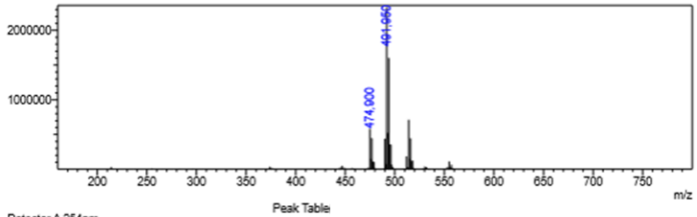

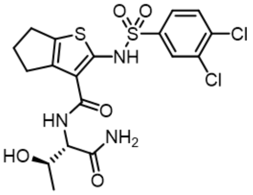

Exact Mass: 491.0143
Observed $[M+H]^+$=491,950

56

Peak Table

Detector A 254nm

Peak#	Ret. Time	Area	Height	Mark	Conc.	Area%
1	1.109	22487	6433	M	0.168	0.168
2	1.274	83710	12752	M	0.627	0.627
3	12.212	13244092	877576	M	99.205	99.205
Total		13350290	896761		100.000	100.000

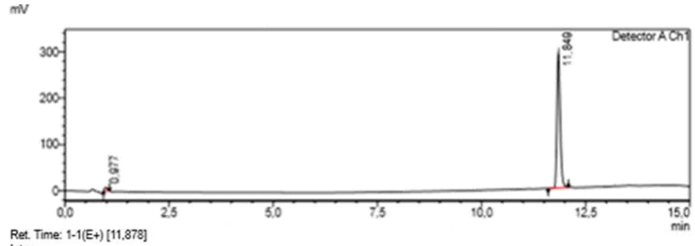

Ret. Time: 1-1(E+) [11,878]
Inten.

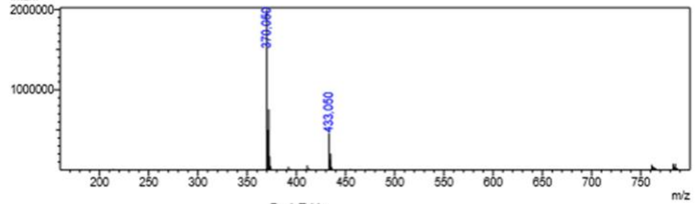

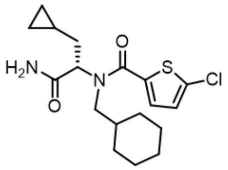

Exact Mass: 368.1325
Observed $[M+H]^+$=370,050

57

Peak Table

Detector A 254nm

Peak#	Ret. Time	Area	Height	Mark	Conc.	Area%
1	0.977	39626	8464	M	2.400	2.400
2	11.849	1611633	294300	M	97.600	97.600
Total		1651259	302764		100.000	100.000

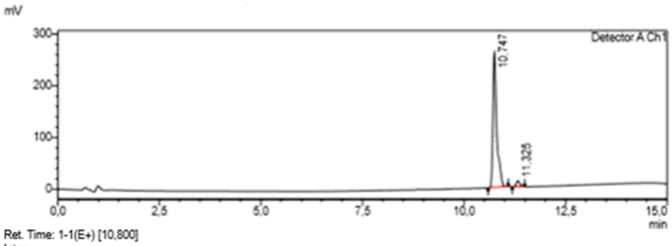

Ret. Time: 1-1(E+) [10,800]

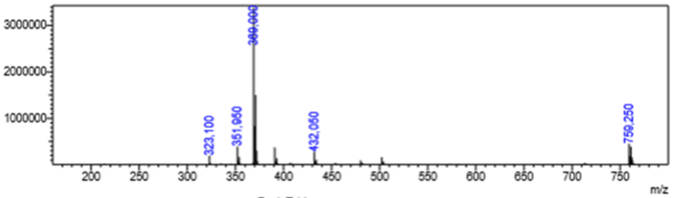

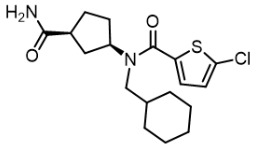

Exact Mass: 368.1325
Observed $[M+H]^+$=369,000

58

Peak Table

Detector A 254nm

Peak#	Ret. Time	Area	Height	Mark	Conc.	Area%
1	10,747	1728018	260824	M	96,130	96,130
2	11,325	69570	11723	M	3,870	3,870
Total		1797588	272547		100,000	100,000

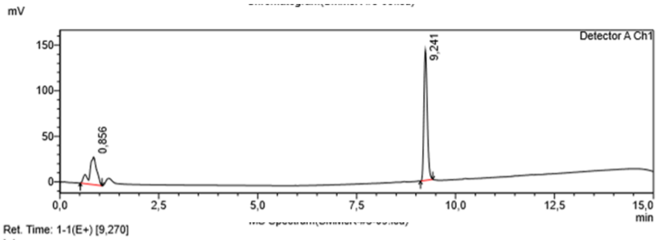

Ret. Time: 1-1(E+) [9,270]

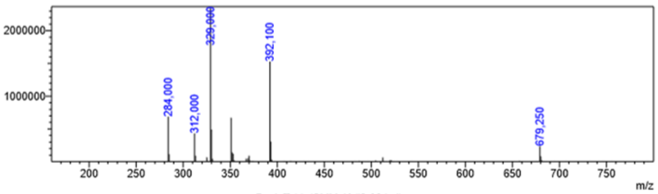

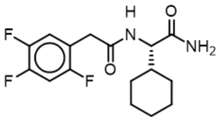

Exact Mass: 328.1399
Observed $[M+H]^+$=329,000

59

Peak Table(SMMcK-#3-09.lcd)

Detector A 254nm

Peak#	Ret. Time	Area	Height	Mark	Conc.	Area%
1	0,856	392078	30296	M	31,763	31,763
2	9,241	842296	142828	M	68,237	68,237
Total		1234373	173124		100,000	100,000

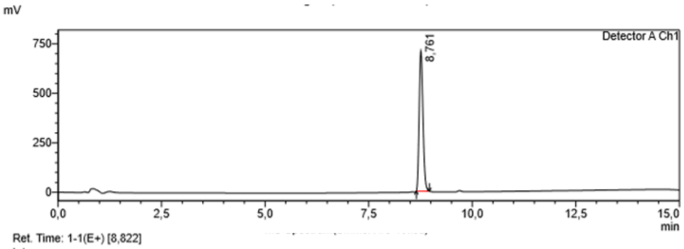

Ret. Time: 1-1(E+) [8,822]

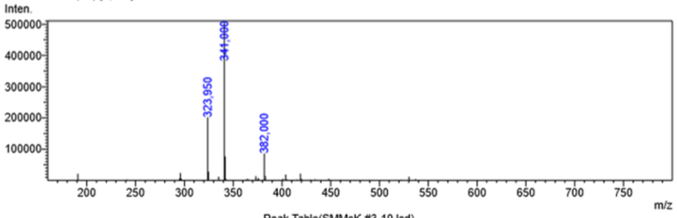

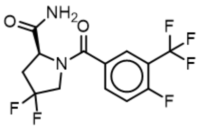

Exact Mass: 340.0646
Observed $[M+H]^+$=341,000

60

Peak Table(SMMcK-#3-10.lcd)

Detector A 254nm

Peak#	Ret. Time	Area	Height	Mark	Conc.	Area%
1	8,761	4542721	708838	M	100,000	100,000
Total		4542721	708838		100,000	100,000

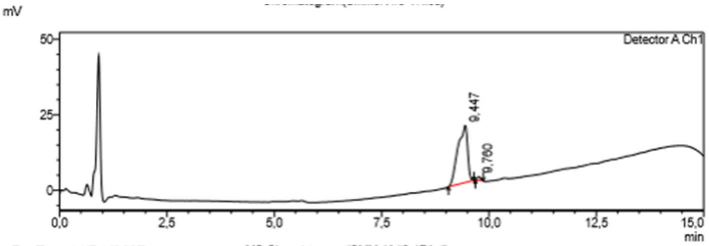

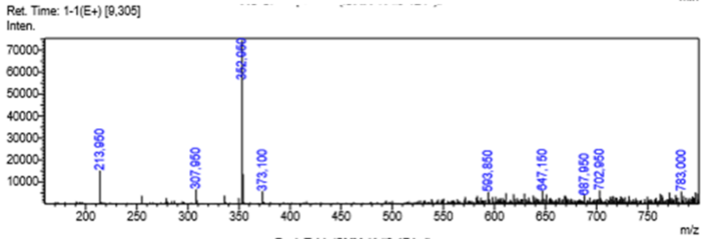

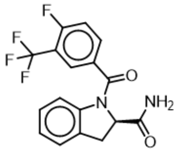

Exact Mass: 352.0835

Observed $[M+H]^{+}$= 352,950

61

Peak Table(SMMcK-#3-17.lcd)

Detector A 254nm

Peak#	Ret. Time	Area	Height	Mark	Conc.	Area%
1	9.447	294547	18869	M	97.819	97.819
2	9.760	6568	1190	M	2.181	2.181
Total		301114	20059		100.000	100.000

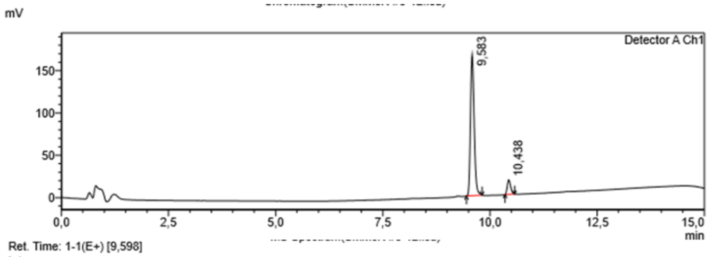

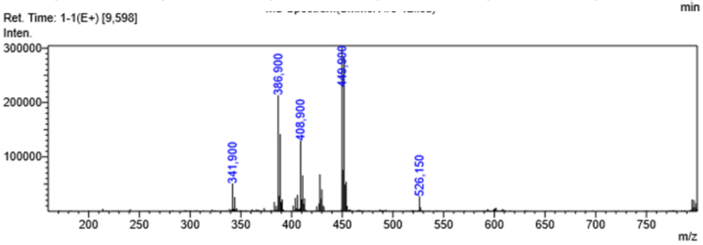

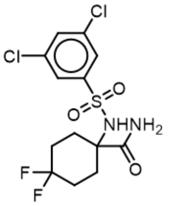

Exact Mass: 386.0070

Observed $[M+H]^{+}$= 386,900

62

Peak Table(SMMcK-#3-12.lcd)

Detector A 254nm

Peak#	Ret. Time	Area	Height	Mark	Conc.	Area%
1	9.583	995523	166846	M	91.380	91.380
2	10.438	93911	17197	M	8.620	8.620
Total		1089435	184042		100.000	100.000

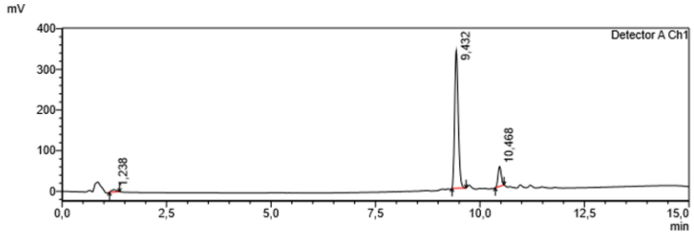

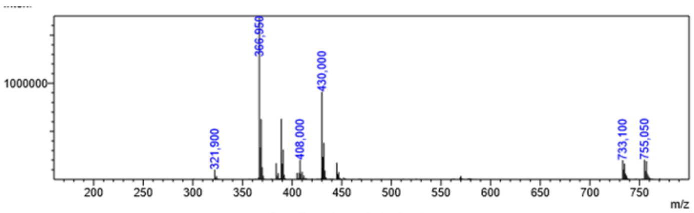

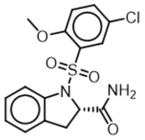

Exact Mass: 366.0441

Observed $[M+H]^{+}$= 366,950

63

Peak Table(SMMcK-#3-15.lcd)

Detector A 254nm

Peak#	Ret. Time	Area	Height	Mark	Conc.	Area%
1	1.238	46093	5222	M	1.996	1.996
2	9.432	1998816	338363	M	86.578	86.578
3	10.468	263781	48585	M	11.426	11.426
Total		2308690	392170		100.000	100.000

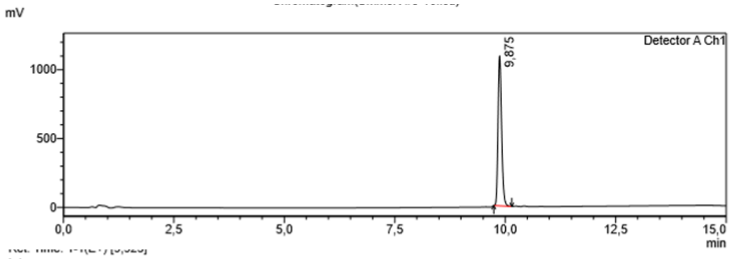

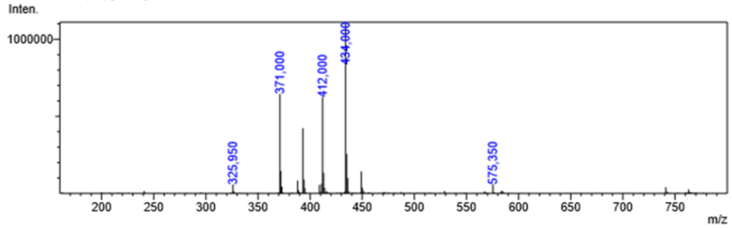

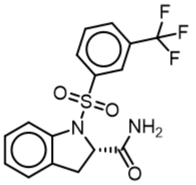

Exact Mass: 370.0599
Observed $[M+H]^+$= 371,000

64

Peak Table(SMMcK-#3-16.lcd)

Detector A 254nm

Peak#	Ret. Time	Area	Height	Mark	Conc.	Area%
1	9.875	6197784	1092240	M	100.000	100.000
Total		6197784	1092240		100.000	100.000

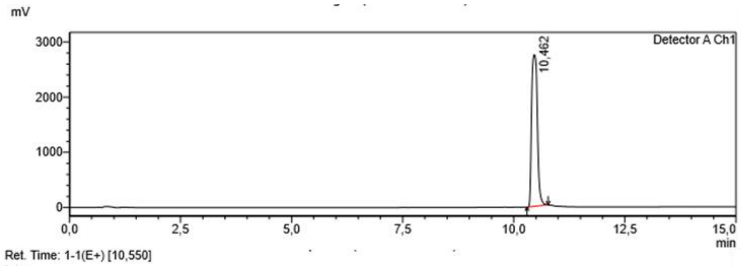

Ret. Time: 1-1(E+) [10,550]

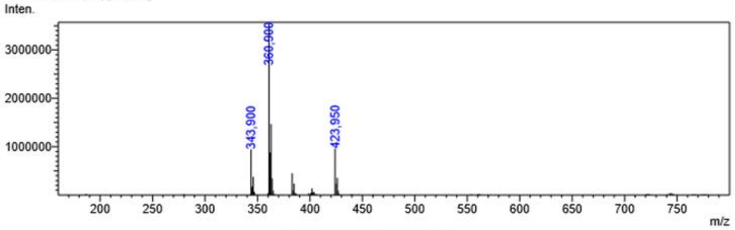

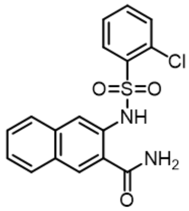

Exact Mass: 360.0335
Observed $[M+H]^+$= 360,900

65

Peak Table(SMMcK-#3-14.lcd)

Detector A 254nm

Peak#	Ret. Time	Area	Height	Mark	Conc.	Area%
1	10.462	24845236	2747796	M	100.000	100.000
Total		24845236	2747796		100.000	100.000

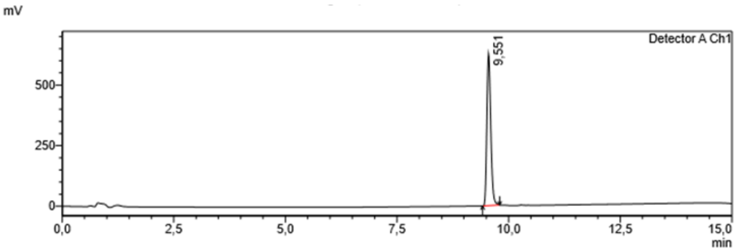

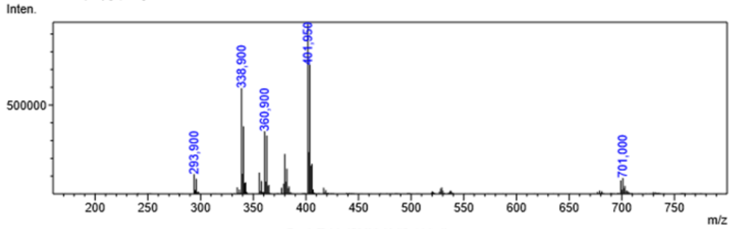

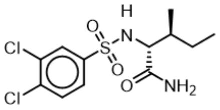

Exact Mass: 338.0259
Observed $[M+H]^+$= 338,900

66

Peak Table(SMMcK-#3-11.lcd)

Detector A 254nm

Peak#	Ret. Time	Area	Height	Mark	Conc.	Area%
1	9.551	3851196	626028	M	100.000	100.000
Total		3851196	626028		100.000	100.000

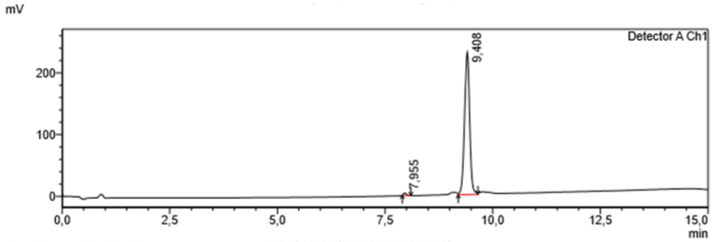

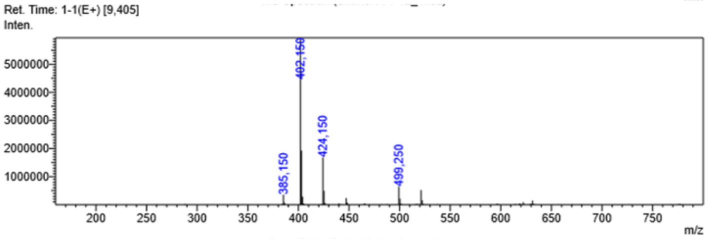

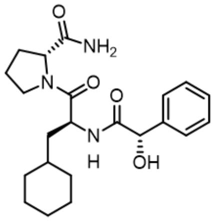

Exact Mass: 401.2315
Observed $[M+H]^+$=402,150

67

Peak Table(SMMcK-#1-12_1.lcd)

Detector A 254nm

Peak#	Ret. Time	Area	Height	Mark	Conc.	Area%
1	7,955	17703	3767	M	0,937	0,937
2	9,408	1871433	230356		99,063	99,063
Total		1889136	234122		100,000	100,000

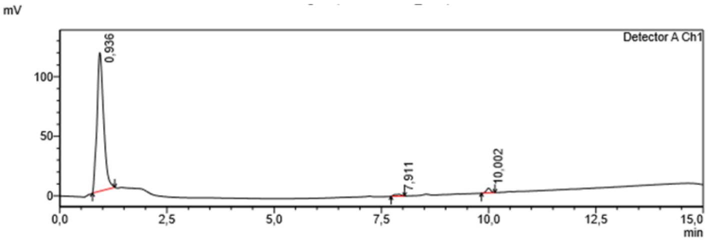

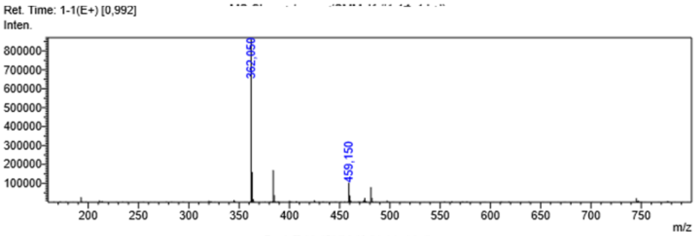

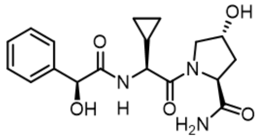

Exact Mass: 361.1638
Observed $[M+H]^+$=362,050

68

Peak Table(SMMcK-#1-14_1.lcd)

Detector A 254nm

Peak#	Ret. Time	Area	Height	Mark	Conc.	Area%
1	0,936	1226253	115928	M	96,504	96,504
2	7,911	17261	1530	M	1,358	1,358
3	10,002	27160	3915	M	2,137	2,137
Total		1270673	121373		100,000	100,000

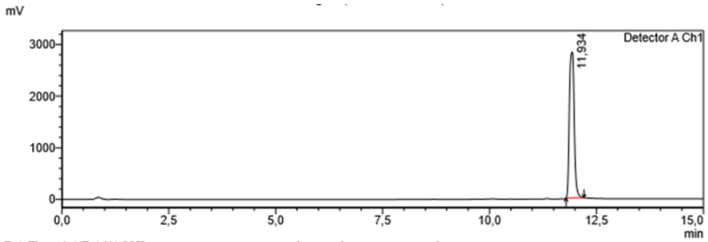

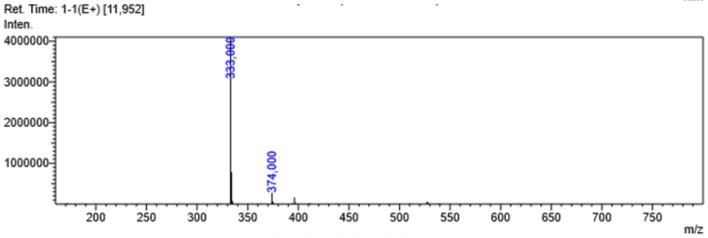

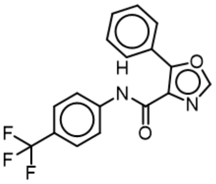

Exact Mass: 332.0773
Observed $[M+H]^+$=333,000

69

Peak Table(SMMcK-#3-03.lcd)

Detector A 254nm

Peak#	Ret. Time	Area	Height	Mark	Conc.	Area%
1	11,934	22365117	2821972	M	100,000	100,000
Total		22365117	2821972		100,000	100,000

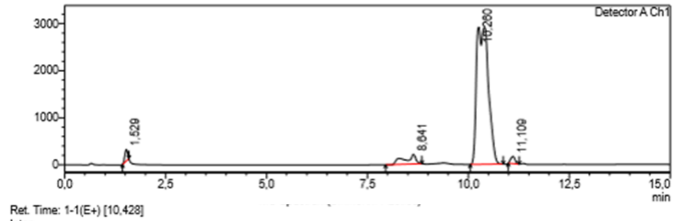

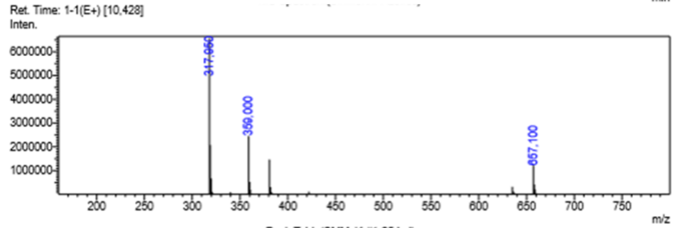

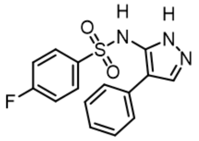

Exact Mass: 317.0634
Observed $[M+H]^+$=317,950

70

Peak Table(SMMcK-#1-25.lcd)

Detector A 254nm

Peak#	Ret. Time	Area	Height	Mark	Conc.	Area%
1	1.529	1180602	239956	M	1.790	1.790
2	8.641	3853578	203452	M	5.843	5.843
3	10.260	59792610	2913843	M	90.654	90.654
4	11.109	1130224	151468	M	1.714	1.714
Total		65957011	3508719		100.000	100.000

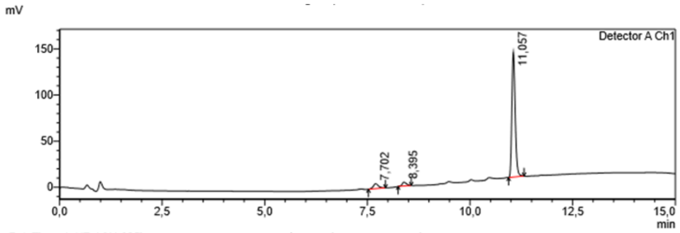

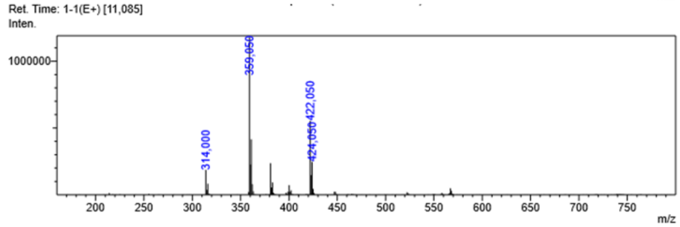

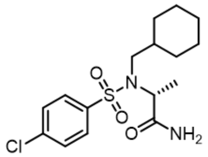

Exact Mass: 358.1118
Observed $[M+H]^+$=359,050

71

Peak Table(JJM.036-18.2.lcd)

Detector A 254nm

Peak#	Ret. Time	Area	Height	Mark	Conc.	Area%
1	7.702	47877	5275	M	5.661	5.661
2	8.395	28836	3993	M	3.410	3.410
3	11.057	768985	135761	M	90.929	90.929
Total		845697	145028		100.000	100.000

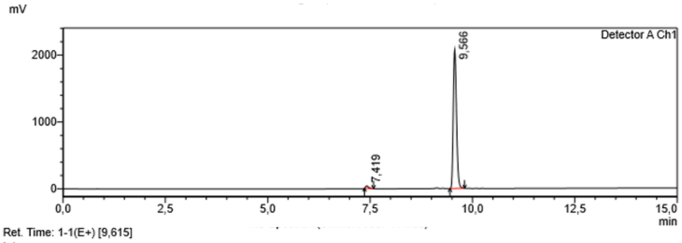

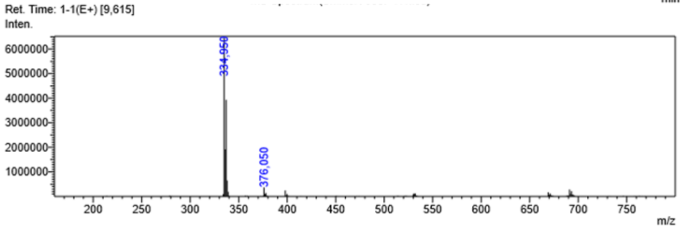

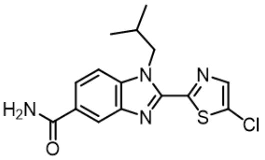

Exact Mass: 334.0655
Observed $[M+H]^+$=334,950

72

Peak Table(SMMcK-053F-f11.lcd)

Detector A 254nm

Peak#	Ret. Time	Area	Height	Mark	Conc.	Area%
1	7.419	169754	35258	M	1.469	1.469
2	9.566	11384692	2072264	M	98.531	98.531
Total		11554446	2107522		100.000	100.000

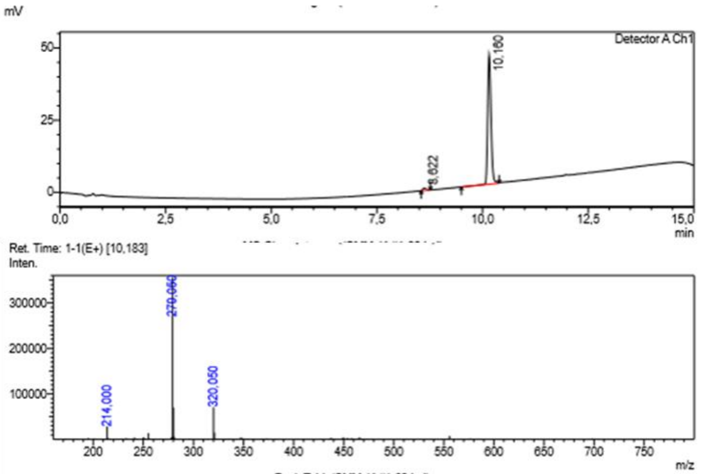

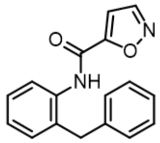

Exact Mass: 278.1055

Observed $[M+H]^+$=279,050

73

Peak Table(SMMcK-#1-69.lcd)

Detector A 254nm

Peak#	Ret. Time	Area	Height	Mark	Conc.	Area%
1	8.622	4341	777	M	1.735	1.735
2	10.160	245831	44902		98.265	98.265
Total		250171	45678		100.000	100.000

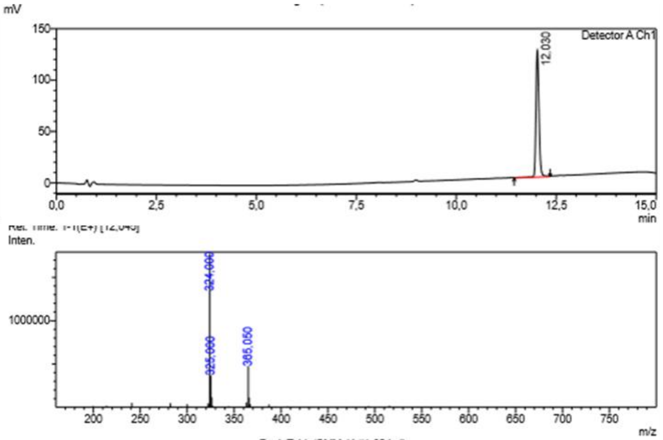

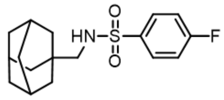

Exact Mass: 323.1355

Observed $[M+H]^+$=324,000

74

Peak Table(SMMcK-#1-82.lcd)

Detector A 254nm

Peak#	Ret. Time	Area	Height	Mark	Conc.	Area%
1	12.030	660358	123569		100.000	100.000
Total		660358	123569		100.000	100.000

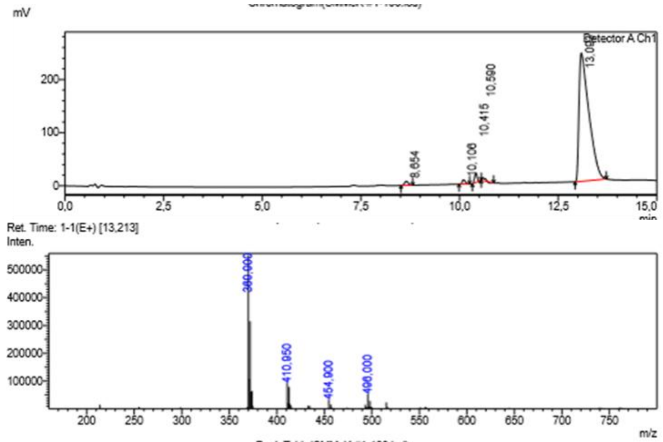

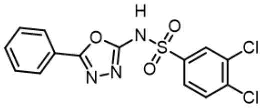

Exact Mass: 368.9742

Observed $[M+H]^+$=369,900

75

Peak Table(SMMcK-#1-100.lcd)

Detector A 254nm

Peak#	Ret. Time	Area	Height	Mark	Conc.	Area%
1	8.654	51639	7721	M	1.186	1.186
2	10.106	41671	8058	M	0.957	0.957
3	10.415	73150	17648	M	1.681	1.681
4	10.590	21203	4421	M	0.487	0.487
5	13.093	4165176	240879	M	95.689	95.689
Total		4352838	278727		100.000	100.000

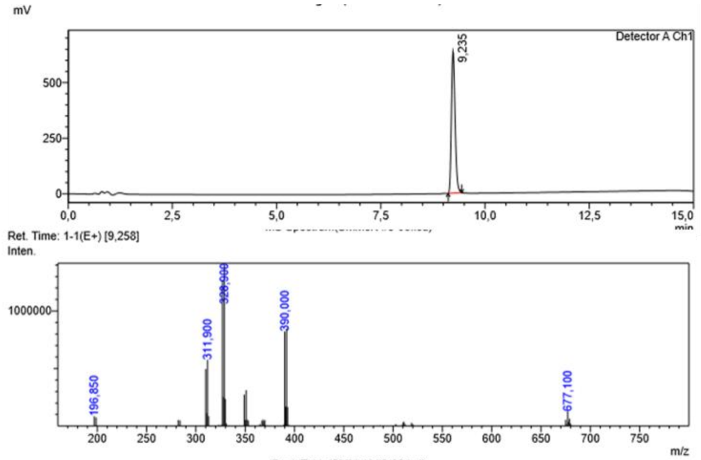

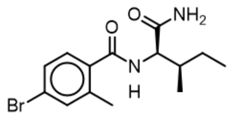

Exact Mass: 326.0630

Observed $[M+H]^+$=326,900

76

Peak Table(SMMcK-#3-08.lcd)

Detector A 254nm

Peak#	Ret. Time	Area	Height	Mark	Conc.	Area%
1	9,235	3891504	637493	M	100,000	100,000
Total		3891504	637493		100,000	100,000

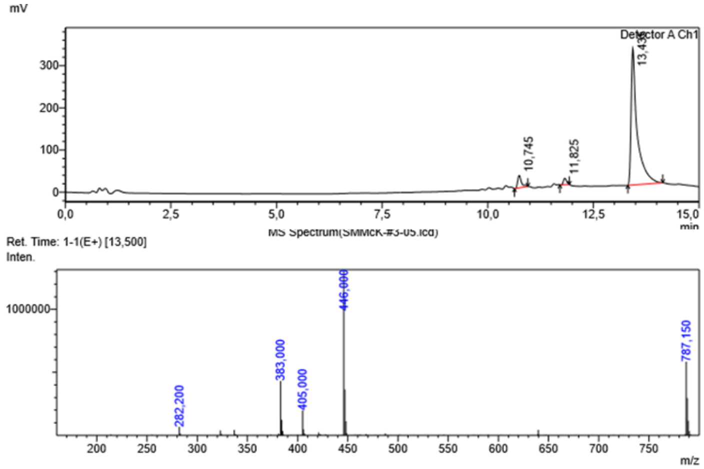

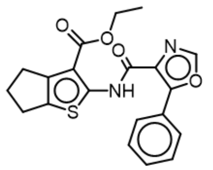

Exact Mass: 382.0987

Observed $[M+H]^+$=383,000

77

Peak Table(SMMcK-#3-05.lcd)

Detector A 254nm

Peak#	Ret. Time	Area	Height	Mark	Conc.	Area%
1	10,745	181853	29361	M	5,165	5,165
2	11,825	77459	14928	M	2,200	2,200
3	13,435	3261579	322247	M	92,635	92,635
Total		3520891	366536		100,000	100,000

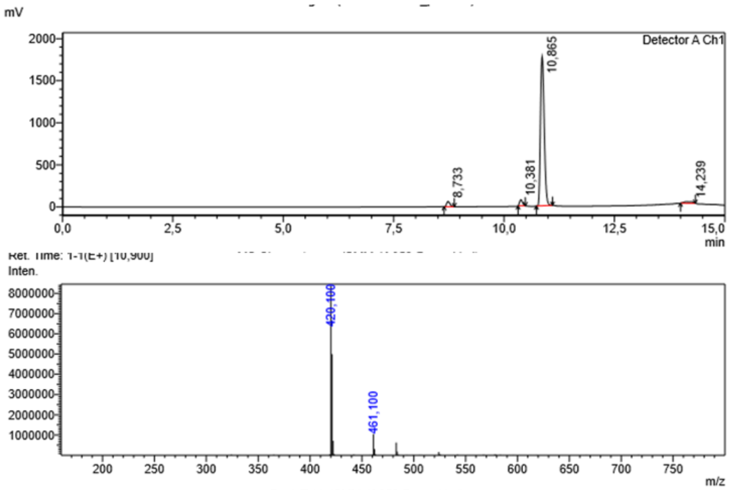

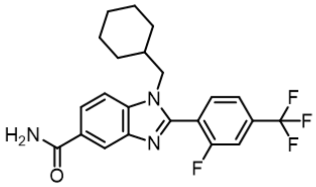

Exact Mass: 419.1621

Observed $[M+H]^+$=420,100

78

Peak Table(SMMcK-053-B_prod.lcd)

Detector A 254nm

Peak#	Ret. Time	Area	Height	Mark	Conc.	Area%
1	8,733	321218	61354	M	2,830	2,830
2	10,381	344434	72398	M	3,035	3,035
3	10,865	10436221	1776569	M	91,948	91,948
4	14,239	248302	17612	M	2,188	2,188
Total		11350174	1927932		100,000	100,000

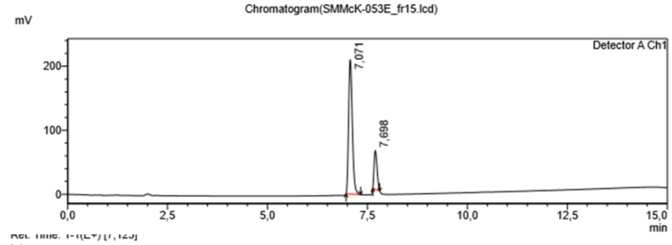

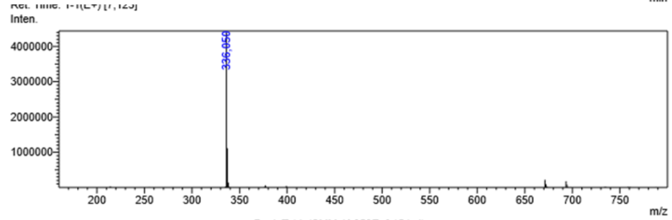

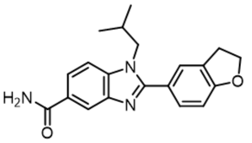

Exact Mass: 335.1634
Observed $[M+H]^+$=336,050

79

Peak Table(SMMcK-053E_fr15.lcd)

Detector A 254nm

Peak#	Ret. Time	Area	Height	Mark	Conc.	Area%
1	7,071	1364443	209406	M	81,211	81,211
2	7,698	315674	60126	M	18,789	18,789
Total		1680117	269532		100,000	100,000

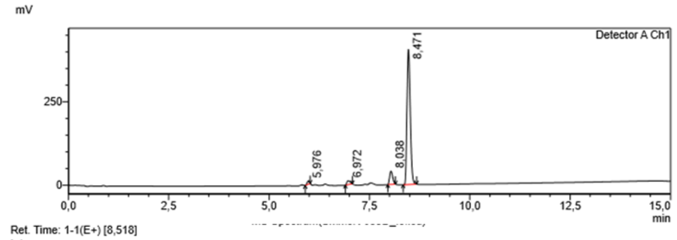

Ret. Time: 1-1(E+) [8,518]

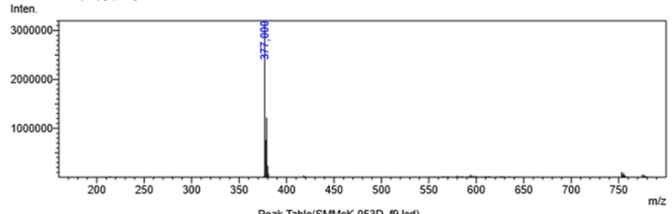

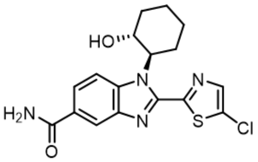

Exact Mass: 376.0761
Observed $[M+H]^+$=377,000

80

Peak Table(SMMcK-053D_f9.lcd)

Detector A 254nm

Peak#	Ret. Time	Area	Height	Mark	Conc.	Area%
1	5,976	36919	8981	M	1,438	1,438
2	6,972	67875	11574	M	2,643	2,643
3	8,038	209381	39285	M	8,153	8,153
4	8,471	2253882	404455	M	87,766	87,766
Total		2568056	464296		100,000	100,000

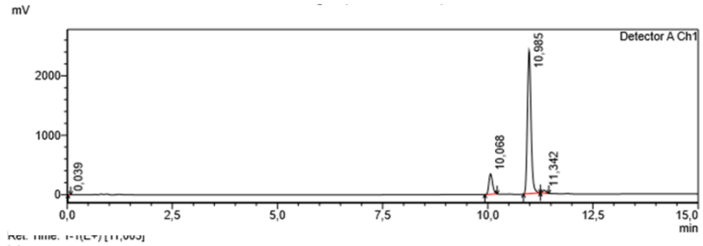

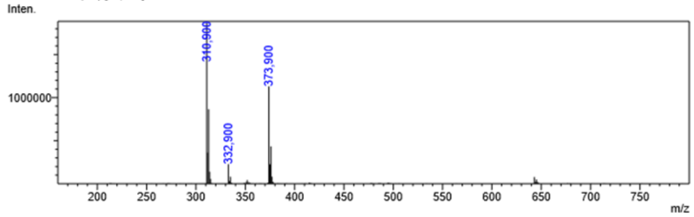

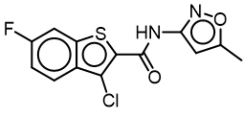

Exact Mass: 309.9979
Observed [M+H]+=310,900

81

Peak Table(SMMcK-#3-04.lcd)

Detector A 254nm

Peak#	Ret. Time	Area	Height	Mark	Conc.	Area%
1	0,039	1180	342		0,007	0,007
2	10,068	2109054	343538	M	12,387	12,387
3	10,985	14663665	2407378	M	86,125	86,125
4	11,342	252182	49365	M	1,481	1,481
Total		17026081	2800624		100,000	100,000

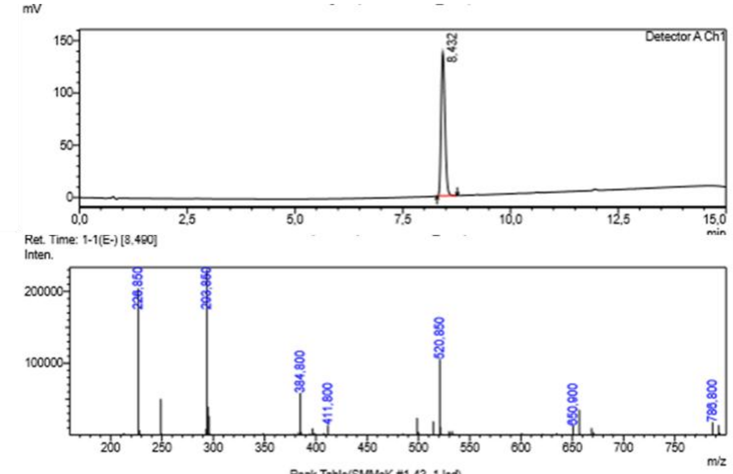

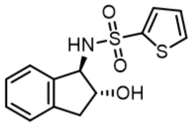

Exact Mass: 295.0337
Observed [M-H]⁻=293,950

82

Peak Table(SMMcK-#1-43_1.lcd)

Detector A 254nm

Peak#	Ret. Time	Area	Height	Mark	Conc.	Area%
1	8.432	824768	137285		100.000	100.000
Total		824768	137285		100.000	100.000

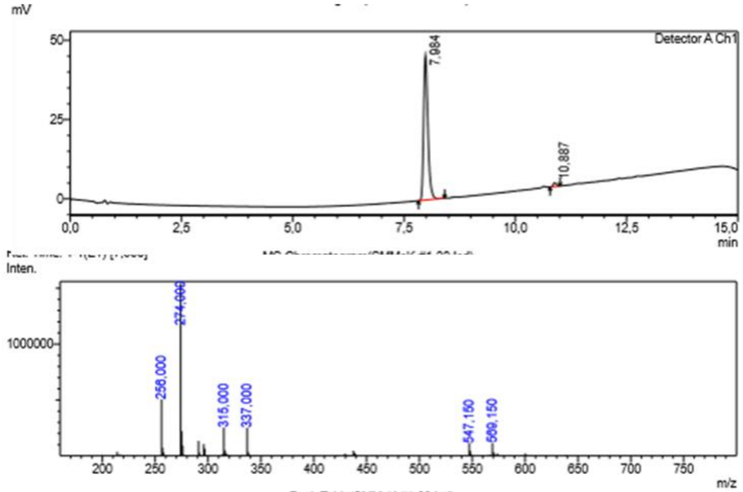

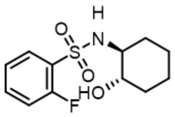

Exact Mass: 273.0835
Observed $[M+H]^+$=274,000

83

Peak Table(SMMcK-#1-33.lcd)

Detector A 254nm

Peak#	Ret. Time	Area	Height	Mark	Conc.	Area%
1	7.984	303904	45663	M	98.092	98.092
2	10.887	5912	1154	M	1.908	1.908
Total		309816	46817		100.000	100.000

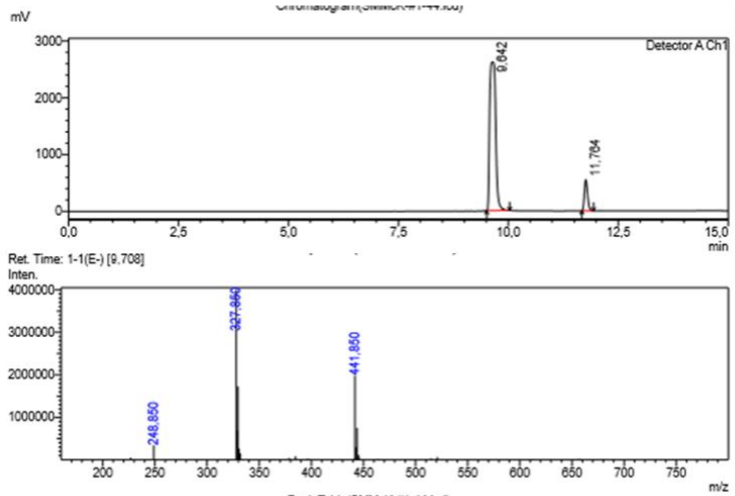

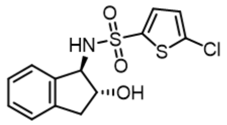

Exact Mass: 328.9947
Observed [M-H]⁻=327,850

84

Peak Table(SMMcK-#1-44.lcd)

Detector A 254nm

Peak#	Ret. Time	Area	Height	Mark	Conc.	Area%
1	9.642	25010413	2621031	M	90.149	90.149
2	11.764	2733040	543454	M	9.851	9.851
Total		27743452	3164485		100.000	100.000

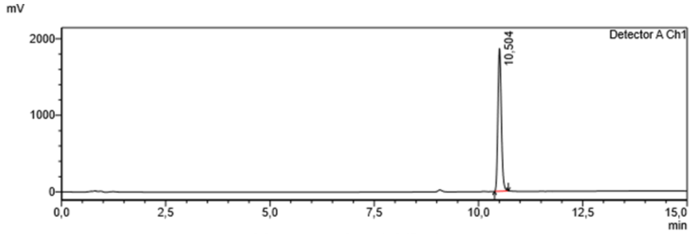

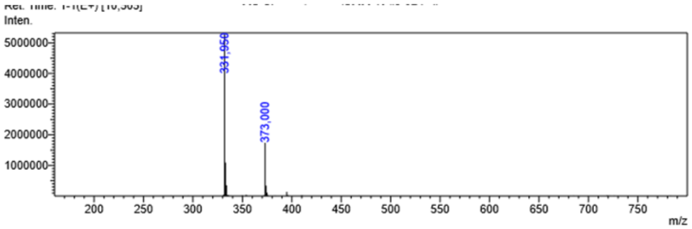

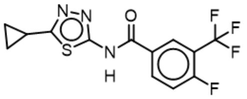

Exact Mass: 331.0402

Observed $[M+H]^+$=331,950

85

Peak Table(SMMcK-#3-07.lcd)

Detector A 254nm

Peak#	Ret. Time	Area	Height	Mark	Conc.	Area%
1	10.504	10600138	1858858	M	100.000	100.000
Total		10600138	1858858		100.000	100.000

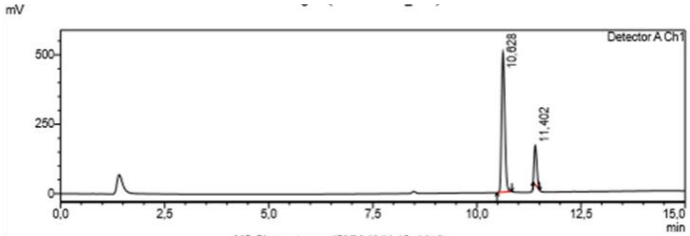

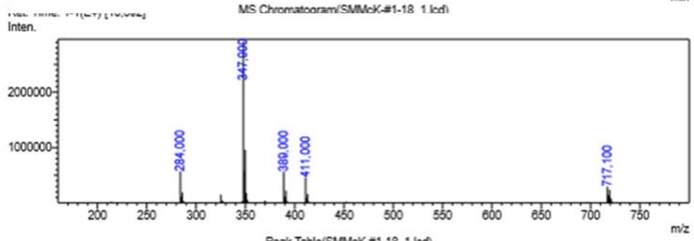

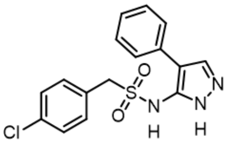

Exact Mass: 347.0495

Observed $[M+H]^+$=347,900

86

Peak Table(SMMcK-#1-18_1.lcd)

Detector A 254nm

Peak#	Ret. Time	Area	Height	Mark	Conc.	Area%
1	10.628	2684679	503415	M	81.296	81.296
2	11.402	617680	140886	M	18.704	18.704
Total		3302359	644300		100.000	100.000

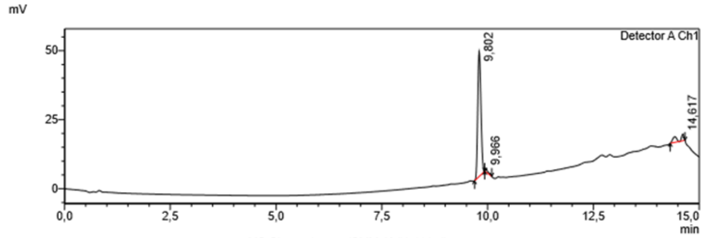

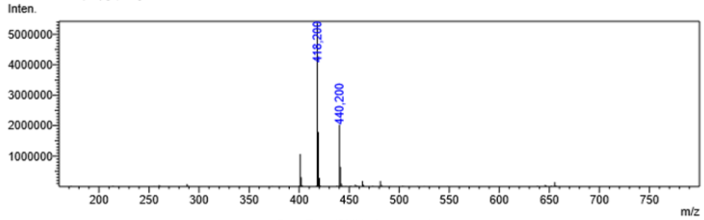

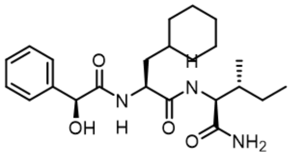

Exact Mass: 417.2628

Observed $[M+H]^+$=418,200

87

Peak Table(SMMcK-#1-11.lcd)

Detector A 254nm

Peak#	Ret. Time	Area	Height	Mark	Conc.	Area%
1	9.802	237067	45260	M	89.166	89.166
2	9.966	6172	1396	M	2.321	2.321
3	14.617	22633	2357	M	8.513	8.513
Total		265872	49014		100.000	100.000

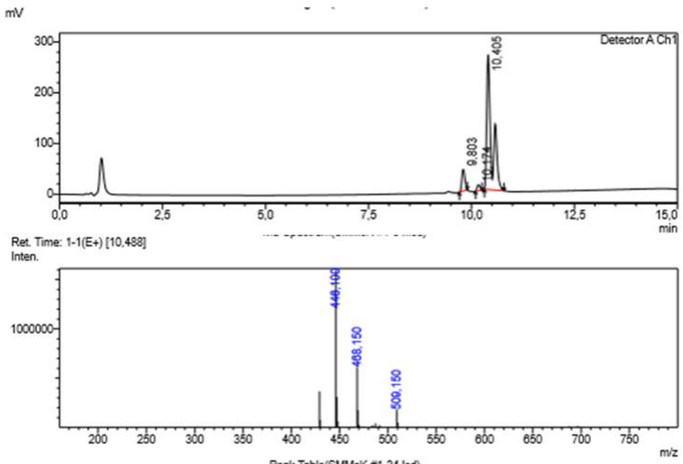

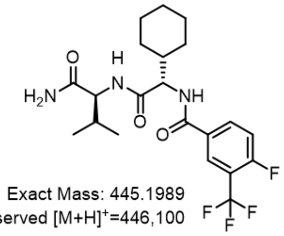

Exact Mass: 445.1989
Observed $[M+H]^+$=446,100

88

Peak Table(SMMcK-#1-34.lcd)

Detector A 254nm

Peak#	Ret. Time	Area	Height	Mark	Conc.	Area%
1	9.803	220733	42718	M	9.015	9.015
2	10.174	59463	11785	M	2.429	2.429
3	10.405	2168253	265001	M	88.556	88.556
Total		2448449	319504		100.000	100.000

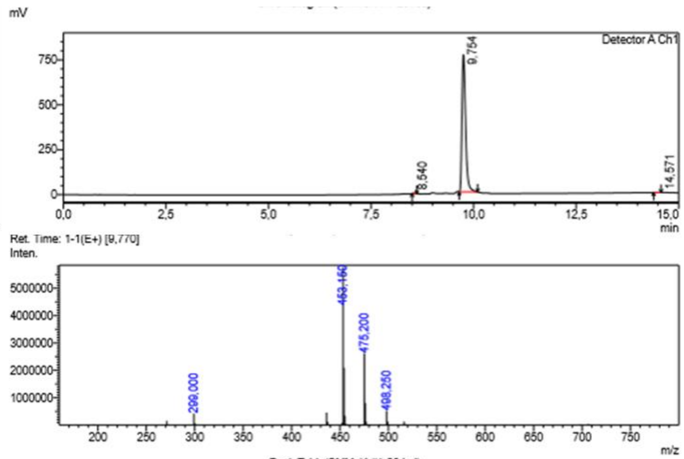

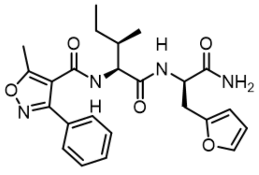

Exact Mass: 452.2060
Observed $[M+H]^+$=453,150

89

Peak Table(SMMcK-#1-26.lcd)

Detector A 254nm

Peak#	Ret. Time	Area	Height	Mark	Conc.	Area%
1	8.540	6103	1710	M	0.130	0.130
2	9.754	4691904	762780	M	99.864	99.864
3	14.571	270	5	M	0.006	0.006
Total		4698278	764494		100.000	100.000

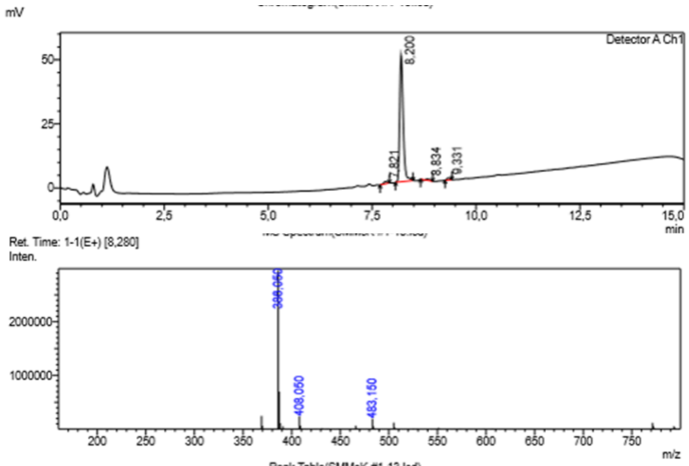

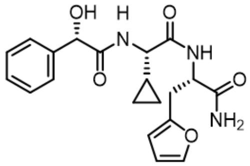

Exact Mass: 385.1638
Observed $[M+H]^+$=386,050

90

Peak Table(SMMcK-#1-13.lcd)

Detector A 254nm

Peak#	Ret. Time	Area	Height	Mark	Conc.	Area%
1	7.821	4988	829	M	1.649	1.649
2	8.200	291315	49455	M	96.274	96.274
3	8.834	2280	483	M	0.754	0.754
4	9.331	4006	809	M	1.324	1.324
Total		302589	51576		100.000	100.000

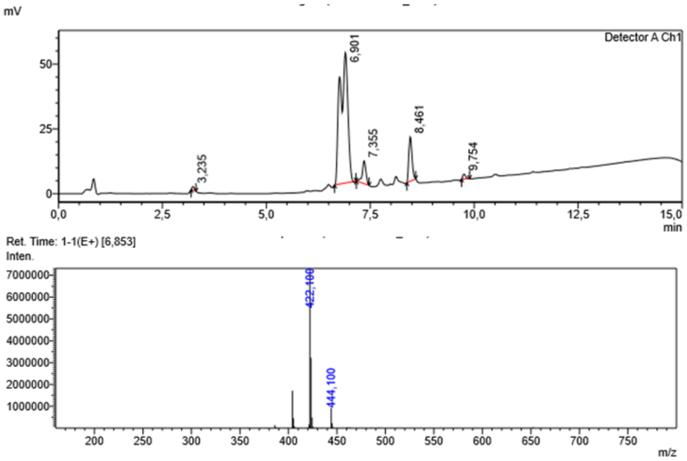

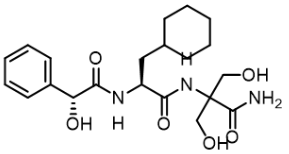

Exact Mass: 421.2213

Observed $[M+H]^+$=422,100

91

Peak Table(SMMcK-#1-15_1.lcd)

Detector A 254nm

Peak#	Ret. Time	Area	Height	Mark	Conc.	Area%
1	3,235	5366	1328	M	0,654	0,654
2	6,901	663384	50227	M	80,873	80,873
3	7,355	54122	8759	M	6,598	6,598
4	8,461	88952	17062	M	10,844	10,844
5	9,754	8455	1795	M	1,031	1,031
Total		820279	79171		100,000	100,000

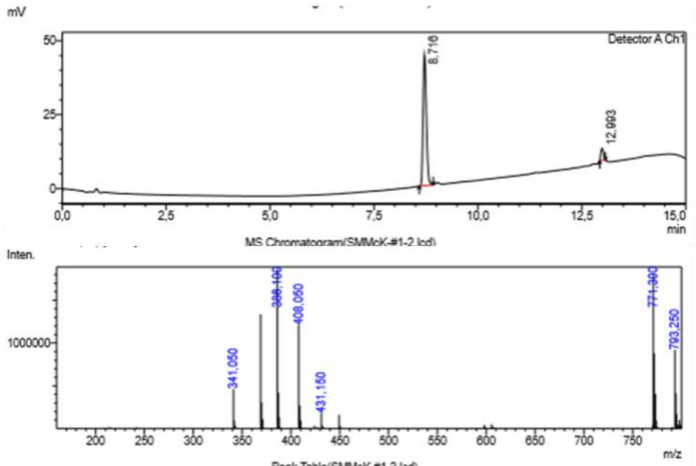

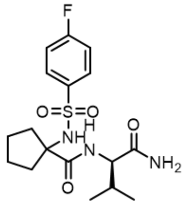

Exact Mass: 385.1472

Observed $[M+H]^+$=386,100

92

Peak Table(SMMcK-#1-2.lcd)

Detector A 254nm

Peak#	Ret. Time	Area	Height	Mark	Conc.	Area%
1	8,716	243471	44330		93,050	93,050
2	12,993	18186	4059	M	6,950	6,950
Total		261657	48389		100,000	100,000

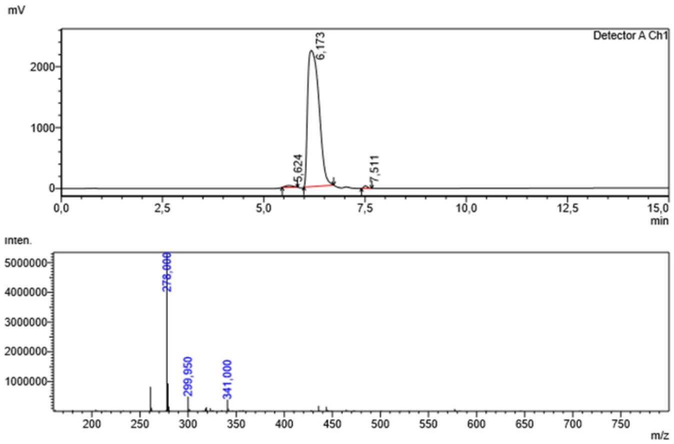

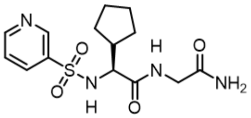

Exact Mass: 340,1205

Observed $[M+H]^+$=341,000

93

Detector A 254nm

Peak#	Ret. Time	Area	Height	Mark	Conc.	Area%
1	5,624	378385	27876	M	0,857	0,857
2	6,173	43491973	2233650	M	98,541	98,541
3	7,511	265713	41363	M	0,602	0,602
Total		44136071	2302889		100,000	100,000

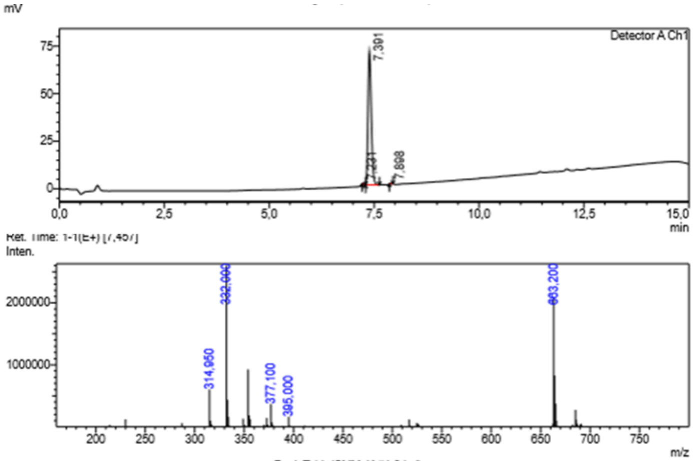

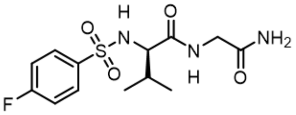

Exact Mass: 331.1002
Observed $[M+H]^+$=332,000

94

Peak Table(SMMcK-#1-3.lcd)

Detector A 254nm

Peak#	Ret. Time	Area	Height	Mark	Conc.	Area%
1	7,231	221	244	M	0,057	0,057
2	7,391	383729	70499	M	99,688	99,688
3	7,898	979	328	M	0,254	0,254
Total		384929	71072		100,000	100,000

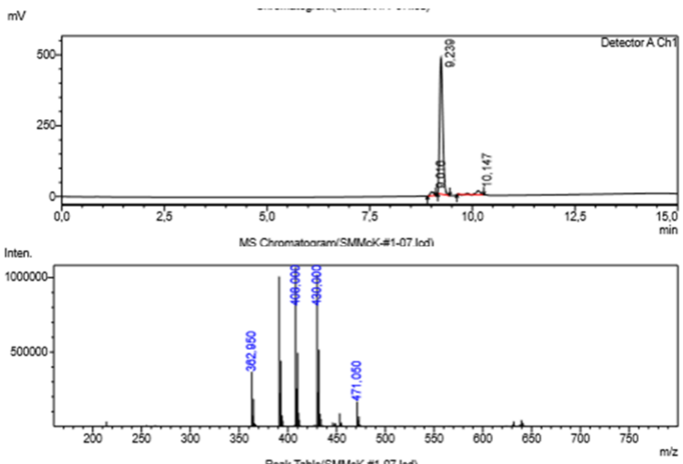

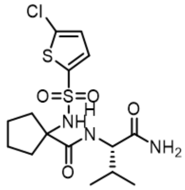

Exact Mass: 407.0740
Observed $[M+H]^+$=408,000

95

Peak Table(SMMcK-#1-07.lcd)

Detector A 254nm

Peak#	Ret. Time	Area	Height	Mark	Conc.	Area%
1	9,016	77183	13733	M	2,842	2,842
2	9,239	2539651	480869	M	93,526	93,526
3	10,147	98628	13745	M	3,632	3,632
Total		2715461	508347		100,000	100,000

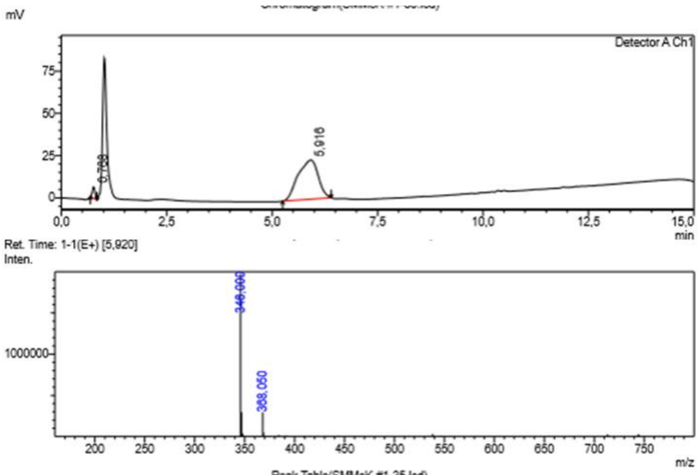

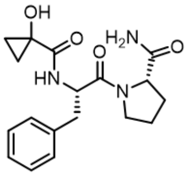

Exact Mass: 345.1689
Observed $[M+H]^+$=346,000

96

Peak Table(SMMcK-#1-35.lcd)

Detector A 254nm

Peak#	Ret. Time	Area	Height	Mark	Conc.	Area%
1	0,768	28348	6699	M	3,546	3,546
2	5,916	771017	23167	M	96,454	96,454
Total		799365	29865		100,000	100,000

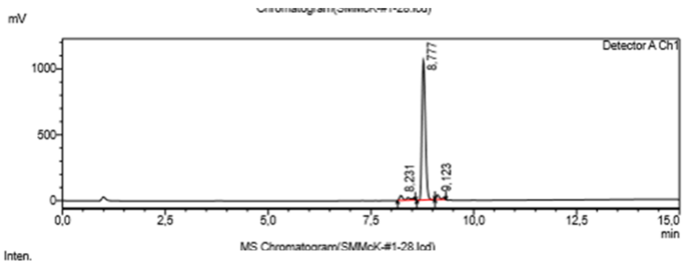

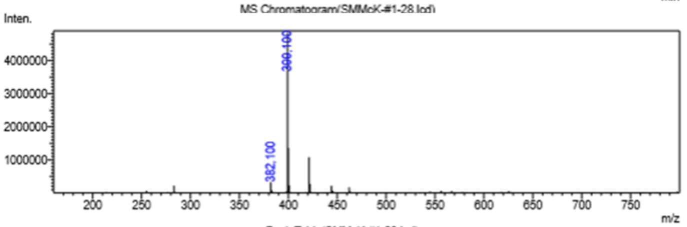

Peak Table(SMMcK-#1-28.lcd)

Detector A 254nm

Peak#	Ret. Time	Area	Height	Mark	Conc.	Area%
1	8.231	351541	35467	M	5.283	5.283
2	8.777	6081671	1055717	M	91.404	91.404
3	9.123	220399	34761	M	3.312	3.312
Total		6653611	1126945		100.000	100.000

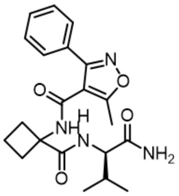

Exact Mass: 398.1954

Observed $[M+H]^+$=399,100

97

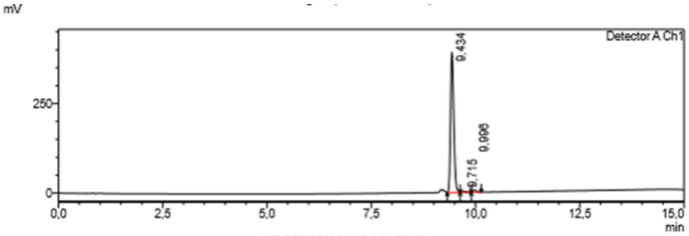

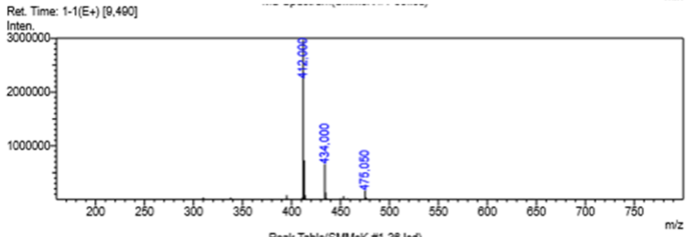

Peak Table(SMMcK-#1-36.lcd)

Detector A 254nm

Peak#	Ret. Time	Area	Height	Mark	Conc.	Area%
1	9.434	2112523	393131		97.269	97.269
2	9.715	30178	3356	V	1.390	1.390
3	9.996	29127	5032	V	1.341	1.341
Total		2171829	401519		100.000	100.000

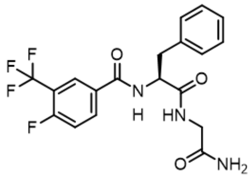

Exact Mass: 411.1206

Observed $[M+H]^+$=412,000

98

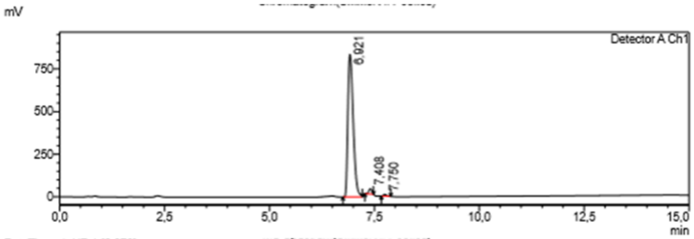

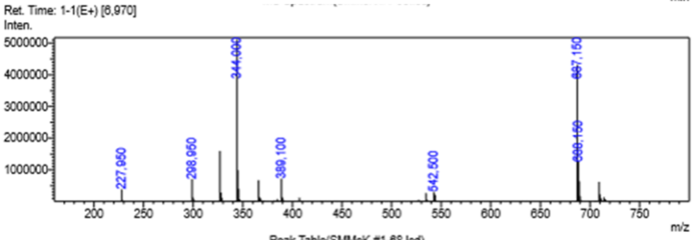

Peak Table(SMMcK-#1-68.lcd)

Detector A 254nm

Peak#	Ret. Time	Area	Height	Mark	Conc.	Area%
1	6.921	7454040	835142		97.163	97.163
2	7.408	161346	31651	M	2.103	2.103
3	7.750	56275	10713	M	0.734	0.734
Total		7671661	877507		100.000	100.000

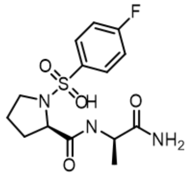

Exact Mass: 343.1002

Observed $[M+H]^+$=344,000

99

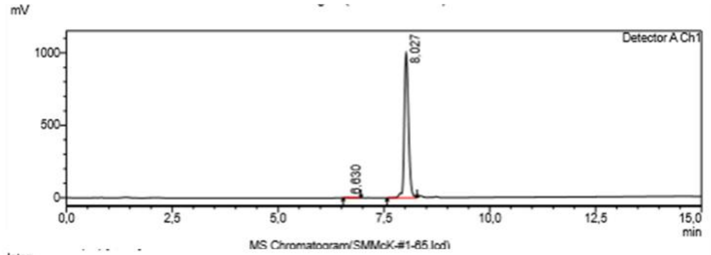

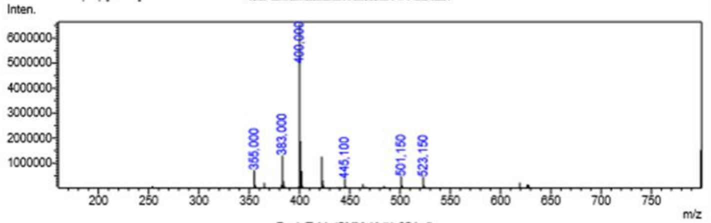

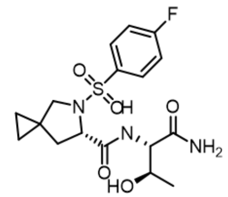

Exact Mass: 399.1264
Observed $[M+H]^+$=400,000

100

Peak Table(SMMcK-#1-65.lcd)

Detector A 254nm

Peak#	Ret. Time	Area	Height	Mark	Conc.	Area%
1	6.630	6181	754	M	0.090	0.090
2	8.027	6886361	994707	V	99.910	99.910
Total		6892542	995461		100.000	100.000

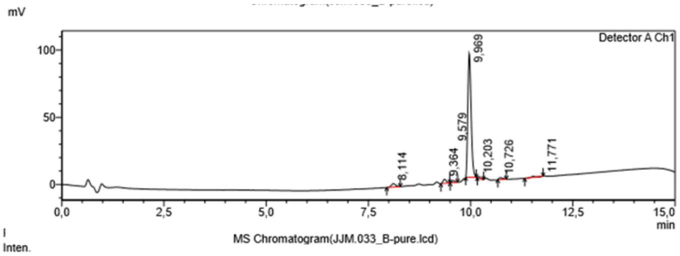

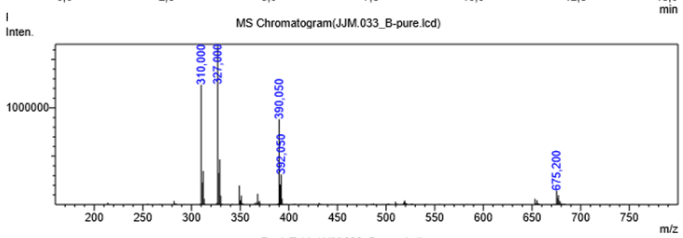

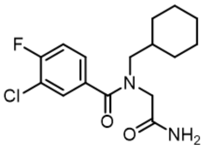

Exact Mass: 326.1197
Observed $[M+H]^+$=327,000

101

Peak Table(JJM.033_B-pure.lcd)

Detector A 254nm

Peak#	Ret. Time	Area	Height	Mark	Conc.	Area%
1	8.114	18970	2494	M	3.356	3.356
2	9.364	15450	2927	M	2.734	2.734
3	9.579	7139	1366	M	1.263	1.263
4	9.969	512529	92040	M	90.681	90.681
5	10.203	2615	1056	M	0.463	0.463
6	10.726	8634	1326	M	1.528	1.528
7	11.771	-140	-73	M	-0.025	-0.025
Total		565198	101135		100.000	100.000

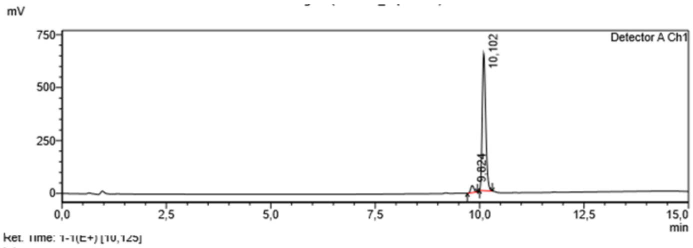

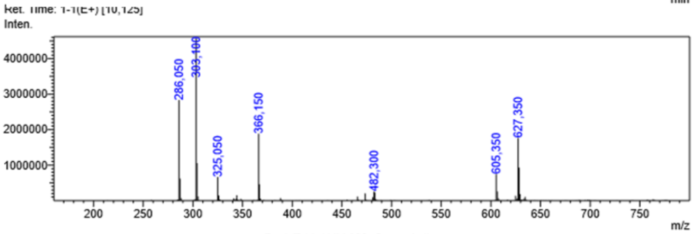

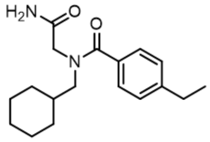

Exact Mass: 302.1994
Observed $[M+H]^+$=303,100

102

Peak Table(JJM.032_C-pure.lcd)

Detector A 254nm

Peak#	Ret. Time	Area	Height	Mark	Conc.	Area%
1	9.824	178481	33841	M	4.547	4.547
2	10.102	3746925	648304	M	95.453	95.453
Total		3925406	682145		100.000	100.000

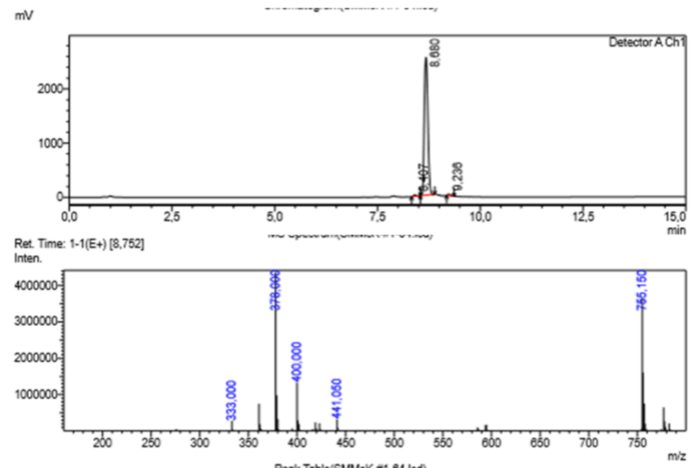

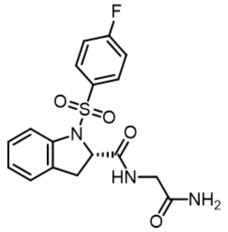

Exact Mass: 377.0846
Observed $[M+H]^+$=378,000

103

Peak Table(SMMcK-#1-64.lcd)

Detector A 254nm

Peak#	Ret. Time	Area	Height	Mark	Conc.	Area%
1	8.407	113038	26342	M	0.727	0.727
2	8.680	15304193	2539912	M	98.377	98.377
3	9.236	139413	27499	M	0.896	0.896
Total		15556644	2593754		100.000	100.000

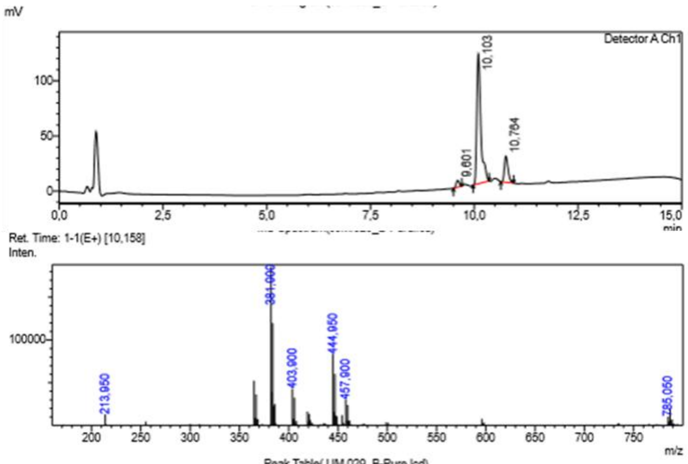

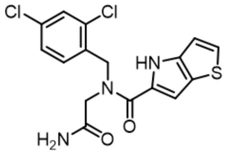

Exact Mass: 381.0106
Observed [M+H]+=381,900

104

Peak Table(JJM.029_B-Pure.lcd)

Detector A 254nm

Peak#	Ret. Time	Area	Height	Mark	Conc.	Area%
1	9.601	31577	6160	M	3.260	3.260
2	10.103	791892	117400	M	81.750	81.750
3	10.764	145208	23749	M	14.990	14.990
Total		968676	147308		100.000	100.000

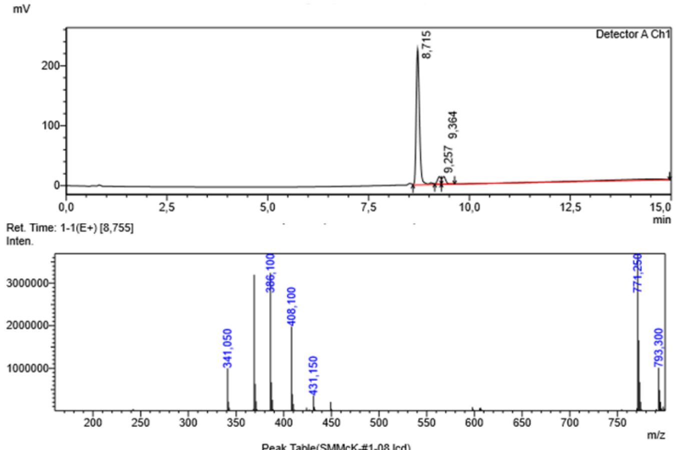

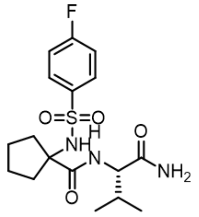

Exact Mass: 385.1472
Observed $[M+H]^+$=386,100

105

Peak Table(SMMcK-#1-08.lcd)

Detector A 254nm

Peak#	Ret. Time	Area	Height	Mark	Conc.	Area%
1	8,715	1541692	226183	S	91,427	91,427
2	9,257	72254	12241	T	4,285	4,285
3	9,364	72318	12421	TV	4,289	4,289
Total		1686263	250846		100,000	100,000

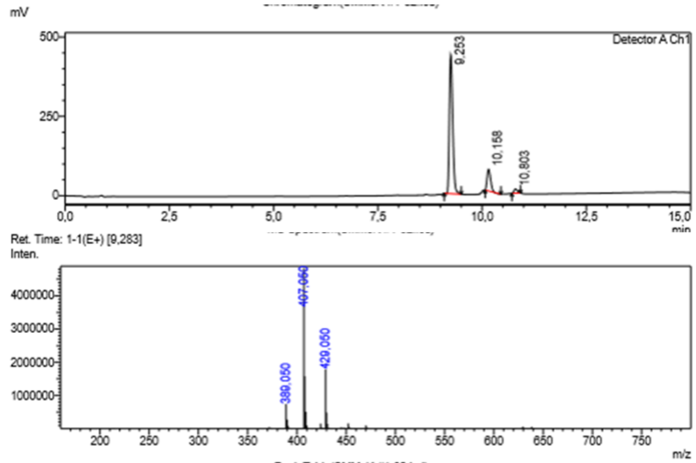

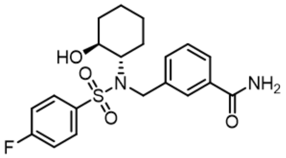

Exact Mass: 406.1363
Observed $[M+H]^+$=407,050

106

Peak Table(SMMcK-#1-32.lcd)

Detector A 254nm

Peak#	Ret. Time	Area	Height	Mark	Conc.	Area%
1	9,253	2445363	437077	M	83,794	83,794
2	10,158	396006	68478	M	13,570	13,570
3	10,803	76947	13139	M	2,637	2,637
Total		2918317	518694		100,000	100,000

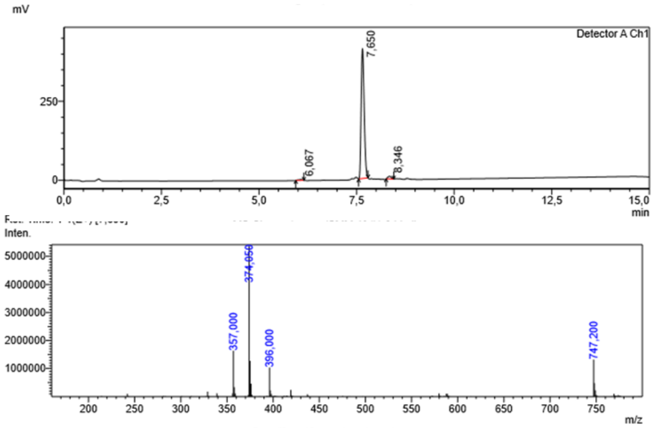

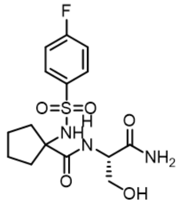

Exact Mass: 373.1108
Observed $[M+H]^+$=374,050

107

Peak Table(SMMcK-#1-04.lcd)

Detector A 254nm

Peak#	Ret. Time	Area	Height	Mark	Conc.	Area%
1	6,067	21448	2947	M	0,944	0,944
2	7,650	2199205	412851	M	96,840	96,840
3	8,346	50315	8330	M	2,216	2,216
Total		2270968	424128		100,000	100,000

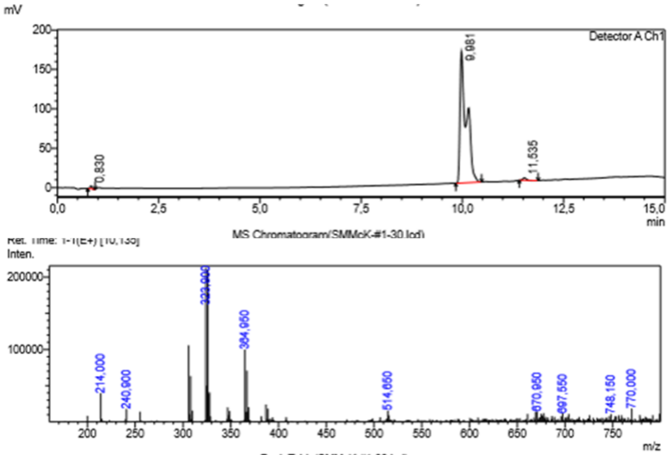

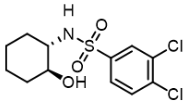

Exact Mass: 323.0150
Observed $[M+H]^+$=323,900

108

Peak Table(SMMcK-#1-30.lcd)

Detector A 254nm

Peak#	Ret. Time	Area	Height	Mark	Conc.	Area%
1	0,830	11554	3549	M	0,609	0,609
2	9,981	1873249	167137	M	98,708	98,708
3	11,535	12962	2980	M	0,683	0,683
Total		1897765	173666		100,000	100,000

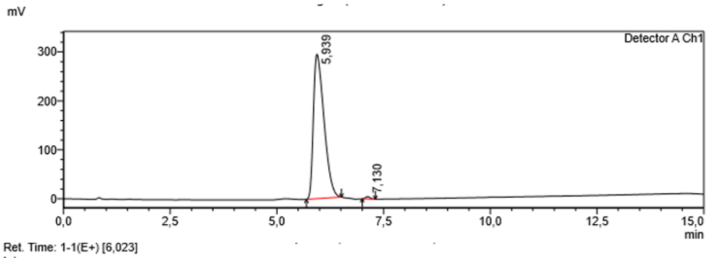

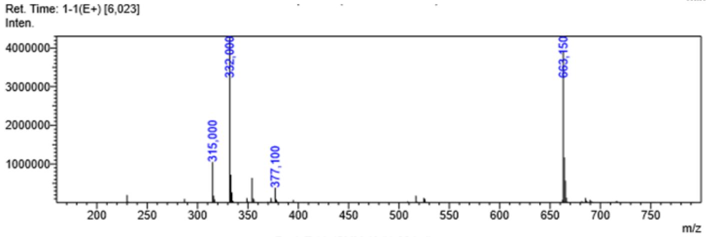

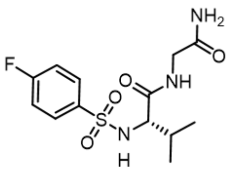

Exact Mass: 331.1002
Observed $[M+H]^+$=332,000

109

Peak Table(SMMcK-#1-09.lcd)

Detector A 254nm

Peak#	Ret. Time	Area	Height	Mark	Conc.	Area%
1	5,939	5135213	295236	M	99,338	99,338
2	7,130	34234	4568	M	0,662	0,662
Total		5169448	299804		100,000	100,000

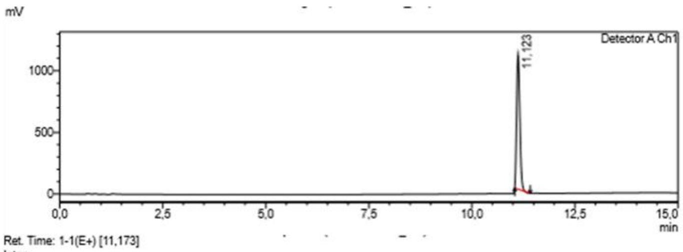

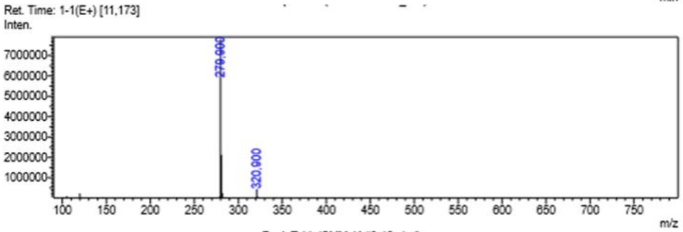

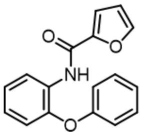

Exact Mass: 279.0895
Observed $[M+H]^+$=279,900

110

Peak Table(SMMcK-#3-18_.lcd)

Detector A 254nm

Peak#	Ret. Time	Area	Height	Mark	Conc.	Area%
1	11,123	5900067	1097380	M	100,000	100,000
Total		5900067	1097380		100,000	100,000

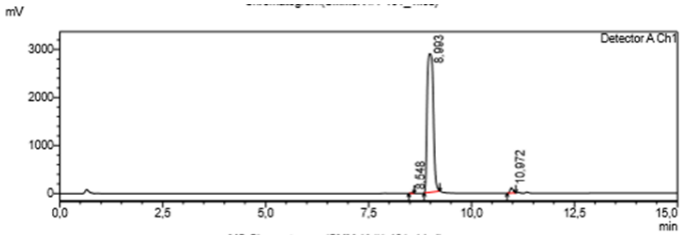

MS Chromatogram(SMMcK-#1-101_1.lcd)

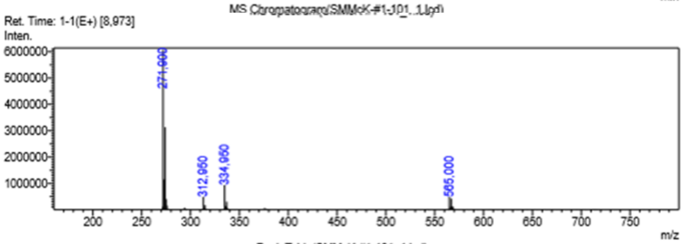

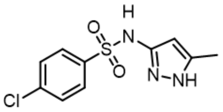

Exact Mass: 271.0182
[M+H]+=271,900

111

Peak Table(SMMcK-#1-101_1.lcd)

Detector A 254nm

Peak#	Ret. Time	Area	Height	Mark	Conc.	Area%
1	8,548	41054	9064	M	0,134	0,134
2	8,993	30135604	2887330	M	98,064	98,064
3	10,972	553948	110837	M	1,803	1,803
Total		30730606	3007230		100,000	100,000

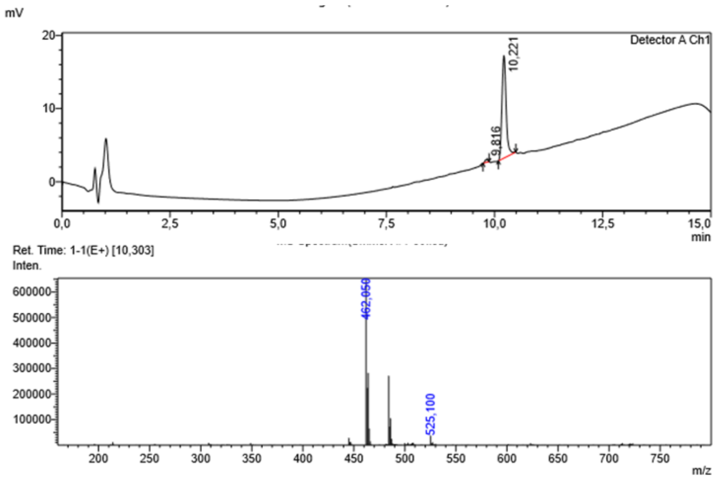

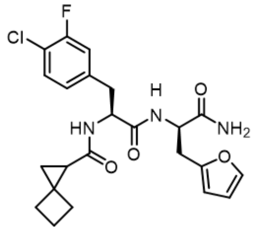

Exact Mass: 461.1518
Observed $[M+H]^+$=462,050

112

Peak Table(SMMcK-#1-80.lcd)

Detector A 254nm

Peak#	Ret. Time	Area	Height	Mark	Conc.	Area%
1	9.816	1822	386	M	1.896	1.896
2	10.221	94253	13966	M	98.104	98.104
Total		96075	14352		100.000	100.000

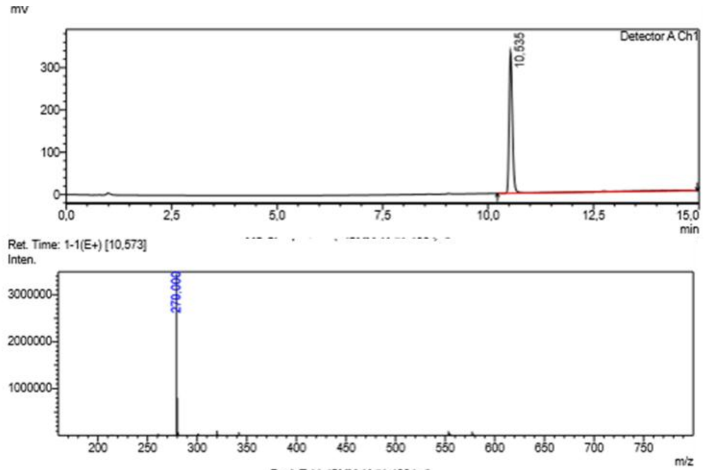

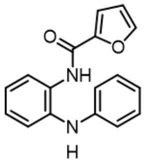

Exact Mass: 278.1055
Observed $[M+H]^+$=279,000

113

Peak Table(SMMcK-#1-130.lcd)

Detector A 254nm

Peak#	Ret. Time	Area	Height	Mark	Conc.	Area%
1	10.535	2039068	334013	S	100.000	100.000
Total		2039068	334013		100.000	100.000

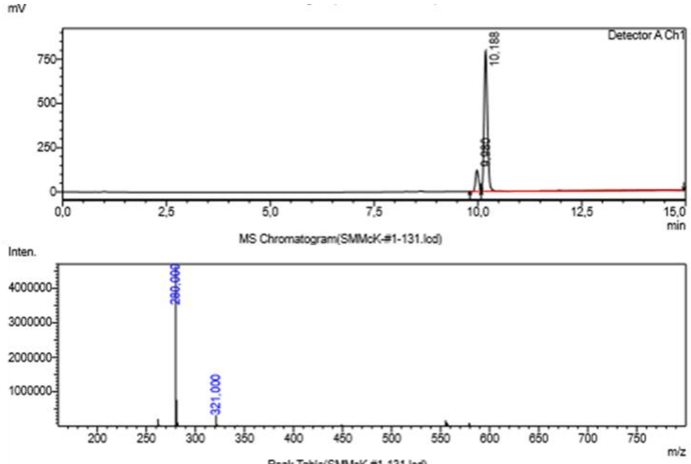

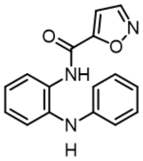

Exact Mass: 279.1008
Observed $[M+H]^+$=280,000

114

Peak Table(SMMcK-#1-131.lcd)

Detector A 254nm

Peak#	Ret. Time	Area	Height	Mark	Conc.	Area%
1	9.980	651942	120142		12.072	12.072
2	10.188	4748714	795803	V	87.928	87.928
Total		5400656	915945		100.000	100.000

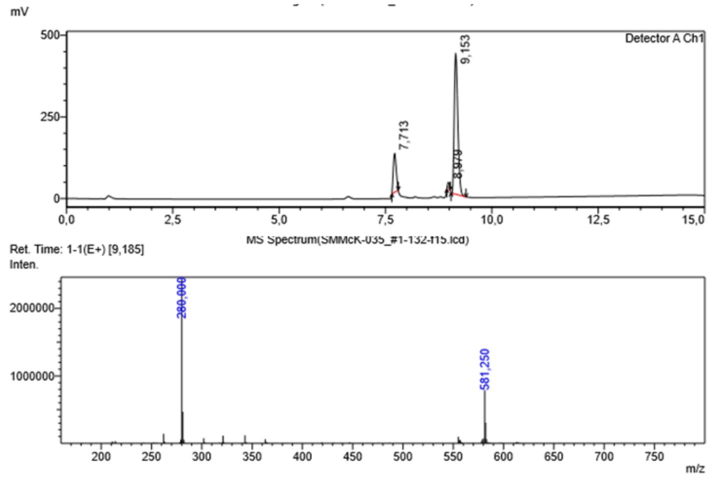

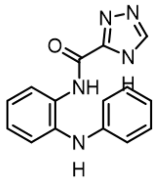

Exact Mass: 279.1120
Observed $[M+H]^+$=280,000
115

Peak Table(SMMcK-035_#1-132-f15.lcd)

Detector A 254nm

Peak#	Ret. Time	Area	Height	Mark	Conc.	Area%
1	7.713	572915	118479	M	18.069	18.069
2	8.979	68817	22237	M	2.170	2.170
3	9.153	2528948	429522	M	79.760	79.760
Total		3170680	570237		100.000	100.000

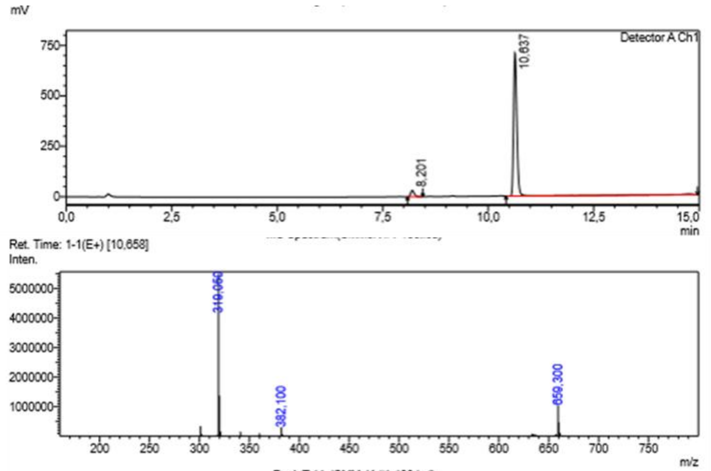

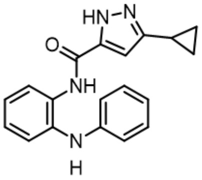

Exact Mass: 318.1481
Observed $[M+H]^+$=319,050
116

Peak Table(SMMcK-#1-133.lcd)

Detector A 254nm

Peak#	Ret. Time	Area	Height	Mark	Conc.	Area%
1	8.201	183556	31089		4.367	4.367
2	10.637	4019813	708097	S	95.633	95.633
Total		4203369	739186		100.000	100.000

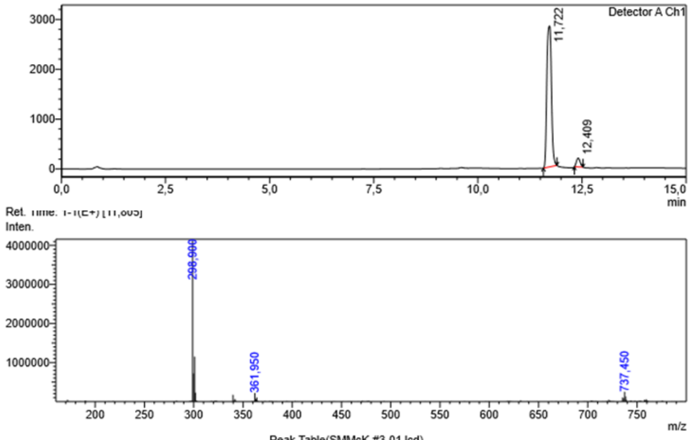

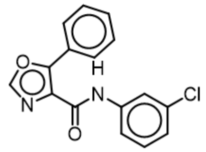

Exact Mass: 298.0509
Observed $[M+H]^+$=298,900

117

Peak Table(SMMcK-#3-01.lcd)

Detector A 254nm

Peak#	Ret. Time	Area	Height	Mark	Conc.	Area%
1	11.722	21827115	2821994	M	95.213	95.213
2	12.409	1097388	176445	M	4.787	4.787
Total		22924503	2998439		100.000	100.000

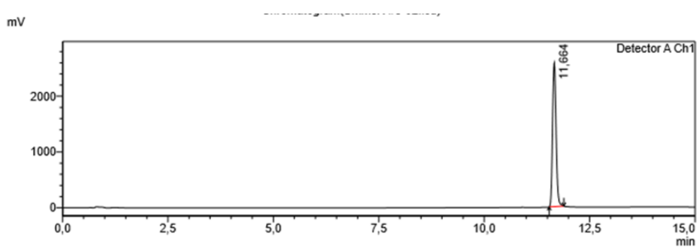

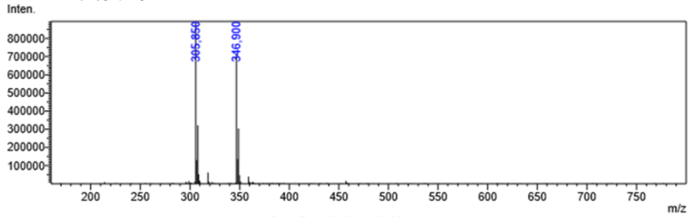

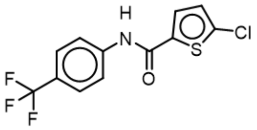

Exact Mass: 304.9889

Observed $[M+H]^{+}$=305,850

118

Peak Table(SMMcK-#3-02.lcd)

Detector A 254nm

Peak#	Ret. Time	Area	Height	Mark	Conc.	Area%
1	11,664	15115639	2578731	M	100,000	100,000
Total		15115639	2578731		100,000	100,000

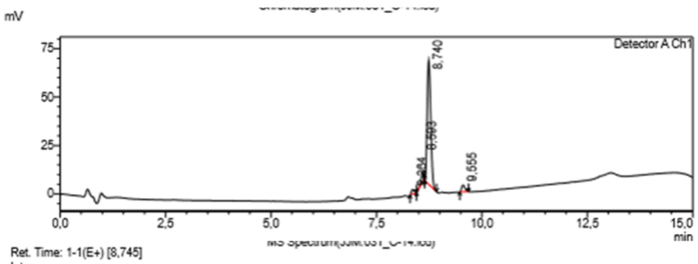

Ret. Time: 1-1(E+) [8,745]

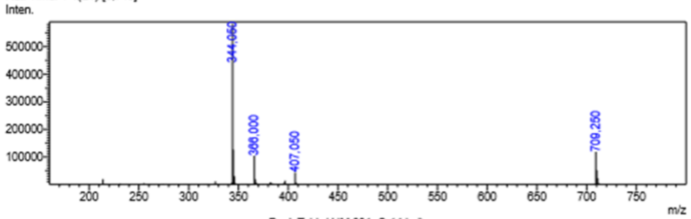

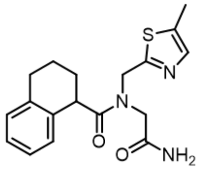

Exact Mass: 343.1354

Observed $[M+H]^{+}$=344,050

119

Peak Table(JJM.031_C-14.lcd)

Detector A 254nm

Peak#	Ret. Time	Area	Height	Mark	Conc.	Area%
1	8,364	10715	2355	M	2,529	2,529
2	8,593	22740	5141	M	5,368	5,368
3	8,740	370505	64062	M	87,462	87,462
4	9,555	19660	3529	M	4,641	4,641
Total		423621	75087		100,000	100,000

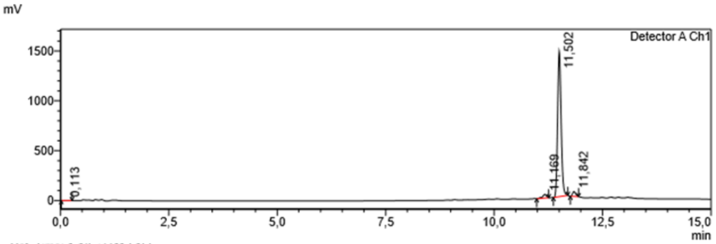

Ret. Time: 1-1(E+) [11,515]

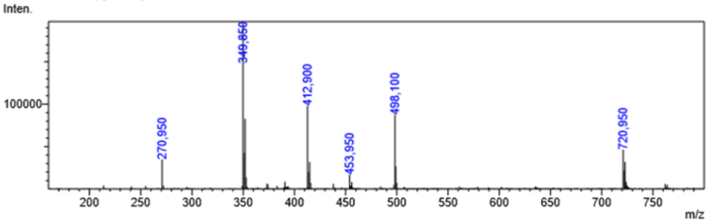

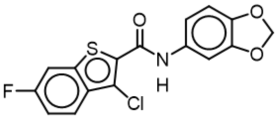

Exact Mass: 348.9976

Observed $[M+H]^{+}$=349,050

120

Peak Table(SMMcK-#3-06.lcd)

Detector A 254nm

Peak#	Ret. Time	Area	Height	Mark	Conc.	Area%
1	0,113	4398	543		0,051	0,051
2	11,169	223738	35916	M	2,578	2,578
3	11,502	8204878	1457517	M	94,542	94,542
4	11,842	245581	47993	M	2,830	2,830
Total		8678595	1541970		100,000	100,000

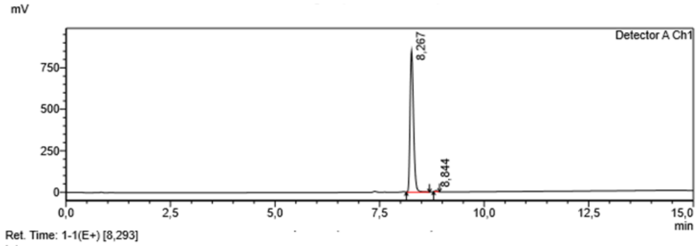

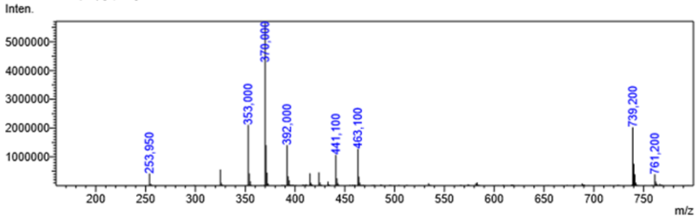

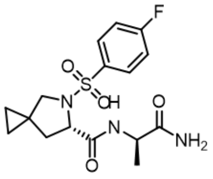

Exact Mass: 369.1159

Observed $[M+H]^+$=370,000

121

Peak Table(SMMcK-#1-67.lcd)

Detector A 254nm

Peak#	Ret. Time	Area	Height	Mark	Conc.	Area%
1	8,267	4949356	852935		99,436	99,436
2	8,844	28084	6350	M	0,564	0,564
Total		4977439	859285		100,000	100,000

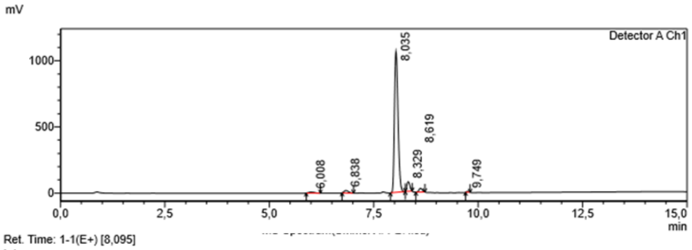

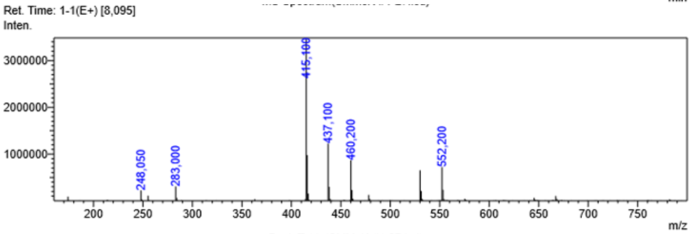

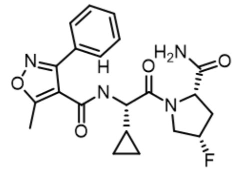

Exact Mass: 414.1703

Observed $[M+H]^+$= 415,100

122

Peak Table(SMMcK-#1-27.lcd)

Detector A 254nm

Peak#	Ret. Time	Area	Height	Mark	Conc.	Area%
1	6,008	71480	7107	M	1,002	1,002
2	6,838	136029	18770	M	1,907	1,907
3	8,035	6368134	1067436	M	89,274	89,274
4	8,329	375981	72939	M	5,271	5,271
5	8,619	155298	27751	M	2,177	2,177
6	9,749	26299	6874	M	0,369	0,369
Total		7133221	1200876		100,000	100,000

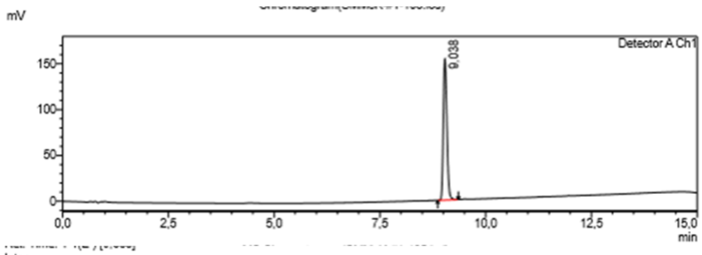

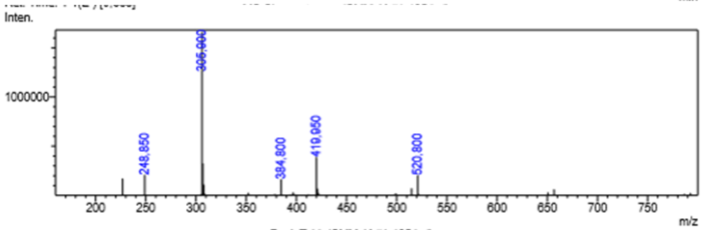

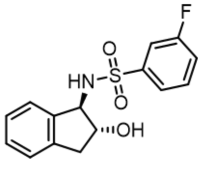

Exact Mass: 307.0678

Observed $[M-H]^-$=306,000

123

Peak Table(SMMcK-#1-105.lcd)

Detector A 254nm

Peak#	Ret. Time	Area	Height	Mark	Conc.	Area%
1	9,038	904020	153962		100,000	100,000
Total		904020	153962		100,000	100,000

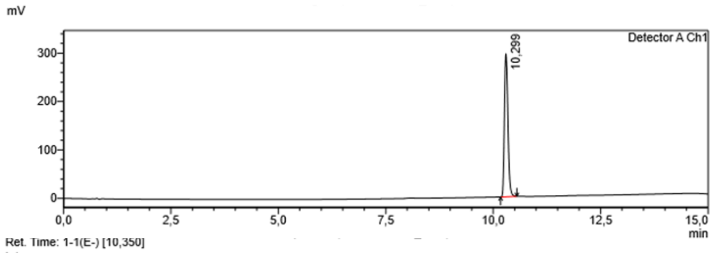

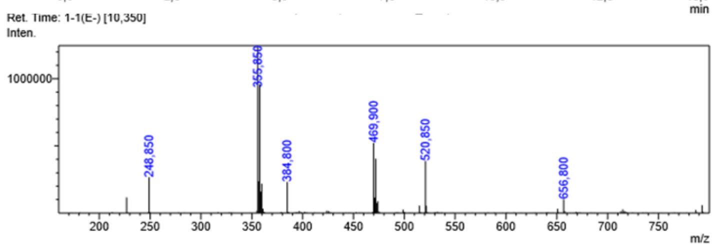

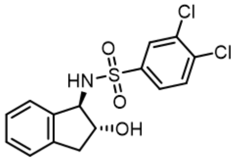

Exact Mass: 356.9993

Observed $[M+H]^+$=357,850

124

Peak Table(SMMcK-#1-45_1.lcd)

Detector A 254nm

Peak#	Ret. Time	Area	Height	Mark	Conc.	Area%
1	10,299	1612202	296148	M	100,000	100,000
Total		1612202	296148		100,000	100,000

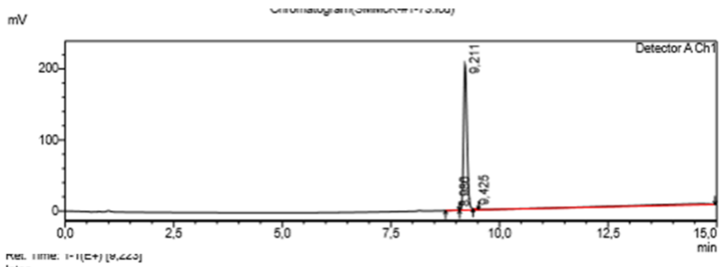

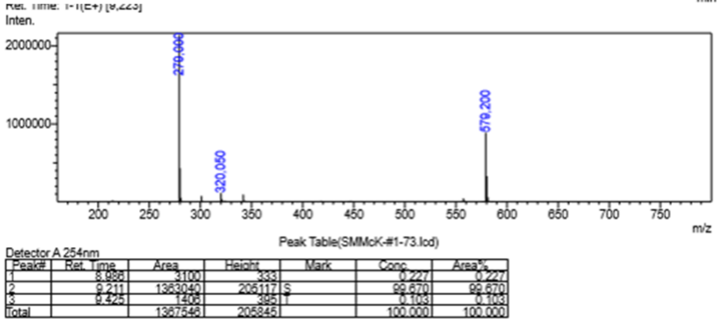

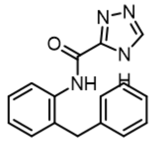

Exact Mass: 278.1168

Observed $[M+H]^+$=279,000

125

Peak Table(SMMcK-#1-73.lcd)

Detector A 254nm

Peak#	Ret. Time	Area	Height	Mark	Conc.	Area%
1	8,986	3100	333		0,227	0,227
2	9,211	1363040	205117	S	99,670	99,670
3	9,425	1406	395	T	0,103	0,103
Total		1367546	205845		100,000	100,000

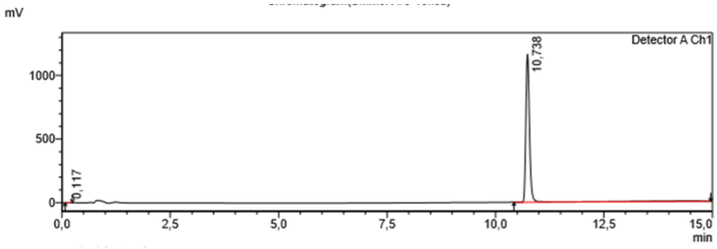

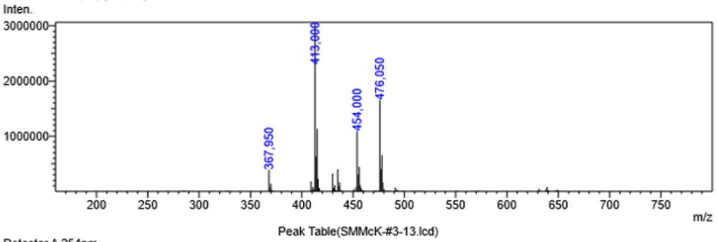

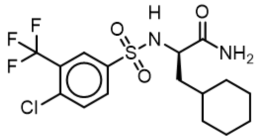

Exact Mass: 412.0835

Observed $[M+H]^+$=413,000

126

Peak Table(SMMcK-#3-13.lcd)

Detector A 254nm

Peak#	Ret. Time	Area	Height	Mark	Conc.	Area%
1	0,117	1085	248		0,015	0,015
2	10,738	7345896	1161897	S	99,985	99,985
Total		7346981	1162145		100,000	100,000

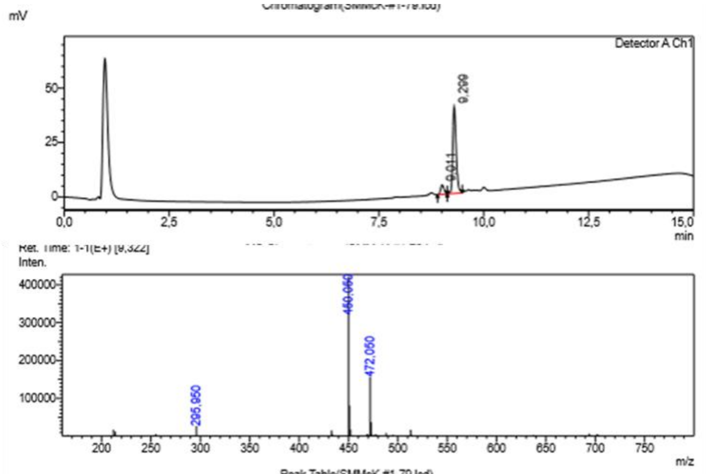

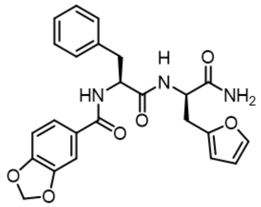

Exact Mass: 449.1587
Observed $[M+H]^+$=450,050

127

Peak Table(SMMcK-#1-79.lcd)

Detector A 254nm

Peak#	Ret. Time	Area	Height	Mark	Conc.	Area%
1	9.011	25049	4395		10.012	10.012
2	9.299	225150	39842	V	89.988	89.988
Total		250199	44238		100.000	100.000

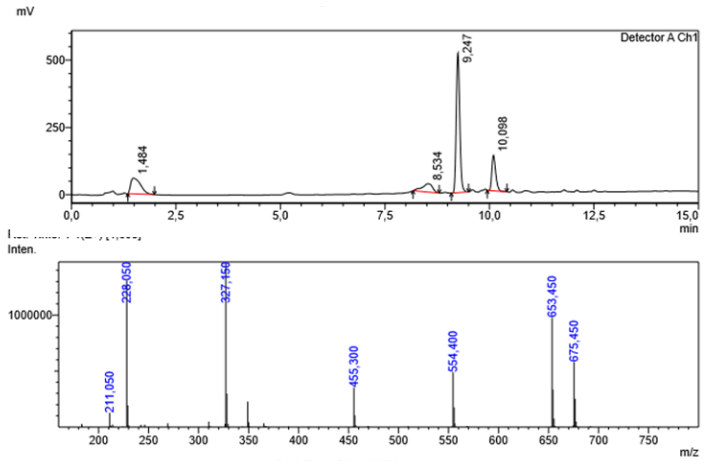

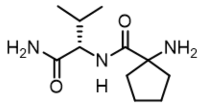

Exact Mass: 227,1634
Observed $[M+H]^+$=228,050

128

Peak Table(SMMcK-020-1.lcd)

Detector A 254nm

Peak#	Ret. Time	Area	Height	Mark	Conc.	Area%
1	1.484	962066	58470	M	17.779	17.779
2	8.534	527887	30702	M	9.756	9.756
3	9.247	3067910	519043	M	56.696	56.696
4	10.098	853289	131886	M	15.769	15.769
Total		5411153	740101		100.000	100.000

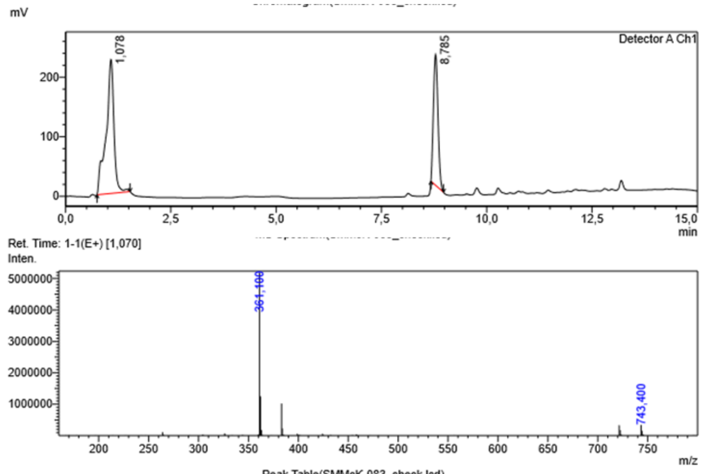

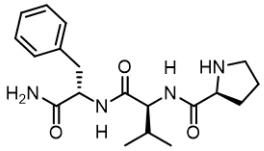

Exact Mass: 360,2161
Observed $[M+H]^+$=361,100

129

Peak Table(SMMcK-083_check.lcd)

Detector A 254nm

Peak#	Ret. Time	Area	Height	Mark	Conc.	Area%
1	1.078	2913494	224972	M	63.710	63.710
2	8.785	1659531	219677	M	36.290	36.290
Total		4573025	444649		100.000	100.000

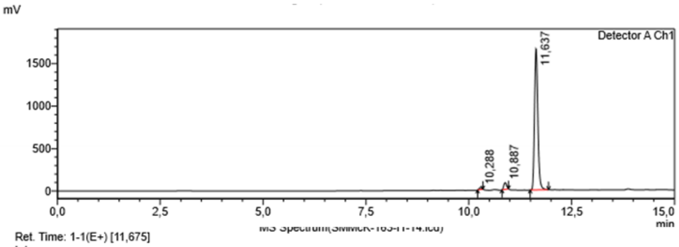

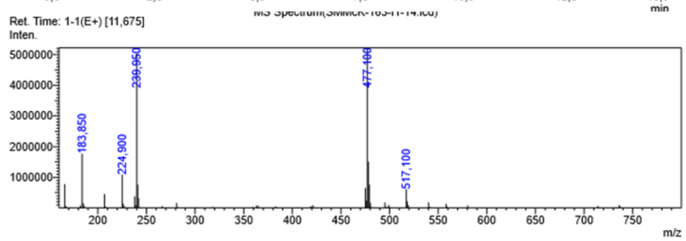

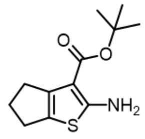

Exact Mass: 239.0980

Observed $[M+H]^+$=239,950

130

Peak Table(SMMcK-163-f1-14.lcd)

Detector A 254nm

Peak#	Ret. Time	Area	Height	Mark	Conc.	Area%
1	10,288	99696	22969	M	1,012	1,012
2	10,887	386573	79634	M	3,923	3,923
3	11,637	9367283	1660700	M	95,065	95,065
Total		9853551	1763303		100,000	100,000

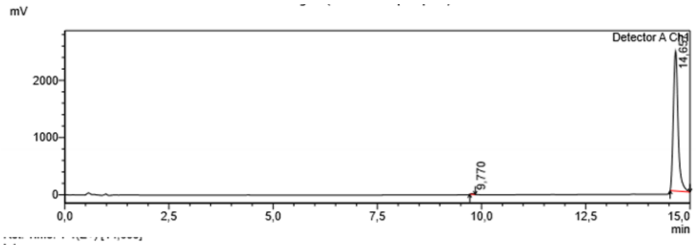

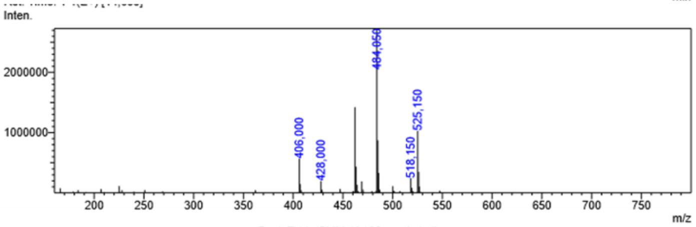

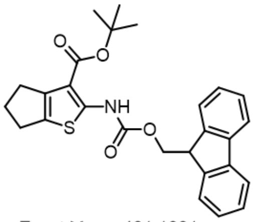

Exact Mass: 461.1661

Observed $[M+H]^+$=462,050

131

Peak Table(SMMcK-166-precip.lcd)

Detector A 254nm

Peak#	Ret. Time	Area	Height	Mark	Conc.	Area%
1	9,770	64604	14383	M	0,334	0,334
2	14,657	19304283	2445009	M	99,666	99,666
Total		19368887	2459392		100,000	100,000

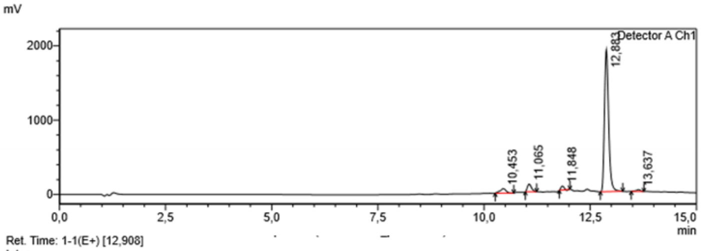

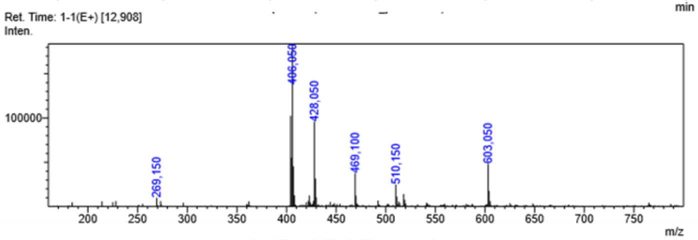

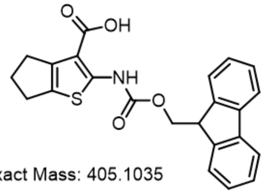

Exact Mass: 405.1035

Observed $[M+H]^+$=406,050

132

Peak Table(SMMcK-167_product.lcd)

Detector A 254nm

Peak#	Ret. Time	Area	Height	Mark	Conc.	Area%
1	10,453	577992	60629	M	3,909	3,909
2	11,065	703684	104256	M	4,759	4,759
3	11,848	234957	52906	M	1,589	1,589
4	12,883	13084995	1914109	M	88,499	88,499
5	13,637	183910	24889	M	1,244	1,244
Total		14785538	2156790		100,000	100,000

NMR Data for compound 4-16, 36, 44 and 130-132

4 – [1]H

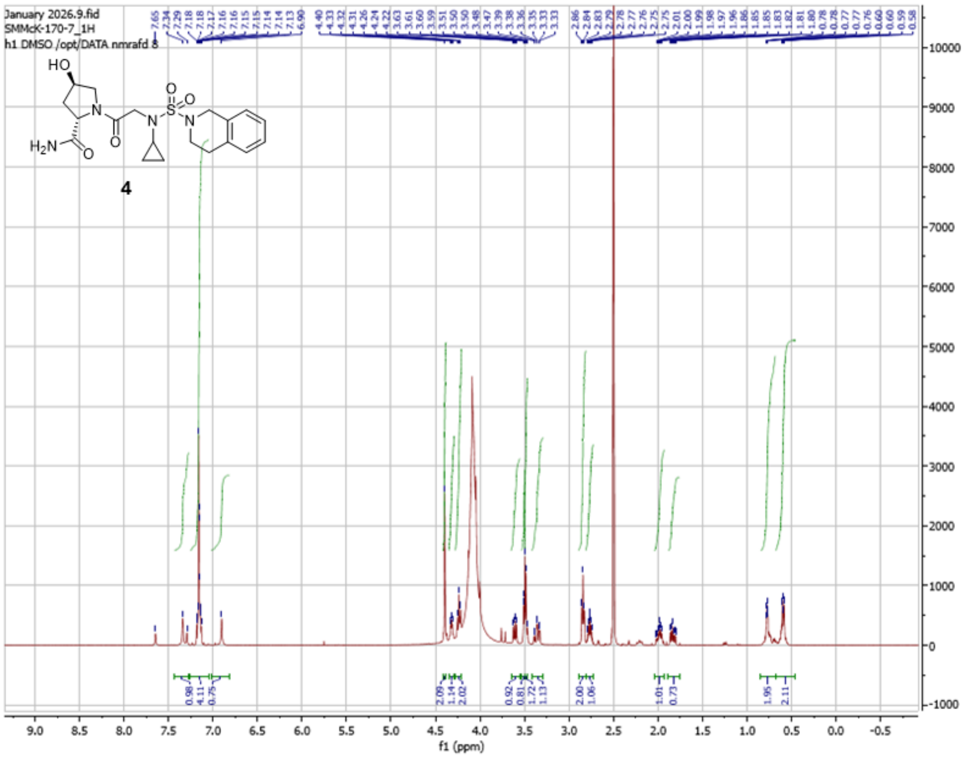

5 – [1]H

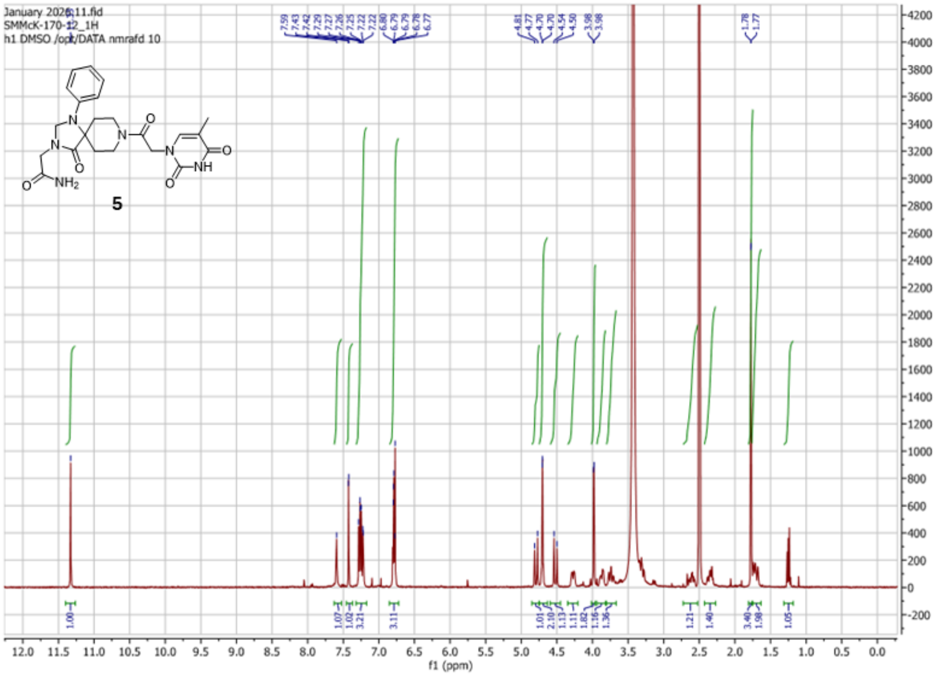

6 – ^{1}H

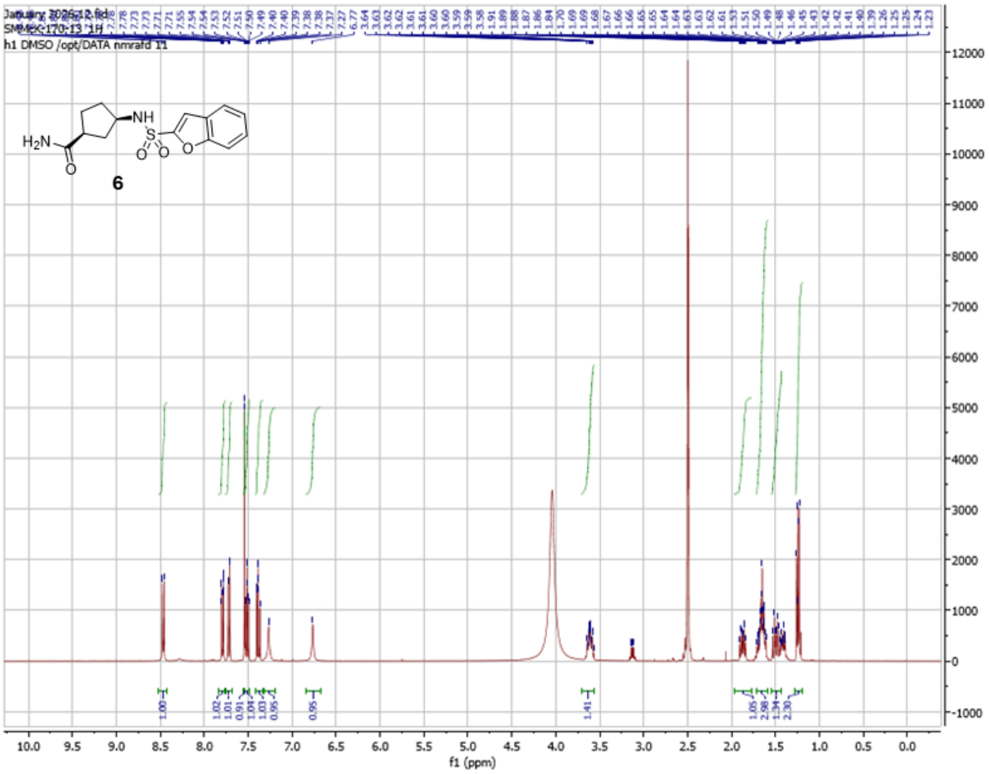

h1 DMSO /opt/DATA nmrafd 11
6
f1 (ppm)

7 – ^{1}H

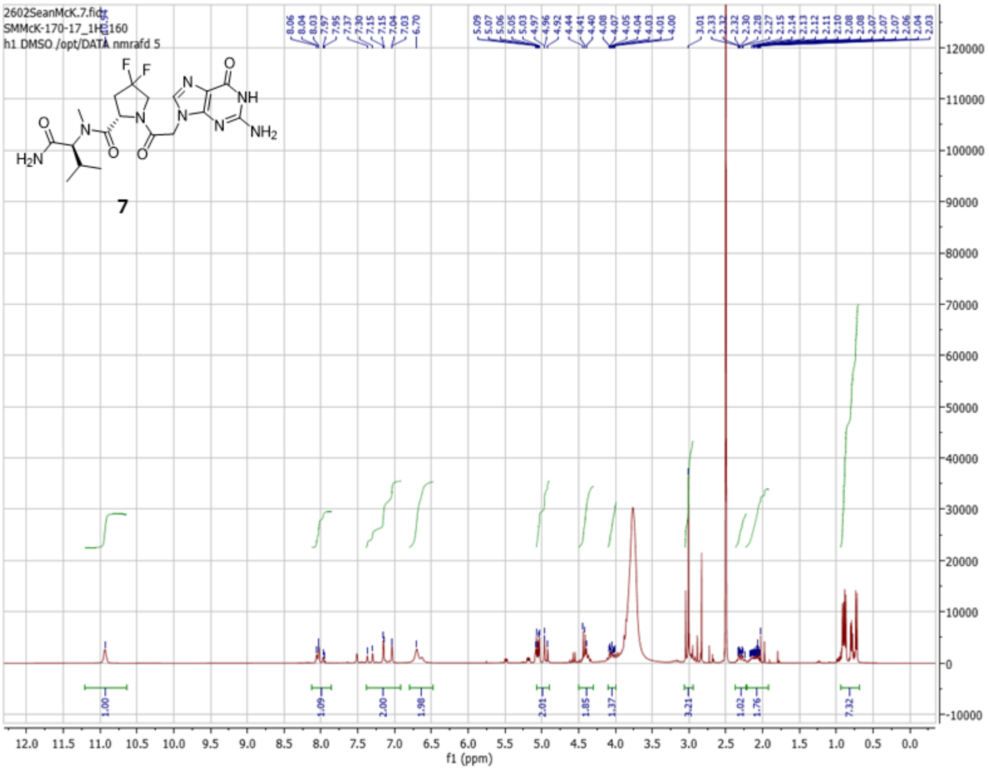

2602SeanMcK.7.fid
SMMcK-170-17_1H_160
h1 DMSO /opt/DATA nmrafd 5
7
f1 (ppm)

8 – [1]H

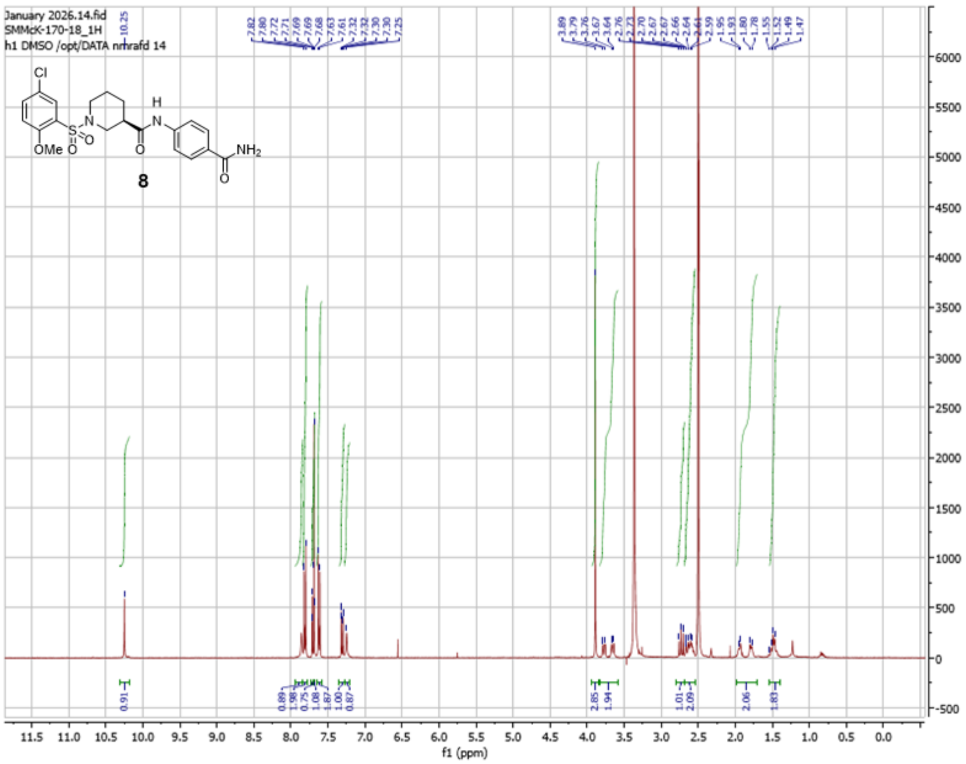
January 2026.14.fid
SMMcK-170-18_1H
h1 DMSO /opt/DATA nmrafd 14
8
f1 (ppm)

9 – [1]H

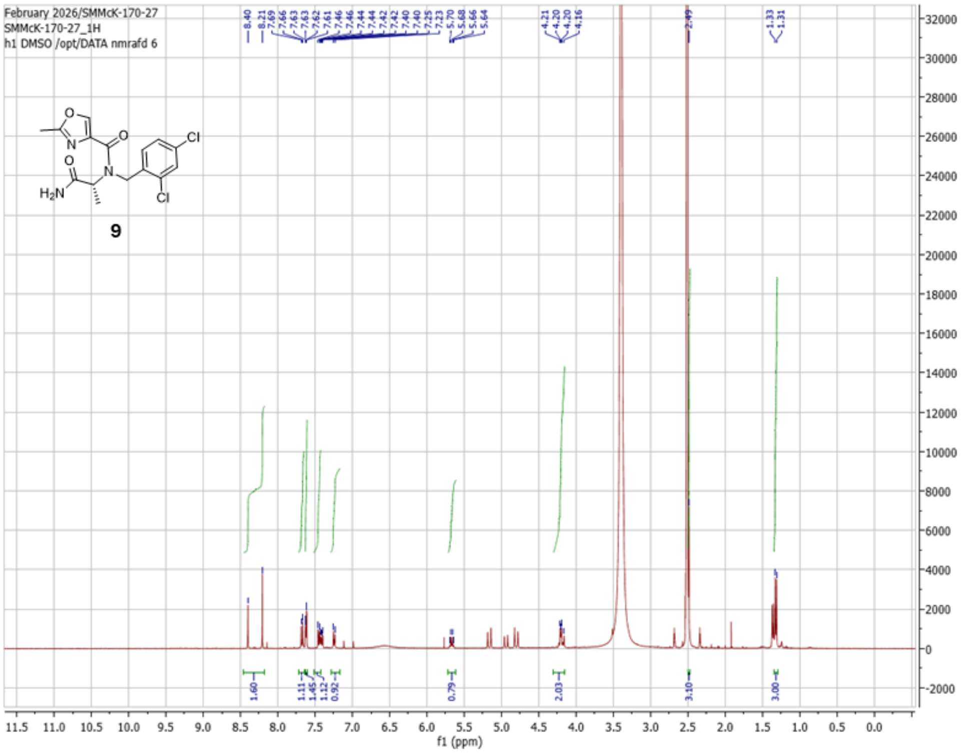
February 2026/SMMcK-170-27
SMMcK-170-27_1H
h1 DMSO /opt/DATA nmrafd 6
9
f1 (ppm)

10 – ¹H

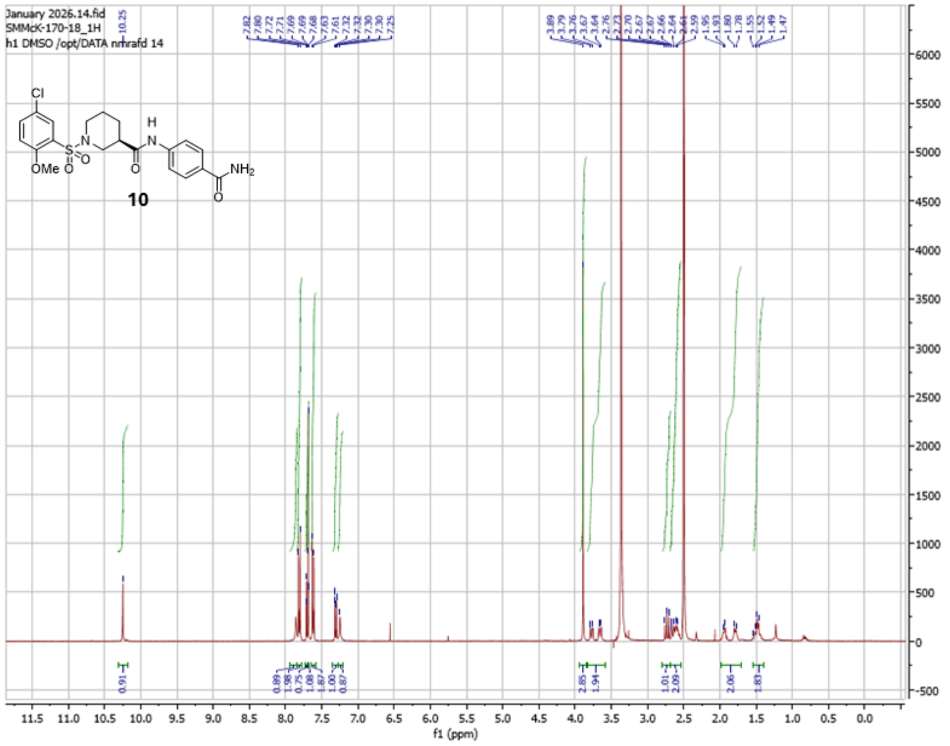

January 2026.14.fid
SMMcK-170-18_1H
h1 DMSO /opt/DATA nmrafd 14
Cl
H
N
S
O
OMe
NH2
10
f1 (ppm)

11 – ¹H

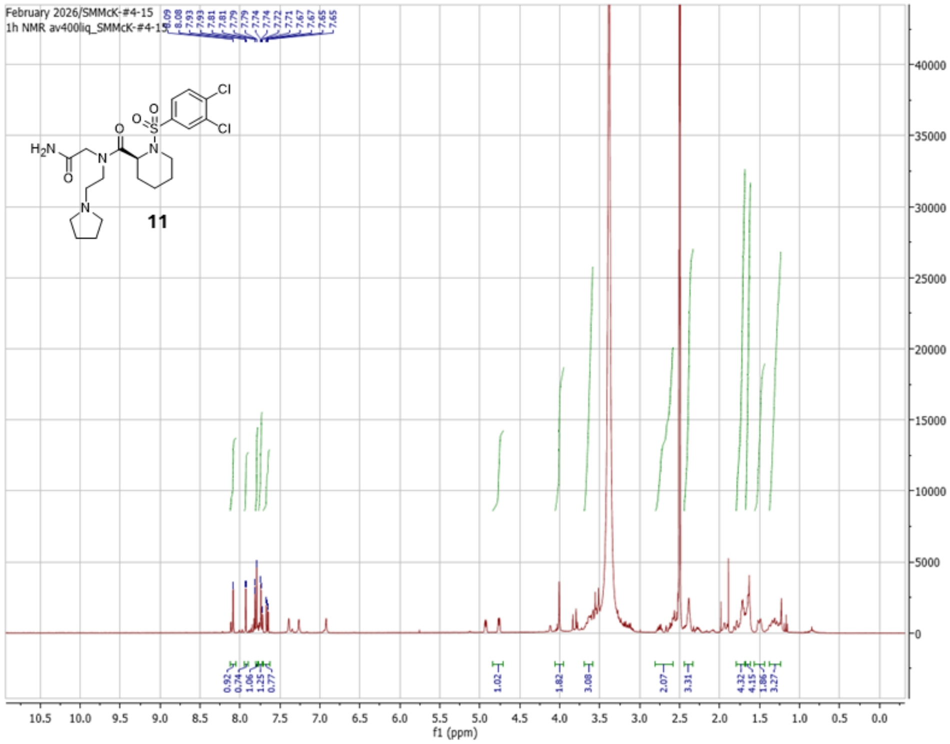

February 2026/SMMcK-#4-15
1h NMR av400liq_SMMcK-#4-15
Cl
H2N
N
O
S
11
f1 (ppm)

12 – [1]H

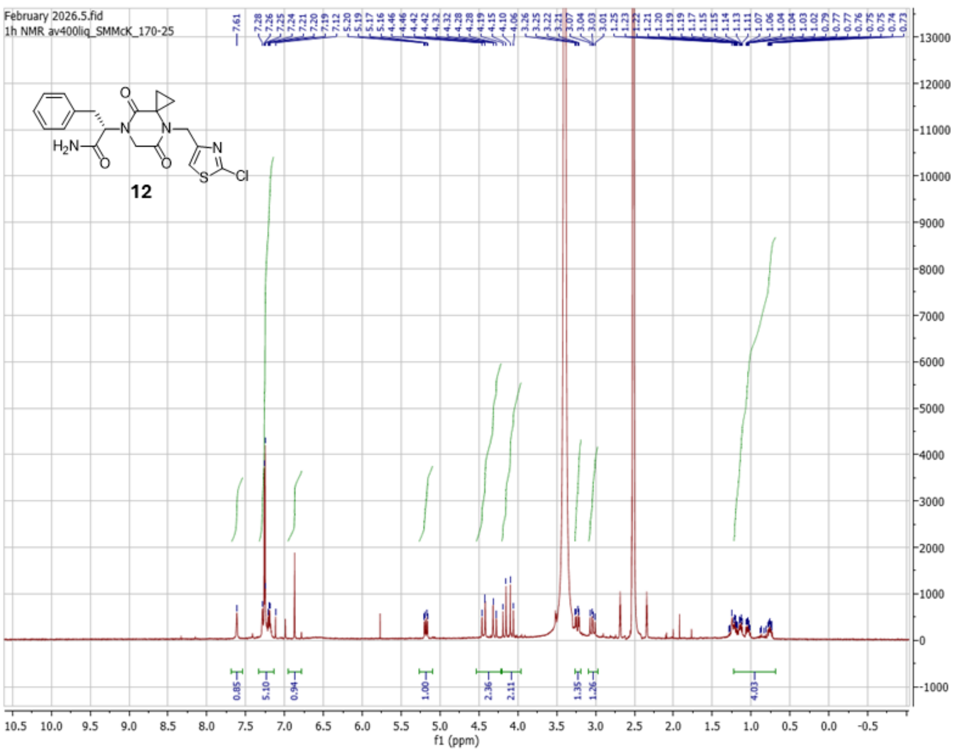

13 – [1]H

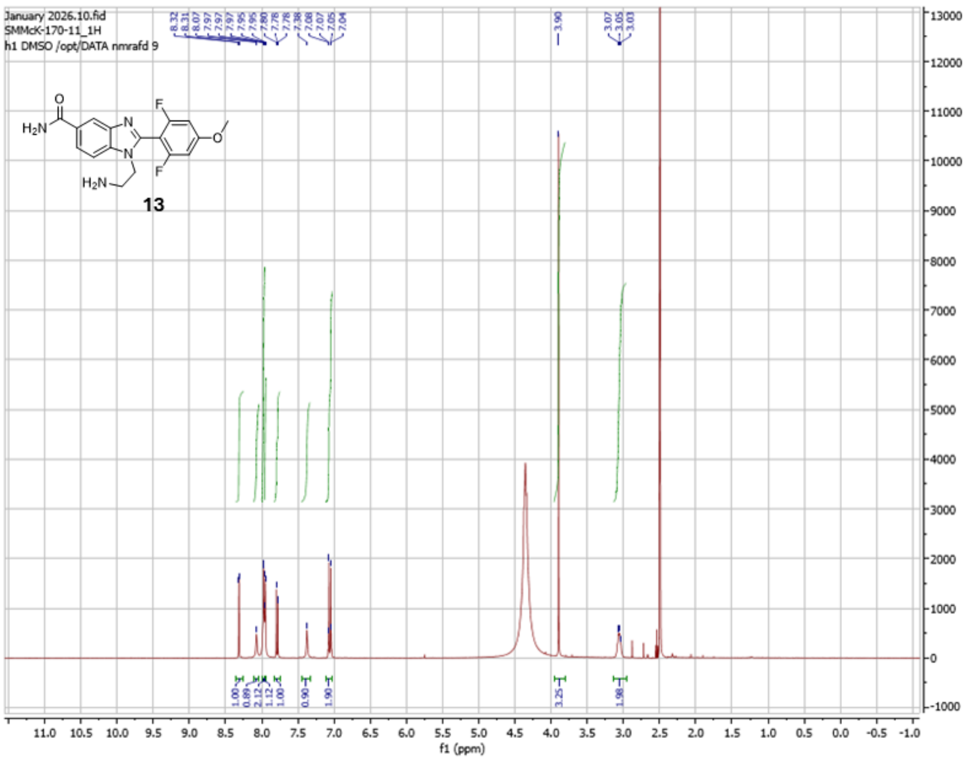

14 – [1]H

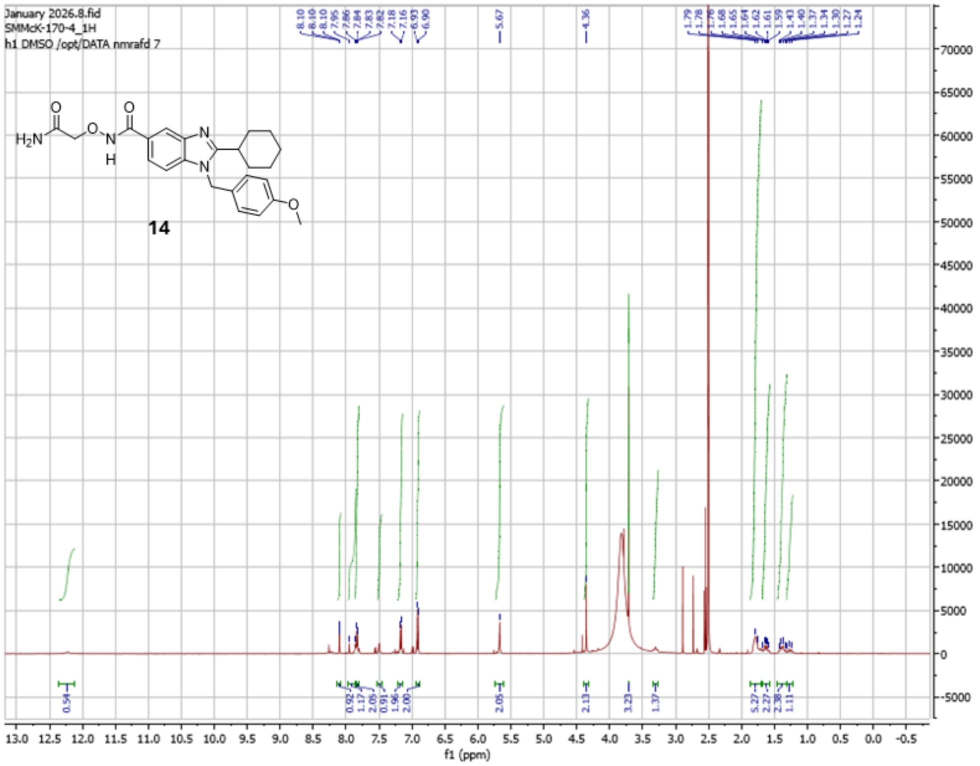

15 – [1]H

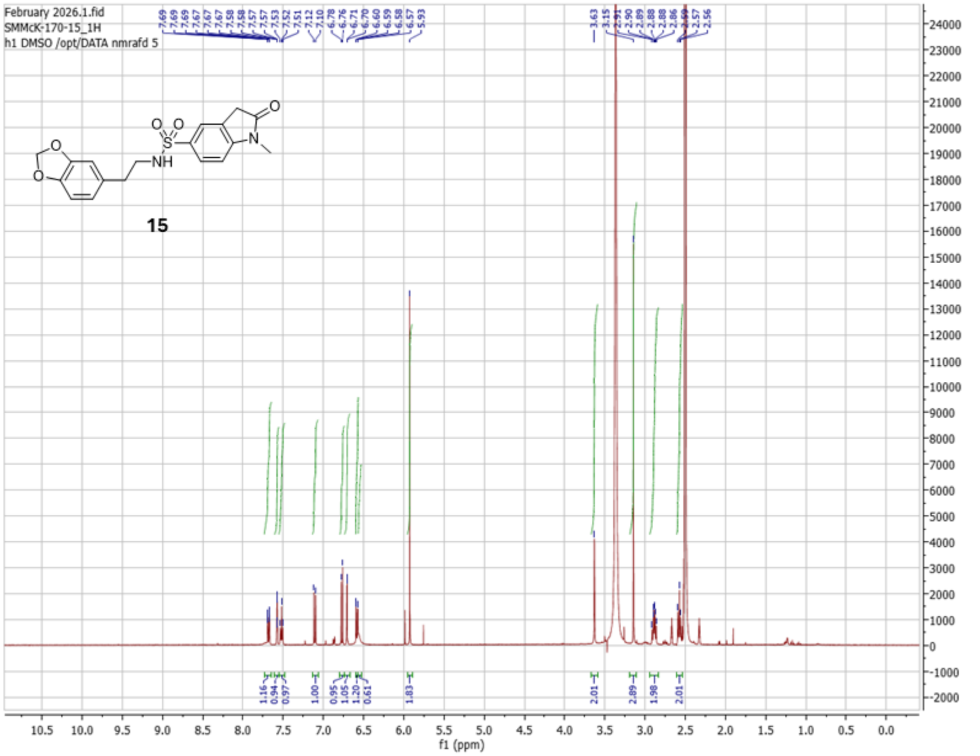

16 – [1]H

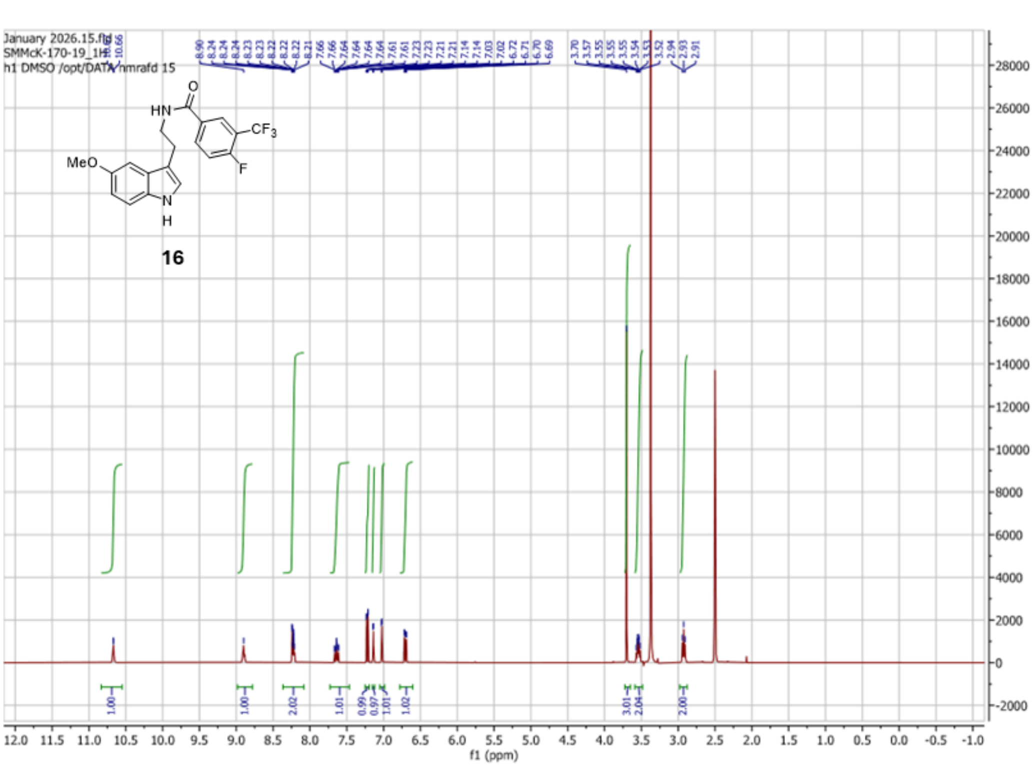

36 – ^{1}H

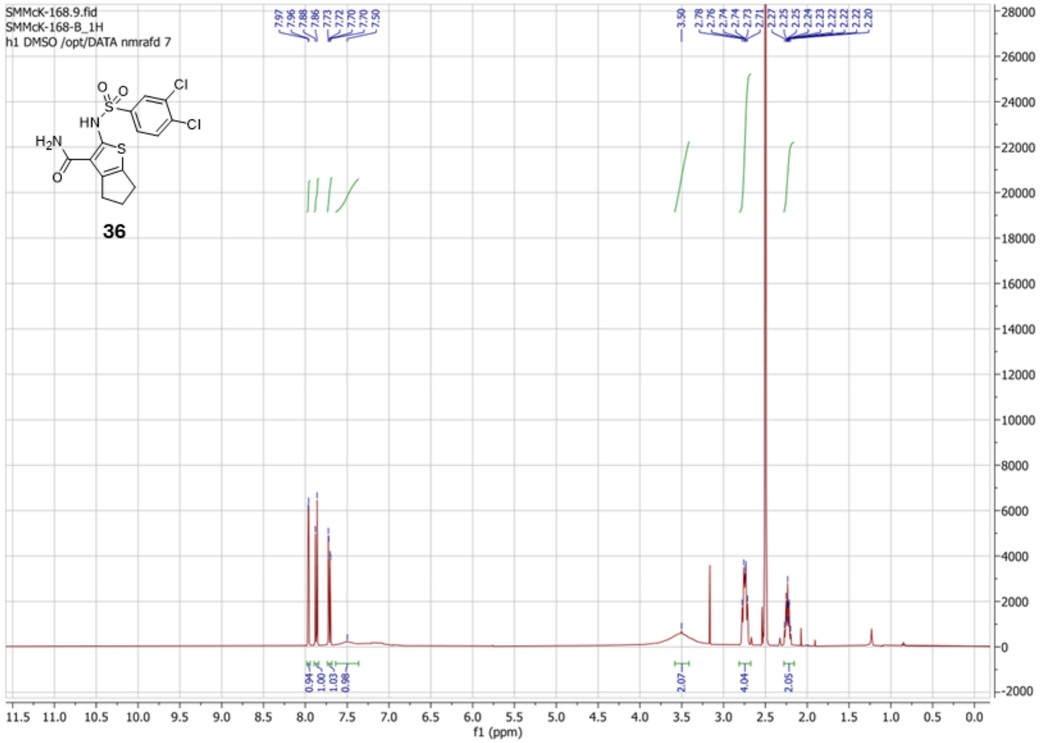
SMMcK-168.9.fid
SMMcK-168-B_1H
h1 DMSO /opt/DATA nmrafd 7
36
f1 (ppm)

36 – ^{13}C

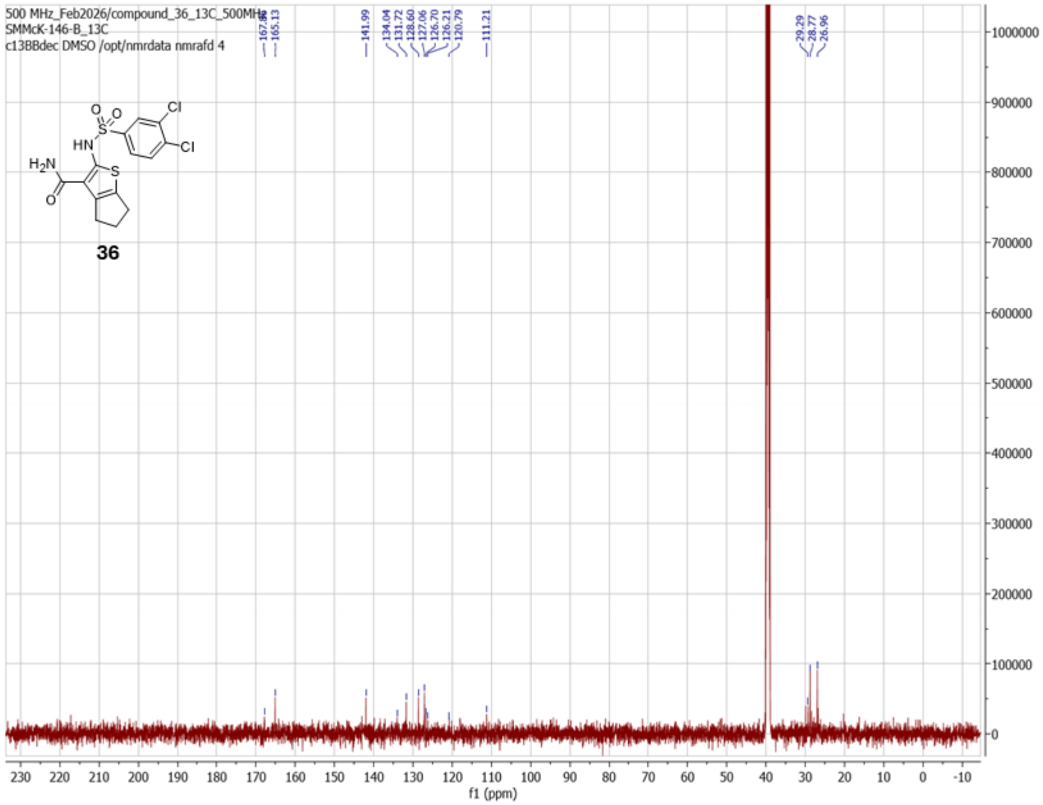
500 MHz_Feb2026/compound_36_13C_500MHz
SMMcK-146-B_13C
c13BBdec DMSO /opt/nmrdata nmrafd 4
36
f1 (ppm)

44 – ¹H

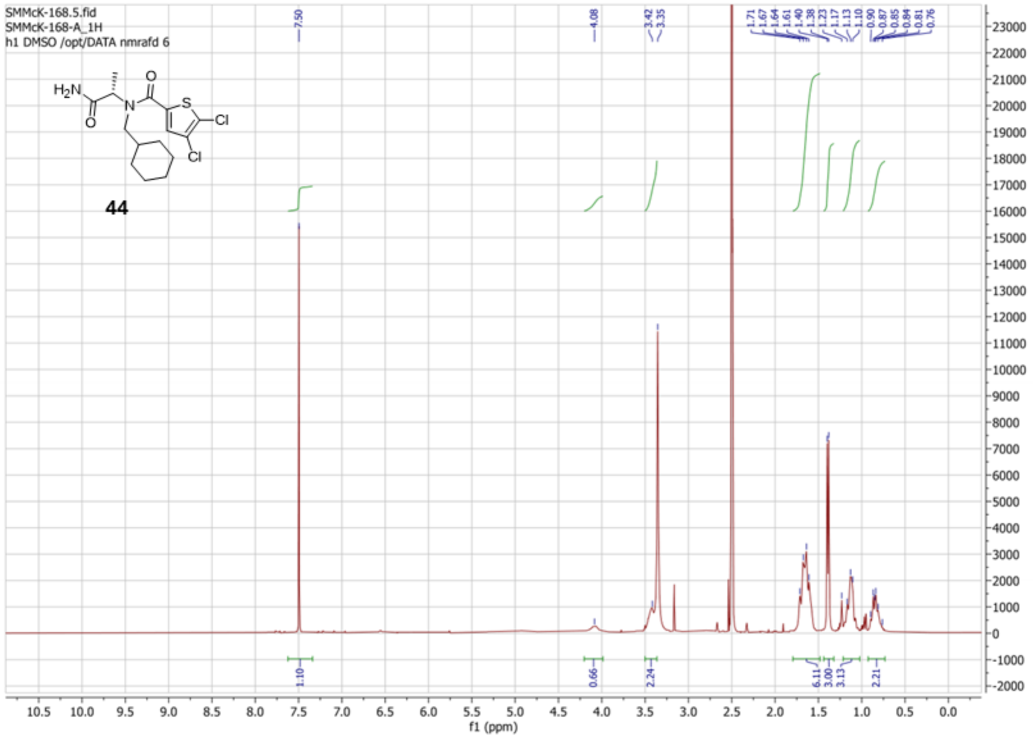

44 – ¹³C

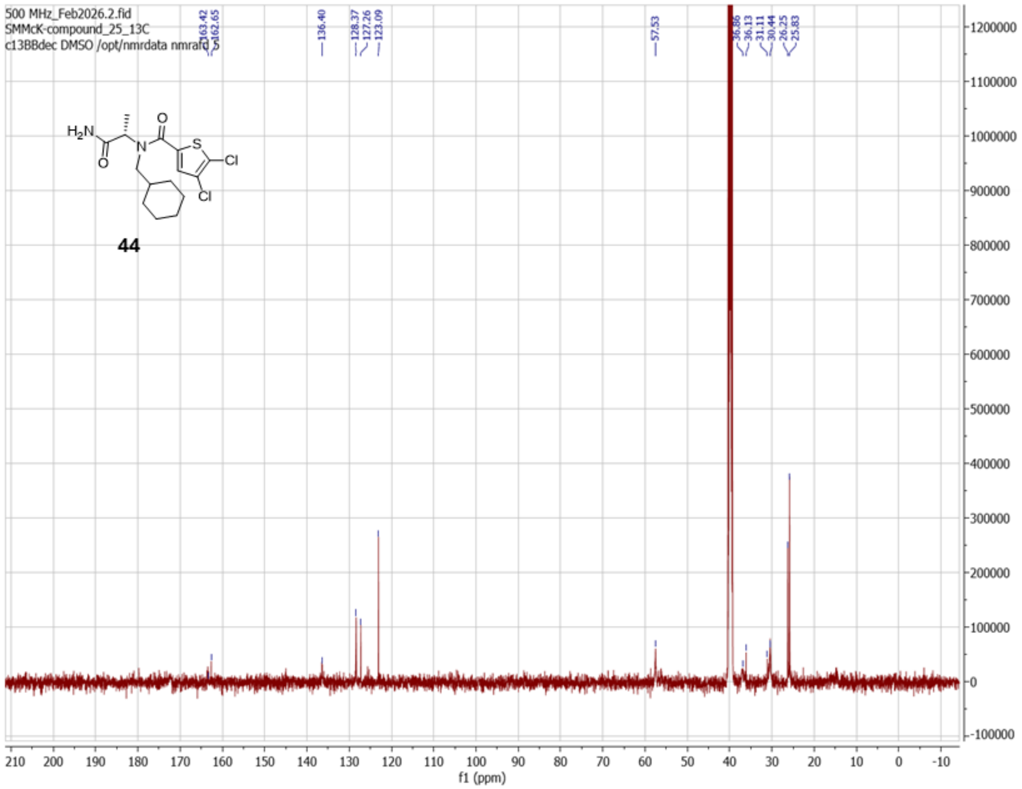

130 – ¹H

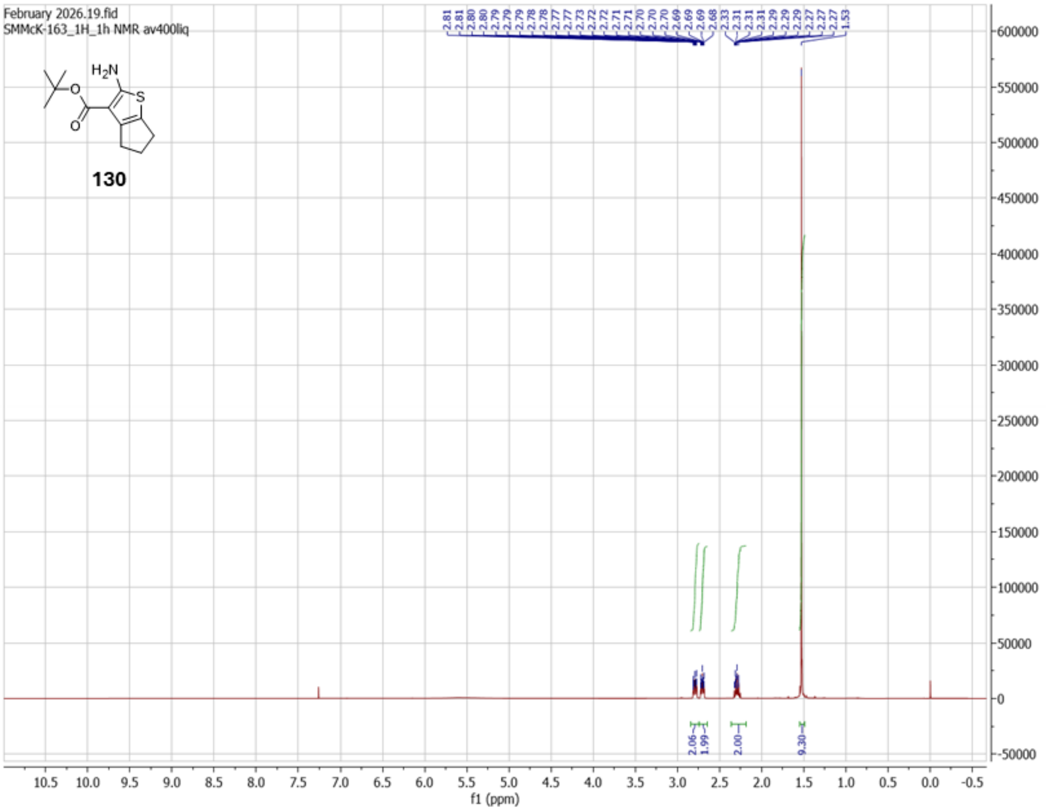

130 – ¹³C

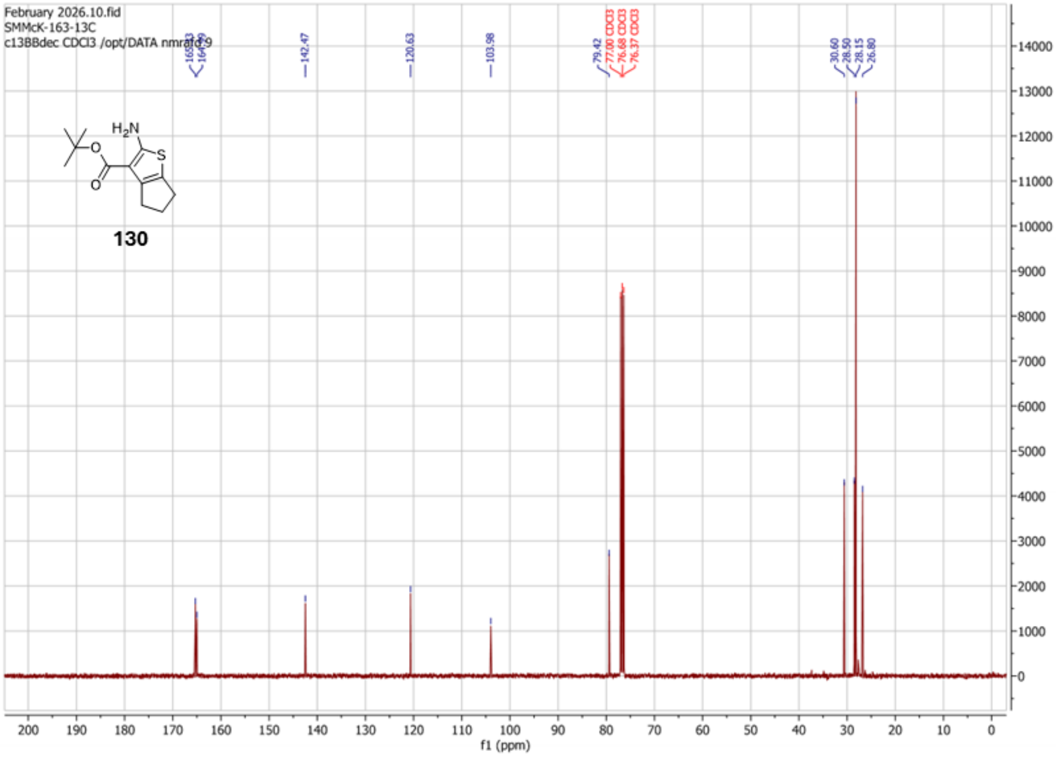

131 – ¹H

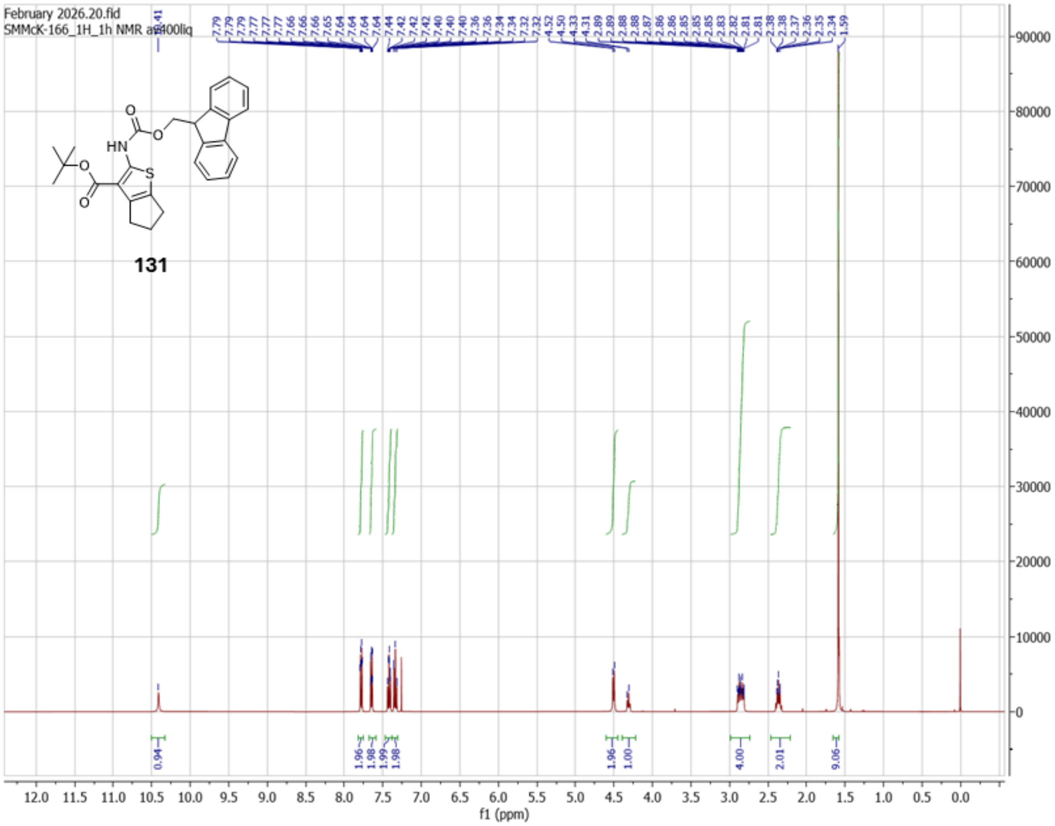

131 – ¹³C

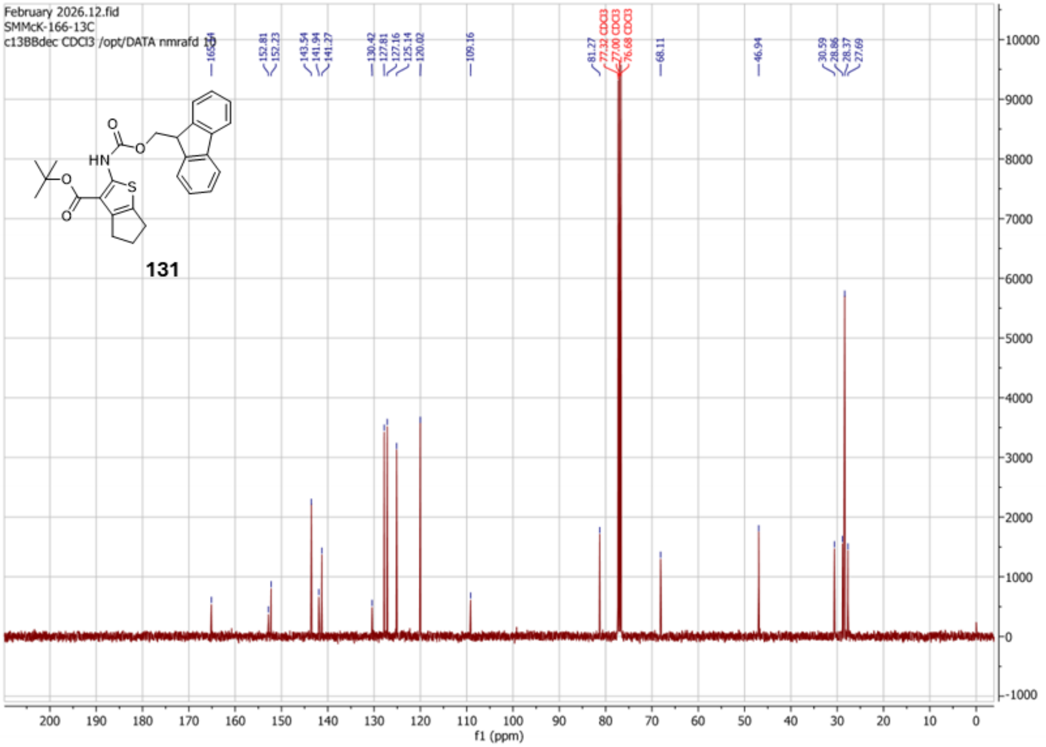

132 – ^{1}H

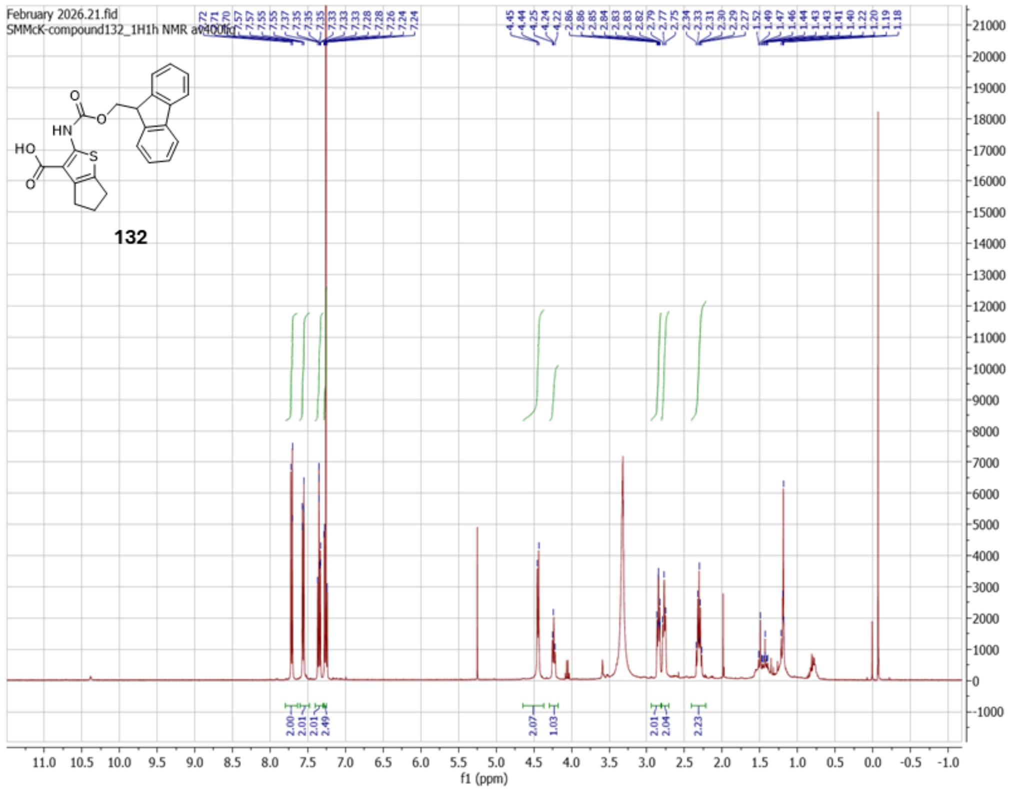

132 – ^{13}C

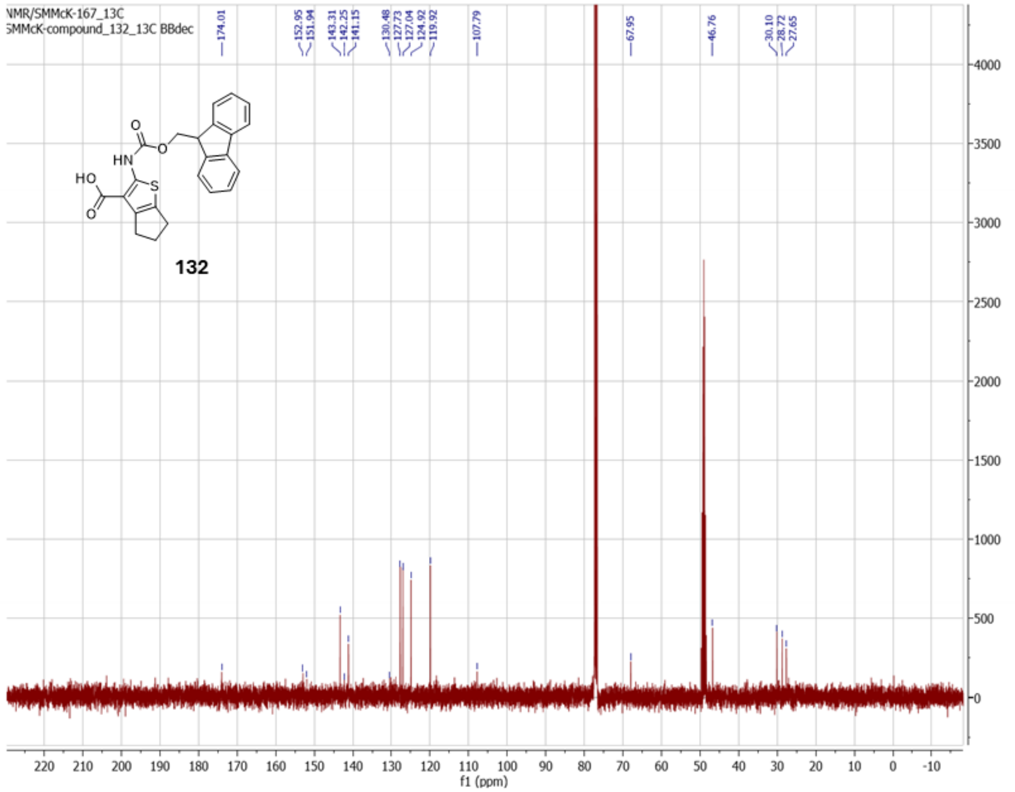

Biological Data

Binding Affinity Determination: Compounds 17-58

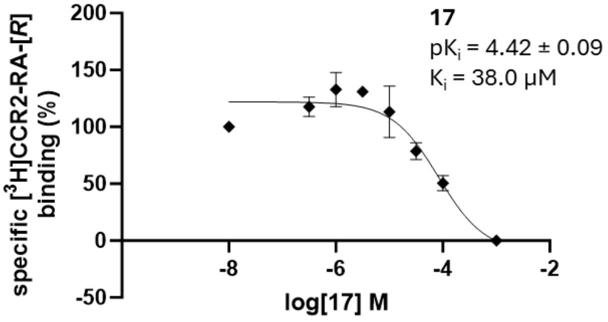

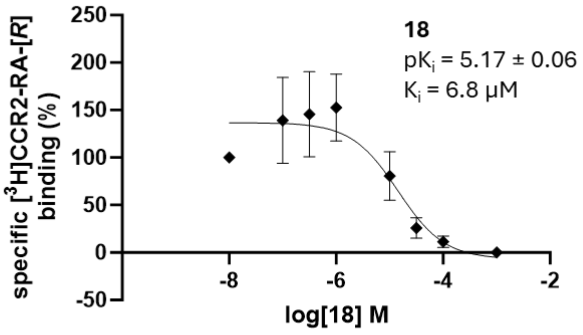

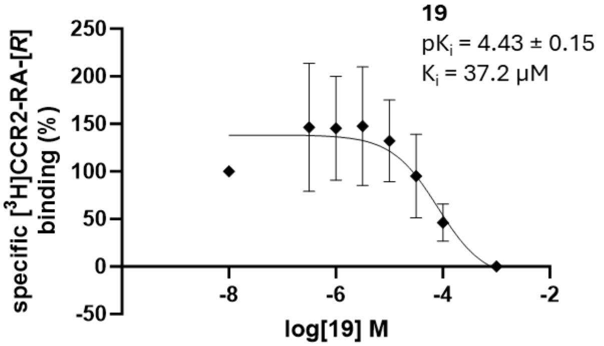

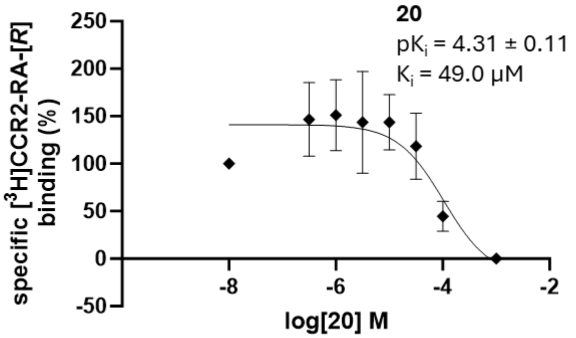

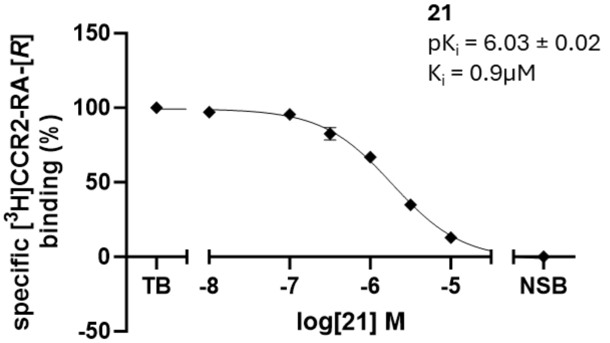

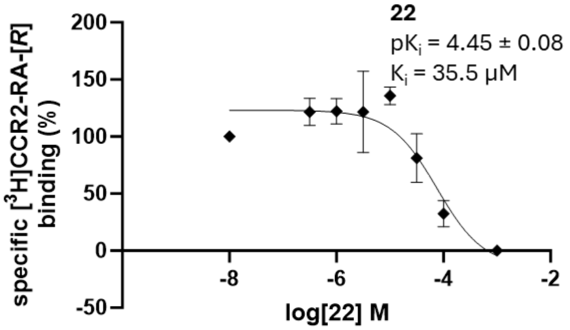

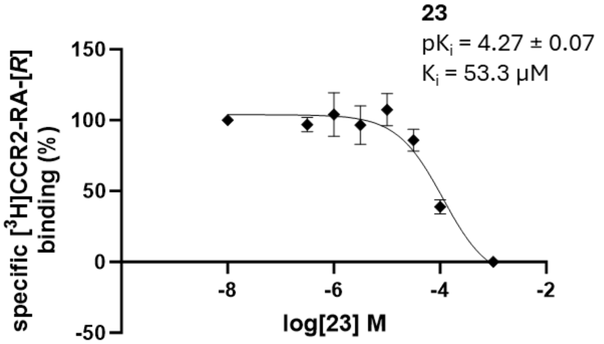

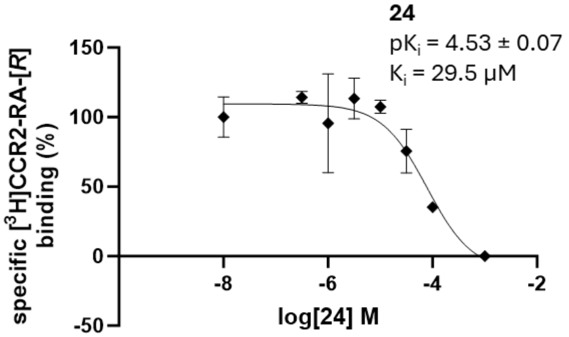

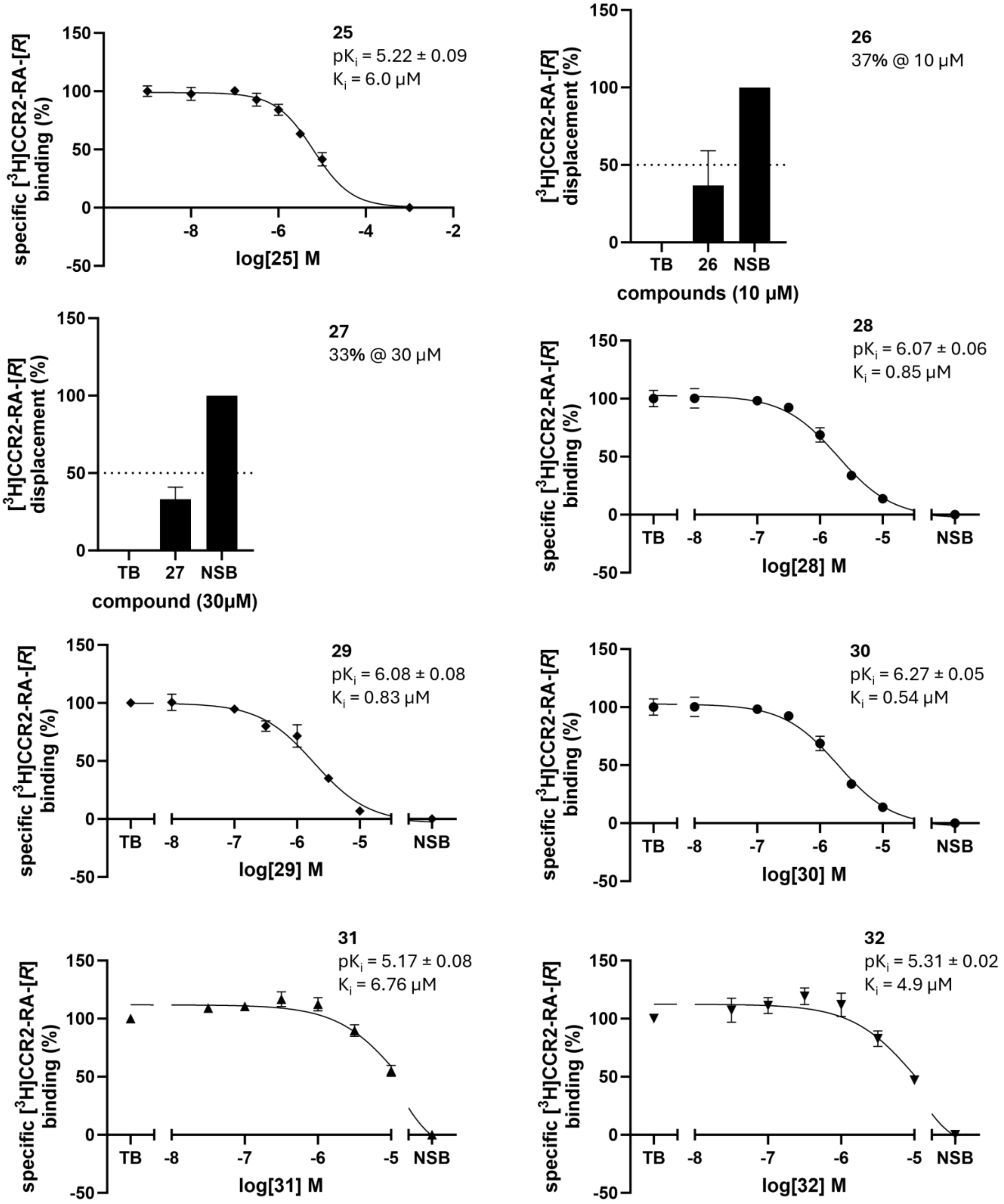
25
pK$_i$ = 5.22 ± 0.09
K$_i$ = 6.0 µM
specific [^{3}H]CCR2-RA-[*R*] binding (%)
log[25] M
26
37% @ 10 µM
[^{3}H]CCR2-RA-[*R*] displacement (%)
TB
26
NSB
compounds (10 µM)
27
33% @ 30 µM
[^{3}H]CCR2-RA-[*R*] displacement (%)
TB
27
NSB
compound (30µM)
28
pK$_i$ = 6.07 ± 0.06
K$_i$ = 0.85 µM
specific [^{3}H]CCR2-RA-[*R*] binding (%)
log[28] M
29
pK$_i$ = 6.08 ± 0.08
K$_i$ = 0.83 µM
specific [^{3}H]CCR2-RA-[*R*] binding (%)
log[29] M
30
pK$_i$ = 6.27 ± 0.05
K$_i$ = 0.54 µM
specific [^{3}H]CCR2-RA-[*R*] binding (%)
log[30] M
31
pK$_i$ = 5.17 ± 0.08
K$_i$ = 6.76 µM
specific [^{3}H]CCR2-RA-[*R*] binding (%)
log[31] M
32
pK$_i$ = 5.31 ± 0.02
K$_i$ = 4.9 µM
specific [^{3}H]CCR2-RA-[*R*] binding (%)
log[32] M

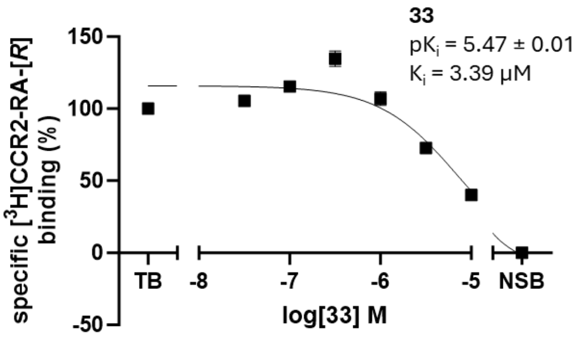
33
pK$_i$ = 5.47 ± 0.01
K$_i$ = 3.39 μM
specific [^{3}H]CCR2-RA-[R] binding (%)
150
100
50
0
-50
TB
-8
-7
-6
-5
NSB
log[33] M

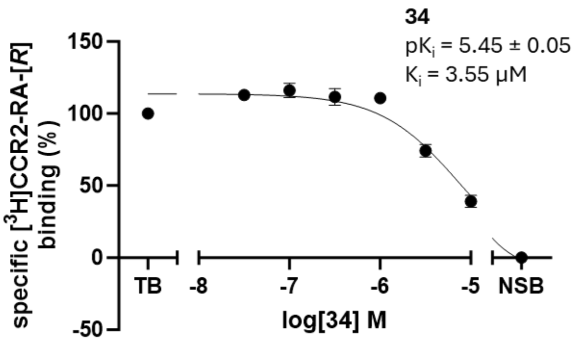
34
pK$_i$ = 5.45 ± 0.05
K$_i$ = 3.55 μM
specific [^{3}H]CCR2-RA-[R] binding (%)
150
100
50
0
-50
TB
-8
-7
-6
-5
NSB
log[34] M

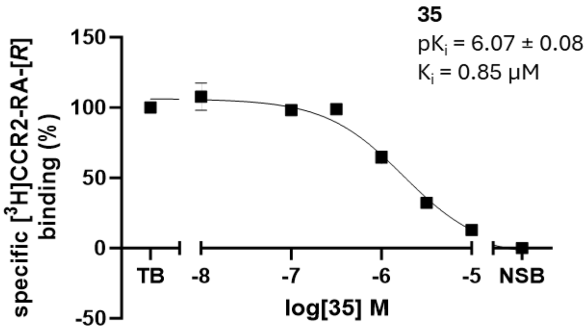
35
pK$_i$ = 6.07 ± 0.08
K$_i$ = 0.85 μM
specific [^{3}H]CCR2-RA-[R] binding (%)
150
100
50
0
-50
TB
-8
-7
-6
-5
NSB
log[35] M

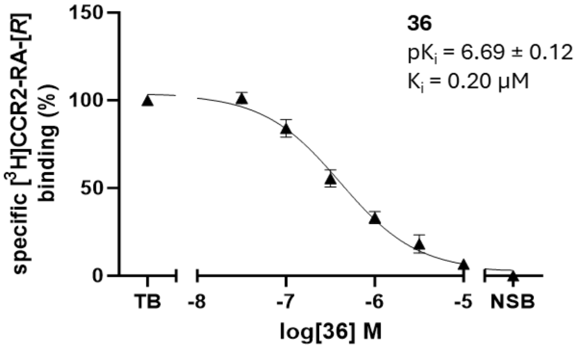
36
pK$_i$ = 6.69 ± 0.12
K$_i$ = 0.20 μM
specific [^{3}H]CCR2-RA-[R] binding (%)
150
100
50
0
TB
-8
-7
-6
-5
NSB
log[36] M

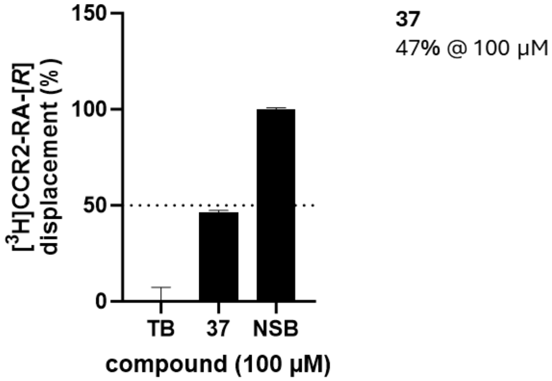
37
47% @ 100 μM
[^{3}H]CCR2-RA-[R] displacement (%)
150
100
50
0
TB
37
NSB
compound (100 μM)

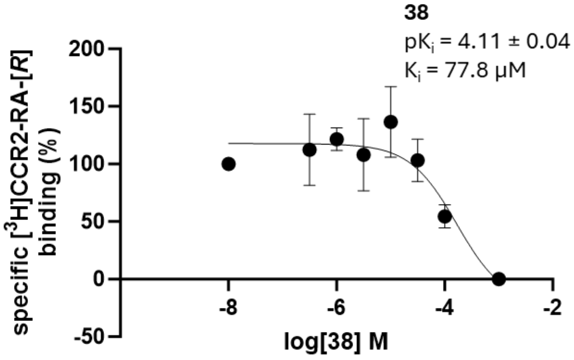
38
pK$_i$ = 4.11 ± 0.04
K$_i$ = 77.8 μM
specific [^{3}H]CCR2-RA-[R] binding (%)
200
150
100
50
0
-50
-8
-6
-4
-2
log[38] M

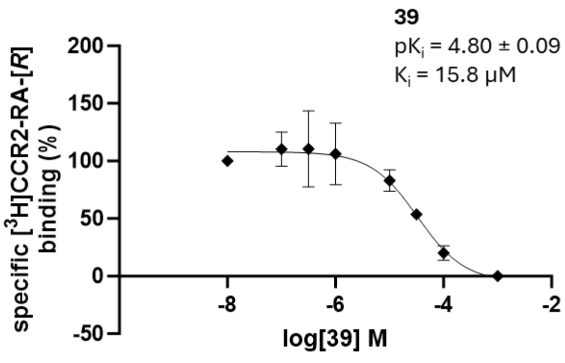
39
pK$_i$ = 4.80 ± 0.09
K$_i$ = 15.8 μM
specific [^{3}H]CCR2-RA-[R] binding (%)
200
150
100
50
0
-50
-8
-6
-4
-2
log[39] M

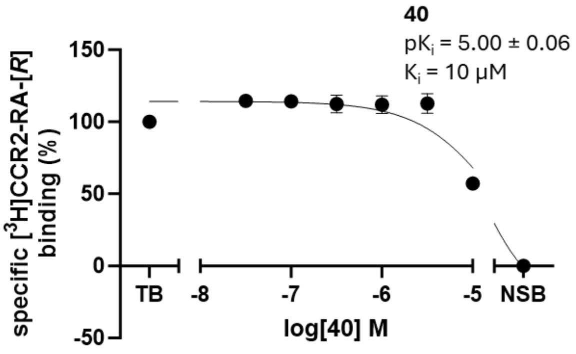
40
pK$_i$ = 5.00 ± 0.06
K$_i$ = 10 μM
specific [^{3}H]CCR2-RA-[R] binding (%)
150
100
50
0
-50
TB
-8
-7
-6
-5
NSB
log[40] M

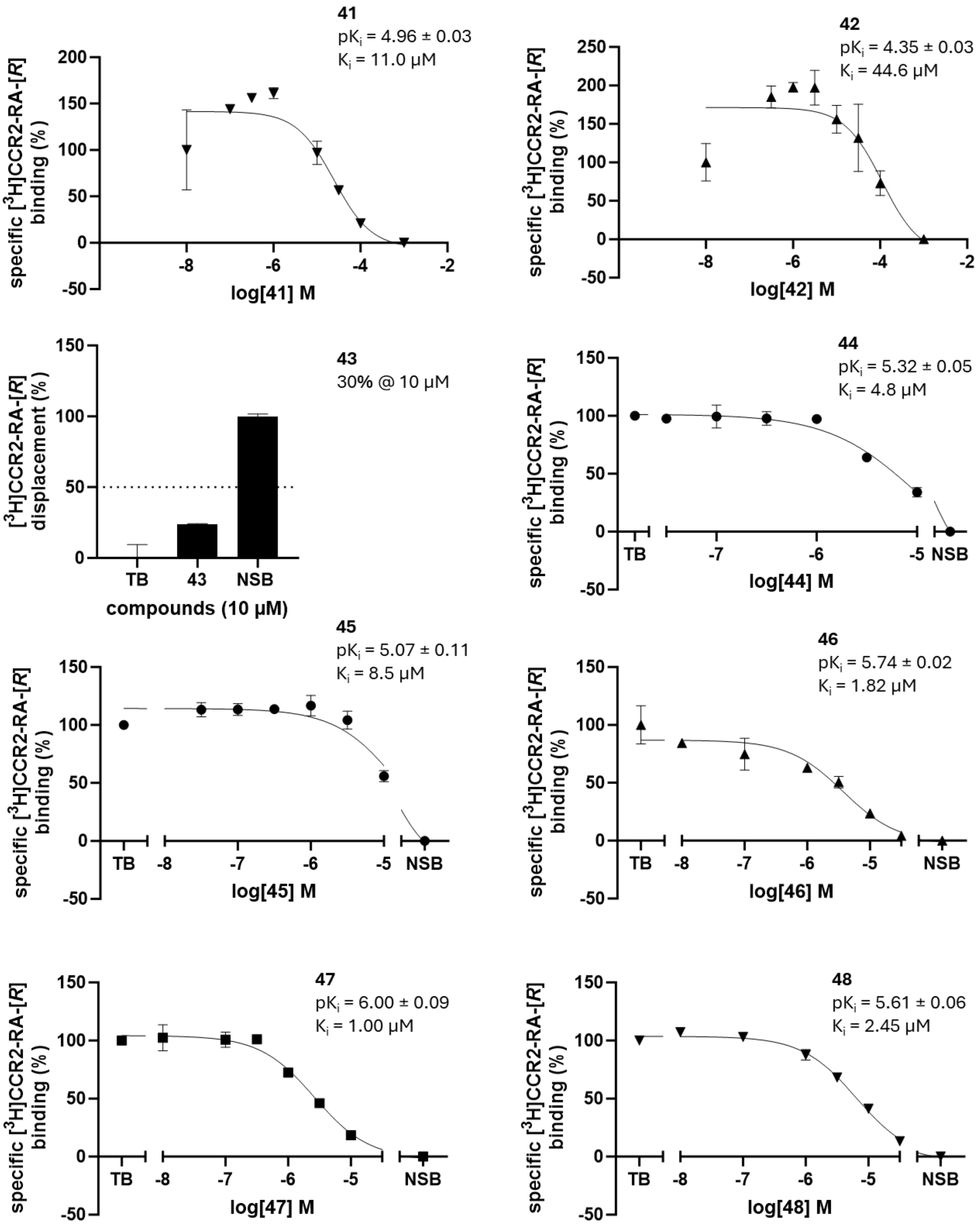
41
$pK_i = 4.96 \pm 0.03$
$K_i = 11.0\ \mu M$
specific [3H]CCR2-RA-[R] binding (%)
log[41] M
42
$pK_i = 4.35 \pm 0.03$
$K_i = 44.6\ \mu M$
specific [3H]CCR2-RA-[R] binding (%)
log[42] M
43
30% @ 10 μM
[3H]CCR2-RA-[R] displacement (%)
TB
43
NSB
compounds (10 μM)
44
$pK_i = 5.32 \pm 0.05$
$K_i = 4.8\ \mu M$
specific [3H]CCR2-RA-[R] binding (%)
log[44] M
45
$pK_i = 5.07 \pm 0.11$
$K_i = 8.5\ \mu M$
specific [3H]CCR2-RA-[R] binding (%)
log[45] M
46
$pK_i = 5.74 \pm 0.02$
$K_i = 1.82\ \mu M$
specific [3H]CCR2-RA-[R] binding (%)
log[46] M
47
$pK_i = 6.00 \pm 0.09$
$K_i = 1.00\ \mu M$
specific [3H]CCR2-RA-[R] binding (%)
log[47] M
48
$pK_i = 5.61 \pm 0.06$
$K_i = 2.45\ \mu M$
specific [3H]CCR2-RA-[R] binding (%)
log[48] M

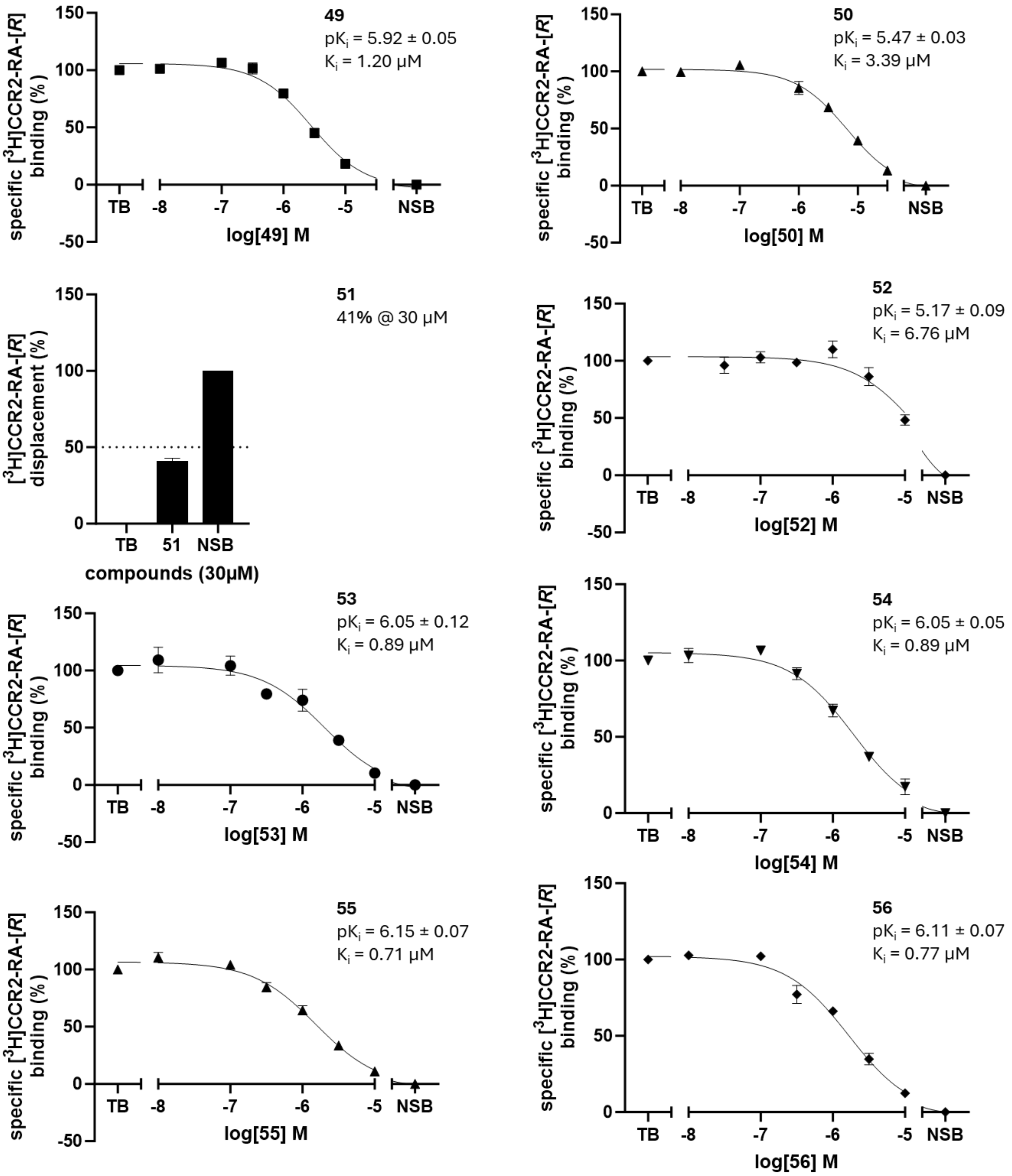
49
pKi = 5.92 ± 0.05
Ki = 1.20 µM
specific [3H]CCR2-RA-[R] binding (%)
150
100
50
0
-50
TB
-8
-7
-6
-5
NSB
log[49] M
50
pKi = 5.47 ± 0.03
Ki = 3.39 µM
log[50] M
51
41% @ 30 µM
[3H]CCR2-RA-[R] displacement (%)
TB
51
NSB
compounds (30µM)
52
pKi = 5.17 ± 0.09
Ki = 6.76 µM
log[52] M
53
pKi = 6.05 ± 0.12
Ki = 0.89 µM
log[53] M
54
pKi = 6.05 ± 0.05
Ki = 0.89 µM
log[54] M
55
pKi = 6.15 ± 0.07
Ki = 0.71 µM
log[55] M
56
pKi = 6.11 ± 0.07
Ki = 0.77 µM
log[56] M

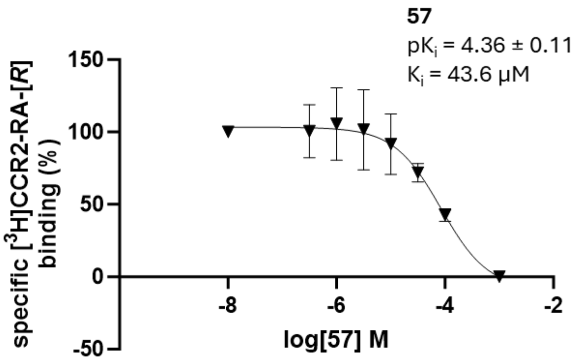

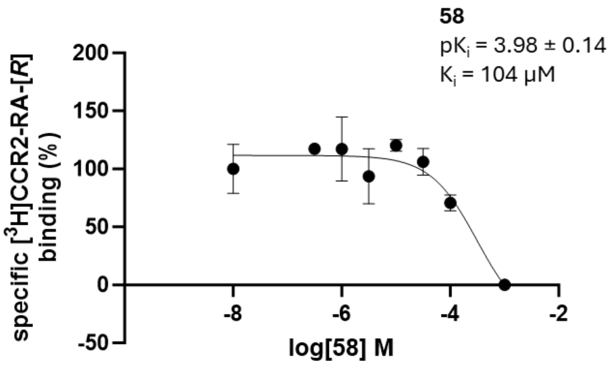

K$_i$ Determination: High-purity Compounds 36 & 44

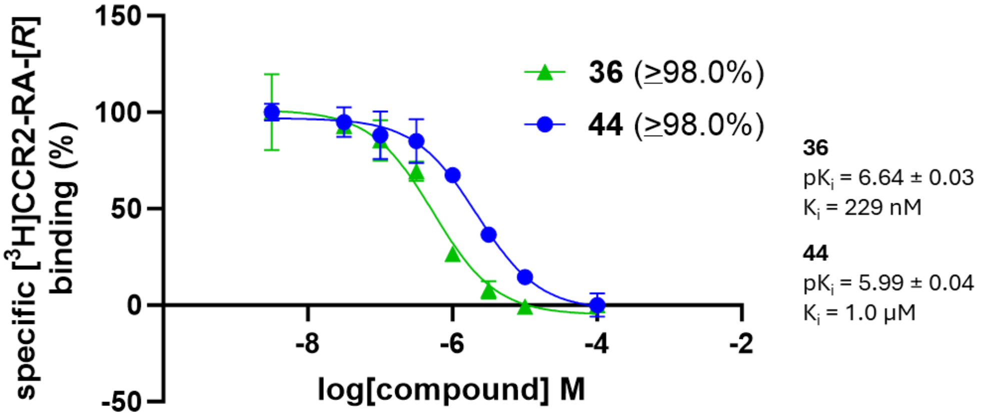

β-Arrestin Recruitment IC_{50} Determination for 36 and 44

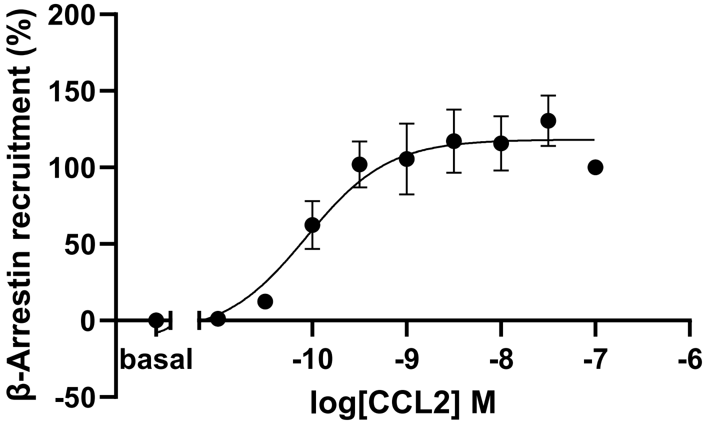

β-arrestin recruitment in U2OS-CCR2 cells by increasing concentrations CCL2 to determine the EC_{80} concentration required for the antagonist assay. Data is presented as mean ± SEM of three independent experiments performed in duplicate. Data is normalized where basal activity is set to 0% and 100 nM CCL2 to 100%.

	CCL2
**pEC_{50} ± SEM**	10.02 ± 0.09
**EC_{50} (nM)**	0.096
**EC_{80} (nM)**	0.384

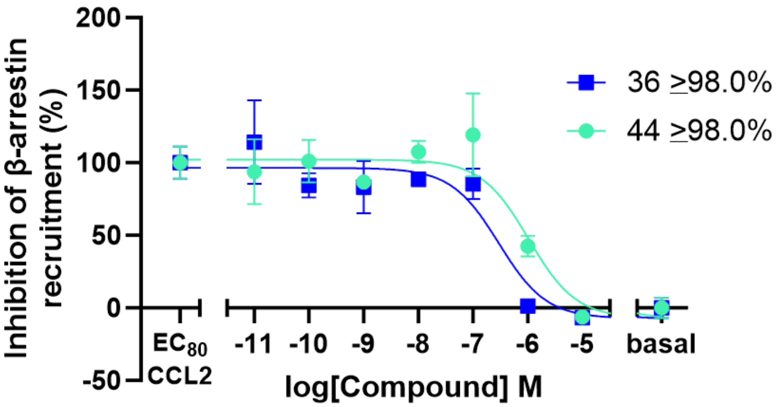

36
pIC_{50} = 6.59 ± 0.05
IC_{50} = 257 nM

44
pIC_{50} = 5.88 ± 0.08
IC_{50} = 1.0 μM

Inhibition of CCL2 activated β-arrestin recruitment in U2OS-CCR2 cells by increasing concentrations of 36 and 44 after stimulation with an EC_{80} concentration CCL2 (0.384 nM). Data is presented as mean ± SEM of three independent experiments performed in duplicate. Data is normalized where basal activity is set to 0% and the EC_{80} concentration CCL2 without compounds is set to 100%.

References

1. Goldberg, F. W., Kettle, J. G., Kogej, T., Perry, M. W. D. & Tomkinson, N. P. Designing novel building blocks is an overlooked strategy to improve compound quality. *Drug Discovery Today* **20**, 11–17 (2015).
2. CCR2 - C-C chemokine receptor type 2 - Homo sapiens (Human) | UniProtKB | UniProt. https://www.uniprot.org/uniprotkb/P41597/entry#sequences\.
3. GitHub - CDDLeiden/combinaut. https://github.com/CDDLeiden/combinaut/tree/main.
4. Ortiz Zacarías, N. V. *et al.* Synthesis and Pharmacological Evaluation of Triazolopyrimidinone Derivatives as Noncompetitive, Intracellular Antagonists for CC Chemokine Receptors 2 and 5. *J. Med. Chem.* **62**, 11035–11053 (2019).
5. Ortiz Zacarías, N. V. *et al.* Pyrrolone Derivatives as Intracellular Allosteric Modulators for Chemokine Receptors: Selective and Dual-Targeting Inhibitors of CC Chemokine Receptors 1 and 2. *J. Med. Chem.* **61**, 9146–9161 (2018).
